# Arenium cation or radical cation? An insight into the cyclodehydrogenation reaction of 2-substituted binaphthyls mediated by Lewis acids†

**DOI:** 10.1039/d0ra04213g

**Published:** 2020-06-09

**Authors:** Patricia Camargo Solórzano, María T. Baumgartner, Marcelo Puiatti, Liliana B. Jimenez

**Affiliations:** INFIQC, Departamento de Química Orgánica, Facultad de Ciencias Químicas, Universidad Nacional de Córdoba, Ciudad Universitaria X5000HUA Córdoba Argentina mpuiatti@fcq.unc.edu.ar ljimenez@fcq.unc.edu.ar +54-351-5353867 int. 53330

## Abstract

Perylene and its derivatives are some of the most interesting chromophores in the field of molecular design. One of the most employed methodologies for their synthesis is the cyclodehydrogenation of binaphthyls mediated by Lewis acids. In this article, we investigated the cyclodehydrogenation reaction of 2-substituted binaphthyls to afford the *bay*-substituted perylene. By using AlCl_3_ as a Lewis acid and high temperatures (the Scholl reaction), two new products bearing NH_2_ and N(CH_3_)_2_ groups at position 2 of the perylene ring were synthesized. Under these conditions, we were also able to obtain terrylene from ternaphthalene in 38% yield after two cyclodehydrogenation reactions in a single step. The attempts to promote the formation of a radical cation (necessary intermediary for the oxidative aromatic coupling mechanism) by using FeCl_3_ or a strong oxidant like 2,3-dichloro-5,6-dicyano-1,4-benzoquinone (DDQ) did not yield the expected products. DFT calculations suggested that the lack of reaction for oxidative aromatic coupling is caused by the difference between the oxidation potentials of the donor/acceptor couple. In the case of the Scholl reaction, the regiochemistry involved in the formation of the σ-complex together with the activation energy of the C–C coupling reaction helped to explain the differences in the reactivity of the different substrates studied.

## Abstract 2

Cyclodehydrogenation reactions of 2-substituted binaphthyls induced by a Lewis acid. Synthesis and theoretical studies of the reaction mechanisms.

## Graphical abstract

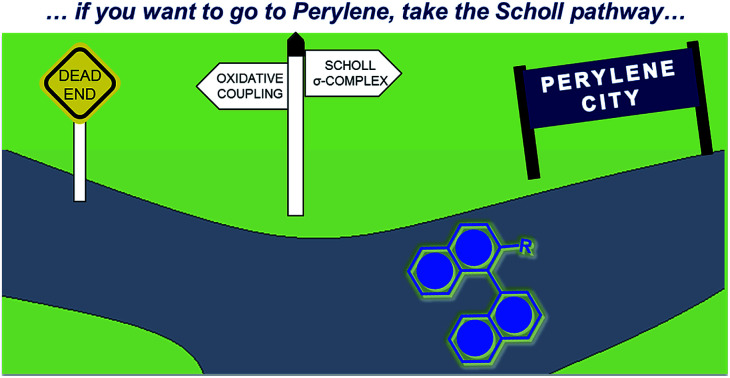

## Introduction

In the last decades, there has been special interest in the design of specific chromophoric structures due to their possible application in molecular devices such as light-collecting antennas and organic light emitting diodes (OLEDs), among other applications.^1^ Among these structures, perylene and its derivatives are particularly interesting, since they exhibit excellent electronic and optical properties.^2^ Perylene shows characteristic fluorescence with high quantum yield, which varies depending on both the nature of the substituents attached to the polycycle and their positions (*e.g.* the axial or equatorial regions).^3^ Recent applications of perylene derivatives (mostly perylene diimides, PDIs),^4^ include organic field effect transistors,^5^ organic photovoltaic cells,^6^ optical switches^7^ and molecular wires.^8^

**Scheme 1:**
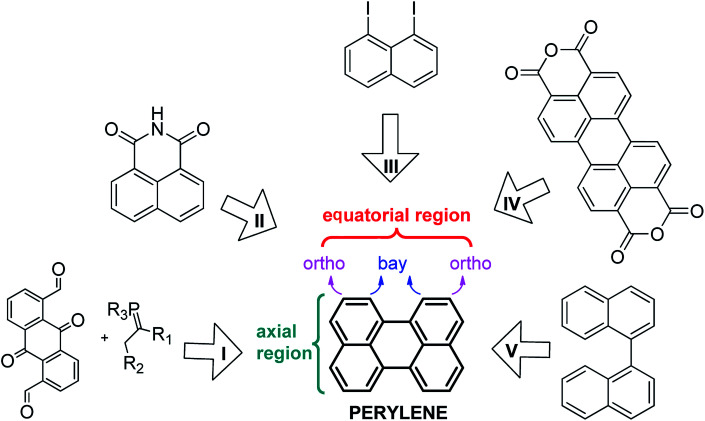
Retrosynthesis of perylene. Route: (I) intramolecular condensation of quinone derivatives; (II) dimerization of benzoisoquinoline-diones in basic media; (III) Ullmann reaction; (IV) decarboxylation of the perylene tetracarboxylic dianhydride mediated by basic conditions and high temperatures and (V) cyclodehydrogenation of the binaphthalene.

Because of the importance of perylene and its derivatives, different strategies have been developed for their synthesis (Scheme 1, I–V). Among these routes are intramolecular condensation of quinone derivatives (I),^9^ base-induced dimerization of benzoisoquinoline-diones (II),^10^ the Ullmann reaction (III),^11^ decarboxylation of the perylene-3,4,9,10-tetracarboxylic dianhydride mediated by basic conditions (IV),^12^ and the cyclodehydrogenation of the binaphthalene (V).^13^ In general, the yields of these reactions are good to low. Perhaps, the best option to obtain large quantities of perylene is route IV, but it usually requires high temperatures (more than 350 °C). On the other hand, route V is an attractive option, since it can take place through up to three different mechanisms depending on the reactants and the experimental conditions used. For example, Rickhaus *et al.*^13*d*^ were able to obtain quantitative yields of perylene by using an excess of electrons (route V), however no *bay*-substituted perylene was reported following this last one. Despite these recent advances, the synthetic procedures to generate perylene are currently limited. Furthermore, only routes I and II can yield substituted perylenes; *peri*-substituted (axial region) by route II and *ortho*- and *bay*-substituted by route I.

**Scheme 2:**
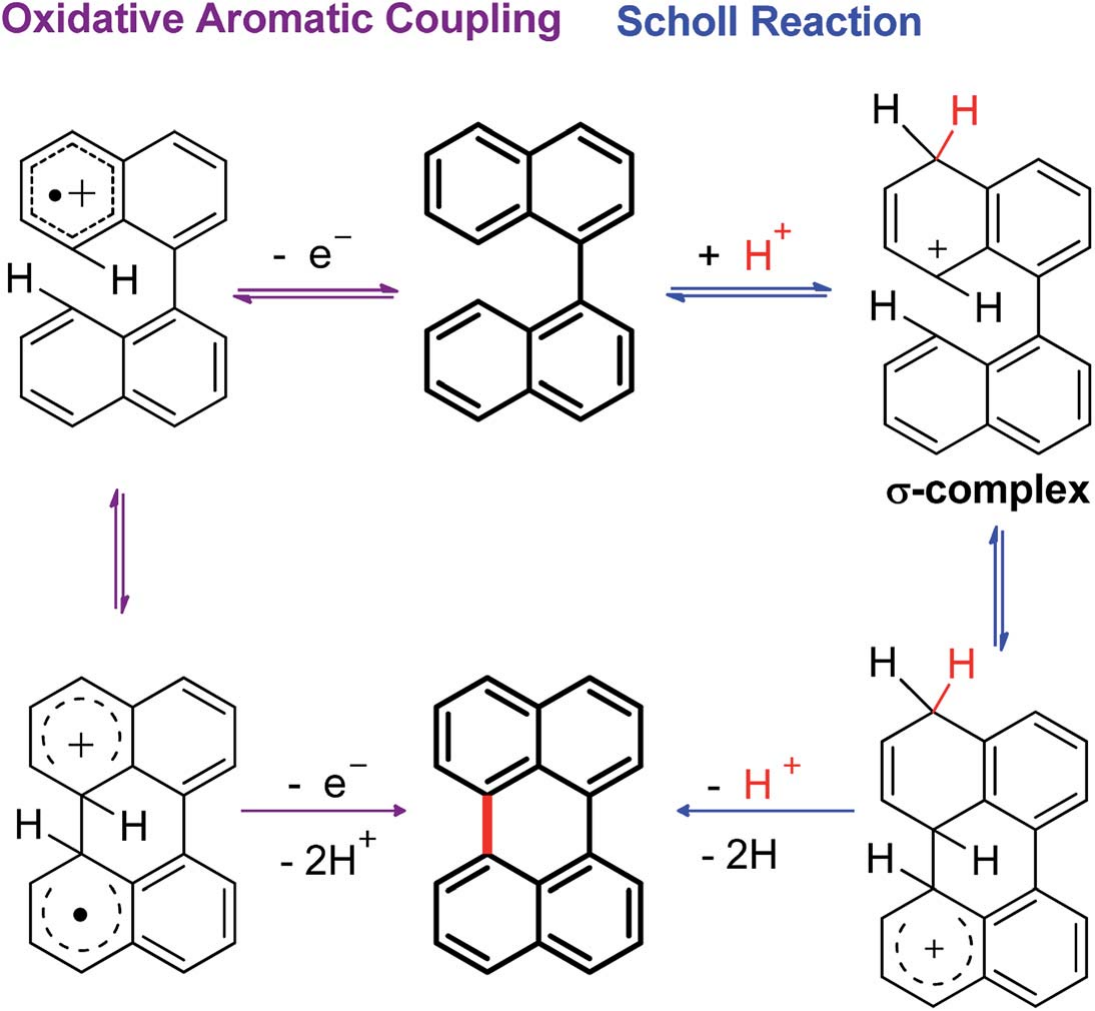
General mechanisms proposed for the cyclodehydrogenation reaction in oxidizing media (mediated by Lewis acids).

Although the Lewis acid-mediated cyclodehydrogenation (another way by which route V can occur) is one of the oldest methodologies used to obtain polycyclic aromatic hydrocarbons,^14^*i.e.* perylene,^13*b*,15^ terrylene,^16^ and dibenzotetraphenylperiflanthene,^17^ the operating mechanism presents some ambiguities. In front of this discussion, Gryko *et al.*^18^ proposed the distinction between two possible mechanisms through which polycyclic aromatic products can be generated. One of the postulated mechanisms is known as the Scholl reaction, that proceeds through the formation of an electrophilic complex or σ-complex (shown as H^+^ for simplicity in Scheme 2, but it could also be a σ-complex formed with a Lewis acid).^18^ Usually, methodologies related to this mechanism involve the use of strong Lewis acids such as AlCl_3_ dissolved in chlorobenzene,^19^ complexes of fused salts such as AlCl_3_/NaCl^20^ or AlCl_3_/SO_2_,^21^ among others.^15^

In contrast, the other proposed mechanism for the reactions mediated by Lewis acids is the oxidative aromatic coupling. This mechanism involves the formation of a radical cation, followed by an intramolecular cyclization and rearomatization to give the polycyclic product (Scheme 2, left side).^18^ The experimental conditions to carry out this reaction involve the use of either Lewis acids such as FeCl_3_ (ref. 22) or strong oxidants like 2,3-dichloro-5,6-dicyano-1,4-benzoquinone (DDQ).^23^

The ambiguity of these reactions relies on the fact that sometimes there is no clear evidence of the operating mechanism, and they are said to occur through the Scholl mechanism without any discrimination. One of the reasons contributing to this confusion is the fact that most of the Lewis acids used in the Scholl reaction are also mild oxidants commonly employed in oxidative aromatic coupling reactions.^22,24^ In this regard, in some cases DFT calculations have been used to help to solve this issue.^25^

**Scheme 3:**
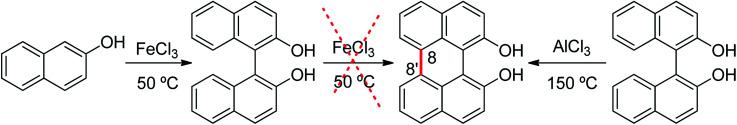
Formation of perylene-1,12-diol.

A prominent example in which it is possible to distinguish between oxidative aromatic coupling and Scholl mechanism is the reaction of 2-naphthol in the presence of an oxidant such as FeCl_3_ at 50 °C.^26^ Under these conditions, 2,2′-dihydroxy-1,1′-binaphthalene is formed by an oxidative aromatic coupling reaction (Scheme 3). However, it is not possible to obtain the perylene-1,12-diol compound, even when additional portions of FeCl_3_ were added to the system.^18^ In contrast, the C–C bond formation (C-8 with C-8′) to give the perylene-1,12-diol can be achieved by heating 2,2′-dihydroxy-1,1′-binaphthalene with AlCl_3_ at 150 °C (Scholl conditions).^27^

A similar behavior was also observed with naphthyl isoquinolines.^13*e*^ The compound 1-(naphthalen-1-yl)-isoquinoline reacts with a melted mixture of AlCl_3_/NaCl at 160 °C to provide 1-azaperylene in 68% yield, but cyclization does not occur when 8-(naphthalen-1-yl)-isoquinoline is exposed to the same conditions. Moreover, neither of the two isomeric naphthyl isoquinoline substrates react in the presence of FeCl_3_ at 25 °C and 80 °C.^18^

Considering the aforementioned antecedents, 2-substituted binaphthalenes^28^ were synthesized and used as substrates for the preparation of equatorially functionalized perylenes. The study of the reactivity of these binaphthyl compounds can generate important contributions to the understanding of mechanisms associated with cyclodehydrogenation reactions mediated by Lewis acids. A comparison between the arenium cation (σ-complex or Scholl) mechanism and the cyclodehydrogenation initiated by radical cations (oxidative aromatic coupling) is presented for all synthesized compounds. The discussion is complemented with DFT computational studies. Furthermore, the peculiar formation of two C–C bonds in a single reaction step is presented in the synthesis of terrylene (tribenzo[*de*,*kl*,*rst*]pentaphene) from 1,1′:4′,1′′-ternaphthalene.

## Results and discussion

### Scholl and oxidative aromatic coupling experimental reactions

In an initial approach, the intramolecular cyclization of the substrates [1,1′-binaphthalen]-2-amine (1), *N*,*N*-dimethyl-[1,1′-binaphthalen]-2-amine (2) and 2-methoxy-1,1′-binaphthalene (3) was experimentally evaluated following both reaction conditions, Scholl (Table 1) and oxidative aromatic coupling.

**Table 1:** Results of the Scholl reaction for the substrates 1–3

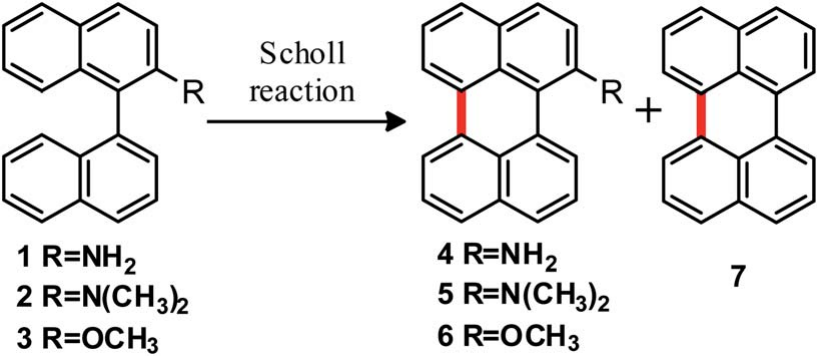				
Entry	Scholl reaction^a^	Products^b^ (%)		Remaining substrate^c^ (%)
1	AlCl_3_/NaCl, 170 °C, 15 min	4 (27)^c^	7 (21)	1 (12)
2	AlCl_3_/NaCl, 170 °C, 30 min	4 (12)^c^	7 (39)	1 (<5)
3	AlCl_3_/NaCl, 170 °C, 10 min	5 (50)	7 (12)	2 (<5)
4	AlCl_3_/NaCl, 170 °C, 20 min	5 (40)	7 (20)	2 (<5)
5^d^	AlCl_3_/C_6_H_5_Cl, 80 °C, 6 h	6 (—)	7 (—)	3 (<5)

a AlCl_3_/NaCl (5 : 1 eq.), substrate (0.2 eq., 0.1 mmol).

b Isolated yields.

c Quantified by CG-FID using the internal standard method.

d Substrate (0.2 mmol), AlCl_3_ (1.8 mmol), C_6_H_5_Cl (10 mL).

The substrates were allowed to react at 170 °C within the salt mixture of AlCl_3_/NaCl during different reaction times (Table 1, entries 1–4). Compounds 1 and 2 afforded the cyclized products 4 and 5, respectively. To the best of our knowledge, this work describes a synthetic protocol for obtaining products 4 and 5 for the first time, since although compound 4 is mentioned in some patents, no data on the synthetic procedure or the reaction performance are known. Compound 4 decomposes under air and during purification in column chromatography (on silica gel). On the other hand, compound 5 has never been reported in literature and showed high fluorescence (*ε*_5_ (455 nm) = 6869 M^−1^ cm^−1^; *Φ*_f_ (5) = 0.92 in dichloromethane).^30^ In all cases, perylene (7) was found to be the main side product, in addition to substrate that did not react.

It is known that 1,1′-binaphthalene isomerizes to 1,2′-binaphthalene under acid-catalyzed conditions, as a consequence of the formation of an *ipso*-arenium ion. This process competes with the cyclodehydrogenation to form perylene;^31^ for example, formation of benzo[*j*]fluoranthene and related compounds is observed when isomerization occurs.^31*a*^ In our case, the C-2′ position is blocked by the amino substituent, precluding isomerization process. In consequence, no isomerization-derived products were observed in the assayed reactions.

On the other hand, we assume that the formation of 7 could come from deamination of the products 4 or 5 due to the strongly acidic and high temperature conditions. This hypothesis is supported by the fact that an increase of reaction time leads to an increase in perylene (7) yield together with a decrease of the amino-perylene product yield (entries 2 and 4, Table 1). Although there exist several possible mechanisms for deamination of different amino compounds,^32^ at this stage we cannot unequivocally choose the mechanism involved in our case.

Substrate 3 has a melting point of 109–110 °C and decomposes at 125–127 °C.^33^ For this reason, milder Scholl reaction conditions were assayed with this substrate.^19^ A solution of AlCl_3_ in dry chlorobenzene at 80 °C was used to promote the reaction (entry 5, Table 1); however, no intramolecular cyclization product was observed and 2-methoxynaphthalene was obtained as major product, due to the C1–C1′ bond cleavage of the substrate. Some minor products from coupling between naphthyl group and chlorobenzene, homocoupling of chlorobenzene and other chlorinated compounds were detected by GC-MS.

The intramolecular cyclization by oxidative aromatic coupling conditions was evaluated by carrying out the reaction of substrates 1–3 with triflic acid (TfOH) and DDQ in dry dichloromethane for 1 hour at 0 °C. Under these conditions, the formation of cyclized products was not observed and the starting materials were fully recovered. Johnson *et al.* reported that 1,1′-binaphthalene in presence of TfOH at room temperature mostly produces the 2,2′-binaphthyl as a consequence of rearrangements;^31*a*^ nevertheless, this isomerization reaction is not possible for our 2-substituted compounds. As an alternative methodology, solutions of 1–3 in dry dichloromethane were treated with a solution of FeCl_3_ in CH_3_NO_2_,^16*a*^ but there was not substrate conversion at all.

To sum up, substrates 1 and 2 reacted in the Scholl reaction conditions, to produce the cyclized products of interest. Substrate 3 reacts by the Scholl pathway, but leading to no cyclodehydrogenation compounds. None of these substrates would react by the radical cation pathway (oxidative aromatic coupling).

### Computational modeling

In order to find the nature of the difference in reactivity of the substrates, in both the Scholl and oxidative aromatic coupling conditions, molecular modeling studies based on Density Functional Theory (DFT)^34^ were done. All calculations reported here were carried out at the B3PW91/6-31+G* level of theory.^35^ The polarizable continuum model (IEFPCM) was employed to assess solvation using two media with different dielectric constant: dichloromethane (*ε* ∼ 8.93) and *N*-methylformamide (*ε* ∼ 181.5). Both solvent models afforded similar results, for simplicity the ones obtained in the latter solvent are included in the main text.^36^ The two possible mechanisms were evaluated, using also reference compounds selected from bibliographic data, which react, or not, within the same conditions as those employed within this work.

### Scholl reaction

Firstly, we address the following question, is it possible that – under Scholl reaction conditions – the substrates 1, 2 and 3 may react *via* the formation of the σ-complex? Two reference compounds were now included, 1-(naphthalen-1-yl)isoquinoline (8) and 8-(naphthalen-1-yl)isoquinoline (9).^18^ From these compounds, 8 reacted by the Scholl experimental condition whereas 9 could not form the cyclized product; they should work as positive and negative tests, respectively.

Since the reactions were carried out in acidic media, the formation of the arenium cation was the first evaluated process. To study the regiochemistry of the protonation of each substrate, the relative energy difference required to form the σ-complex at different positions of the aromatic rings was calculated. A Boltzmann population analysis for the formation of the σ-complex was estimated at 170 °C (see ESI†), the most favored positions are shown in Table 2.^29^ It should be noted that the amino group must be protonated due to the acidic media used in the reactions. In this way, the cation of the substrate 1 (1a) was used,^37^ as well as the isoquinolinium cations (8a and 9a) which come from the protonation of the reference compounds 8 and 9.

**Table 2:** Most probable positions for the protonation to form the σ-complex for 1a, 3, 8a and 9a

Substrates		Reference compounds	
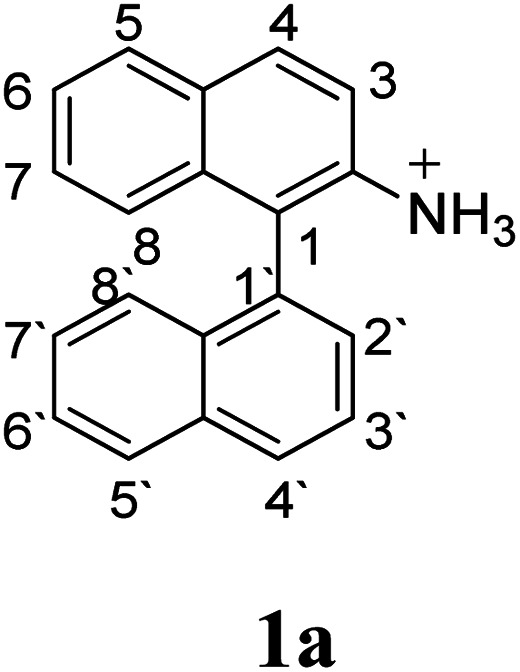	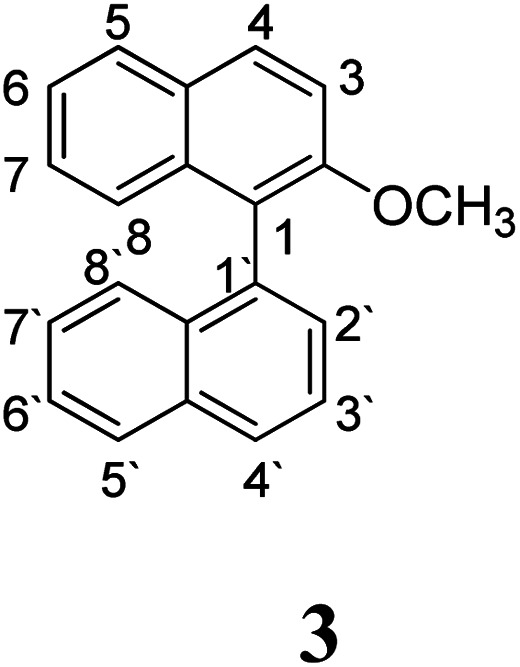	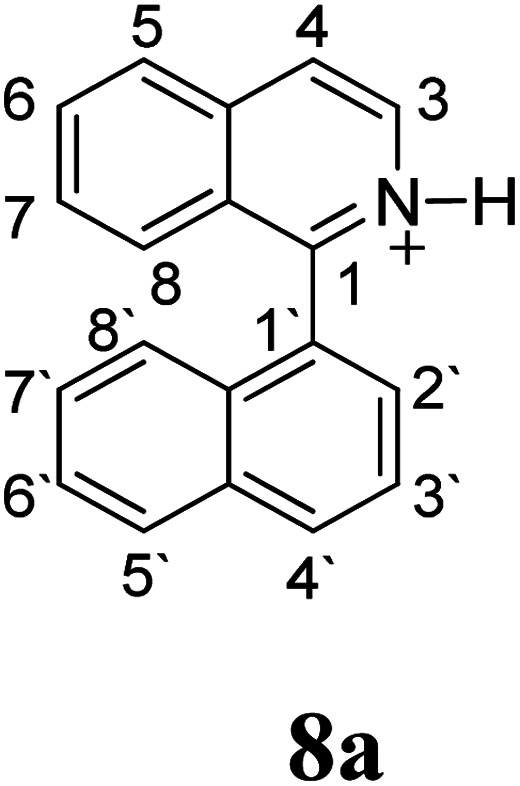	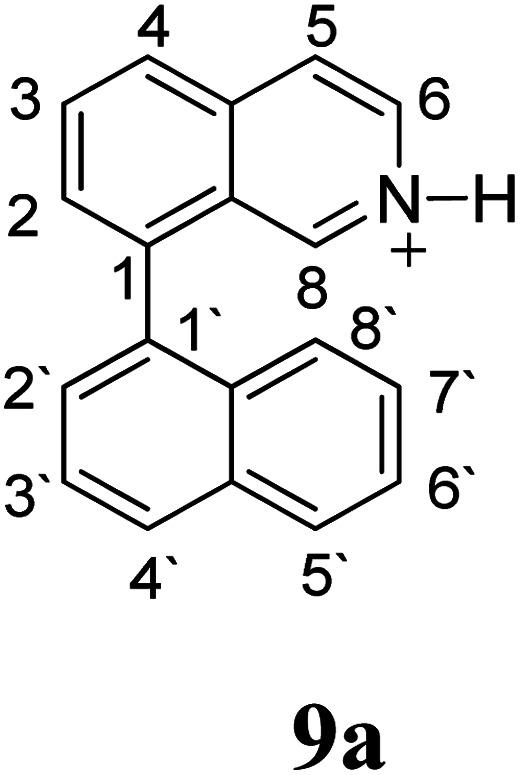
5′ (72%)	1 (98%)	5′ (31%)	5′ (46%)
8′ (18%)	6 (1%)	8′ (66%)	8′ (39%)

The protonation at positions 5′ and 8′ was found to be the energetically favored for 1a, 8a and 9a. When the σ-complex is generated on position 5′, the redistribution of the charge within the ring leads to an intramolecular electrophilic attack, which would generate a new C–C bond between C-8 and C-8′ (Scheme 2). On the other hand, if the σ-complex is generated in position 8′, the cyclization does not take place because a C-sp^3^ is formed at this position. For compound 3 the position 1 is the favored one for the protonation; however, this position does not favor the cyclization reaction.

**Scheme 4:**
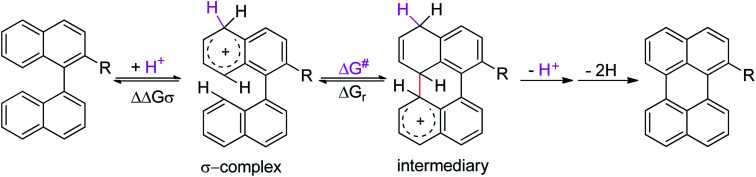
Scholl mechanism proposed for the formation of 2-R-perylene.

To gain a deeper understanding of these reactions, the calculations of two different stages of the proposed Scholl mechanism (Scheme 4) were carried out. First, the free energy values obtained for the formation of the σ-complex (ΔΔ*G*_σ_) were computed, using the reaction of 8a as reference.^38^ Secondly, the activation and reaction free energies (Δ*G*^#^ and Δ*G*_r_ respectively) involved in the intramolecular cyclization were evaluated; the results are presented in Table 3. The last stage of the total reaction is an oxidation with re-aromatization of the formed cycle, which should provide an important driving force.

**Table 3:** Relative free energy of formation of σ complex (ΔΔ*G*_σ_), free energies of activation (Δ*G*^#^) and reaction (Δ*G*_r_) for the intramolecular ring closure by Scholl reaction mechanism^a^

	σ-Complex	ΔΔ*G*_r_ (kcal mol^−1^)	Δ*G*_r_ (kcal mol^−1^)	Δ*G*^#^ (kcal mol^−1^)	Intermediary
1σ	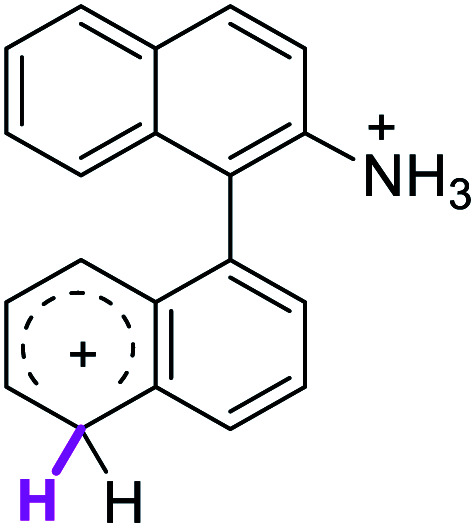	2.0	19.1	24.5	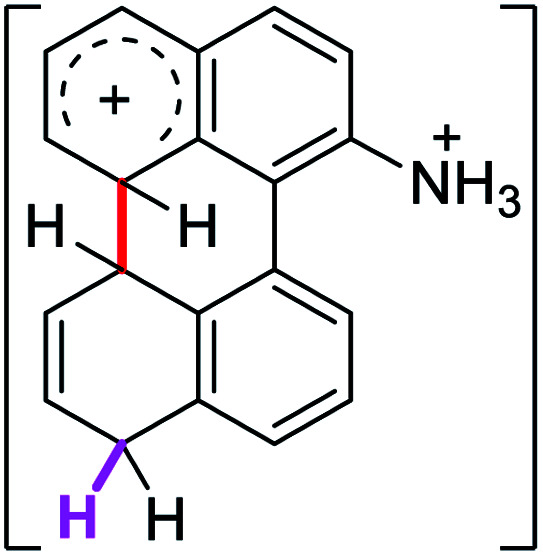
2σ	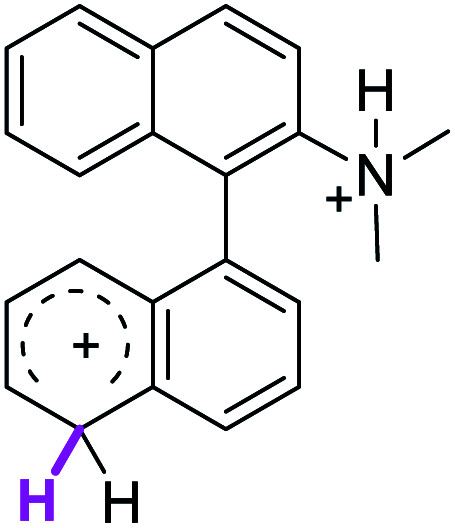	6.7	18.3	23.1	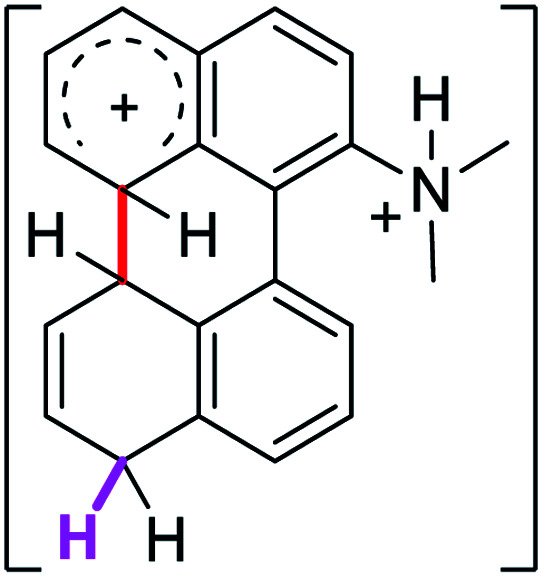
8σ	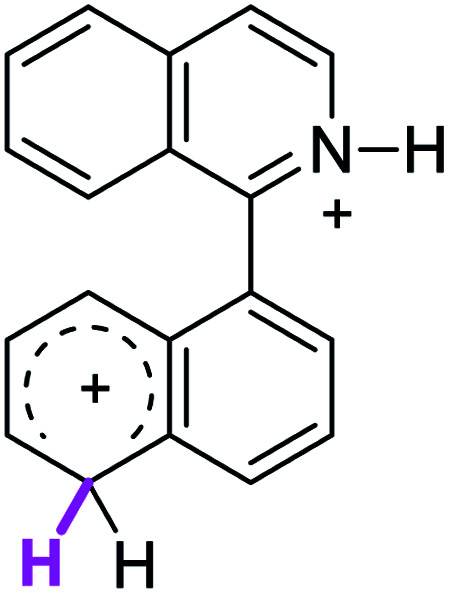	0.0	23.2	26.1	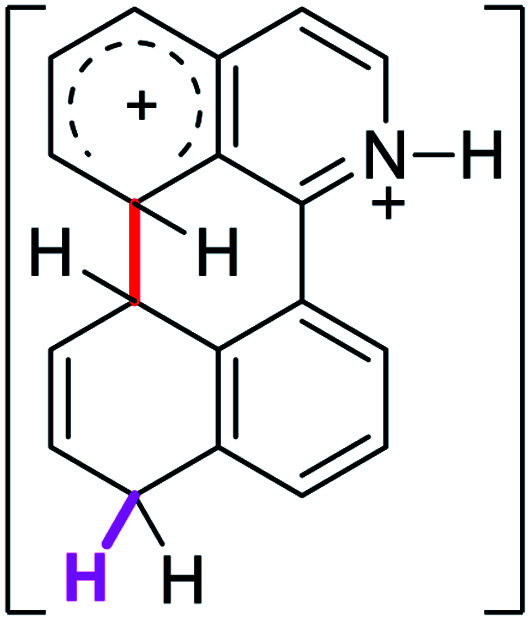
9σ	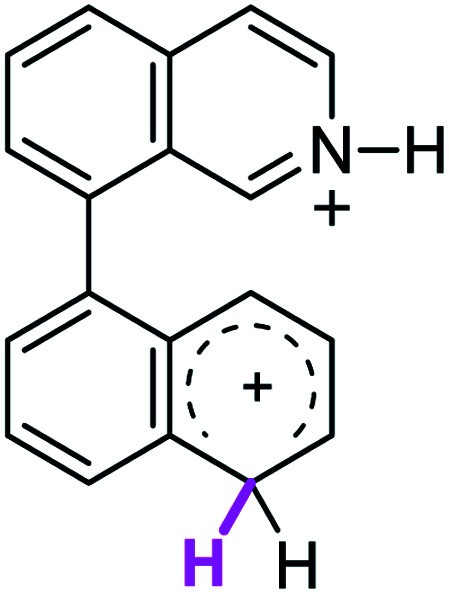	3.0	39.9	40.3	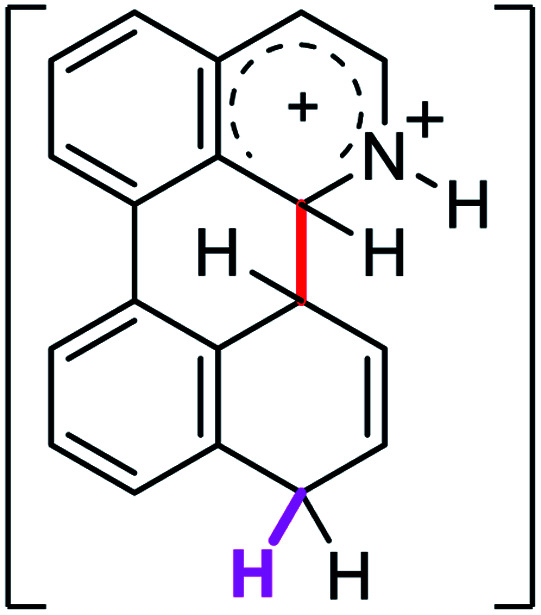

a All the energies were calculated at B3PW91/6-31+G* using *N*-methylformamide as solvent, IEFPCM model. The energies correspond to the sum of electronic energy, solvation, and thermal free energies.

The ΔΔ*G*_σ_ values for the formation of complexes 1σ and 2σ to promote the Scholl reaction compared to that of 8σ, that is used as a reference of a compound that effectively reacts by the Scholl mechanism, indicate that it might be a possible step, even though it would entail a higher energy cost (2.0 kcal mol^−1^ for 1σ and 6.7 kcal mol^−1^ for 2σ). According to the energy of formation of the 9σ complex (∼3.0 kcal mol^−1^ higher than that of 8σ), compound 9 could also lead to the cyclization product. However, this product was not found when the reactions were carried out, hence the discrimination could take place after that step.

**Fig. 1:**
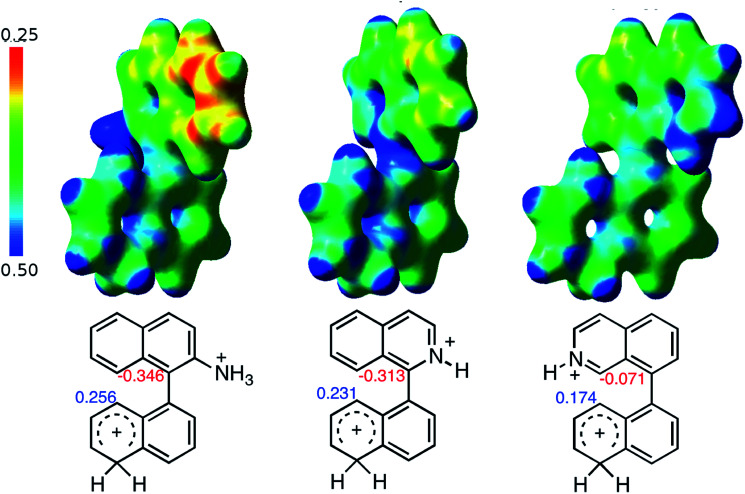
Representation of the electrostatic potential of the σ-complex of 1σ, 8σ and 9σ. In the small figure are represented the point charges that fit the electrostatic potential, according to the Merz–Singh–Kollman^40^ scheme, for C-5 and C-8′.

When the Δ*G*^#^ energies for the intramolecular coupling were studied, it was found that the Δ*G*^#^ for arenium cations 1σ and 2σ were lower than 8σ. Particularly, the 9σ complex presented values of Δ*G*^#^ and Δ*G*_r_ for ring closure higher than the other compounds. The difference exceeds 15 kcal mol^−1^, which would indicate that, in this case, the C–C coupling stage does discriminate between substrates that cyclize and those that do not generate intramolecular cyclization by this mechanism. This difference in the Δ*G*^#^ and Δ*G*_r_, compared with the values for 8σ, are ascribed to the differences in the electronic distributions of 1σ, 8σ and 9σ, as presented in Fig. 1. For 1σ and 8σ the charges at position 5 and 8′ are negative and positive respectively, whereas in the case of 9σ the negative charge is closer to zero due to the protonated nitrogen adjacent to the 8′ position. The electrostatic potential help to explain how the charge is distributed after the formation of the σ complex, and which positions are nucleophilic and/or electrophilic, favoring (or not) the cyclization by an electrophilic attack.

As it was mentioned before, the cyclized product 6 derived from compound 3 by Scholl reaction was not observed (entry 5, Table 1). According to the analysis of the electrostatic potential of 3σ (with H^+^ in position 1), C-5′ and C-8 do not possess the expected charges that favor the coupling (see ESI†). In addition, the free energy for the intramolecular ring closure of 3σ is higher than the one found for compound 9σ which was used as reference of a nonreactive compound.^39^

### Oxidative aromatic coupling

**Fig. 2:**
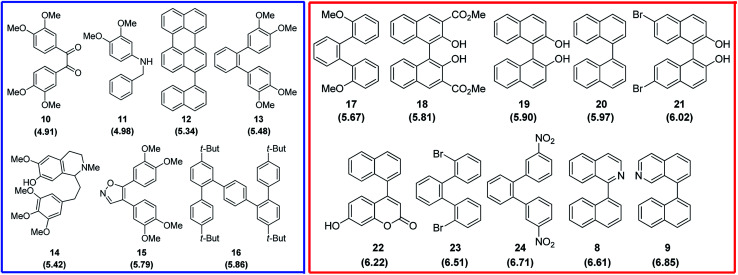
Structure of the reference compounds for oxidative aromatic coupling mechanism. Blue square: Group 1 (reactive); red square: Group 2 (non reactive). Calculated oxidation potential (eV) in parenthesis.^42^

Finally, the reactivity of substrates 1–3 within oxidative aromatic coupling mechanism was also examined with computational studies. The feasibility of initiating the reaction through this mechanistic pathway (Scheme 2) was first evaluated by comparing the computed oxidation potentials.^41^ They were calculated for both, substrates and a group of reference molecules, the results are represented in Fig. 2. The set of reference compounds was split into two groups (Fig. 2): Group 1 (blue box) includes those compounds reported as useful for generating intramolecular cyclization mediated by an intermediary radical cation (using different oxidant agents, i. e. FeCl_3_, DDQ/H^+^, MoCl_5_, *etc.*); Group 2 (red box) includes those compounds that do not produce cyclization under oxidative aromatic coupling experimental conditions.^42^

**Fig. 3:**
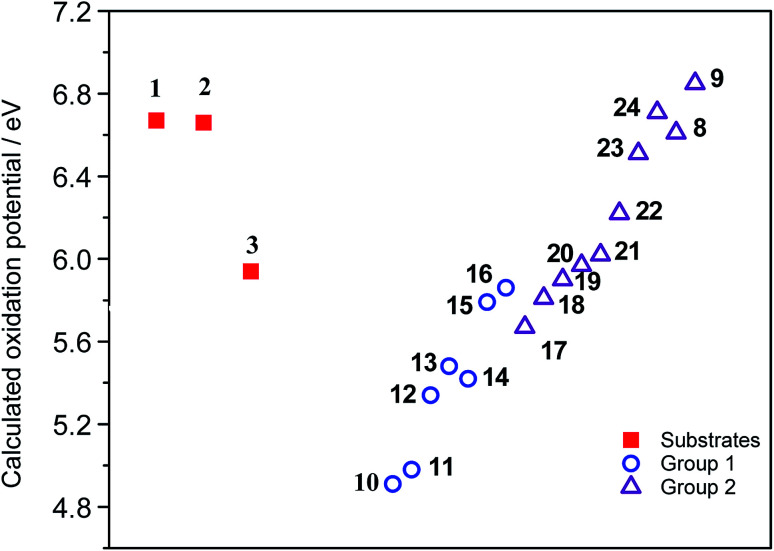
Calculated oxidation potential of the studied compounds.

As expected, the results suggest that compounds from Group 1 have lower oxidation potentials. Meanwhile, the compounds from Group 2 have higher oxidation potentials (Fig. 3).

The oxidation potential values found for substrates 1–3 are in the region of the compounds that would hardly generate the necessary radical cation which initiates the reaction *via* the oxidative aromatic coupling mechanism. This could explain the lack of reactivity of these substrates experimentally observed. These results are in concordance with the previously proposed oxidation potential limit by Rathore *et al.* when DDQ/H^+^ is used as oxidant agent for cyclization of electron-rich molecules.^18,23^ It should be noted that this estimation of the oxidation potential, could help to predict the reactivity of new compounds toward these oxidative aromatic coupling reactions.

### Cyclization of 1,1′:4′,1′′-ternaphthalene

**Scheme 5:**
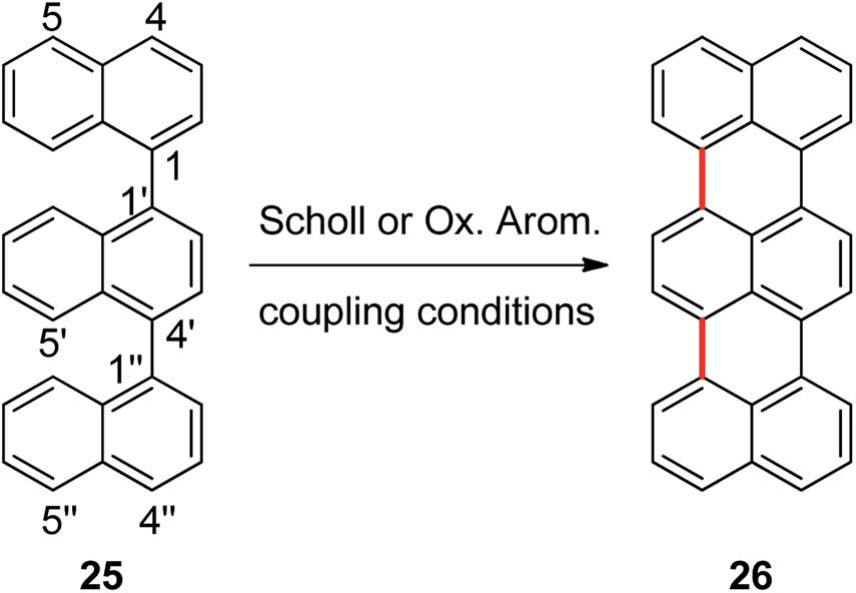
Synthesis of terrylene (26) from 1,1′:4′,1′′-ternaphthalene (25) by Scholl or oxidative aromatic coupling experimental conditions.

In order to explore the scope of these reactions, we propose the synthesis of terrylene from 1,1′:4′,1′′-ternaphthalene (25), Scheme 5. In this reaction there is also another challenge, two new C–C bonds have to be formed to yield the expected product. Reaction of 1,1′:4′,1′′-ternaphthalene with a mixture of AlCl_3_/NaCl (5 : 1) was carried out during 20 min at 170 °C, the corresponding double cycled product terrylene (26) was obtained in 38% isolated yield. It is important to stand out that two new C–C bonds were generated within the same procedure. Perylene 7 (23%) was also obtained as a side product and substrate 25 (20%) was recovered.

The molecular modeling studies of compound 25 indicated that the positions 4 (64%) and 5 (11%) are the ones that protonate mostly, but the Δ*G*^#^ and Δ*G*_r_ for the coupling are markedly favored for the σ-complex generated in position 5 (see ESI†). The electrostatic potential of both intermediary σ-complexes are really different and help to explain this energetic difference observed for the coupling at these positions. Finally, starting form the σ-complex protonated at position 5, the energies involved in two consecutive C–C couplings of 25σ, are slightly lower than the energy values found for the other binaphthyl derivatives previously studied (1, 2, and 8).

As it was found for substrates 1–3, when substrate 25 was also exposed to oxidative aromatic coupling conditions using DDQ as the oxidant agent, the formation of the product 26 was not detected. Again, 25 was also treated with a solution of FeCl_3_ in CH_3_NO_2_. The reaction led to the formation of an inseparable mixture of products that could not be characterized. Moreover, none of them showed the typical fluorescence or UV-absorption profile of the terrylene.^16*a*^ The computed oxidation potential found for 25 was 6.15 eV. Considering Fig. 2, the compound 25 is classified within the group of compounds that do not react by the methodology of oxidative aromatic coupling.

In brief, the results obtained by computational modeling help to explain the observed reactivity of the tested ternaphthyl compound, under both reaction conditions. Moreover, it was possible to obtain terrylene, going through two consecutive C–C coupling reactions, in a simple experimental step with Scholl conditions.

## Conclusions

To summarize, three compounds derived from perylene, two of them functionalized in the equatorial region, were synthesized by a cyclodehydrogenation reaction mediated by Lewis acids, from their respective 2-substituted binaphthalene compounds through a mechanistic pathway that involves the formation of the σ-complex. Regarding the studies performed by computational methods, the regiochemistry for the formation of the σ-complex should be studied. An analysis of the electrostatic potential isosurface of the σ-complexes was very useful to predict the outcome of the cyclization reaction. According to these analyses, within this experimental condition (AlCl_3_/NaCl, 170 °C), substrates 1, 2 and 25 can generate the σ-complex to initiate the reaction and consequently, produce an intramolecular cyclization. For substrate 3, the results suggest that the formation of the initial σ-complex is possible on position 1, but the ring closure from the σ-complex is not energetically favored, consequently the product 6 was not observed.

On the other hand, the formation of the 2-substituted perylene products by cyclodehydrogenation reactions *via* oxidative aromatic coupling (FeCl_3_ or DDQ as oxidants), *e.g.* radical cations as intermediates, was not detected. The studies carried out with DTF methods support that substrates 1, 2, 3, and 25 could not be experimentally oxidized and therefore, could not generate the intermediary radical cation, which is necessary to initiate the reaction and produce the cycled perylene derivative. In summary, we introduce an experimental system where it can be raised the difference between both mechanisms mediated by Lewis acids, and again, molecular modeling is a powerful tool which, on this occasion, helped us to deeply understand the key points to be considered for achieving a successful reaction.

As Gryko proposed,^18^ there is a clear mechanistic difference when any substrate is exposed to Lewis acids to generate a dehydrogenative aromatic coupling product by Scholl reaction or by oxidative aromatic coupling. According to the analysis of both reactions by using molecular modeling methods, it could be possible to explain differences in reactivity and to predict the outcome of a reaction with a new substrate.

## Experimental section

### General methods

Sodium chloride (NaCl), nitromethane (CH_3_NO_2_), 2,3-dichloro-5,6-dicyano-*p*-benzoquinone (DDQ), and trifluoromethanesulfonic acid (TFMSA) were obtained from Sigma-Aldrich and used as received. Iron trichloride (FeCl_3_) was obtained from Merck Millipore and used as received. Aluminum trichloride (AlCl_3_) was obtained commercially and sublimated before used. Dichloromethane was obtained commercially, distilled, and stored under molecular sieves (4 Å). Gas chromatographic analysis was performed on a Varian GC with a flame ionization detector and equipped with a VF-5 ms, 30 m × 0.25 mm × 0.25 μm column. Gas chromatographic/mass spectrometer analysis was carried out on a Shimadzu GC-MS QP 5050 spectrometer equipped with a VF-5 ms, 30 m × 0.25 mm × 0.25 μm column. HRMS were recorded on a Bruker, MicroTOF Q II equipment, operated with an ESI source in (positive/negative) mode, using nitrogen as nebulizing and drying gas and sodium formate 10 mM as an internal standard. HPLC analysis was carried out on a Waters 1525 Binary HPLC Pump connected to a Waters 2998 Photodiode Array Detector, and employing an Agilent Rx-Sil Analytical column (4.6 × 150 mm, 5 μm). Elemental analysis was determined on a Perkin Elmer 2400 Series II CHNS/O with a thermal conductivity detector (TCD). ^1^H NMR and ^13^C NMR were recorded on a 400 MHz Bruker nuclear magnetic resonance spectrometer.

The substrates 1,^28*a*,28*c*^3,^43^ and 25 (ref. 44) were synthesized and purified by previously published methods, their NMR spectra are shown in ESI.† The new compound *N*,*N*-dimethyl-[1,1′-binaphthyl]-2-amine (2) was obtained by the methylation of amine 1 (see ESI†).

#### General procedure for the cyclodehydrogenation by Scholl reaction

A mixture of dry sodium chloride (30 mg, 1 eq.) and aluminum chloride (333 mg, 5 eq.) was heated and stirred at 170 °C under nitrogen atmosphere. Once this mixture is melted, the substrate (0.2 eq., 0.1 mmol) was added. In the reactions carried out with substrates 1 and 2, after the reaction finished (reaction times are indicated in Table 1), the mixture was cooled to room temperature and extracted with CH_2_Cl_2_ and H_2_O (3 × 25 mL). The organic layer was dried under Na_2_SO_4_, filtered, and evaporated under reduced pressure. The crudes were analyzed by GC and GC-MS. In the case of the reaction of substrate 25, after the end of the reaction time (20 min), the mixture was cooled at room temperature and quenched with diluted (10%) HCl (5 mL). The resulting precipitate was filtered under suction and rinsed with H_2_O.

When milder conditions were employed in the Scholl reaction, 2-methoxy-1,1′-binaphthalene (3) (0.2 mmol, 1 eq.) was dissolved in dry dichloromethane (10 mL). After stirring for 20 min under N_2_ atmosphere, anhydrous aluminum chloride (1.8 mmol, 9 eq.) was added. The solution was stirred at 80 °C for 6 h. For the extraction process, CH_2_Cl_2_ and H_2_O (3 × 20 mL) were used.

#### General procedure for the oxidative cyclodehydrogenation with DDQ and methanesulfonic acid

A solution of the substrate (0.4 mmol, 1 eq.) in dry dichloromethane (4 mL) containing methanesulfonic acid (10% v/v, 0.8 mL) at ∼0 °C was exposed to DDQ (0.4 mmol, 1 eq. per C–C bond formation). The reaction was stirred under a nitrogen atmosphere from 30 min to 5 h, at room temperature. The progress of the reaction was monitored by TLC. The reaction was quenched with a saturated aqueous solution of NaHCO_3_ (20 mL). The dichloromethane layer was separated, washed with water and brine solution, dried over anhydrous NaSO_4_, and filtered.

#### General procedure for the oxidative cyclodehydrogenation using FeCl_3_

A solution of anhydrous iron(iii) chloride (3.2 mmol) in dry nitromethane (3.2 mL) was injected to a solution of the substrate (0.4 mmol) in dry dichloromethane (30 mL) through a syringe. The solution was stirred at room temperature for 24 h under nitrogen atmosphere. Dry methanol (30 mL) was added to the solution. The resulting precipitate was filtered, rinsed with methanol, and dried.

### *N*,*N*-Dimethyl-[1,1′-binaphthalen]-2-amine (2)

The compound was purified as a pale yellow solid by column chromatography using as eluent a solvent gradient of pentane/ethyl acetate (100 : 0 → 90 : 10). Mp: 193–195 °C. Yield: 47% (0.17 mmol, 52 mg). ^1^H NMR (400 MHz, CDCl_3_) *δ*: 7.92 (t, ^1^*J* = 8.0 Hz, 3H); 7.82 (d, ^1^*J* = 8.0 Hz, 1H); 7.61 (m, 1H); 7.50 (d, ^1^*J* = 8.0 Hz, 1H); 7.48–7.44 (m, 2H); 7.37 (d, ^1^*J* = 8.0 Hz, 1H); 7.31–7.25 (m, 2H); 7.18–7.14 (m, 1H); 7.09 (d, ^1^*J* = 8.5 Hz, 1H); 2.51 (s, 6H). ^13^C NMR (100 MHz, CDCl_3_) *δ* = 149.9 (C_q,_ C_Ar_–N); 137.2 (C_q_); 134.4 (C_q_); 133.9 (C_q_); 133.2 (C_q_); 129.7 (C_q_); 129.1 (C_Ar_–H); 128.9 (C_Ar_–H); 128.3 (C_Ar_–H); 127.8 (C_q_), 127.8 (C_Ar_–H); 127.6 (C_Ar_–H); 126.7 (C_Ar_–H); 126.1 (C_Ar_–H); 125.9 (C_Ar_–H); 125.8 (C_Ar_–H); 125.8 (2 × C_Ar_–H); 123.7 (C_Ar_–H); 119.8 (C_Ar_–H); 44.0 (2 × CH_3_). HRMS (ESI-TOF) *m*/*z* [M + Na]^+^ calcd for C_22_H_19_NNa: 320.1410; found: 320.1424.

### Perylene-1-amine (4)

The product was purified as a yellow oily solid by semipreparative TLC using as eluent pentane/ethyl acetate (90 : 10). In this specific case, a second consecutive semipreparative TLC was made. The product decomposes when it is exposed to air. Isolated yield: 14% (4 mg). ^1^H, COSY, HMBC and HSQC-DEPT NMR spectra are showed in ESI.†^1^H NMR (400 MHz, (CD_3_)_2_CO) *δ* = 7.97 (d, ^1^*J* = 7.6 Hz, 1H); 7.73–7.69 (m, 2H); 7.23 (d, ^1^*J* = 7.7, 1H); 7.17–7.10 (m, 3H); 7.06–6.99 (m, 2H); 6.81 (t, ^1^*J* = 7.6 Hz, 1H); 6.72 (d, ^1^*J* = 8.8 Hz, 1H); 5.58 (s, 2H, NH_2_). ^13^C NMR (100 MHz, (CD_3_)_2_CO) *δ* = 145.6 (C_q_, C–N); 134.6 (C_q_); 132.3 (C_q_); 131.6 (C_q_); 130.5 (C_q_); 129.7 (C_q_); 129.1 (C_q_); 128.9 (C_Ar_–H); 128.2 (C_Ar_–H); 127.9 (C_q_); 126.9 (C_Ar_–H); 126.7 (2C, C_Ar_–H); 124.8 (C_Ar_–H); 122.9 (C_Ar_–H); 122.2 (C_Ar_–H); 121.7 (C_Ar_–H); 121.5 (C_Ar_–H); 118.9 (C_Ar_–H); 109.7 (C_q_). ^1^H–^1^H COSY NMR ((CD_3_)_2_CO): *δ*_H_/*δ*_H_ 7.97/7.01; 7.71/6.81; 7.69/7.04; 7.23/7.04; 7.16/6.81; 7.11/6.72; 7.13/7.01. ^1^H–^13^C HSQC NMR ((CD_3_)_2_CO): *δ*_H_/*δ*_C_ 7.97/121.5; 7.71/121.7; 7.69/118.9; 7.23/126.7; 7.16/128.2; 7.13/124.8; 7.11/128.9; 7.04/126.7; 7.01/126.9; 6.81/122.9; 6.72/122.2. ^1^H–^13^C HMBC NMR ((CD_3_)_2_CO): *δ*_H_/*δ*_C_ 7.97/109.7; 7.97/124.8; 7.97/129.7; 7.71/128.2; 7.71/130.5; 7.71/131.6; 7.69/126.7; 7.69/129.1; 7.69/129.7; 7.23/118.9; 7.23/124.8; 7.23/129.7; 7.16/121.7; 7.16/128.9; 7.16/130.5; 7.13/121.5; 7.13/126.7; 7.13/129.7; 7.11/128.2; 7.11/130.5; 7.11/145.6; 7.04/131.6; 7.04/134.6; 7.01/132.3; 7.01/134.6; 6.81/127.9; 6.81/129.1; 6.72/109.7; 6.72/127.9; 5.58/109.7; 5.58/122.2. MS (EI): *m*/*z* 267 (100.0%), 133 (36.0%), 118 (19%).

### *N*,*N*-Dimethylperylene-1-amine (5)

The compound was purified as an orange oily solid by semipreparative TLC using as eluent a solvent gradient of pentane/ethyl acetate (100 : 0 → 90 : 10). Isolated yield: 50% (15 mg). ^1^H, ^13^C, COSY, HMBC and HSQC-DEPT NMR spectra are showed in ESI.†^1^H NMR (400 MHz, (CD_3_)_2_CO) *δ*: 9.21 (dd, ^1^*J* = 7.8, ^2^*J* = 1.0 Hz, 1H); 8.21 (dd, ^1^*J* = 7.5, ^2^*J* = 1.1 Hz, 2H); 7.73–7.64 (m, 4H); 7.54–7.46 (m, 3H); 7.38 (t, ^1^*J* = 7.8 Hz, 1H); 2.85 (s, 6H). ^13^C NMR (100 MHz, (CD_3_)_2_CO) *δ*: 151.0 (C_q_, C–N); 135.6 (C_q_); 132.5 (C_q_); 132.4 (C_q_); 131.9 (C_q_); 131.6 (C_q_); 131.6 (C_q_); 130.8 (C_q_); 129.5 (C_Ar_–H); 128.5 (C_Ar_–H); 128.2 (C_Ar_–H); 127.5 (C_Ar_–H); 127.1 (C_Ar_–H); 127.0 (C_Ar_–H); 125.2 (C_Ar_–H); 124.5 (C_Ar_–H); 122.1 (C_Ar_–H); 121.5 (C_Ar_–H); 120.1 (C_Ar_–H); 119.6 (C_q_); 43.4 (2C, C̲H_3_). HRMS (ESI-TOF) *m*/*z* [M + Na]^+^ calcd for C_22_H_17_NNa: 318.1253; found: 318.1261.

### Perylene (7)

The product was purified as a pale yellow solid by semipreparative TLC using as eluent pentane/ethyl acetate (90 : 10). ^1^H and ^13^C NMR spectra are showed in ESI.†^1^H NMR (400 MHz, CDCl_3_) *δ*: 8.19 (d, ^1^*J* = 7.8 Hz, 4H); 7.68 (d, ^1^*J* = 8.0 Hz, 4H); 7.48 (t, ^1^*J* = 7.7 Hz, 4H). ^13^C NMR (100 MHz, CDCl_3_) *δ*: 134.9 (2 × C_q_); 131.4 (2 × C_q_); 129.0 (4 × C_q_); 128.0 (4 × C_Ar_–H); 126.7 (4 × C_Ar_–H); 120.4 (4 × C_Ar_–H). MS (EI): *m*/*z* 252 (100%), 125 (18%), 113 (7%).

### Terrylene (26)^16*a*^

After reaction the crude was extracted in a Soxhlet apparatus, using solvents in increasing polarity starting from ethyl acetate (100 mL, refluxing for 24 h) and then toluene (100 mL, refluxing for 24 h). The product 26 was isolated as a dark solid. Isolated yield: 38% (29 mg). UV-visible spectrum is showed in ESI.† UV-vis: *λ*_max_ in dichloromethane/nm: 550.4, 508.9, 476.1. Elemental analysis: calc. for C_30_H_16_: C 95.7%, H 4.28%; found: C 95.82%, H 4.18%.

### Computational procedure

All the calculations were performed with the Gaussian09 program.^34^ Based on previous results,^45^ the B3PW91 DFT functional^46^ and the 6-31+G(p) basis set were selected. Calculations were performed with full geometry optimization including in all cases the effect of the solvent through the Tomasi's polarized continuum model (IEFPCM)^36*a*^ as implemented in the Gaussian package. After refinement, the characterization of stationary points was done by Hessian matrix calculations, with all positive eigenvalues for a minimum and only one negative eigenvalue for the TSs. The *xyz* coordinates of all the obtained geometries are included in the ESI,† together with the SCF energy, in the case of TSs, the value of the negative frequency is also included.

## Conflicts of interest

There are no conflicts to declare.

## Supplementary Material

RA-010-D0RA04213G-s001
