# Supplementary material for: Arenium cation or radical cation? An insight into the cyclodehydrogenation reaction of 2-substituted binaphthyls mediated by Lewis acids
Source: RSC Adv. 2020 Jun 9;10(37):21974–85. doi: 10.1039/d0ra04213g (PMC9054548; doi:10.1039/d0ra04213g)
Supplement: RA-010-D0RA04213G-s001 [file RA-010-D0RA04213G-s001.pdf]

Electronic Supplementary Material (ESI) for RSC Advances.
This journal is © The Royal Society of Chemistry 2020

Arenium cation or radical cation? An insight on the cyclodehydrogenation reaction of 2-substituted binaphthyls

Patricia Camargo Solórzano, María T. Baumgartner, Marcelo Puiatti and Liliana B. Jimenez**

INFIQC, Departamento de Química Orgánica, Facultad de Ciencias Químicas, Universidad Nacional de Córdoba, Ciudad Universitaria, X5000HUA, Córdoba, Argentina.

SUPPLEMENTARY INFORMATION

INDEX

1. **Synthesis procedure for *N,N*-dimethyl-[1,1'-binaphthalen]-2-amine (2).** **SI -3-**
2. **Spectroscopic data.**
2.1 ^{1}H-NMR spectrum of **[1,1'-binaphthalen] -2-amine (1)** **SI -4-**
2.2 ^{1}H- and ^{13}C-NMR spectra of ***N,N*-dimethyl-[1,1'-binaphthalen]-2-amine (2)** **SI -5-**
2.3 ^{1}H-NMR spectrum of **2-methoxy-1,1'-binaphthalene (3)** **SI -7-**
2.4 ^{1}H- and 2D NMR spectra of **perylene-1-amine (4).** **SI -8-**
2.5 ^{1}H-, ^{13}C- and 2D NMR spectra of ***N,N*-dimethylperylene-1-amine (5)** **SI -12-**
2.6 ^{1}H- and ^{13}C-NMR spectra of **perylene (7).** **SI -18-**
2.7 ^{1}H-NMR spectrum of **1,1': 4',1"- ternaphthalene (25)** **SI -20-**
2.8 UV-Visible spectra of **terrylene (26).** **SI -21-**
3. **Boltzman population analysis for the formation of the σ-complex for 1a, 8a, 9a, 3, and 25 at 170 °C.** **SI -22-**
4. **Representation of the electrostatic potential of 3σ.** **SI -25-**
5. ΔG_r **and** $\Delta G^{\#}$ **for the ring closure by Scholl reaction of the complexes 1σ, 2σ, 8σ, 9σ and 25σ.** **SI -26-**
6. **Description of the procedure employed for the calculation of Redox Potential.** **SI -27-**
7. **Compounds used as references in the theoretical study of aromatic oxidative coupling mechanism.** **SI -28-**
8. **Formation of Terrylene: study of Scholl reaction**.
8.1 Calculated energies for the first cyclization step from isomers σ_4 and σ_5 of protonated 25. **SI -29-**
8.2 Calculated energies involved in both ring closures of 25σ by the Scholl mechanism, σ-complex generated in position 5. **SI -30-**
9. **Electrostatic potential isosurface of 25σ and the intermediary product of cyclization to afford terrylene.** **SI-31-**
10. **XYZ Coordinates of the computed compounds.** **SI-32-**

1. Synthesis of N,N-dimethyl-[1,1'-binaphthalen]-2-amine (2).

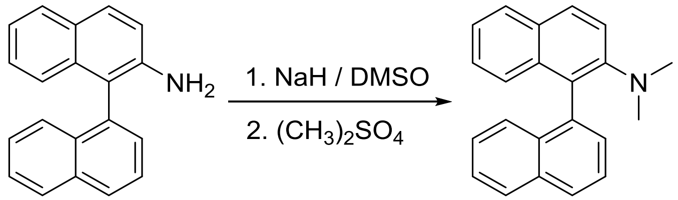

100 mg (1 eq., 0.37 mmol) de [1,1'-binaphthalen]-2-amine (**1**) was dissolved with DMSO (5 mL) in 50 mL round bottom flask at room temperature. After stirring 10 minutes, NaH was added (10 eq., 89 mg) and the reaction mixture was left stirring for 30 minutes. After this, $(CH_3)_2SO_4$ (10 eq, 3.7 mmol, 0.350 mL) was added slowly over a period of 30 minutes, and finally stirring overnight. Worked up by adding carefully water (10 mL). Extracted the aqueous portion 3 times with ethyl acetate. The ethyl acetate layers were combined and rinsed 3 times with water (10 mL). The ethyl acetate layer was then dried over Na_2SO_4 and then, the solvent was evaporated under reduced pressure. The solid was purified over silica gel in 1:1 hexanes/ethyl acetate. The product was obtained as pale yellow solid, 47% yield (0.17 mmol, 52 mg).
Spectroscopic characterization is described in experimental section in main article.

2. *Spectrocopic data*

2.1 - **[1,1'-Binaphthalen]-2-amine (1).** ^{1}**H-NMR (400 MHz, acetone-*d*$_6$)**

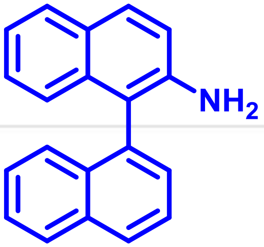

1

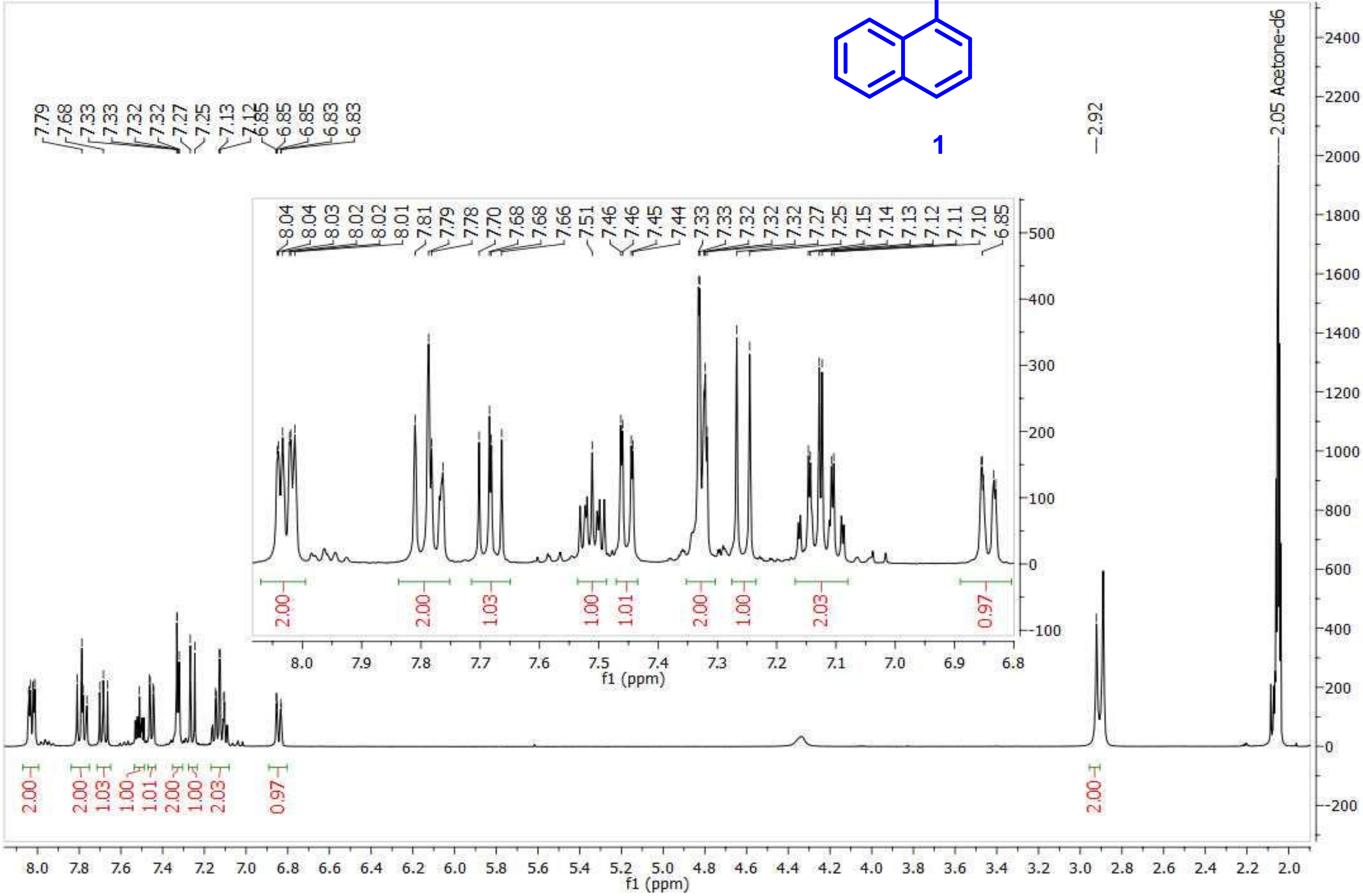

_2.2_ - _N,N_-dimethyl-[1,1'-binaphthalen]-2-amine (2)

^{1}H-NMR (400 MHz, chloroform-d_1)

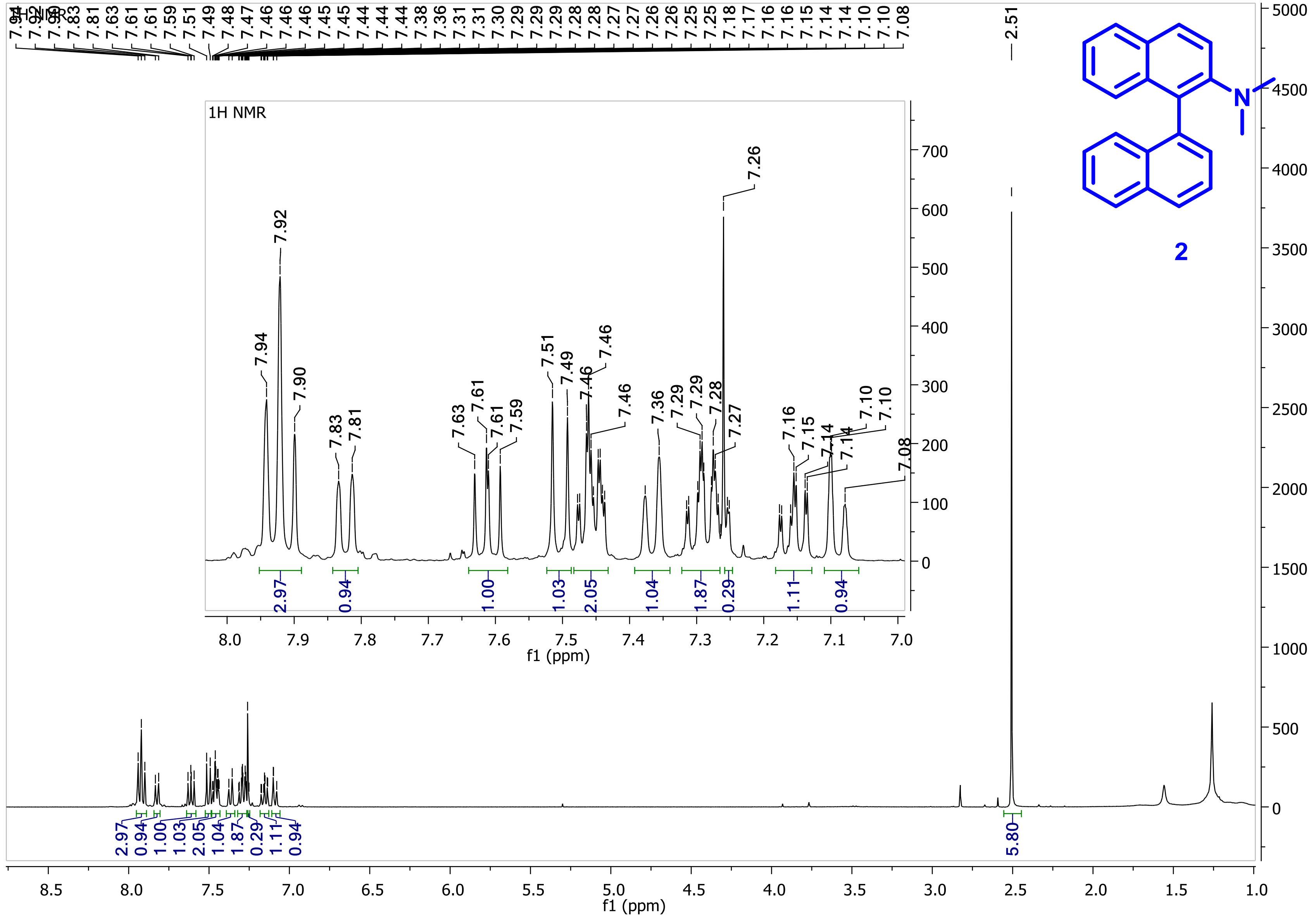

^{13}C-NMR (100 MHz, chloroform-d_1)

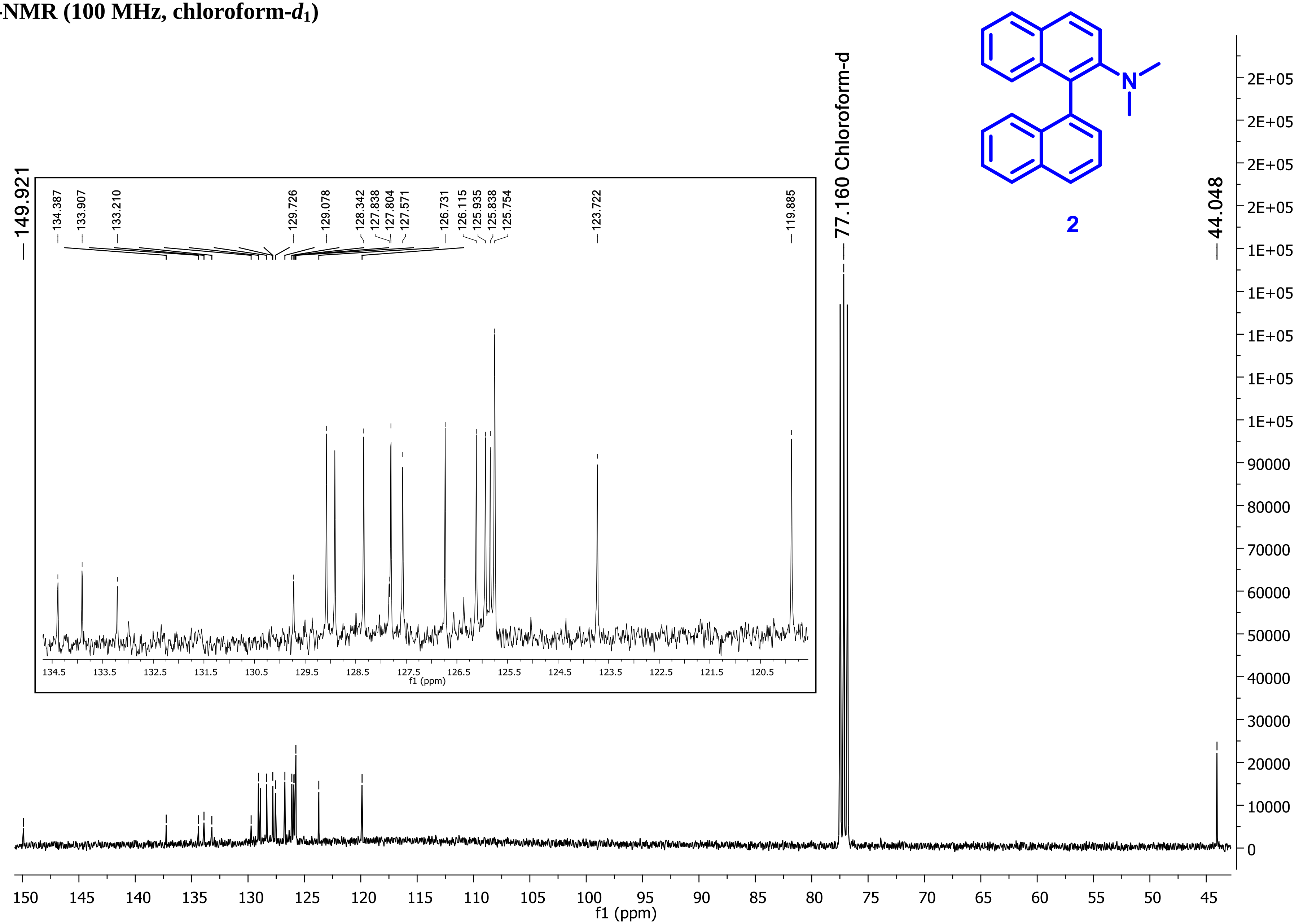

2.3 – **2-Methoxy-1,1'-binaphthalene (3)**
^{1}H-NMR (400 MHz, acetone-d$_6$)

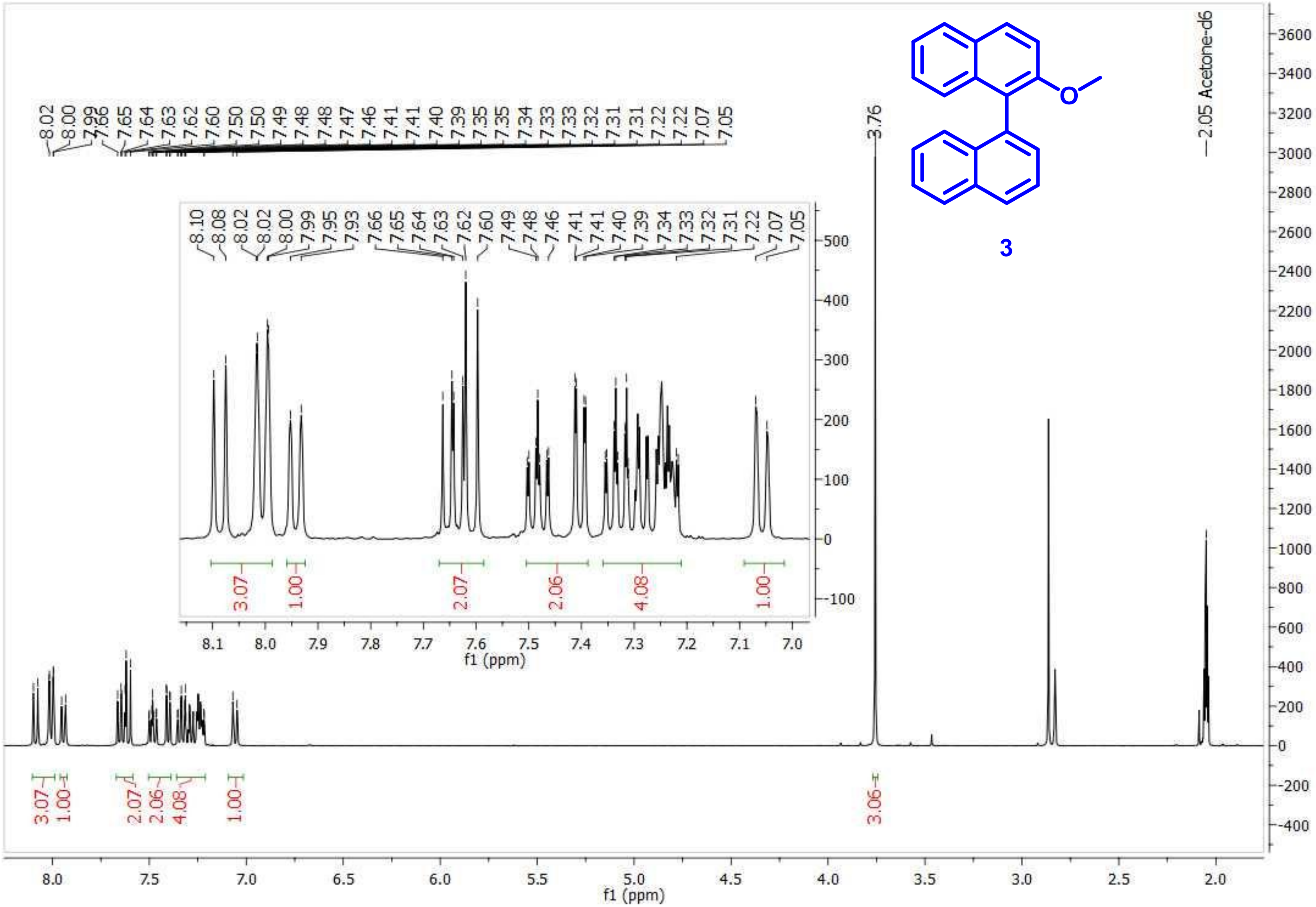

2.4 **– Perylene-1-amine (4)**
^{1}H-NMR (400 MHz, acetone-d_6)

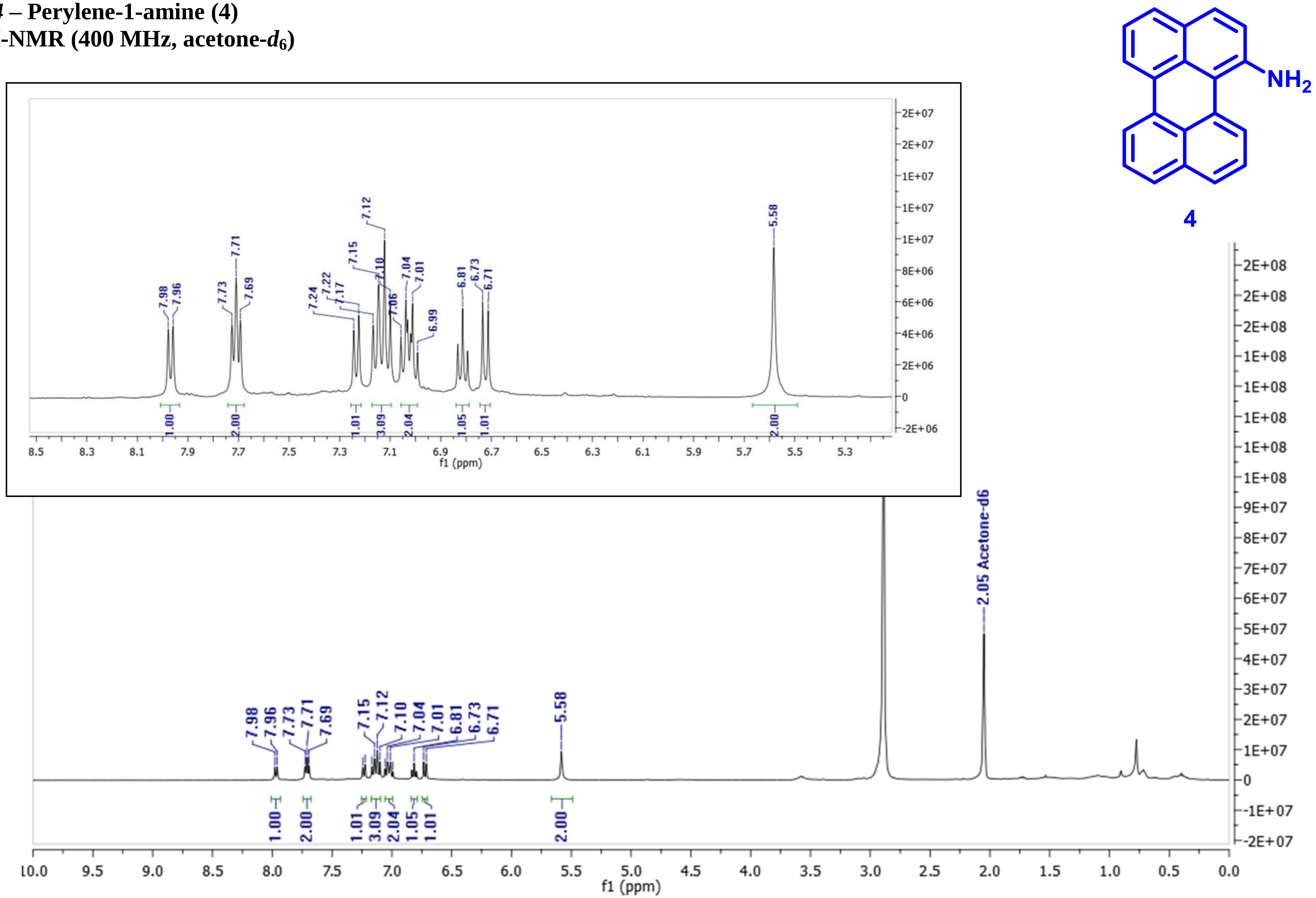

COSY

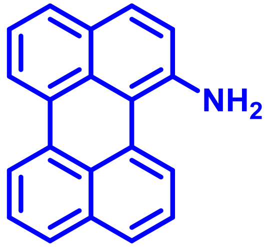
NH2

4

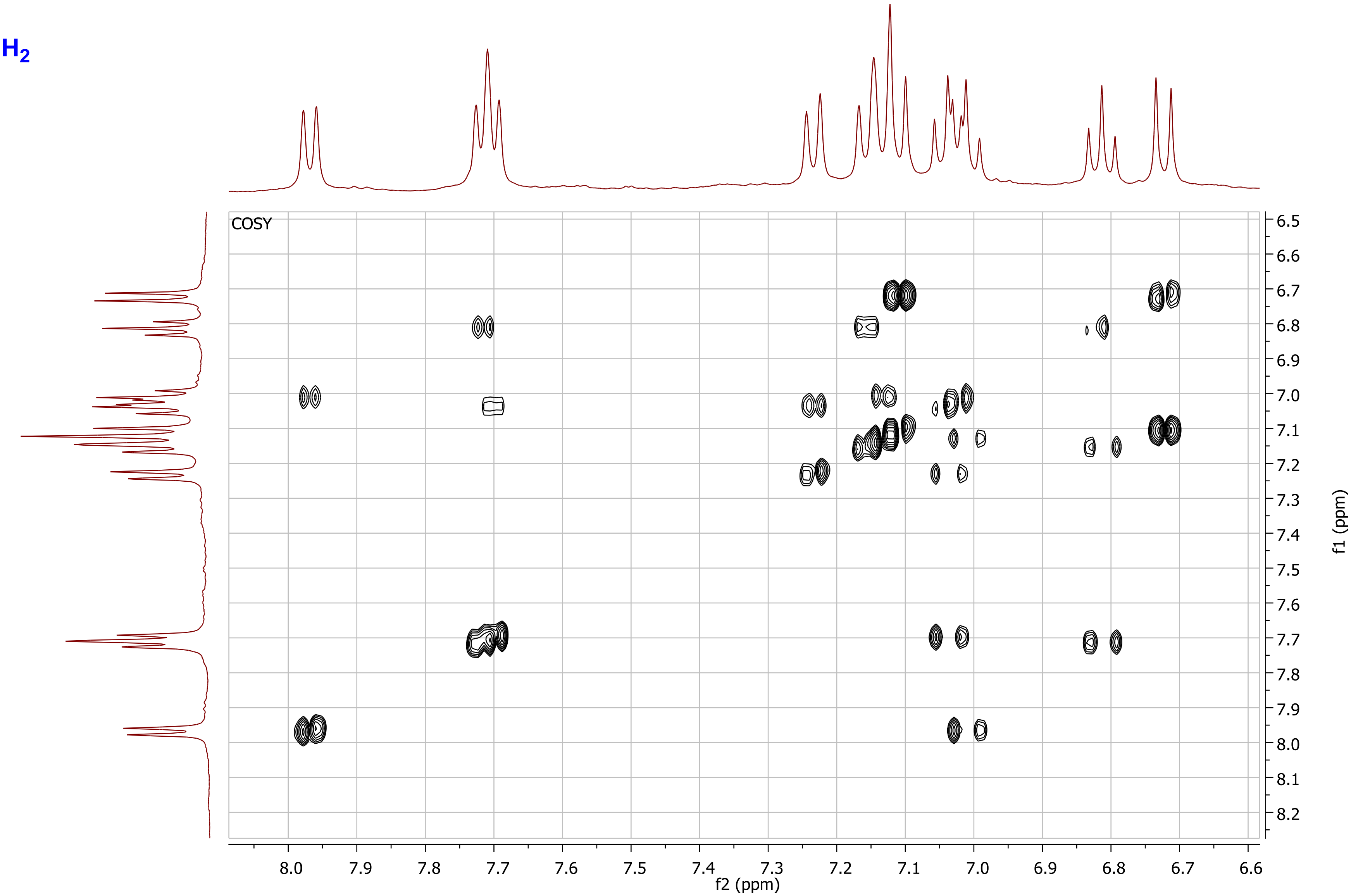
COSY
8.0
7.9
7.8
7.7
7.6
7.5
7.4
7.3
7.2
7.1
7.0
6.9
6.8
6.7
6.6
f2 (ppm)
6.5
6.6
6.7
6.8
6.9
7.0
7.1
7.2
7.3
7.4
7.5
7.6
7.7
7.8
7.9
8.0
8.1
8.2
f1 (ppm)

HSQC

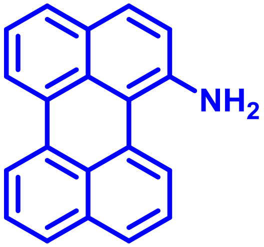
NH2

4

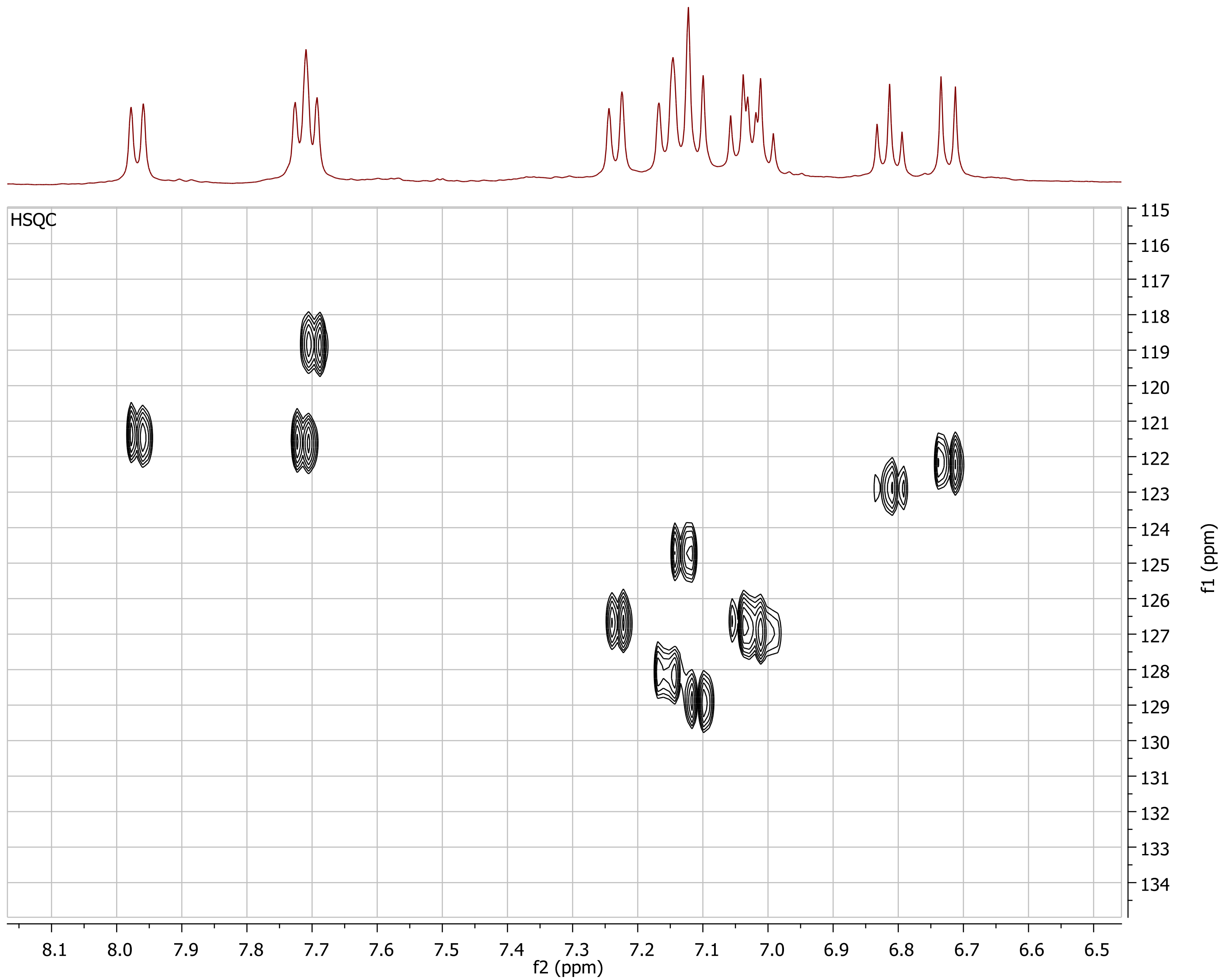
HSQC
8.1
8.0
7.9
7.8
7.7
7.6
7.5
7.4
7.3
7.2
7.1
7.0
6.9
6.8
6.7
6.6
6.5
f2 (ppm)
115
116
117
118
119
120
121
122
123
124
125
126
127
128
129
130
131
132
133
134
f1 (ppm)

HMBC

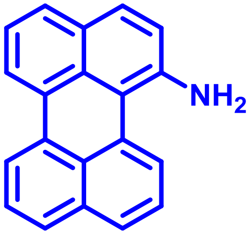
NH2

4

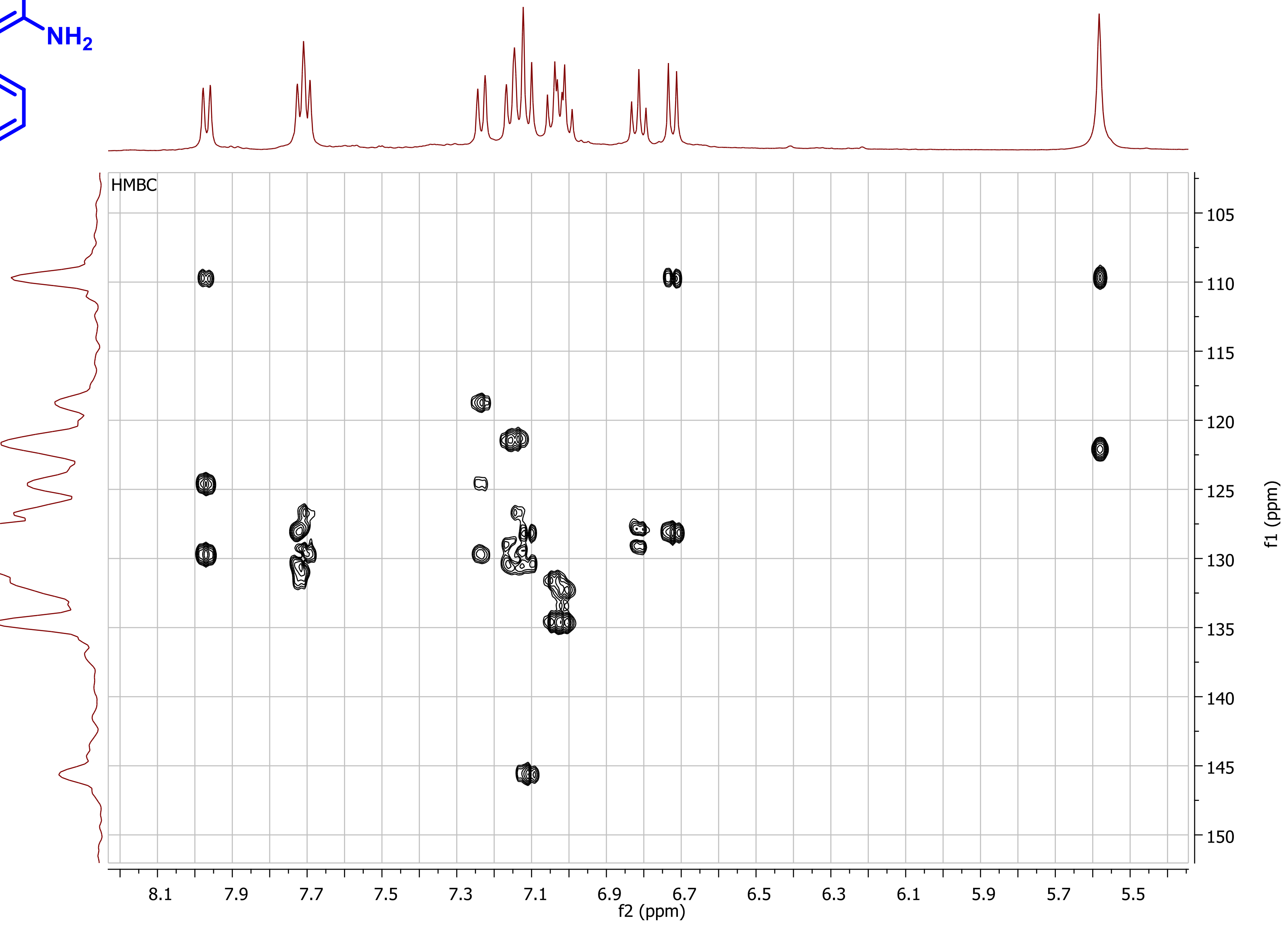
HMBC
105
110
115
120
125
130
135
140
145
150
f1 (ppm)
8.1
7.9
7.7
7.5
7.3
7.1
6.9
6.7
6.5
6.3
6.1
5.9
5.7
5.5
f2 (ppm)

2.5 – ***N,N*-dimethylperylene-1-amine (5)**

^{1}H-NMR (400 MHz, acetone - d_6)

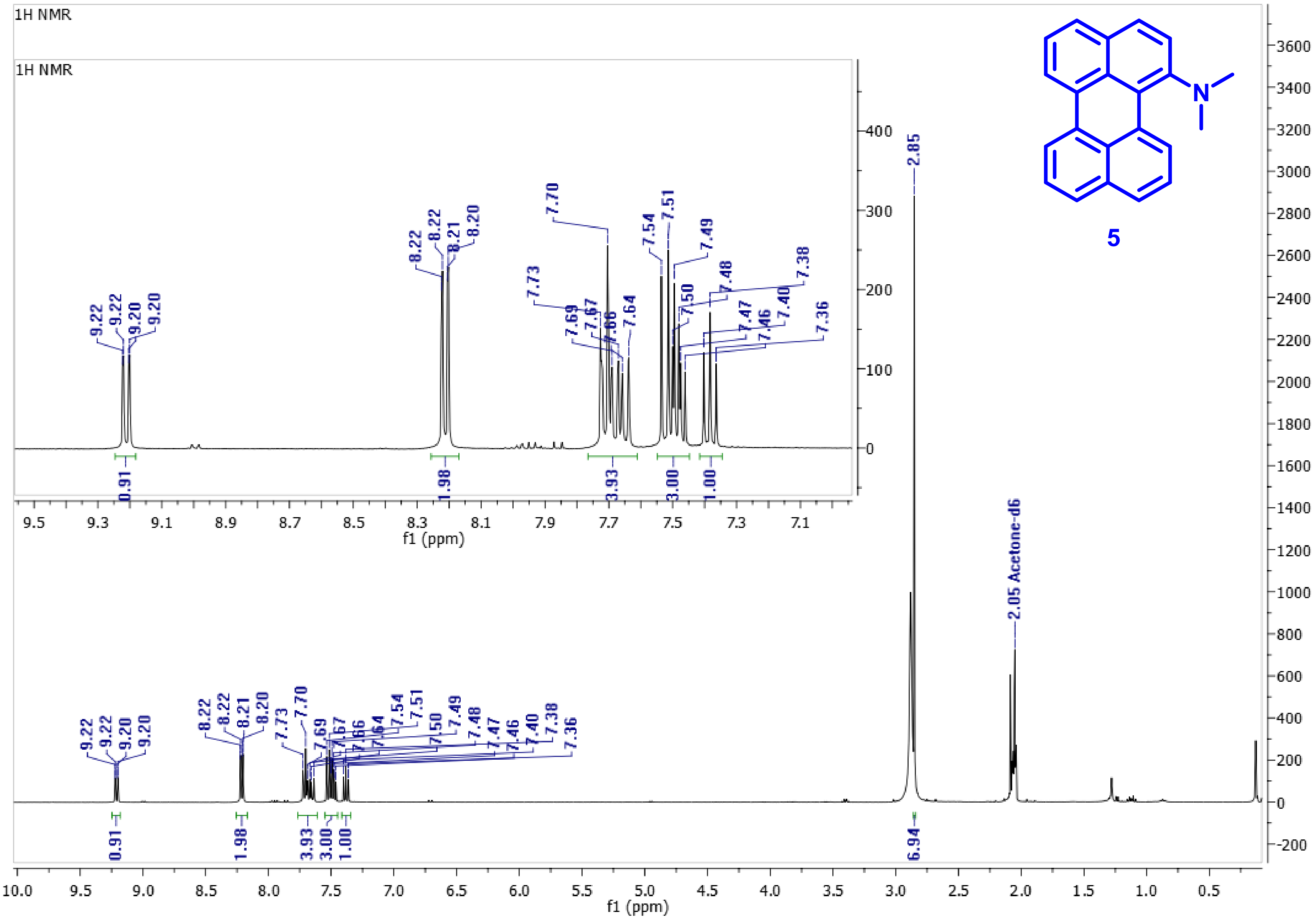

^{13}C-NMR (100 MHz, acetone-d_6)

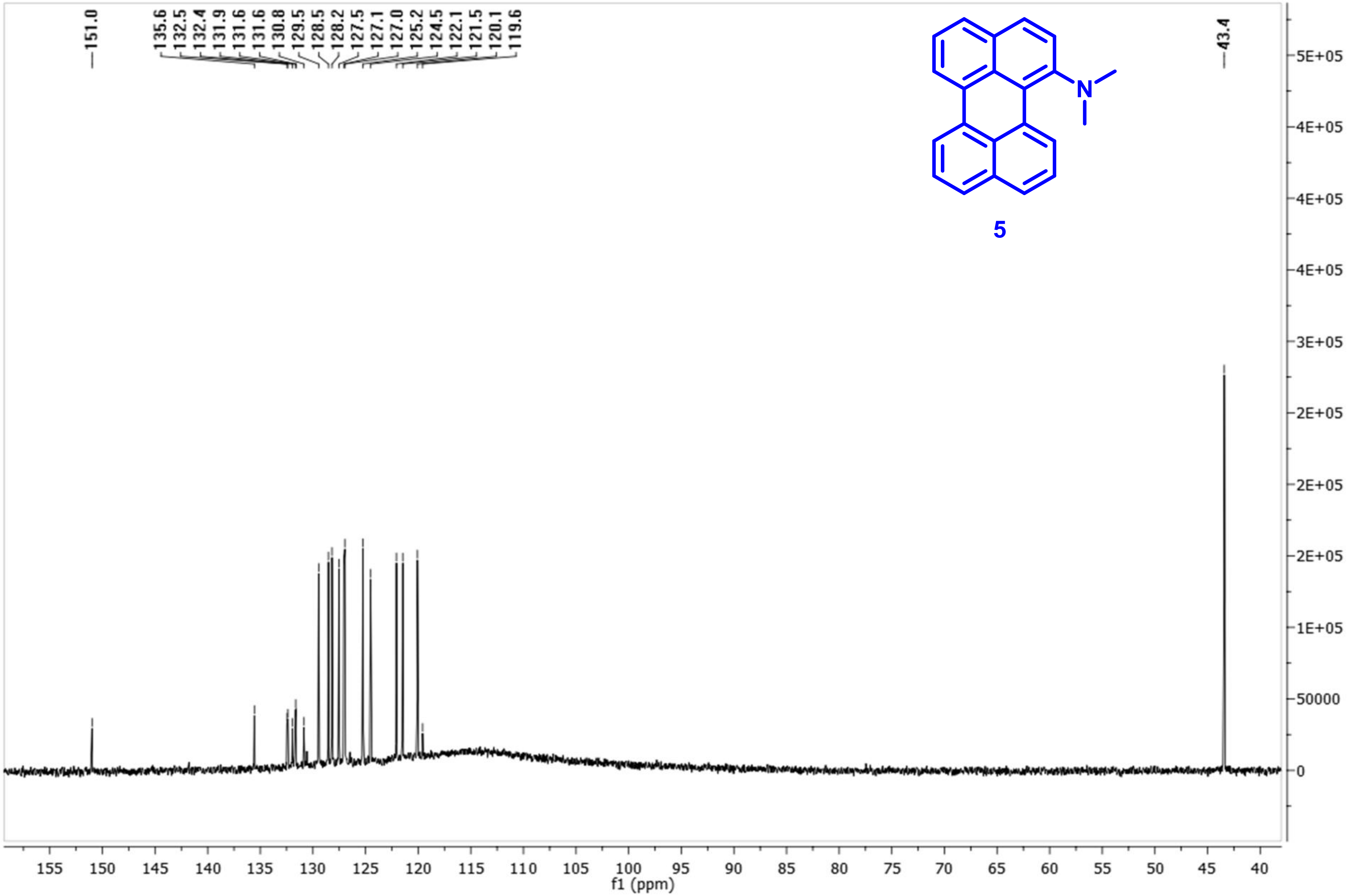

COSY

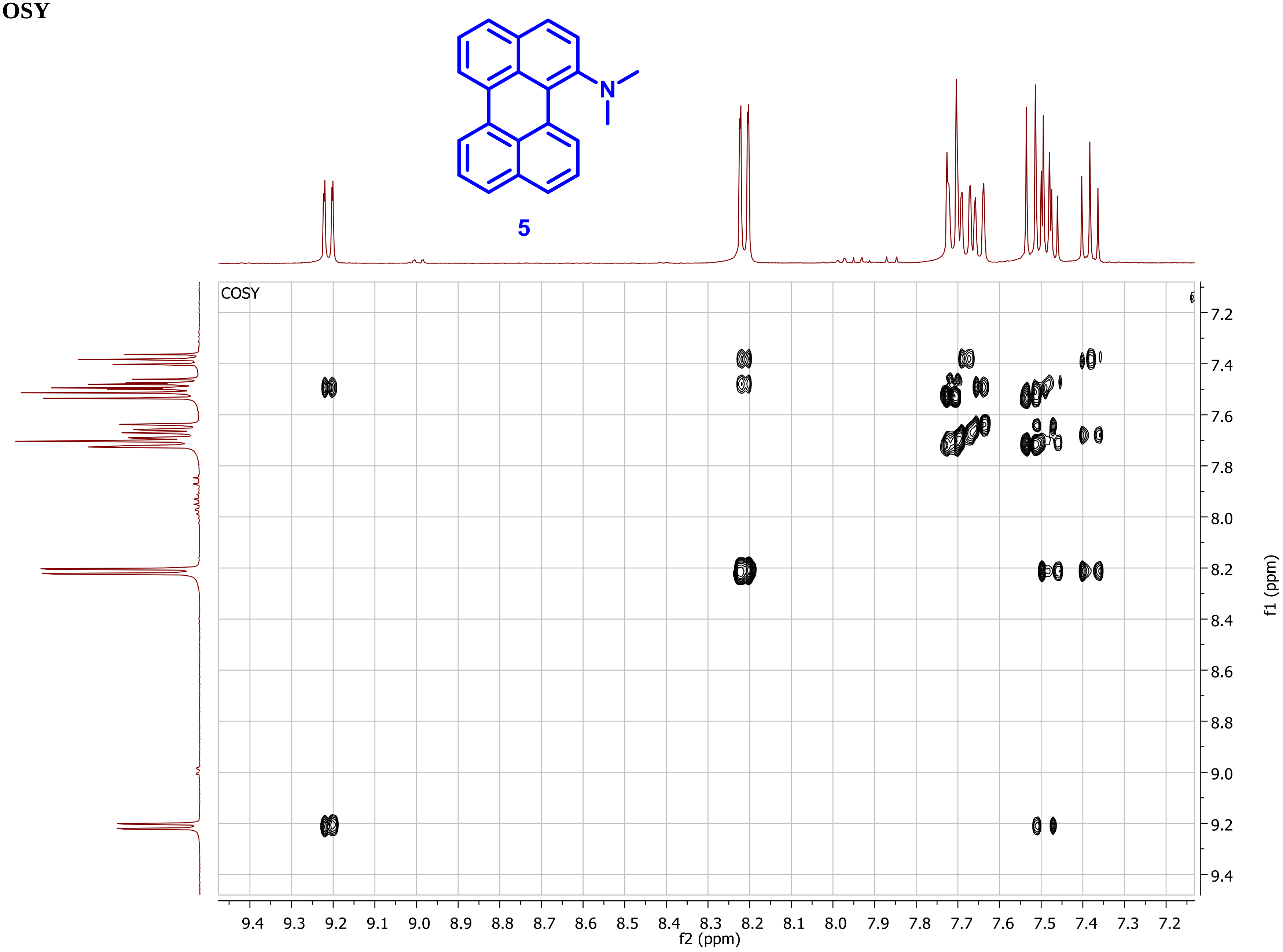
N
5
COSY
9.4
9.3
9.2
9.1
9.0
8.9
8.8
8.7
8.6
8.5
8.4
8.3
8.2
8.1
8.0
7.9
7.8
7.7
7.6
7.5
7.4
7.3
7.2
f2 (ppm)
f1 (ppm)

HSQC-DEPT

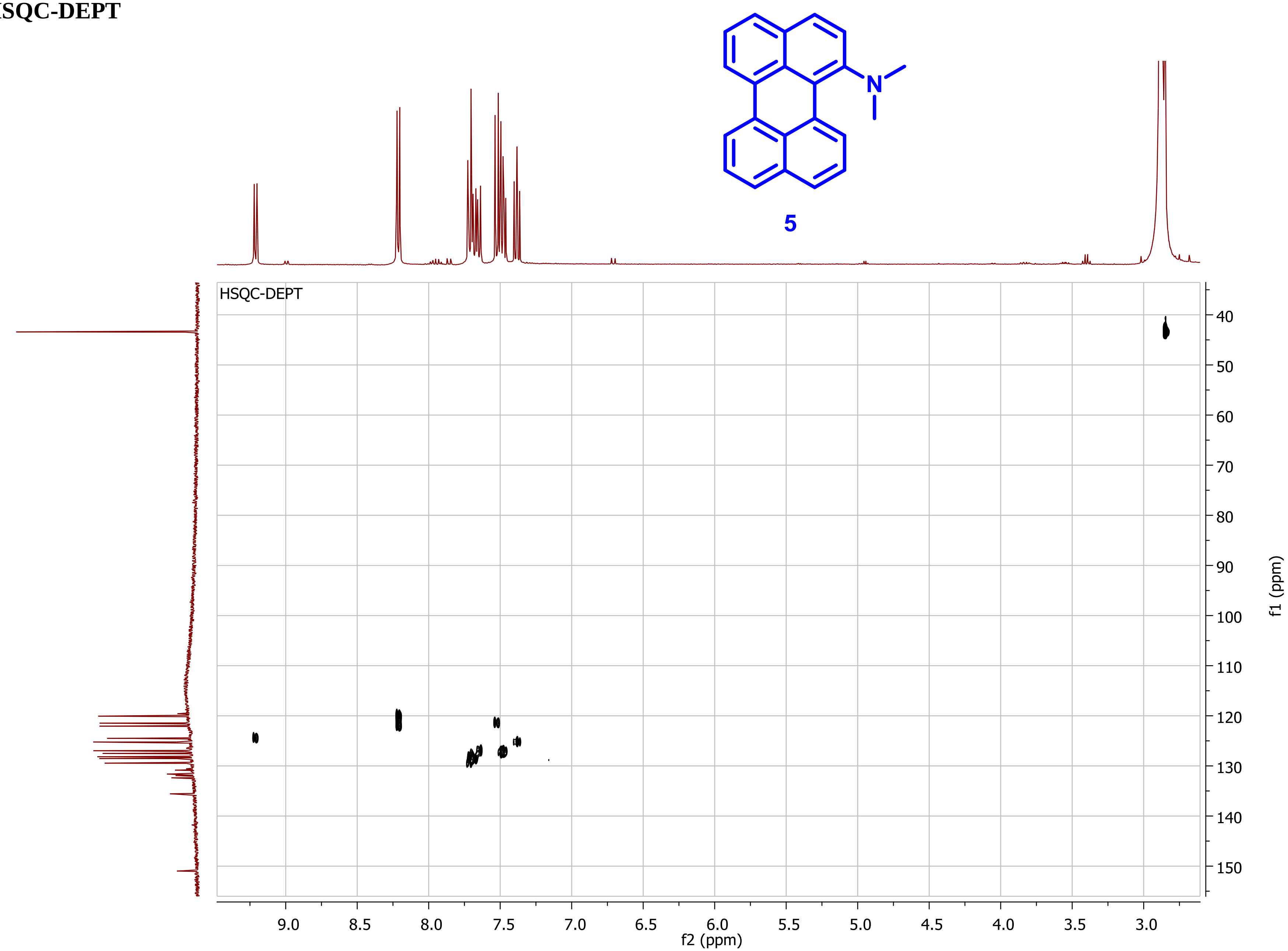
5
HSQC-DEPT
f1 (ppm)
f2 (ppm)
40
50
60
70
80
90
100
110
120
130
140
150
9.0
8.5
8.0
7.5
7.0
6.5
6.0
5.5
5.0
4.5
4.0
3.5
3.0

HMBC

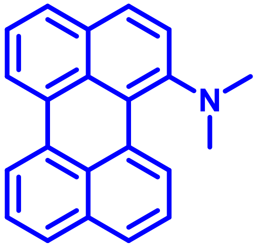
5

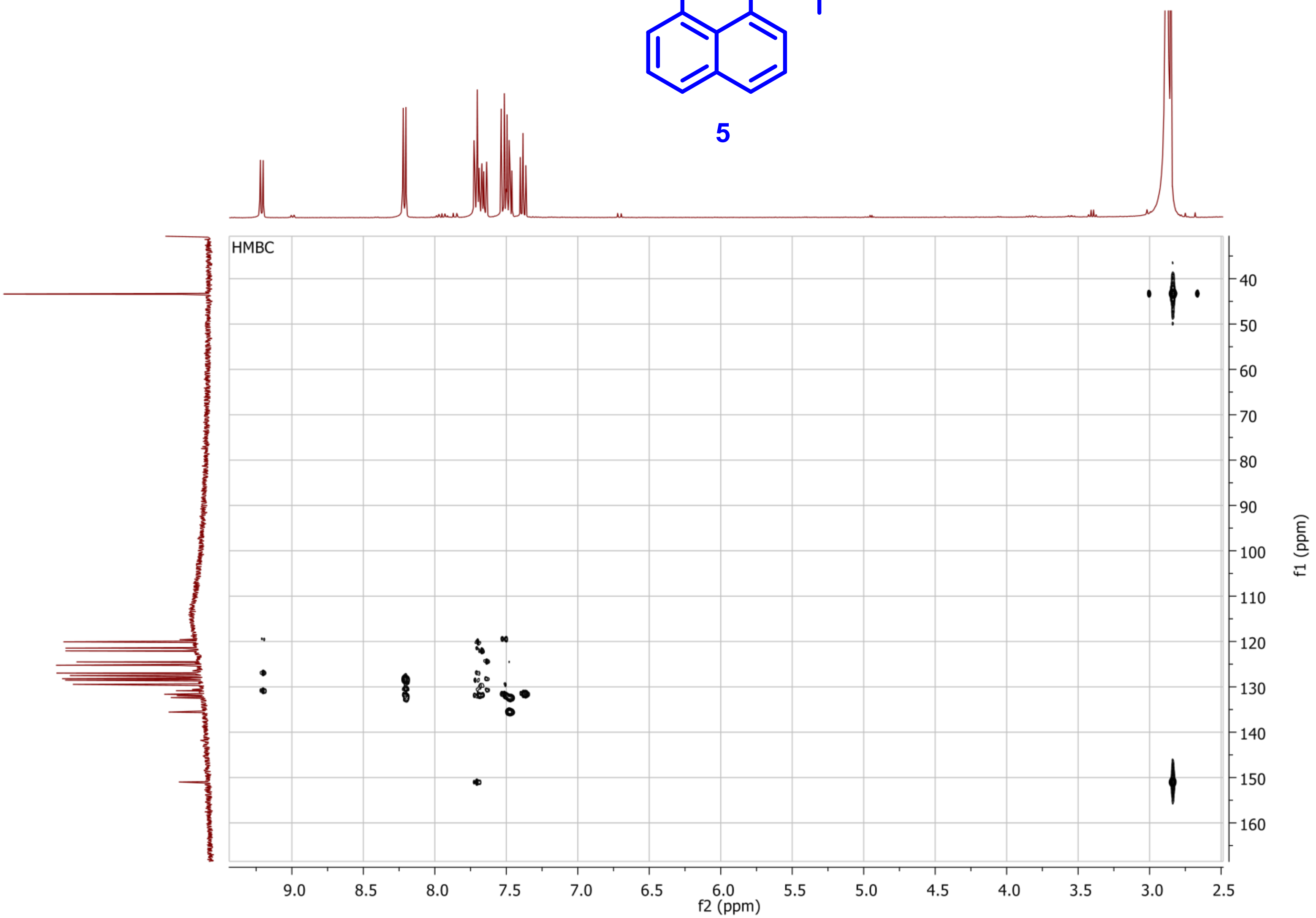

HMBC

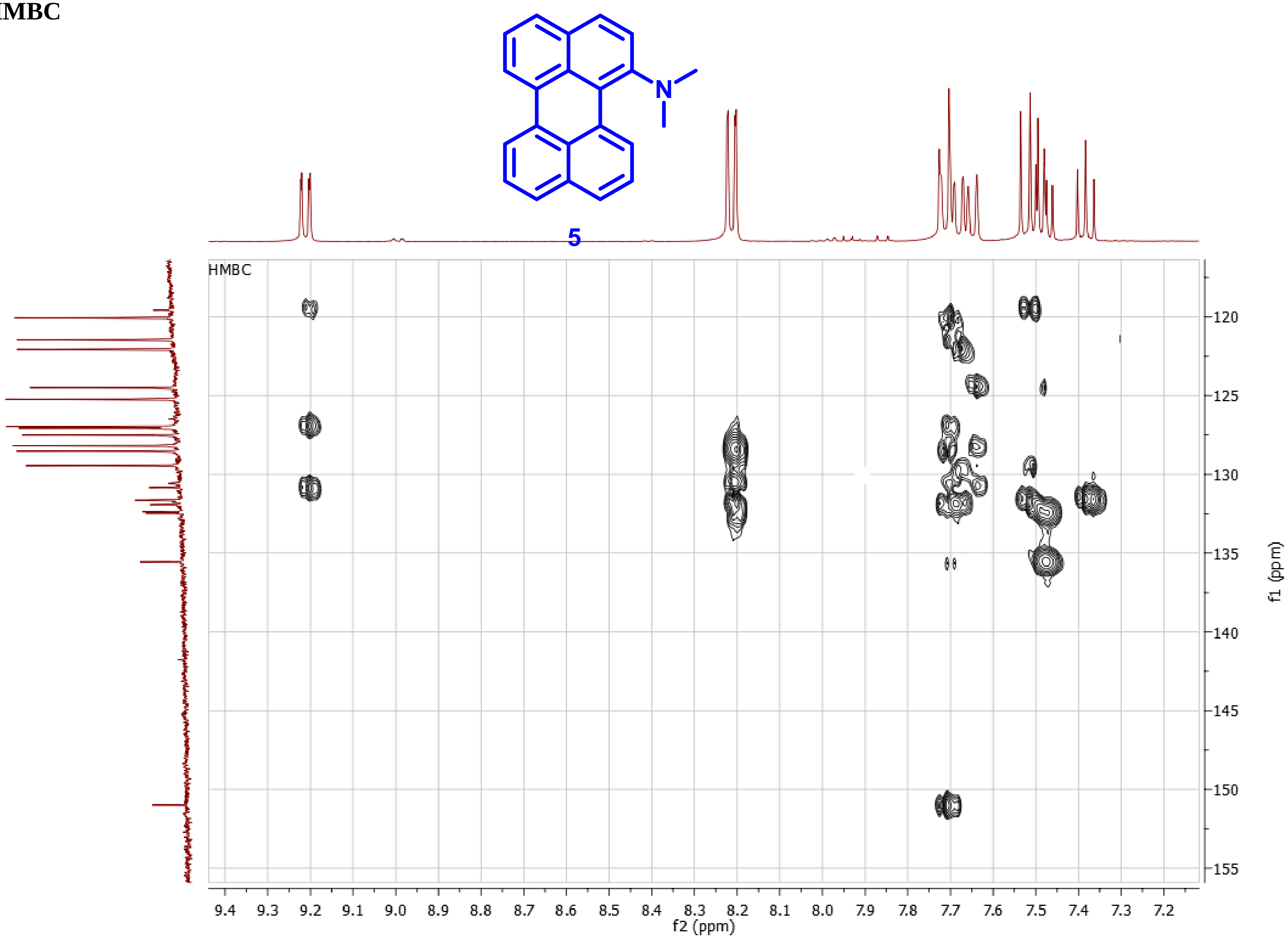

2.6 – **Perylene (7)**

^{1}H-NMR (400 MHz, chloroform - d_1)

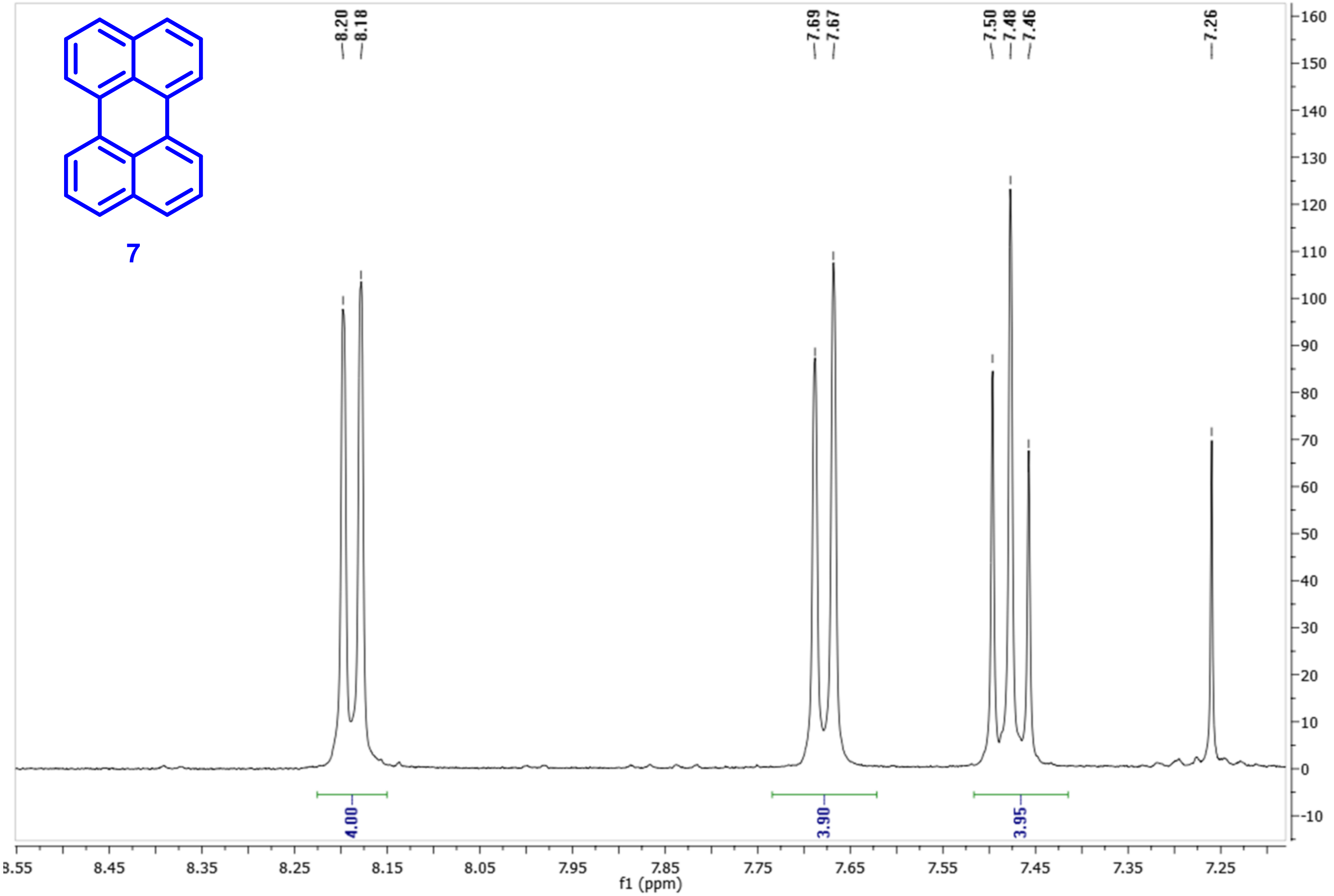

^{13}C-NMR (100 MHz, chloroform-d_1)

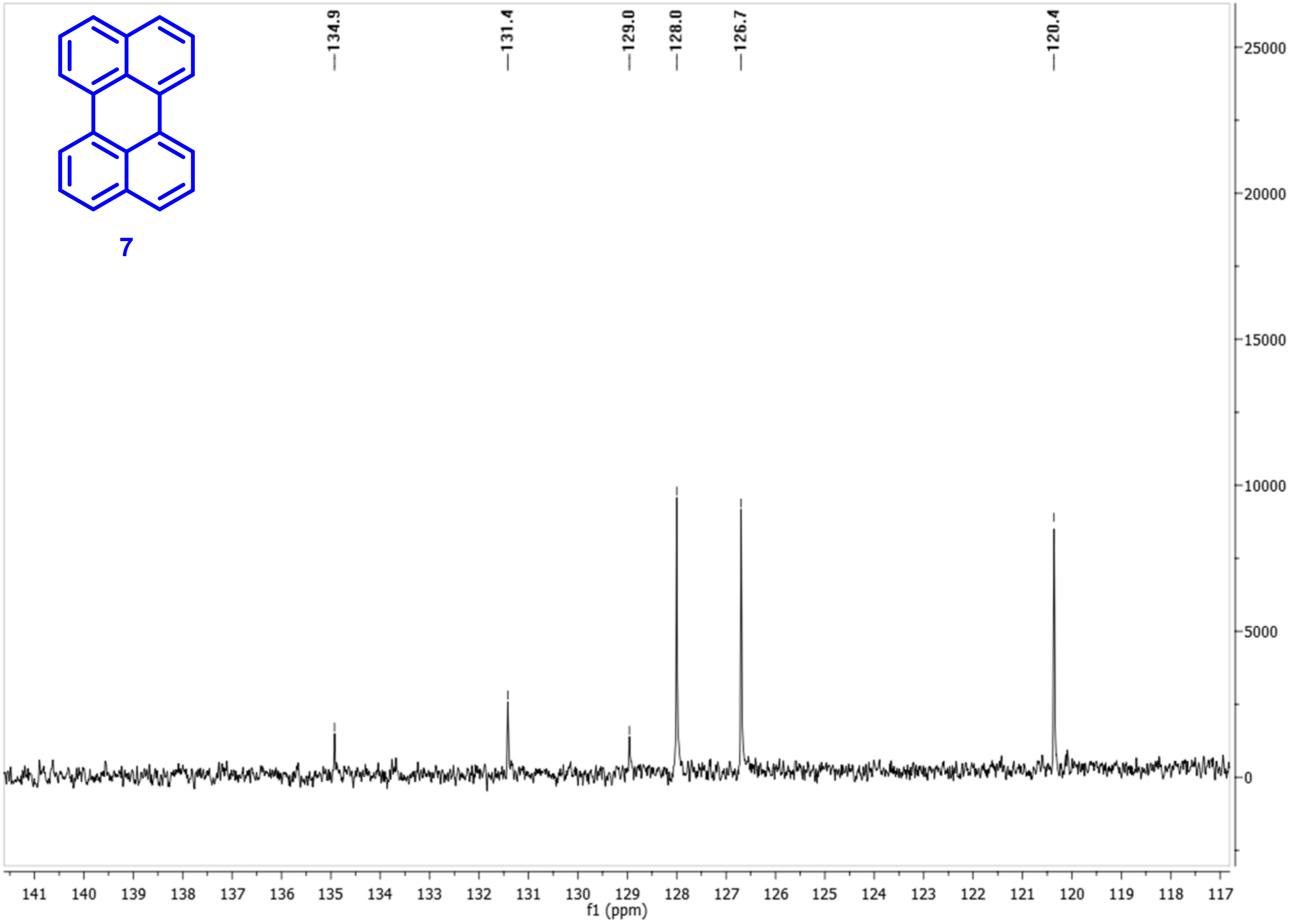

2.7 **– 1,1': 4',1"- Ternaphthalene (25)**

^{1}H-NMR (400 MHz, dimethyl sulfoxide - d_6)

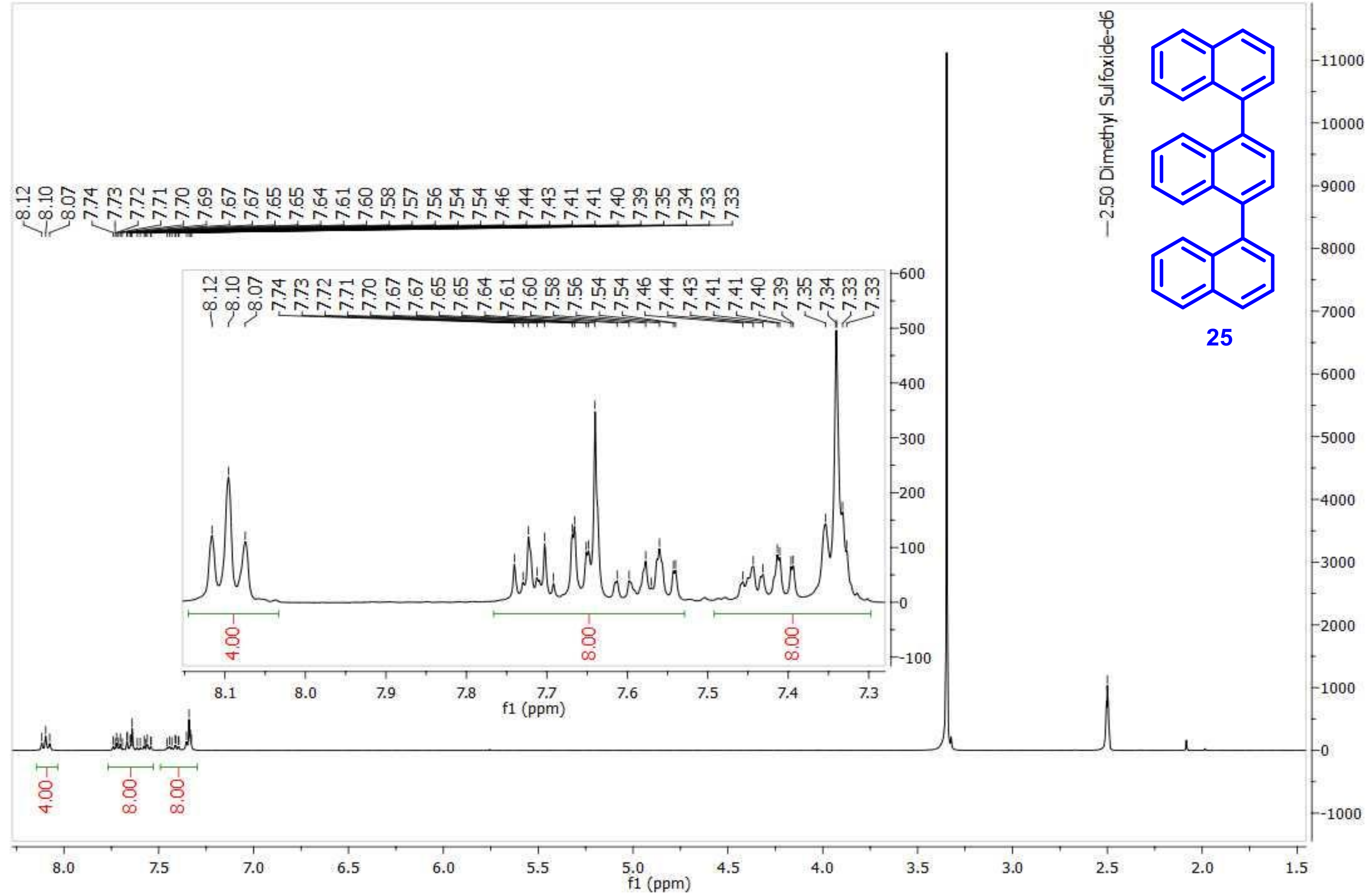

2.8 – Terrylene (26)

UV-Visible spectrum (max plot: 290 nm – 600 nm), λ_{max} (dichloromethane/nm): 550.4, 508.9, 476.1.

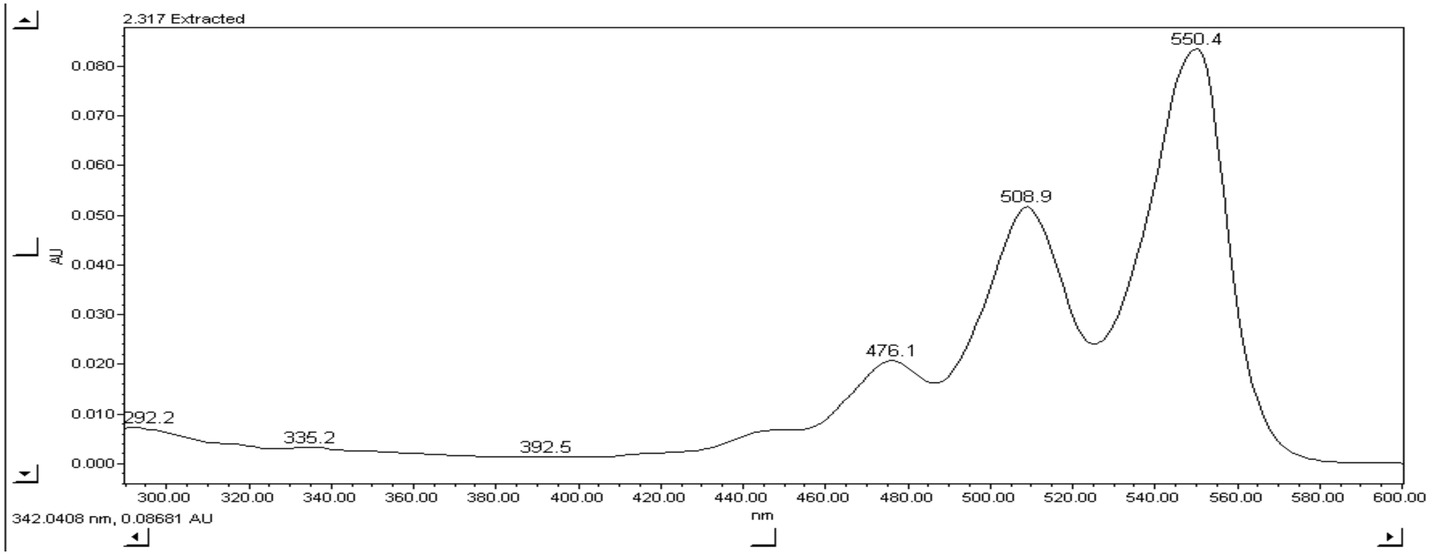

HPLC profile of the crude (Retention time of the terrylene (26): 2,325 minutes)

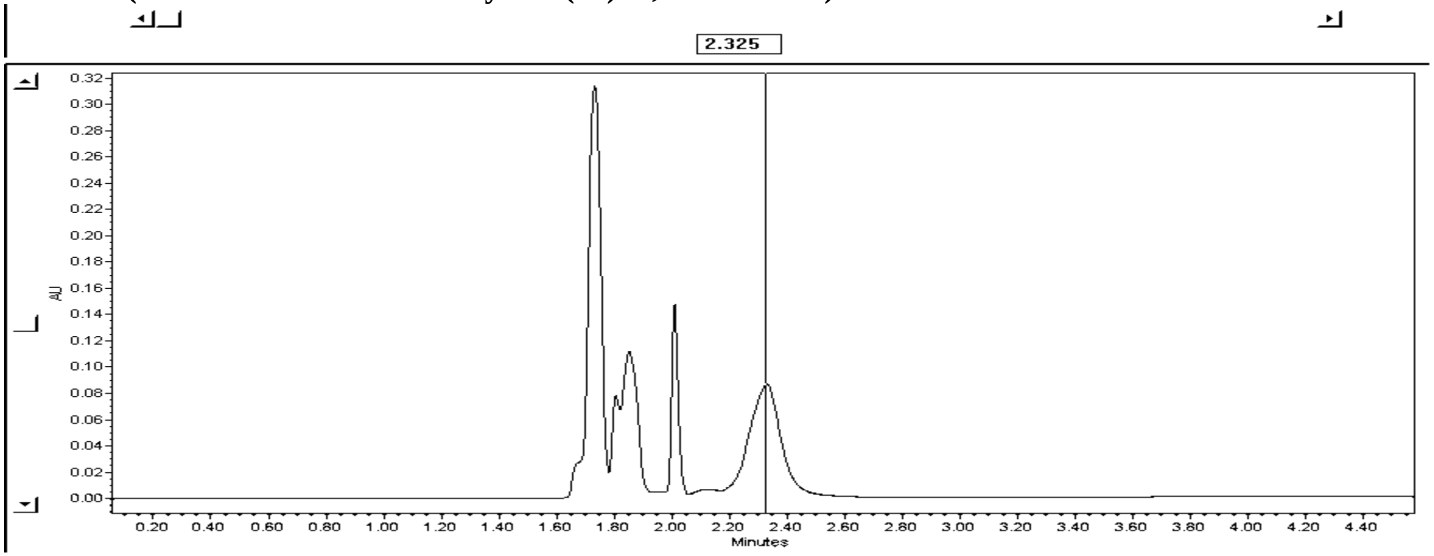

3. Boltzman population analysis for the formation of the σ-complex for 1a, 3, 8a, 9a and 25 at 170 °C.

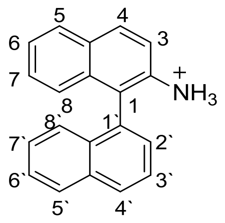

1a	POSITION	*Free energy* (hartree)	*DISTRIBUTION* (%)
	3	-826,2282921	0,002
	4	-826,2324889	0,042
	6	-826,2295532	0,005
	7	-826,231729	0,024
	8	-826,2367836	0,906
	1'	-826,2183974	0,00
	1'	-826,2221522	0,000
	2'	-826,230442	0,009
	3'	-826,2352079	0,295
	4'	-826,2367529	0,887
	6'	-826,2356459	0,403
	7'	-826,234777	0,217
	8'	-826,2410341	18,744
	5'	-826,2429349	72,627
	5	-826,2393963	5,834

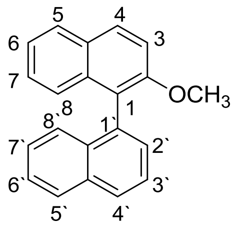

3	POSITION	*Free energy* (hartree)	*DISTRIBUTION (%)*
	1	-884,9680662	98,028
	1'	-884,9496278	0,000
	4'	-884,9595187	0,221
	3	-884,9586138	0,116
	4	-884,9566046	0,027
	6	-884,9621101	1,406
	5'	-884,9576465	0,058
	7	-884,9457156	0,000
	5	-884,9568769	0,033
	8	-884,9580566	0,078
	2'	-884,9524577	0,001
	3'	-884,9510377	0,000
	6'	-884,9518688	0,000
	7'	-884,9525107	0,001
	8'	-884,9563727	0,023

8a	**POSITION**	***Free energy* (hartree)**	***DISTRIBUTION (%)***
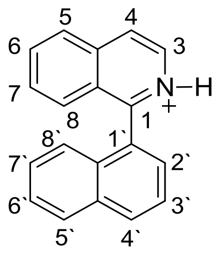	2'	-786,9548501	0,00
	3	-786,9370503	0,00
	3'	-786,9632236	0,86
	4	-786,9441799	0,00
	4'	-786,9616616	0,28
	5	-786,952354	0,00
	5'	-786,9682493	30,79
	6	-786,9319044	0,00
	6'	-786,9641105	1,61
	7	-786,9446108	0,00
	7'	-786,962033	0,37
	8	-786,9451072	0,00
	8'	-786,9693208	66,08
	1	-786,9145461	0,00
	1'	-786,9539631	0,00
	1'(isomer)	-786,9535537	0,00

9a	**POSITION**	***Free energy* (hartree)**	***DISTRIBUTION (%)***
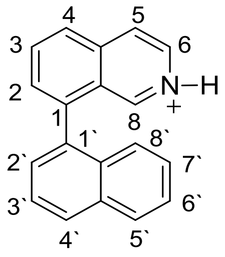	1'	-786,9599028	0,006
	1' (isomer)	-786,9596527	0,005
	1	-786,9402599	0,000
	2	-786,9493587	0,000
	2'	-786,963592	0,094
	3	-786,9312256	0,000
	3'	-786,9666114	0,814
	4	-786,9586207	0,002
	4'	-786,9702838	11,142
	5	-786,9342957	0,000
	5'	-786,9722998	46,887
	6	-786,936962	0,000
	6'	-786,9667391	0,8917
	7'	-786,9664098	0,7052
	8'	-786,9720572	39,4444
	8	-786,9216706	0,00

25	POSITION	*Free energy* (hartree)	*DISTRIBUTION (%)*
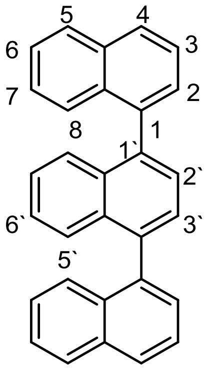	1	-1154,942848	0,012
	1 (isomer a)	-1154,942902	0,012
	1 (isomer b)	-1154,942579	0,010
	1 (isomer c)	-1154,942535	0,009
	1'	-1154,945507	0,080
	1' (isomer a)	-1154,946398	0,152
	1' (isomer b)	-1154,945421	0,075
	1' (isomer c)	-1154,944482	0,038
	2	-1154,947452	0,322
	3	-1154,947285	0,286
	4	-1154,954901	65,068
	5	-1154,95249	11,672
	6	-1154,946967	0,228
	7	-1154,947646	0,369
	8	-1154,952525	11,966
	5'	-1154,952153	9,179
	6'	-1154,947341	0,297
	2'	-1154,946895	0,216

4. *Representation of electrostatic potential isosurface of 3σ. Protonated at position 1 (most stable), left side. Protonated at position 5’, right side.*

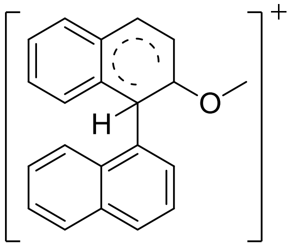

3σ C-1

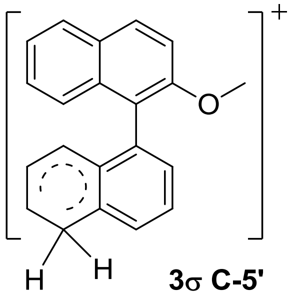

3σ C-5'

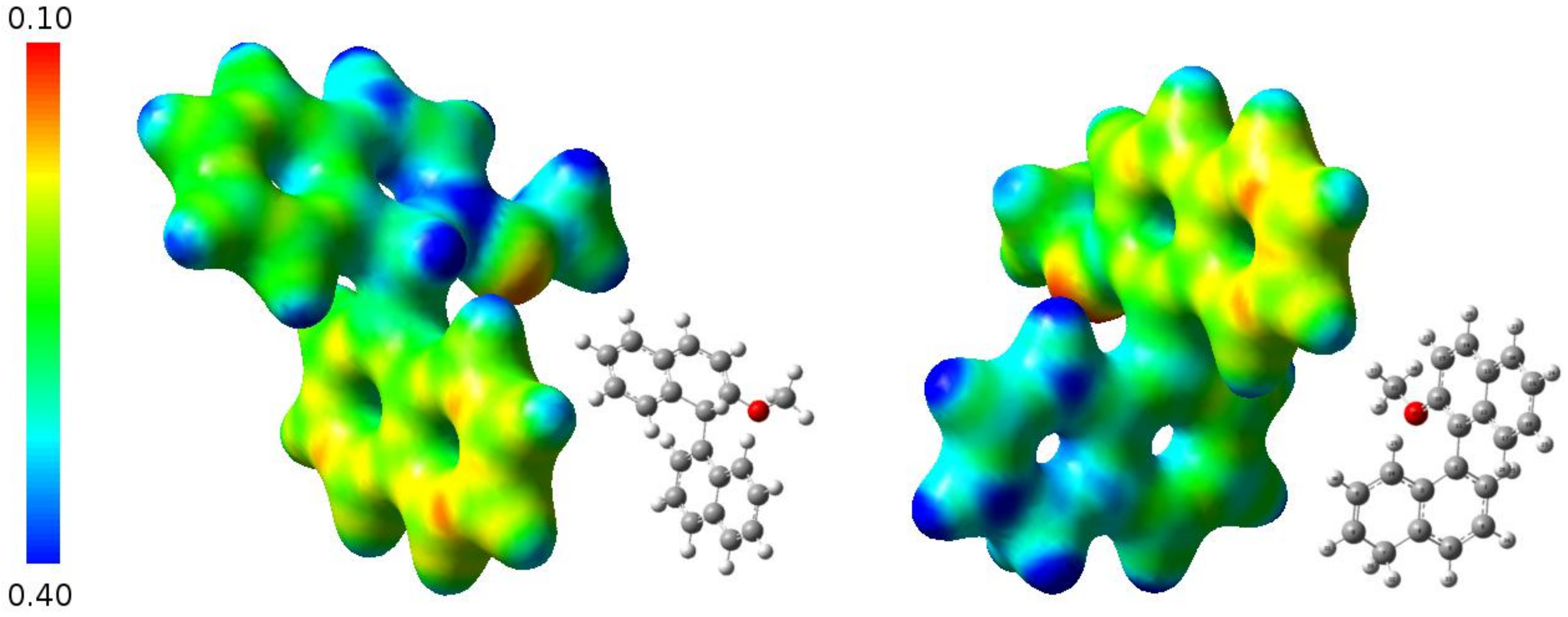

5. *Comparison between the free energies of reaction (ΔG_r) and activation ($\Delta G^{\#}$) for the ring closure by Scholl reaction of the complexes 1σ, 2σ, 8σ, 9σ and 25σ calculated in N-methylformamide and dichloromethane.*

	N-methylformamide (ε ~ 181.5)		Dichloromethane (ε ~ 8.93)		
**σ-complex**	**ΔG_r** (kcal/mol)	**$\Delta G^{\#}$** (kcal/mol)	**ΔG_r** (kcal/mol)	**$\Delta G^{\#}$** (kcal/mol)	**Intermediary**
**1σ**	19.1	24.5	16.3	21.18	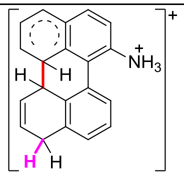
**2σ**	18.3	23.1	16.1	21.46	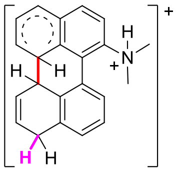
**25σ**	11.13	19.79	11.97	19.97	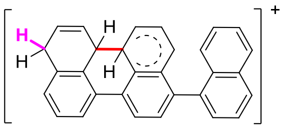
**8σ**	23.2	26.1	23.21	24.47	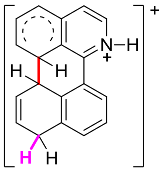
**9σ**	39.9	40.3	40.06	40.41	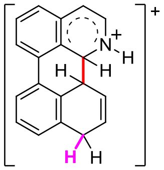

6. Description of the procedure employed for the calculation of Redox Potential. Taken from Ref. 38.

The overall calculated reaction is presented in the thermodynamic cycle shown in scheme 1:

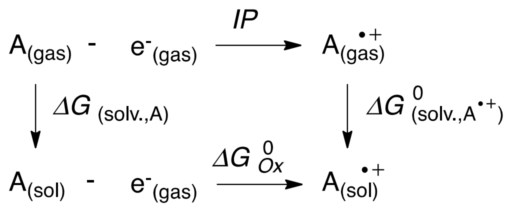

scheme 1

In some cases the $\Delta\Delta G^o_{(Solv)}$ was calculated over the gas phase geometries, in other cases different solvent models were employed to improve its quality and hence the ΔG^o_{Ox}. In the present work the ΔG^o_{Ox} was directly calculated from the optimized geometries in solvent by employing the IEF-PCM continuum solvation model,[1] including the thermal corrections and the changes in enthalpy and entropy.

Finally, the ΔG^o_{Ox} is related to the redox potential, E^o_{Ox}, by the Faraday's equation:

$$E^o_{Ox} = -\Delta G^o_{Ox} / nF$$

where n is the number of electrons transferred (in this case is equal to 1) and F is the Faraday constant (96485 C mol^{-1} or 23.061 kcal mol^{-1} V^{-1}).

It should be noted that the E^o_{Ox} represents the absolute value of half of the redox reaction, a hemi-couple, whereas the experimental values ($E^o_{Ox.Exp}$) are relative to a reference hemi-couple where the oxidation takes place $E^o_{Reference}$, i.e. H^+/H_2, ferricenium/ferrocene (II/III), Ag/Ag^+, Hg/Hg^{+2} (Saturated Calomel Electrode, SCE); according to the following equation:

$$E^o_{Ox.Exp} = E^o_{Ox} - E^o_{Reference}$$

Because of that, the difference between the computed and the experimental values corresponds to the absolute value of the oxidation potential of the reference electrode ($E^o_{Reference}$). If all the experimental values are compared to the H^+/H_2 reference the $E^o_{Reference}$ recommended is 4.44 V in water[2] and 4.48 V in acetonitrile.[3] The usual procedure is to subtract this experimental value to the computed E^o_{Ox} in order to get a computed value comparable with the $E^o_{Ox.Exp}$.

References:

1-J. Tomasi, B. Mennucci and R. Cammi, *Chem. Rev.*, 2005, **105**, 2999-3093.
2-S. Trasatti, *Pure Appl. Chem.*, 1986, **58**, 955-966.
3-This is a computed value. C.P. Kelly, C.J. Cramer, and D.G. Truhlar, *J. Phys. Chem. B*, 2007, **111**, 408-422.

7. Name of the compounds used as references in theoretical studies by aromatic oxidative coupling.

Compound Number	Compound Name	Oxidation Potential (eV)	Ref.
	**Group 1**		
**10**	1,2-bis(3,4-dimethoxyphenyl) ethane-1,2-dione	4.91	[4]
**11**	*N*-benzyl-3,4-dimethoxyaniline	4.98	[4]
**12**	3-(naphthalen-1-yl) perylene	5.34	[5]
**13**	3,3'',4,4''-tetramethoxy-1,1':2',1''-terphenyl	5.48	[4]
**14**	6-methoxy-2-methyl-1-(3,4,5-trimethoxyphenethyl)-1,2,3,4-tetrahydroisoquinolin-7-ol	5.42	[4]
**15**	4,5-bis(3,4-dimethoxyphenyl) isoxazole	5.79	[4]
**16**	4,4',5''',4''''-tetra-*tert*-butyl-1,1':2',1'':4'',1''':2''',1''''-quinquephenyl	5.86	[4]
DDQ	2,3-dichloro-5,6-dicyanobenzoquinone	5.74	

Compound Number	Compound Name	Oxidation Potential (eV)	Ref.
	**Group 2**		
**17**	2,2''-dimethoxy-1,1':2',1''-terphenyl	5.67	[4]
**18**	dimethyl 2,2'-dihydroxy-[1,1'-binaphthalene]-3,3'-dicarboxylate	5.81	[6]
**19**	[1,1'-binaphthalene]-2,2'-diol	5.90	[4]
**20**	1,1'-binaphthalene	5.97	[4]
**21**	6,6'-dibromo-[1,1'-binaphthalene]-2,2'-diol	6.02	[6]
**22**	7-hydroxy-4-(naphthalen-1-yl)-2H-chromen-2-one	6.22	[4]
**23**	2,2''-dibromo-1,1':2',1''-terphenyl	6.51	[4]
**24**	3,3''-dinitro-1,1':2',1''-terphenyl	6.71	[7]
**8**	1-(naphthalen-1-yl) isoquinoline	6.61	[4]
**9**	8-(naphthalen-1-yl) isoquinoline	6.85	[4]

References:

4- M. Grzybowski, K. Skonieczny, H. Butenschön, D. T. Gryko, *Angew. Chem., Int. Ed.*, 2013, **52**, 9900-9930.
5- Y. Avlasevich, C. Kohl, K. Müllen, *J. Mater. Chem.*, 2006, **16**, 1053-1057.
6- A. A. O. Sarhanw, C. Bolm, *Chem. Soc. Rev.*, 2009, **38**, 2730-2744.
7- B. T. King, J. Kroulík, C. R. Robertson, P. Rempala, C. L. Hilton, J. D. Korinek, L. M. Gortari, *J. Org. Chem.*, 2007, **72**, 2279-2288.

8. *Formation of Terrylene: study of Scholl reaction*.

8.1. Calculated energies for the first cyclization step from isomers σ_4 and σ_5 of protonated 25.

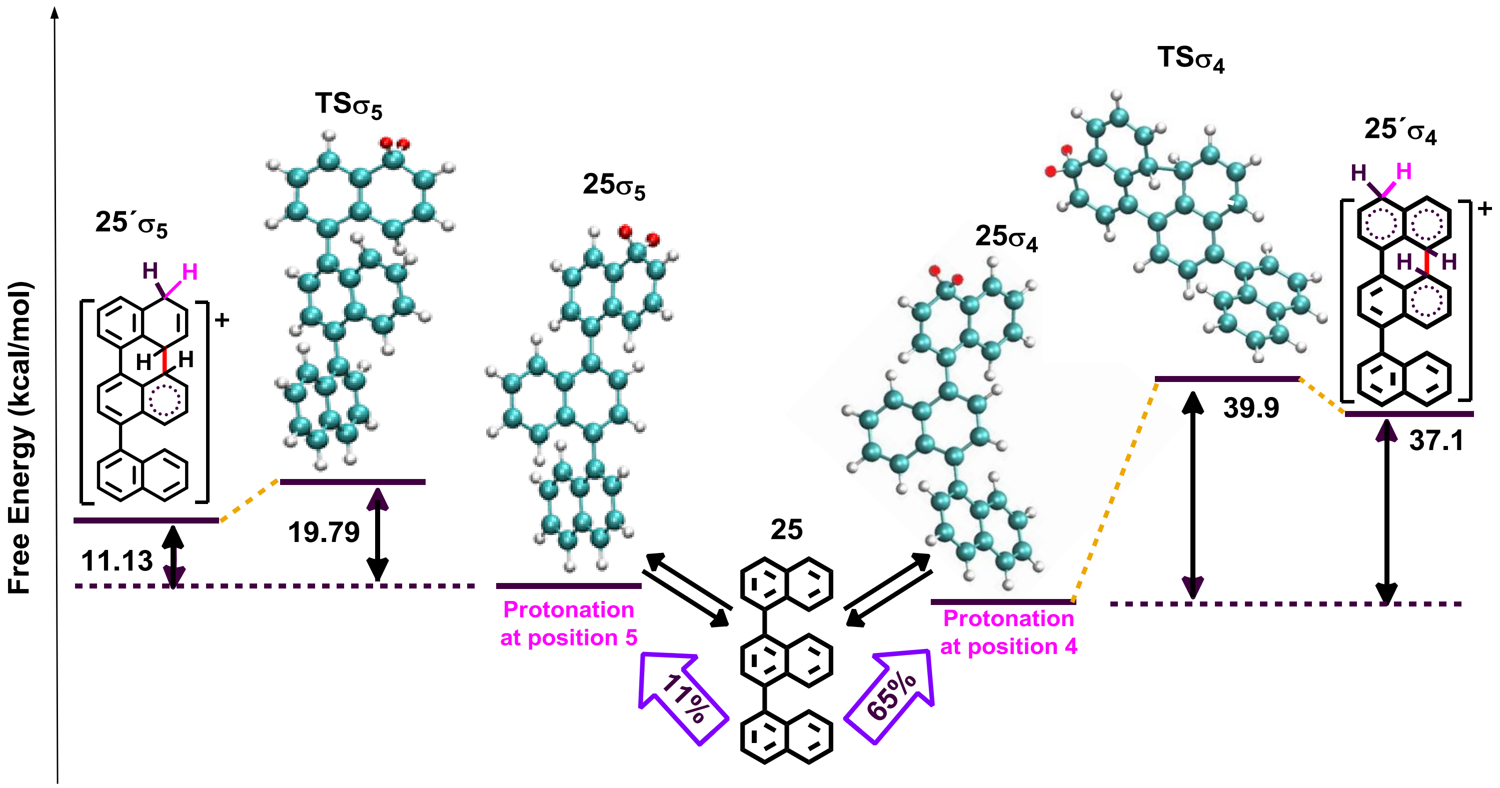

In red are represented both hydrogens at position 5, that originally formed **25σ$_5$** complex.

8.2 Calculated energies involved in both ring closures of 25σ by the Scholl mechanism, σ-complex generated in position 5.

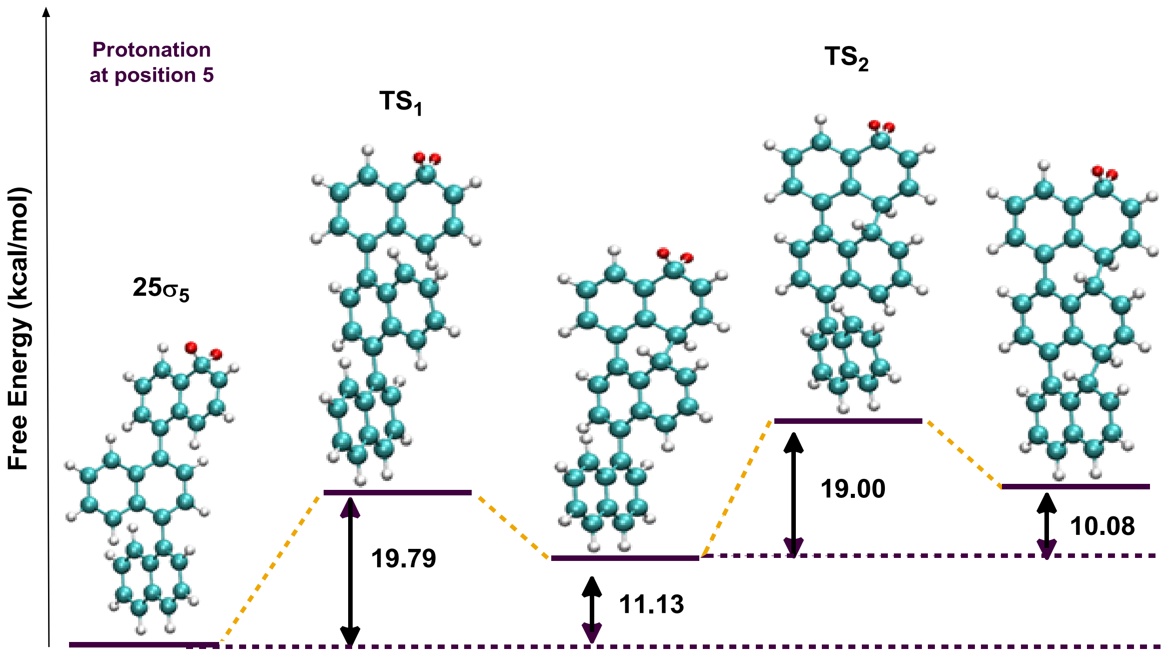

Double cyclization of the substrate 1,1':4',1''-ternaphthalene by Scholl mechanism. **25σ**$_5$ is the σ-complex of **25** after protonation at C-5, TS_1 and TS_2 are the transition states for each cyclization step. In red are represented both hydrogens at position 5.

9- Electrostatic potential isosurface of 25σ and the intermediary product of cyclization to afford terrylene.

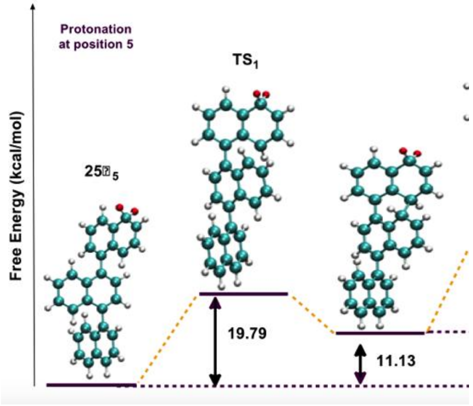

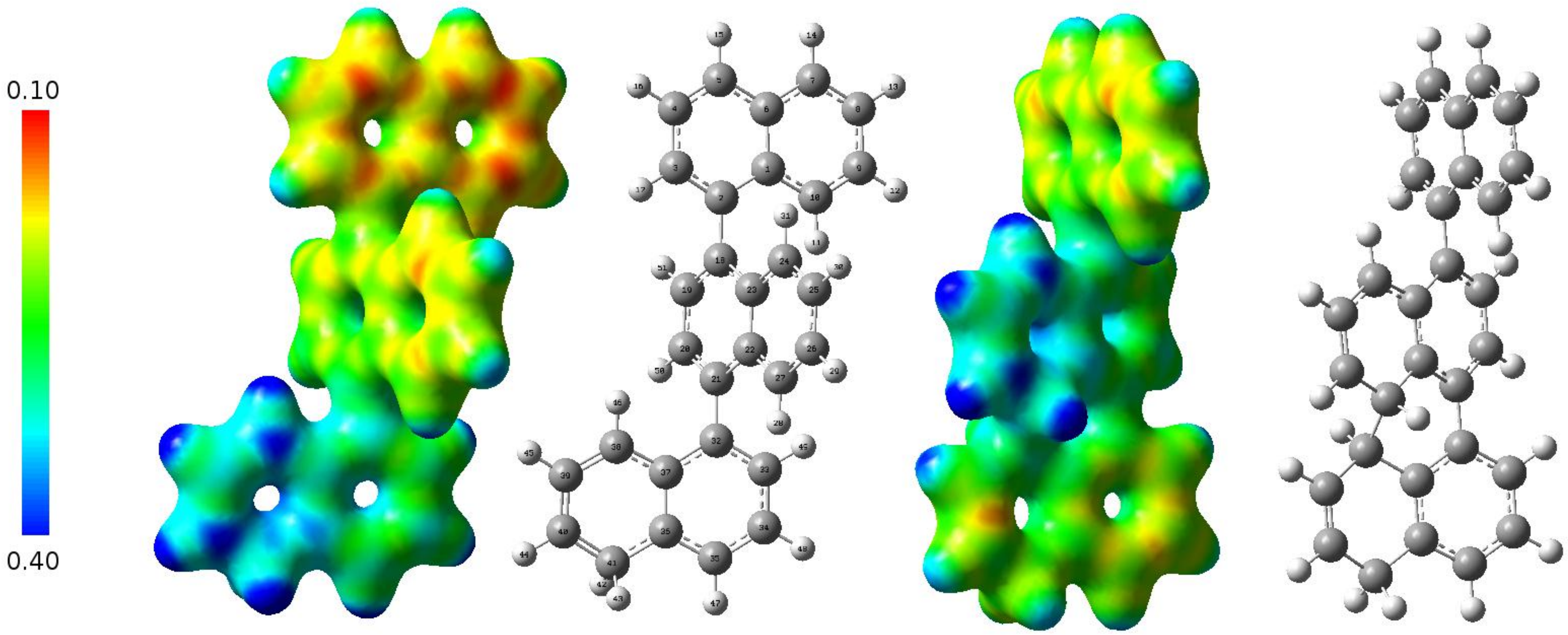

10. *XYZ Coordinates of the computed compounds*

1a-sigma-1’.xyz

```
38
-826.465870231
 C       -0.022629446688     -0.180555373409     -0.027340912632
 C       -0.065290512103     -0.128727370202      1.387773909520
 C        1.157530273949     -0.029456579044      2.125659943529
 C        2.410874767581      0.016308538320      1.425396232187
 C        2.373463084687     -0.032050343765      0.045557797394
 C        1.174354583554     -0.131723271319     -0.691164980600
 C        1.077308223170      0.020149414244      3.541588377941
 C       -0.137001927535     -0.032739760952      4.190033115111
 C       -1.339843295889     -0.134264443308      3.456687602877
 C       -1.301266097920     -0.180406148044      2.082157803047
 C        3.697075277908      0.089142131792      2.243063568489
 C        3.930465414572      1.384544282456      2.978024582440
 C        4.613137522426      1.351099040826      4.233779885594
 C        4.946791477558      0.113182832203      4.812275774467
 C        4.611443193970     -1.124534425656      4.230241157639
 C        3.964844080385     -1.146972676488      3.025829267653
 C        4.915221702758      2.561727199839      4.912851015174
 C        4.542206821520      3.770204475708      4.363772894228
 C        3.866078240994      3.790480538505      3.131522990000
 C        3.568022811188      2.614570777734      2.442019129304
 N        3.592739017609      0.018989816896     -0.786633098728
 H        4.460158988128      0.142778084636     -0.259146681284
 H        3.554536236241      0.796561721842     -1.456568035691
 H        3.707904510098     -0.842156756953     -1.334926856136
 H        4.570335408929      0.041253703915      1.559213247177
 H        3.041371914895      2.669047724596      1.494174339593
 H        3.564183545481      4.742535808659      2.703940263345
 H        4.764654800181      4.700654138748      4.876218837323
 H        5.437439340457      2.516551641795      5.864395439045
 H        5.469014601824      0.116366106324      5.766416324550
 H        4.857364779936     -2.047581309470      4.743981718612
 H        3.701314276525     -2.091158413518      2.555949486233
 H       -2.219350808214     -0.258376713075      1.505233099023
 H       -2.290892473209     -0.174387545761      3.980298310108
 H       -0.167252546058      0.007398606107      5.275287488531
 H        1.978083615921      0.107815043473      4.139786645072
 H        1.206698085662     -0.172234991387     -1.776149522508
 H       -0.950490465695     -0.260448360181     -0.586635555548
```

1a-sigma-1.xyz

```
38
-826.460515448
 C        0.005395932161     -0.328469639787     -0.036152584321
 C       -0.068140601461     -0.193720119608      1.350171989328
 C        1.126809597869      0.072281940321      2.098449100713
 C        2.461675476294      0.088616961006      1.402937619198
 C        2.359147806420      0.043005063471     -0.080935175643
```

C	1.214648713526	-0.191786842977	-0.766240738530
C	1.032287096801	0.240340473344	3.472555437495
C	-0.212194055874	0.153326150748	4.099290678031
C	-1.392000060654	-0.110796942125	3.377189512918
C	-1.325618095139	-0.285008287945	2.014174462892
C	3.479988808626	1.097533102038	1.936315901422
C	3.236455143667	2.505962691706	1.841480209504
C	4.225263549832	3.399457463910	2.370642339133
C	5.401511372270	2.873081181315	2.967069479475
C	5.609160378730	1.516814710881	3.039643135903
C	4.641609236407	0.627501446486	2.520264258434
C	4.008577104835	4.799702139534	2.287326126871
C	2.868276041657	5.306554611678	1.706192191940
C	1.892724968881	4.427754753675	1.183495857002
C	2.072263020324	3.063187523023	1.252298208811
N	3.623767929259	0.149256085371	-0.809050876218
H	4.317374249657	0.683587939033	-0.264114964690
H	4.033987606167	-0.778801882727	-0.992007215257
H	3.514529249408	0.615073583837	-1.718665639040
H	2.885632925749	-0.910694012396	1.646192952221
H	1.297986177623	2.415000735353	0.851030147449
H	0.992088720376	4.829927887682	0.727607816202
H	2.713610070313	6.380551430749	1.648922100968
H	4.764680057385	5.468193447820	2.692456520352
H	6.139730640189	3.564894425660	3.365662676967
H	6.509672653160	1.117090773021	3.496473129651
H	4.817620630602	-0.443456652029	2.592494561611
H	-2.217592116651	-0.489773484510	1.429448327189
H	-2.342547602418	-0.174502153220	3.896452627697
H	-0.268971486030	0.295503462889	5.174810866756
H	1.920802697752	0.445720299985	4.061333340974
H	1.195356859130	-0.277999535133	-1.847618595075
H	-0.904138226080	-0.531900482388	-0.595921493932

1a-sigma-2’.xyz

38

-826.471109032

C	-0.007839597447	-0.038652089585	-0.006179893216
C	-0.076440587113	0.032007600389	1.407204762636
C	1.131906757212	0.022455688031	2.174072203217
C	2.392089679165	-0.039727644277	1.489881565225
C	2.385403961213	-0.122332258927	0.110672350387
C	1.201013144248	-0.120009348565	-0.650059843199
C	1.039002608763	0.052802086074	3.590696661930
C	-0.188323777712	0.118786538454	4.212259534846
C	-1.380539315907	0.149926078699	3.453452763599
C	-1.324073827591	0.101828246750	2.079568539032
C	3.648141260438	0.028828416296	2.287519574742
C	4.372159055679	-1.104185497640	2.634524199638
C	5.590366905469	-0.954563560818	3.417455870794
C	6.029932770484	0.337751809954	3.845397590341
C	5.316431164988	1.447096292002	3.537991285386
C	4.089234058654	1.367764127300	2.729084152743
C	6.314137793269	-2.109061168622	3.755132897445

C	5.866290450115	-3.358873563969	3.364420593679
C	4.668746947931	-3.520619092806	2.622010607581
C	3.935760833309	-2.422248663228	2.263738338399
N	3.645154518372	-0.170601913286	-0.657392731940
H	3.622980923614	-0.914050002041	-1.365502816236
H	4.472702846225	-0.352022126455	-0.079963003279
H	3.819234072421	0.711159448387	-1.156404729074
H	4.195737167741	2.020733900201	1.841568592392
H	3.250531028868	1.871651160146	3.244400210963
H	3.011732708186	-2.536020025127	1.705664993407
H	4.337068376763	-4.516362535422	2.346201873307
H	6.440008953640	-4.239498804014	3.639575551277
H	7.227651849076	-2.012674497730	4.333894750727
H	6.941072473129	0.405192292219	4.432373809696
H	5.634929333928	2.430768198503	3.869721563770
H	-2.235266139725	0.112788180840	1.486850693658
H	-2.340409222473	0.204230247240	3.959351831851
H	-0.239420455189	0.141593901971	5.297250305582
H	1.937004097790	0.008670040232	4.200042231631
H	1.250336347177	-0.175648328678	-1.733678328138
H	-0.927758909587	-0.032421431373	-0.583797391985

1a-sigma-3’.xyz

38

-826.477509893

C	0.002016831756	0.029144018622	-0.021167701059
C	-0.063345250272	0.037584627102	1.395611415941
C	1.147847413596	-0.008155816824	2.157612685125
C	2.405574040437	-0.050494605014	1.472913502172
C	2.392389130461	-0.060445623801	0.093796583649
C	1.210017460220	-0.021290432696	-0.670421132537
C	1.061182703318	-0.012148236754	3.574714507974
C	-0.163571587739	0.035792346229	4.203733314844
C	-1.358982619568	0.087157534766	3.450360093859
C	-1.308436360441	0.085382837280	2.074859017447
C	3.682538076066	-0.092085557187	2.249090508032
C	4.259046206375	1.141056690746	2.761459599230
C	5.483031537159	1.098749898083	3.534728888718
C	6.093633449440	-0.114647611358	3.774906078798
C	5.545687423257	-1.370327807267	3.275434907277
C	4.295188538856	-1.278842055301	2.499147012421
C	6.055649695860	2.307371788056	4.042464942062
C	5.448531712733	3.513881101061	3.799199070799
C	4.257016737833	3.545981887035	3.042202775128
C	3.673529156249	2.390662657341	2.533248494781
N	3.662533489229	-0.090712446160	-0.653407159576
H	4.467474703734	-0.243240617440	-0.034715628811
H	3.831401717111	0.787493346114	-1.158515898341
H	3.676975107909	-0.845015791226	-1.349430536283
H	6.330696150610	-1.892777371558	2.694660683537
H	5.422434072555	-2.066358181155	4.127940479304
H	2.758770700333	2.465815519503	1.954669991680
H	3.779664850605	4.502778400856	2.849015224237
H	5.874185388358	4.436722016369	4.179265909543

H	6.974591731885	2.246384194645	4.618255991600
H	7.014035288166	-0.153583609640	4.353138242096
H	3.864844367285	-2.207364895688	2.133477542302
H	-2.222944889833	0.120246374567	1.487788407574
H	-2.317162167859	0.125285394835	3.961399306353
H	-0.211484532051	0.032409659903	5.289336697408
H	1.968090039745	-0.059452832316	4.170018173181
H	1.260397757523	-0.028701144917	-1.755382762849
H	-0.919654895413	0.061548863165	-0.595590483097

1a-sigma-3.xyz

38

-826.469794054

C	3.583513832218	-0.213746699087	0.309818291737
C	3.789481584731	-0.334562535489	-1.090281632536
C	3.345467206658	-1.445605963079	-1.773605521284
C	2.678844968291	-2.490527942317	-1.092328589964
C	2.470705800956	-2.405666922585	0.265936463458
C	2.913953803737	-1.274642007777	1.001145516774
C	2.709132276990	-1.177383803903	2.402809009717
C	3.145117427878	-0.079573019608	3.106977710386
C	3.800543437016	0.976659896142	2.434928469629
C	4.018210663856	0.920541593375	1.069134189468
C	4.688136189084	2.070734334614	0.393318889739
C	4.520788037475	4.198209705400	-0.951916963701
C	3.731217816228	5.183922194783	-1.622256673472
C	2.361425534588	5.075850216161	-1.634270612572
C	1.751794096209	3.987538262262	-0.978956882443
C	2.494006842744	3.007794829907	-0.318619212936
C	3.884947454015	3.080670940030	-0.289364870794
C	6.030360540250	2.231802100085	0.410333464365
C	6.749794989738	3.351625173956	-0.232638923978
C	5.892208031395	4.315230661675	-0.919324097178
N	6.867012322256	1.239738334188	1.091310094233
H	7.503134218756	2.991359656079	-0.959042811961
H	7.370984894458	3.912728298711	0.491034168924
H	4.307246136276	0.453760252055	-1.630355078543
H	3.510713965744	-1.521032706258	-2.845151416751
H	2.334509497230	-3.360788263693	-1.644721372163
H	1.962732495203	-3.206867422985	0.797870881411
H	2.200183246802	-1.991250135704	2.914140328412
H	2.986195671121	-0.011630164623	4.179243158765
H	4.125209163195	1.849552031848	2.996169710260
H	4.234182562106	6.012485949866	-2.112007028689
H	1.750645578178	5.818145039204	-2.137065424994
H	0.668452383604	3.906223734864	-0.985340222986
H	1.980515100009	2.189937142519	0.174621247101
H	6.382467897952	5.152527227908	-1.409854086702
H	7.526180286403	0.777224509586	0.452120835743
H	7.425891432580	1.650383125795	1.850573298425
H	6.279395704067	0.506014766092	1.512675405103

1a-sigma-4’.xyz

38

-826.478273939

C	0.024932660000	-0.023145791354	-0.012341098254
C	-0.042001714824	0.036572878591	1.401850546581
C	1.167206572261	0.026266522189	2.168124440903
C	2.423502757634	-0.037014707855	1.479680937027
C	2.416903566708	-0.104854897693	0.101352670995
C	1.233283504781	-0.091564855271	-0.659538454506
C	1.078310621968	0.067681385596	3.584625763088
C	-0.148287660684	0.130899056465	4.207675656807
C	-1.342167172163	0.152435293905	3.450653574736
C	-1.288570736730	0.103680588552	2.076703326479
C	3.688879953914	-0.078718391892	2.266366637873
C	4.247872439499	1.113514270363	2.801302612685
C	5.451909038697	1.030513616746	3.569399807143
C	6.093637689762	-0.289484309472	3.798826284761
C	5.448167742229	-1.451612737773	3.190321230444
C	4.296025932483	-1.342959329117	2.456647998068
C	6.007525887436	2.198289935575	4.088885903378
C	5.396433636924	3.426286295257	3.858987080913
C	4.214973498044	3.520058351425	3.098990262980
C	3.645748109704	2.381368983867	2.574052014950
N	3.681446045320	-0.139656225367	-0.658531967871
H	4.506427807007	-0.300927634552	-0.071059063267
H	3.844706658676	0.742158958849	-1.160441434492
H	3.676721224027	-0.887158276810	-1.362469332601
H	6.195289635857	-0.486832965496	4.882687259663
H	7.150935316756	-0.270154747930	3.476565678807
H	2.738927231032	2.446097191684	1.981682487119
H	3.757593998869	4.489042626293	2.925739284832
H	5.841543148648	4.328189006133	4.269649494458
H	6.921594652120	2.146045418098	4.673689543943
H	5.904505568251	-2.425300439291	3.347583485641
H	3.825852815352	-2.225214246792	2.033502642737
H	-2.201076057472	0.114664561166	1.485938851924
H	-2.301330975208	0.204794702187	3.958194817402
H	-0.198041894216	0.163885213843	5.292513497231
H	1.980448212183	0.047715419866	4.188724663604
H	1.282255859641	-0.128966409285	-1.743877812307
H	-0.895886895381	-0.012959625742	-0.588518626156

1a-sigma-4.xyz

38

-826.474406779

C	3.664891959549	-0.296981411727	0.197093447864
C	3.903461392392	-0.656289048642	1.548398885294
C	3.786892610920	-1.971466515213	1.959123429553
C	3.393638594909	-2.975225944992	1.049623425800
C	3.102083045187	-2.644075359914	-0.256697446261
C	3.223859598187	-1.307698726872	-0.713630157680
C	2.888525180347	-0.950901699631	-2.045652240701
C	2.960611043242	0.358011845041	-2.471445524430
C	3.395665764543	1.362220485549	-1.589495187072

C	3.788795139394	1.049331291739	-0.287290133239
C	4.409967830828	2.113797038087	0.502831067909
C	4.387718347356	4.419731281992	1.401812512813
C	3.692725796720	5.599852159494	1.650763095540
C	2.350152949612	5.712651808229	1.301034663239
C	1.664399923207	4.634262155374	0.717214072343
C	2.330239060000	3.454344607003	0.463015481728
C	3.714285983766	3.329694549790	0.769246925007
C	5.767896833452	1.980711602132	0.948484597466
C	6.445793224561	2.994592903888	1.539459825598
C	5.813770202332	4.289910472186	1.802047131775
N	6.504019142815	0.751380780310	0.618073497492
H	5.935730289529	4.525713970711	2.873514203853
H	6.430930861199	5.064939767900	1.313790525470
H	4.124967261856	0.112019873757	2.287264613233
H	3.978814245652	-2.227604976147	2.997246001473
H	3.299797841385	-4.003244946035	1.387194653057
H	2.764017585586	-3.405084724488	-0.955464473310
H	2.561795443798	-1.730999495866	-2.728710403005
H	2.698803311650	0.622412805065	-3.491254578995
H	3.496314495221	2.381964169910	-1.949377551803
H	4.202592564243	6.433879446720	2.124871545242
H	1.821876149518	6.640107764751	1.503200723367
H	0.607897737271	4.723308877031	0.485005161754
H	1.792398010550	2.607227815972	0.053189782645
H	7.496414154265	2.890071391504	1.799275333712
H	7.448307473511	0.762058983591	1.019337801096
H	6.612050612070	0.630656963417	-0.397724563504
H	6.033434889377	-0.095889301619	0.974685405680

1a-sigma-5’.xyz

38

-826.482838884

C	0.070192157358	0.286383891279	0.076908016632
C	0.004964907396	0.114738203553	1.483981633025
C	1.217964192132	0.103619708622	2.244422184282
C	2.452744337136	0.252389108208	1.559033188828
C	2.481447039976	0.414622595247	0.191239897898
C	1.282421114910	0.435626736275	-0.557553329498
C	-1.237116298655	-0.051126047693	2.148007804582
C	-1.291724202438	-0.226956566338	3.508012582586
C	-0.089964360572	-0.234316088182	4.242962748903
C	1.151651003219	-0.064475493420	3.666397404199
C	2.405143559976	-0.080998124193	4.477204847030
C	2.981147728959	-1.285146125524	4.842952434031
C	4.180548924833	-1.312907426807	5.581116983151
C	4.822919447349	-0.141254462200	5.953186833276
C	4.272600358898	1.092881987822	5.602761817400
C	3.053276828181	1.136608677068	4.866658973573
C	4.940610225722	2.365352683609	5.985472446991
C	4.278974527007	3.612724377817	5.596598678909
C	3.098366069712	3.619947135697	4.899263549471
C	2.505451524419	2.398360295668	4.543346348183

N	-0.201260755918	-0.414731048088	5.701682572702
H	-0.856780954132	0.295490610671	-0.491086949171
H	1.322127606930	0.565843860705	-1.635543074084
H	3.435431351316	0.525250117650	-0.317139371344
H	3.385145736299	0.229523143923	2.115217869647
H	-2.244048407548	-0.355127458173	4.014534327383
H	-2.154838493136	-0.040919655754	1.566439067062
H	1.569404932703	2.427819978489	3.990722957204
H	2.617832403193	4.550891587054	4.618700559473
H	4.759408178280	4.545856953803	5.878861342860
H	5.972220656656	2.387924836754	5.586816230465
H	5.751208823514	-0.181034544472	6.515862886472
H	4.610577821682	-2.272279863574	5.853839296626
H	2.512512969890	-2.219727459533	4.547867262542
H	-0.740116109115	-1.255287066512	5.941764789783
H	0.718633957330	-0.515394772597	6.146610315589
H	5.121810480665	2.393742027581	7.075392432907
H	-0.668102645541	0.380839624235	6.153757712428

1a-sigma-5.xyz

38

-826.478780312

C	0.042442891789	0.276257262416	0.081579743170
C	0.002080087394	0.100727206181	1.556632081849
C	1.219136425977	0.054265185679	2.289365124637
C	2.441173009362	0.165744646541	1.582565393109
C	2.520289057113	0.327689399768	0.192819690040
C	1.361932870954	0.382544759230	-0.542081691983
C	-1.214116639005	-0.023891787296	2.230658110327
C	-1.226104005697	-0.197126413549	3.603712555118
C	-0.019057668596	-0.242455674104	4.315169857549
C	1.217151796301	-0.115472757109	3.710511516378
C	2.486103437824	-0.166542322172	4.493588884989
C	3.044752788118	-1.396336931093	4.797970621247
C	4.250019274245	-1.481710436425	5.530776907459
C	4.884303346213	-0.335572085458	5.949316483861
C	4.344184101823	0.944949644288	5.659636479164
C	3.121906338660	1.044882957854	4.918970889189
C	4.990104426187	2.134317050830	6.088709415705
C	4.455250979647	3.370450622971	5.803764680758
C	3.247146143083	3.468773403767	5.074919304792
C	2.595034345796	2.334876602276	4.642051255136
N	-0.087516989987	-0.424382778510	5.771817175313
H	-0.515590401415	-0.535814558230	-0.419661969569
H	1.398597175678	0.508507088533	-1.620897203970
H	3.491053454291	0.405859213259	-0.284130330770
H	3.367023203351	0.117261191057	2.150208869467
H	-2.171832099243	-0.297561008632	4.128736292511
H	-2.151001839988	0.010111084789	1.683534654693
H	1.665895732484	2.431689505001	4.086601449931
H	2.828854058425	4.447615083537	4.855556656589
H	4.958270206633	4.273802370515	6.138312721693
H	5.918021626591	2.050007752873	6.649761954916
H	5.813223327808	-0.395835757493	6.511637640096

```
 H         4.669284201481     -2.458482550538      5.754077963015
 H         2.556456016637     -2.307589202932      4.461143561271
 H        -0.608907738834     -1.270093969963      6.033341263585
 H        -0.539925992461      0.369937573556      6.240724674764
 H        -0.548003160751      1.164572070489     -0.212358770655
 H         0.856833764759     -0.517173809842      6.170420056510
```

1a-sigma-6’.xyz

```
38
-826.476906922
 C         0.000769087414      0.050844006878     -0.004190781635
 C        -0.054107295148     -0.002404031201      1.411832520835
 C         1.163812976545     -0.034931633910      2.164178400979
 C         2.418520877857     -0.005436723698      1.471564452688
 C         2.393980460705      0.041690386877      0.093192148064
 C         1.205049330169      0.069600680897     -0.661405679954
 C         1.086429808295     -0.099100358181      3.580478758821
 C        -0.135015776007     -0.123672250475      4.217512119648
 C        -1.336654806705     -0.086501236681      3.473652979772
 C        -1.295290786363     -0.028380694502      2.099040981891
 C         3.698887893871     -0.050454883579      2.242493816444
 C         4.262479067855      1.120929689819      2.804513108016
 C         5.485474451279      0.989611029348      3.569008657161
 C         6.099679519666     -0.288900902396      3.745199854222
 C         5.527781799919     -1.404275536144      3.191146131324
 C         4.331609268183     -1.276015779384      2.451403041027
 C         6.049065726830      2.118565161317      4.132518613950
 C         5.454617840031      3.441691684691      3.985199287368
 C         4.215712759187      3.520414668124      3.192185182137
 C         3.661112595611      2.414539824932      2.639010063980
 N         3.655752262488      0.061635464020     -0.669927639375
 H         3.793988478032     -0.804767954716     -1.203989889673
 H         4.475744754981      0.172194752922     -0.063533208603
 H         3.678591113163      0.835178961172     -1.344407937256
 H         6.224142970070      4.132546602772      3.588755257688
 H         5.301723235116      3.869451386454      4.995914494214
 H         2.747990546852      2.494157316115      2.057892409988
 H         3.755554697118      4.495452167808      3.064026001027
 H         6.965663525190      2.032328468189      4.711542470062
 H         7.016675512796     -0.354831934253      4.323422035034
 H         5.976794008463     -2.383821976039      3.317134594407
 H         3.881078236946     -2.171091367342      2.029271833199
 H        -2.214292153606     -0.003233992268      1.518526891824
 H        -2.292116881928     -0.106840814931      3.990751382056
 H        -0.175006411955     -0.173785779760      5.302276266487
 H         1.998059910364     -0.134687078220      4.169100135806
 H         1.247771233573      0.108084609014     -1.745944034042
 H        -0.925556252212      0.076062841793     -0.571451365208
```

1a-sigma-6.xyz

```
38
-826.470060227
 C        -2.592935527703     -0.526088850773      0.908535706380
 C        -3.855382620980     -0.661553606108      1.545124559238
```

C	-4.168296120070	-1.801358589854	2.253392280371
C	-3.238326577922	-2.861622610699	2.357847836038
C	-2.007484244510	-2.761539614130	1.749080228864
C	-1.651285999374	-1.600788871307	1.012974343650
C	-0.385062477195	-1.485315619315	0.381140839392
C	-0.049651938673	-0.356738189366	-0.329543829177
C	-0.968945038756	0.711133288217	-0.436815327811
C	-2.214185072948	0.638411592101	0.164058981075
C	-3.156661477268	1.789594607467	0.038305792596
C	-4.191418811434	3.870926236220	0.869539466558
C	-4.293141698378	4.840000975310	1.853431453744
C	-3.482861784342	4.800487167030	3.061360597661
C	-2.544844597865	3.675274856225	3.192031633580
C	-2.447477903385	2.722944006426	2.229914775963
C	-3.257988238637	2.783459531389	1.050171418917
C	-3.962594077214	1.943688133949	-1.080206072777
C	-4.880996632600	2.993776703500	-1.278716586538
C	-4.996146379024	3.949693746226	-0.306458705938
N	-3.862816682767	0.940257769839	-2.148244072152
H	-4.156117175511	4.860832590199	3.939962242839
H	-2.952815222061	5.769621512543	3.159080973870
H	-4.585811271866	0.139831983851	1.473025606643
H	-5.138923855799	-1.887066195564	2.734832131719
H	-3.499681076288	-3.754782205845	2.919076231992
H	-1.287556597076	-3.573324349609	1.823521033140
H	0.319711212780	-2.309080459183	0.467930935665
H	0.921197268767	-0.274218133920	-0.809483972893
H	-0.688521293621	1.604554282367	-0.989669475024
H	-4.993732375886	5.662859651292	1.732603715699
H	-1.927349064887	3.628673224097	4.083746284379
H	-1.747869555822	1.901296660505	2.338861155770
H	-5.482452264157	3.038298961549	-2.180700448002
H	-5.693884326746	4.773107048523	-0.421123339722
H	-3.581508463550	1.361877700231	-3.042926893501
H	-4.755113289773	0.455083376427	-2.307199473097
H	-3.158547767463	0.226287170187	-1.912608989113

1a-sigma-7’.xyz

38

-826.476761971

C	-0.004914774984	0.031963964776	0.008945478814
C	-0.060584014869	-0.025407416310	1.424578233756
C	1.156511517669	-0.047644165252	2.178444919104
C	2.410175007271	-0.002969302969	1.484900900751
C	2.388124368378	0.032452606424	0.105752189649
C	1.199475993035	0.056072784317	-0.648552914572
C	1.079607457862	-0.127501557041	3.594113384589
C	-0.141886966701	-0.173342741611	4.229263022162
C	-1.343369089154	-0.141475768068	3.484594412284
C	-1.301926665716	-0.070522416849	2.110775712274
C	3.690578109095	-0.021612442338	2.250582244514
C	4.210279591462	1.187767451950	2.852320272078
C	5.437211784435	1.142451574563	3.620328588983

C	6.108397476411	-0.086513687206	3.752382147498
C	5.596881858611	-1.222060504596	3.157628565156
C	4.390210825908	-1.194622110557	2.411807199012
C	5.957574484790	2.331542101986	4.229531000273
C	5.322586833140	3.521244406659	4.094878910035
C	4.079289370532	3.629865641932	3.313950106374
C	3.570438230452	2.403311568873	2.709259540231
N	3.651489813996	0.067681868778	-0.653619385716
H	3.676069075144	-0.660150619263	-1.377166085891
H	4.469147226198	-0.087807422633	-0.052682377269
H	3.791552005409	0.968010518933	-1.127731975168
H	3.269931231477	4.078387052976	3.923520183273
H	4.180007507797	4.395869797394	2.519798903875
H	2.654090587561	2.475832850382	2.128836108038
H	5.715601145405	4.421979418721	4.556457810380
H	6.877140039237	2.252510318617	4.801991475973
H	7.030412840445	-0.134103300656	4.323696467351
H	6.121778646908	-2.167023496335	3.264602589278
H	4.013648466878	-2.117020038125	1.979018393135
H	-2.220636571808	-0.051944014315	1.529655627621
H	-2.298756584228	-0.177660310554	4.000850538955
H	-0.182228210632	-0.237480310517	5.313228460017
H	1.990858950402	-0.161818262636	4.183676040425
H	1.241823764651	0.094084879518	-1.733221679532
H	-0.931088671666	0.051607133019	-0.558674809339

1a-sigma-7.xyz

38

-826.471647950

C	-2.497002646019	-0.514517349676	0.886651500174
C	-3.717299904143	-0.718506310677	1.585058574257
C	-3.932705468956	-1.876239495119	2.300083218678
C	-2.943116349926	-2.885543399940	2.351176966217
C	-1.752352930211	-2.718593838298	1.680986437385
C	-1.496695996964	-1.538811071029	0.933458718429
C	-0.275484704471	-1.357364967339	0.232893204847
C	-0.039318939217	-0.213483596075	-0.493135387533
C	-1.017184665044	0.804710852320	-0.547825265149
C	-2.219613917936	0.667400158224	0.125310243565
C	-3.225230655512	1.763676514352	0.041590114111
C	-4.316914110620	3.821154026089	0.986328259470
C	-4.426522332677	4.806372259770	2.019918498197
C	-3.608564312401	4.774478265515	3.101085169888
C	-2.580454236416	3.731080325145	3.240569301166
C	-2.493270450408	2.740536811290	2.175153729467
C	-3.335518859910	2.763801845569	1.079067893643
C	-4.081863401926	1.882880874487	-1.025168565010
C	-5.040078977861	2.915932396479	-1.131984366114
C	-5.157803653117	3.865528392429	-0.142137971533
N	-4.017947814995	0.894996161528	-2.109428203352
H	-1.578049579591	4.183393724415	3.379283676039
H	-2.687133215535	3.195265252767	4.204972599749
H	-4.492113185820	0.043123052809	1.554394591556

```
 H        -4.871878468295     -2.015911095169      2.828815576161
 H        -3.127505811432     -3.792908011110      2.920042941197
 H        -0.987400851906     -3.490937917068      1.712689944288
 H         0.474617608433     -2.143563013862      0.277913243931
 H         0.897102868309     -0.080849771182     -1.027105468625
 H        -0.817195059434      1.709490059432     -1.116668363175
 H        -5.180556616682      5.580470982814      1.913755509556
 H        -3.689653533898      5.519280368649      3.886777229635
 H        -1.732468828133      1.969347095150      2.262473043579
 H        -5.692173131961      2.959475809417     -2.000200295382
 H        -5.899777400860      4.651856079803     -0.233765975237
 H        -3.298045817160      0.187088035045     -1.907746234593
 H        -3.774358114270      1.322527505807     -3.011786530216
 H        -4.907587923037      0.396909947239     -2.236770879268
```

1a-sigma-8’.xyz

```
38
-826.483234059
 C        -0.010509288768     -0.005867751681     -0.005651512245
 C        -0.060295825867     -0.034852386765      1.411234158220
 C         1.160094552011     -0.034945060371      2.160071664984
 C         2.413084270343      0.010556248924      1.463938977491
 C         2.382842723586      0.026240851526      0.084453677469
 C         1.191460899296      0.017655742060     -0.666833426741
 C         1.087033499930     -0.088853188483      3.577265937402
 C        -0.132018922427     -0.125985096073      4.218374711895
 C        -1.336142853284     -0.114971161840      3.477754098667
 C        -1.299029006466     -0.073377684048      2.102486991426
 C         3.697109978171      0.022451207421      2.230112081174
 C         4.197102901763      1.206699310113      2.798077040008
 C         5.412566602815      1.157447142014      3.540311023146
 C         6.108105404123     -0.065957659241      3.707321977646
 C         5.602775152541     -1.220943563011      3.149905752039
 C         4.404318376214     -1.172042925745      2.421366081622
 C         5.920300663177      2.345564331396      4.113474685375
 C         5.290900649482      3.593985763589      3.996002823237
 C         4.118191680177      3.678933123155      3.291300860913
 C         3.497042650041      2.513203965103      2.654478959076
 N         3.641971859729      0.048882456695     -0.683766887435
 H         3.781058120933     -0.819311343764     -1.214557756928
 H         4.463504013734      0.163567427104     -0.079705398815
 H         3.660944572433      0.819952017964     -1.361156654527
 H         3.332313759683      2.760207695668      1.590183673656
 H         2.458445609116      2.443024791773      3.027485632398
 H         3.606822057979      4.632070256256      3.184744133727
 H         5.732567067839      4.469064095518      4.459870987400
 H         6.849902449516      2.281967827705      4.675279524426
 H         7.032954824264     -0.079108850092      4.276629464803
 H         6.119663219688     -2.167214900287      3.272262389817
 H         4.005989730500     -2.089736277979      1.995517313096
 H        -2.219761110167     -0.072466980049      1.524181280608
 H        -2.289757242295     -0.144753383726      3.997774545483
 H        -0.167859912519     -0.167979133074      5.303628452873
```

H	2.001204361473	-0.111797664226	4.163399588520
H	1.230406329612	0.033259324405	-1.752112709256
H	-0.938769186876	-0.006568375421	-0.570306596735

1a-sigma-8.xyz

38

-826.478278576

C	-0.011513324019	0.094857841867	-0.017401827288
C	-0.020647908174	-0.009017568110	1.393436576502
C	1.196717604789	-0.062952891764	2.122496244247
C	2.426448009451	-0.018016900263	1.441252752623
C	2.372725205364	0.088570333277	0.051343740964
C	1.188909974078	0.145249763061	-0.690897735228
C	1.145771412870	-0.158777836036	3.604587980946
C	-0.178776014387	-0.188072475082	4.226096591515
C	-1.339036007907	-0.139660930537	3.491102721446
C	-1.251342480315	-0.051955378217	2.097440634254
C	3.727151340028	-0.053161348876	2.174925650614
C	4.302110174677	-1.301625766884	2.580419427796
C	5.536887943673	-1.276881401335	3.306943605852
C	6.153800610262	-0.031440299961	3.596511139731
C	5.583786076502	1.151483483106	3.188239173795
C	4.363021445649	1.139603858759	2.476289804157
C	6.119043040215	-2.503840219509	3.721400029547
C	5.513838199670	-3.706163494916	3.432623219523
C	4.297624908777	-3.731187741653	2.711229647230
C	3.706468705155	-2.558910533176	2.292888371467
N	3.641821044498	0.133243063622	-0.688844624530
H	3.702028364507	0.952480073665	-1.306160605959
H	3.773234564844	-0.700217404210	-1.275971484761
H	4.439357659427	0.180464843130	-0.038716446499
H	1.702571165935	-1.046522087297	3.960364152401
H	1.727942578378	0.659424843115	4.067929179544
H	2.776929947089	-2.599937220942	1.730571870872
H	3.826804485599	-4.683613373386	2.482239387251
H	5.968785353806	-4.638745679377	3.755628889496
H	7.055704780003	-2.475969384258	4.273458284671
H	7.090794120989	-0.027922969749	4.148648755864
H	6.060917501708	2.101226056816	3.412054908413
H	3.913617892149	2.081424178712	2.170004760082
H	-2.170079065529	-0.012477211160	1.516372275912
H	-2.310005619050	-0.166826449779	3.973051671167
H	-0.217183968457	-0.255085281229	5.310205815160
H	1.218124286540	0.227543974169	-1.772560649184
H	-0.949056520393	0.137846318727	-0.562584743692

3-Sigma-1’.xyz

39

-885.188205847

C	0.083800454348	0.087046403214	-0.057442088142
C	0.058400654528	0.492659231071	1.358880380148
C	1.282248322466	0.153426913591	2.152760180655

C	2.439065591865	-0.340800733256	1.475380625758
C	2.374807592268	-0.593051782607	0.090735031250
C	1.212040753070	-0.396853026693	-0.670491113546
C	3.631577720893	-0.604333875594	2.200811743276
C	3.671225554349	-0.387905147423	3.561173480120
C	2.525029226433	0.094774206923	4.221243028928
C	1.348476588424	0.365862605349	3.528960674396
C	-1.282487021584	0.278679801464	2.048496229443
C	-1.769453809718	-1.030560693449	2.327376523634
C	-3.040017914965	-1.177698155930	2.973780301525
C	-3.773475177595	-0.014193847596	3.314003738366
C	-3.293555996039	1.243377321231	3.037325471798
C	-2.037907046924	1.392799985631	2.397065566418
C	-1.048768202728	-2.209846178340	1.995442226708
C	-1.557024686998	-3.458697467062	2.279881747397
C	-2.812766305617	-3.599552174114	2.915800358757
C	-3.535733954943	-2.477837683212	3.254313511424
O	-1.505711910818	2.604477573202	2.092075564162
C	-2.218316680712	3.793709641961	2.425666453590
H	-4.502915571603	-2.571315089700	3.743704875520
H	-3.201163120885	-4.590712711780	3.134412517400
H	-0.985528327032	-4.344462023607	2.014177626457
H	-0.078900085458	-2.134354913781	1.510685167208
H	-3.878008376445	2.115135210589	3.309338209422
H	-4.738214512591	-0.124317051510	3.803915832317
H	0.477115334080	0.734780751786	4.061927339097
H	2.557311223407	0.257183907030	5.295184129676
H	4.577181201977	-0.588381836178	4.124640326898
H	4.502537029711	-0.976340159836	1.668369388566
H	3.266804447392	-0.970630490747	-0.405043152713
H	1.211350088097	-0.633008378674	-1.729282257470
H	-0.824792203822	0.252449766347	-0.631367164114
H	-1.589438650397	4.614926896090	2.080953725580
H	-3.183603492379	3.830454644688	1.909540830543
H	-2.364227148897	3.873643501203	3.508199281065
H	0.115189464836	1.605533009718	1.245745288483

3-Sigma-1.xyz

39
-885.207593173

C	0.842470066043	0.263356752613	1.993735160658
C	-0.129300717407	0.137373687447	1.019922306829
C	0.242801170522	0.027767246508	-0.356519128600
C	1.641748629967	0.065889040049	-0.684022960348
C	2.604802113009	0.195837175220	0.349132665936
C	2.214133001092	0.289831885495	1.664275822729
C	2.043256694681	-0.029781233191	-2.043533058788
C	1.115598691466	-0.160657983329	-3.050699071234
C	-0.261953760009	-0.202879387548	-2.734126266045
C	-0.684296081036	-0.113148719875	-1.426218746896
C	-1.592733501138	0.112738931703	1.470260855269
C	-2.002727893897	1.336209292350	2.257053548027
C	-2.748831148303	1.201428950598	3.451826077708

C	-3.060899112068	-0.102941628474	3.933717494120
C	-2.681425591439	-1.270828478877	3.304913003309
C	-1.945603429159	-1.195952275883	2.116702308562
C	-1.695334822100	2.613467480380	1.794637995866
C	-2.117726887213	3.737938363081	2.504340212787
C	-2.852440240694	3.609827905709	3.691070439521
C	-3.166764878362	2.347357583031	4.164085847599
O	-1.534617715134	-2.228757592426	1.443828350427
C	-1.828935692455	-3.575668190717	1.881595376779
H	-3.735865350441	2.223984244384	5.081711322039
H	-3.171859479922	4.494432994418	4.233650951606
H	-1.869190953095	4.726894786250	2.128888528039
H	-1.121121576286	2.736770984284	0.880608696978
H	-2.945598156927	-2.227072283955	3.739809421152
H	-3.630793031856	-0.180692665857	4.857247666568
H	-1.751585687877	-0.157623490326	-1.229007347722
H	-0.995371324339	-0.309429876519	-3.529231284473
H	1.435980774358	-0.233227124300	-4.086731897453
H	3.106145176475	0.001938273593	-2.273288626470
H	3.659009677797	0.220262748676	0.082538962614
H	2.952691342746	0.388532090488	2.455163418451
H	0.550521653612	0.351097829306	3.038199671416
H	-1.374761669487	-4.216591561900	1.129069494449
H	-1.374763146125	-3.752948337068	2.858480842337
H	-2.909905396913	-3.726764595568	1.911030502258
H	-2.234832208083	0.123506130231	0.574659523996

3-Sigma-2’.xyz

39

-885.191105741

C	1.596219279378	-0.160838414077	0.037123917602
C	2.387065089248	0.874608727607	0.561631229387
C	1.821735457019	1.862195768101	1.351417588382
C	0.442991533052	1.852278992838	1.665819706752
C	-0.361260528207	0.857386029273	1.169300771053
C	0.178995921853	-0.161748063462	0.325239547724
C	-0.625470783259	-1.189330565334	-0.179150399746
C	-0.030213645758	-2.225373825525	-1.051661060797
C	1.432501503625	-2.212879978797	-1.241129149322
C	2.189694113752	-1.210552538421	-0.743533258595
C	-2.077554031998	-1.269075642019	0.056627599052
C	-2.632988222097	-2.324763019010	0.850405994635
C	-1.831341535592	-3.238438200183	1.587434153736
C	-2.409016875885	-4.231937552580	2.349095363502
C	-3.814772373055	-4.356409697757	2.432140327301
C	-4.618620655312	-3.465040678594	1.757428723243
C	-4.054764902244	-2.432314099068	0.964065794906
C	-4.869702654053	-1.486764656270	0.292929728446
C	-4.327518267305	-0.467582014575	-0.452202544748
C	-2.921586684519	-0.348741057282	-0.573906942393
O	-2.323810218261	0.591894793339	-1.337334926907
C	-3.124737632227	1.560854799258	-2.013802237318
H	-0.420489917746	-3.226435805249	-0.819294655967

H	-0.493359070983	-2.036264855746	-2.044516130267
H	3.451720464671	0.890237279793	0.347084861520
H	2.452291832033	2.652591804390	1.749551559457
H	0.029309233026	2.626555225538	2.304264987917
H	-1.417374975820	0.827771482718	1.415873174787
H	1.872483179144	-3.017197843901	-1.823313034325
H	3.263104590302	-1.184674485646	-0.908489197458
H	-0.748594182907	-3.147046295535	1.573904856124
H	-1.774021768458	-4.920132626647	2.901198781553
H	-4.254706271594	-5.143707013334	3.038293973258
H	-5.701893236292	-3.532933508842	1.829196174342
H	-5.950623723350	-1.572258616941	0.375379173019
H	-4.979917933552	0.236397890048	-0.955876341140
H	-3.786605357768	1.082231792827	-2.743171149554
H	-3.709264781746	2.154008500912	-1.302854490676
H	-2.416856478311	2.207002980119	-2.533183152351

3-Sigma-3’.xyz

39

-885.188969712

C	1.527018765086	-0.141458420577	0.356001271099
C	2.026854071724	0.824122053682	1.288786766633
C	1.160728460931	1.647512872820	1.959575515074
C	-0.229609585963	1.528863639004	1.723330202696
C	-0.747115740435	0.609528753075	0.822800365116
C	0.102743745857	-0.246456848054	0.107223709664
C	-0.408021476515	-1.211004446828	-0.847447481981
C	0.463308421304	-2.036805834148	-1.484924329494
C	1.918304714839	-1.984994208663	-1.254966453347
C	2.393408350941	-0.985935769707	-0.304695127735
C	-1.869674859956	-1.287604229504	-1.131458339992
C	-2.679984975691	-2.311287603241	-0.557081185069
C	-2.163598878439	-3.287524989388	0.341328465301
C	-2.979094015027	-4.261697038916	0.874548851262
C	-4.354535941551	-4.316028870105	0.544822553043
C	-4.887328202662	-3.382128475416	-0.314806199043
C	-4.075147765493	-2.363940135752	-0.880519858687
C	-4.609562422532	-1.390955380433	-1.760032797977
C	-3.820609792657	-0.404536460343	-2.303576601129
C	-2.440117796717	-0.349541908758	-1.988108698586
O	-1.602240674300	0.592629343878	-2.492085322366
C	-2.129922439964	1.603251308590	-3.346242678751
H	2.458225721027	-1.843610621381	-2.211493992868
H	2.303192448588	-2.975997080802	-0.944523981509
H	3.099327574679	0.885398433981	1.450831879505
H	1.528496242487	2.381840467573	2.668932237283
H	-0.913208668988	2.178308359494	2.263728255508
H	-1.819342452519	0.550438625047	0.667700217239
H	0.097298478073	-2.766805786269	-2.201698561474
H	3.463565350288	-0.921297076759	-0.121449851861
H	-1.111246191043	-3.263452435582	0.609719551093
H	-2.561266905642	-4.996580309982	1.558446753907
H	-4.984372834349	-5.091710330695	0.972592749217

```
 H        -5.943697045949     -3.409189994710     -0.574352579496
 H        -5.667970211141     -1.428782018796     -2.008039408608
 H        -4.259978301713      0.324682432253     -2.975083303564
 H        -2.546146338605      1.170021346281     -4.262630733399
 H        -2.893510366655      2.197399597781     -2.831775913944
 H        -1.283078708935      2.241874296535     -3.600074425730
```

3-Sigma-3.xyz

```
39
-885.197129842
 C         3.032215993523     -1.327821556621      0.418912344008
 C         3.209672084381     -2.382191204782      1.354631431674
 C         4.143773143078     -2.285005718347      2.361451755740
 C         4.943743261415     -1.123200192960      2.475285677571
 C         4.796596791160     -0.083189171248      1.583691739571
 C         3.842691278785     -0.151368362000      0.532663997537
 C         3.656808462438      0.905551945386     -0.413186513437
 C         2.708698181525      0.780502504535     -1.410142771280
 C         1.911908263957     -0.382531587445     -1.517955084404
 C         2.071085354056     -1.414771265169     -0.622451338323
 C         4.483468773437      2.146735097745     -0.338927543516
 C         4.052236097121      3.273109369829      0.415561400824
 C         2.839171061684      3.250447815232      1.148041143947
 C         2.433964441339      4.347805982159      1.874306442098
 C         3.200424795928      5.548451274678      1.926297629828
 C         4.376590188697      5.621545390080      1.240837737271
 C         4.841996002866      4.497854549661      0.470377967806
 C         6.022942184306      4.562048375858     -0.215309819700
 C         6.530544824499      3.436312878283     -1.008629685601
 C         5.683049573351      2.218050623174     -1.030539029690
 O         6.057776453117      1.154807029901     -1.721842807364
 C         7.283477381063      1.153429716692     -2.467921685049
 H         6.718802497479      3.794009167097     -2.036118737877
 H         7.544159779302      3.191170631964     -0.643768494474
 H         2.590510999278     -3.271955830326      1.261059573798
 H         4.269424235326     -3.098848778123      3.071065088510
 H         5.679068642411     -1.051929931705      3.272847970902
 H         5.418045754461      0.803193930197      1.682453016931
 H         2.574320671433      1.589228781080     -2.124683211530
 H         1.174570760685     -0.453030975814     -2.313307223226
 H         1.460881872039     -2.312117033179     -0.700148019581
 H         2.226062272283      2.355837038928      1.130817835612
 H         1.499114633819      4.298700225365      2.426542445700
 H         2.841991584684      6.390346087998      2.510107069827
 H         4.985961988920      6.520823654954      1.259807043635
 H         6.630127385746      5.463190395704     -0.189971104345
 H         7.333313160585      0.168414265108     -2.928934628293
 H         7.263846001383      1.925295569579     -3.242785239133
 H         8.140675442236      1.295856530131     -1.803700175279
```

3-Sigma-4’.xyz

```
39
```

-885.199277705

C	-0.031165153843	0.753563524971	0.164983976726
C	0.015111226011	0.290797331846	1.512483677397
C	1.265325668513	-0.116520432528	2.090632735542
C	2.430032004982	-0.183287077762	1.275747566530
C	2.354229754737	0.152034919763	-0.174142593400
C	1.058007675063	0.658843711425	-0.646542971570
C	3.646619220941	-0.553817185074	1.852189519432
C	3.727683631544	-0.831983503940	3.211559985965
C	2.590917162777	-0.730017049127	4.031912604924
C	1.377492889896	-0.374857857276	3.479952545809
C	-1.244857668217	0.224201162769	2.248848755148
C	-2.095828327649	1.380394906410	2.360072286159
C	-3.389538249337	1.241303130040	2.950981131099
C	-3.796940714195	-0.034292789362	3.418538457381
C	-2.979296514015	-1.133114106137	3.325699536378
C	-1.695419508602	-1.017821179275	2.740544130697
C	-1.679022797351	2.681231482288	1.977159989547
C	-2.522397029186	3.766649176034	2.112366660864
C	-3.813549578276	3.616944849598	2.660976787920
C	-4.231436362150	2.373349728904	3.083637902918
O	-0.895944055356	-2.079372771328	2.542111975955
C	-1.304659339047	-3.373784890854	2.988174140228
H	-5.212065176240	2.243315066976	3.535699318947
H	-4.464248763293	4.480773114419	2.765046831330
H	-2.178588234146	4.750641397345	1.804108356845
H	-0.677186030041	2.839763831261	1.589487645562
H	-3.328783682233	-2.093821107682	3.685113326876
H	-4.783978304754	-0.139882928395	3.862474466100
H	0.501906972295	-0.268918679896	4.111872179689
H	2.670690453006	-0.919426097477	5.098239435951
H	4.683257590778	-1.112368632719	3.646334657646
H	4.536915638766	-0.612110063736	1.231272475983
H	2.622735058764	-0.727135850003	-0.787757719398
H	0.974965710217	0.925986740816	-1.696930847997
H	-0.981568496833	1.090698878844	-0.235711730655
H	-0.481606958271	-4.038480419960	2.726183706746
H	-1.456357739218	-3.386445368780	4.072086410310
H	-2.214855011206	-3.694856932985	2.471495272628
H	3.141268435169	0.878293660587	-0.439886058209

3-Sigma-4.xyz

39

-885.196064555

C	3.100524044484	-1.341729573834	0.264143048765
C	3.427542392294	-2.567910231923	0.900551976035
C	4.534639827370	-2.666772844634	1.713675889150
C	5.353843060889	-1.535619361328	1.930757668366
C	5.063785579773	-0.331967571694	1.323655830952
C	3.946729439525	-0.204863277053	0.460541934027
C	3.584778631890	1.030609206974	-0.172881337052
C	2.408039005617	1.115478228451	-0.910854220821
C	1.596951559141	-0.017415476569	-1.116248361986

C	1.939568811836	-1.222068745029	-0.544250487135
C	4.441568599977	2.224096467917	-0.135412951408
C	3.949478980982	3.450021322281	0.410065458475
C	2.709062714297	3.496317437456	1.105470071574
C	2.243151431987	4.685187902406	1.627126727674
C	3.002143084717	5.859471159768	1.485546380535
C	4.233649367486	5.832084573898	0.841184823263
C	4.725177317303	4.641269544540	0.306056166557
C	6.050587528196	4.602025922110	-0.360041478691
C	6.503117509248	3.294557773691	-0.849998197109
C	5.749385033146	2.157176000826	-0.727979719264
O	6.102146994050	0.949171406955	-1.207675792817
C	7.381185217746	0.811917224988	-1.825285715206
H	6.820193894280	5.013922238913	0.316112819650
H	6.071227635961	5.310594510063	-1.207196266036
H	2.778540316719	-3.426304209064	0.743264377386
H	4.774760555432	-3.609052032414	2.198932359153
H	6.217274010717	-1.613723903960	2.586263899104
H	5.693527014921	0.531224578662	1.520611777977
H	2.139598881454	2.055455186742	-1.385048182276
H	0.704803044559	0.069960036108	-1.729515964905
H	1.314957428221	-2.099386192788	-0.695790462603
H	2.138637584507	2.585025690839	1.244676847417
H	1.297662110245	4.710216312030	2.160439977988
H	2.632825510553	6.794038253702	1.898754848288
H	4.823861332815	6.740565874136	0.754171484037
H	7.460673253609	3.271123472019	-1.359548392347
H	7.456199738447	-0.238363214488	-2.105958020693
H	7.451688885821	1.438270635206	-2.721528037807
H	8.182524322466	1.064667430042	-1.122175262352

3-Sigma-5’.xyz

39

-885.195179513

C	-0.068616071189	0.288748731897	0.010895117674
C	-0.049139004245	0.120316683423	1.386109369072
C	1.222422932385	-0.043377197884	2.026285924308
C	2.426285214473	-0.061420921969	1.257561314171
C	2.358900843157	0.101818645364	-0.123335612731
C	1.121922974986	0.279344397463	-0.734564586889
C	3.732418658307	-0.249709400556	1.948342116357
C	3.702833671204	-0.386178002456	3.408997168952
C	2.532319490809	-0.338073249639	4.116163990008
C	1.317943087450	-0.164795775961	3.425795670948
C	-1.322627425277	0.100037631595	2.155802851311
C	-2.050467328570	1.303427563993	2.401343408760
C	-3.305832139809	1.235360972161	3.088540157902
C	-3.785852573962	-0.028863155713	3.510026046002
C	-3.074113663145	-1.181481035513	3.275337796465
C	-1.833199525505	-1.120723108507	2.593254285387
C	-1.571963999587	2.584159420064	2.006025965909
C	-2.303155587308	3.723819506394	2.261366790660
C	-3.548698369560	3.651097742834	2.929370486652

C	-4.034469835381	2.428734259178	3.335644417214
O	-1.088130536072	-2.220437045011	2.313405716932
C	-1.560888478363	-3.501852225342	2.719871756485
H	-4.986787526073	2.358295499003	3.857202505352
H	-4.113960862699	4.558964085216	3.123147612361
H	-1.916458710838	4.690842964457	1.948979917314
H	-0.615183028057	2.664413275277	1.497963242122
H	-3.470024267957	-2.133915146721	3.609606281365
H	-4.738792641262	-0.084371059964	4.031416299916
H	0.398001990149	-0.117454576992	4.003932332857
H	2.525720026861	-0.428661170452	5.197115305426
H	4.652214397468	-0.523705003167	3.920103494006
H	4.422847532733	0.574585401868	1.694511891872
H	3.267525968395	0.090955796664	-0.719030825756
H	1.072005896427	0.405844540272	-1.812698482278
H	-1.018934485692	0.416425449971	-0.499298356432
H	-0.803442923709	-4.210920712732	2.384046334996
H	-1.658429714216	-3.561758779319	3.809534414496
H	-2.518669572644	-3.739074536557	2.243517244087
H	4.261429102960	-1.126245892559	1.532113891391

3-Sigma-5.xyz

39

-885.194946942

C	-0.261493036932	-0.048391167502	-0.260081179670
C	-0.268671189100	-0.126550419805	1.120927794866
C	0.968956919971	-0.197569794934	1.836784260985
C	2.194324107652	-0.203341922655	1.094773660093
C	2.153317787988	-0.134434185955	-0.323235830254
C	0.951336069256	-0.054993664723	-0.986683740844
C	3.427060510541	-0.270688830092	1.797413476398
C	3.455232736290	-0.324150896560	3.173091264508
C	2.245732074044	-0.308293684824	3.907111690608
C	1.033589500264	-0.245080308663	3.254961552615
C	-1.572486412056	-0.146983134555	1.839688017774
C	-2.344686517005	1.039216708383	2.014995844326
C	-3.623279334323	1.005164853108	2.651756546133
C	-4.101292082629	-0.212962305792	3.120435135838
C	-3.350507929322	-1.371488149168	2.963971523447
C	-2.090185403335	-1.350378244961	2.329755050888
C	-1.850747331615	2.291706927489	1.588712382871
C	-2.560238737484	3.499160999198	1.718434866531
C	-3.800348812307	3.493037575807	2.299800509221
C	-4.408822250509	2.259209332980	2.804780220575
O	-1.336455618157	-2.452482156070	2.141929634262
C	-1.810598839484	-3.712183592205	2.616667725235
H	-5.402328352188	2.151759094289	2.331765322700
H	-4.366355226614	4.414709739410	2.406303356285
H	-2.114170659057	4.420802085242	1.359979661837
H	-0.863055723013	2.324590925876	1.136078013352
H	-3.756347856613	-2.306987140751	3.333290054895
H	-5.069640092134	-0.267981622550	3.610170598511
H	0.111779658299	-0.227760627100	3.830484062545

H	2.274377463158	-0.344328334228	4.993264168226
H	4.405268831882	-0.374913507741	3.698950056877
H	4.353284105340	-0.276234203338	1.226621765589
H	3.089854714276	-0.141588566483	-0.876620442127
H	0.923541839935	0.000736686825	-2.071659953100
H	-1.205282366107	0.004198587085	-0.797855450435
H	-1.033938876072	-4.430418138938	2.353646000588
H	-1.943273972929	-3.699445487522	3.703841450110
H	-2.747905398138	-3.991443380267	2.123296747183
H	-4.678544301773	2.419779951688	3.864454180555

3-Sigma-6'.xyz

39

-885.188969834

C	-2.793449178073	2.228215660571	-2.073378339428
C	-3.760009296363	3.181899593430	-2.318217439294
C	-4.127823875090	3.594043053029	-3.670120899206
C	-3.405790510745	2.933785939438	-4.774032117474
C	-2.457782640015	1.996735812799	-4.534004420077
C	-2.115491874999	1.606481588868	-3.194226637998
C	-1.128622025778	0.630029627304	-2.931243685758
C	-0.845642760396	0.307800149724	-1.605505041242
C	-1.500758365726	0.910376892336	-0.506666494420
C	-2.464135247585	1.857701144293	-0.731456316235
C	-0.397242770225	-0.060244649675	-4.032149251545
C	0.840342562167	0.450414783856	-4.521899034877
C	1.430452329422	1.642590374265	-4.015599626898
C	2.627484797514	2.107453782882	-4.515477462358
C	3.300925537959	1.410812266937	-5.547306935726
C	2.756175055555	0.254630490992	-6.059431442269
C	1.524175397742	-0.251337778118	-5.567063621013
C	0.946944757674	-1.437740579087	-6.082177438245
C	-0.246757179596	-1.922842302994	-5.601386104440
C	-0.926954592789	-1.230786793169	-4.567700471453
O	-2.109564758518	-1.649688876154	-4.048078806231
C	-2.705696968652	-2.840247184606	-4.554001446119
H	-4.030935919295	4.694853677043	-3.739084198422
H	-5.222228457159	3.473933322711	-3.787733416889
H	-4.276151876034	3.653712642235	-1.484900974066
H	-3.658161977080	3.223469394989	-5.789829988063
H	-1.933840578701	1.518775454943	-5.355744154328
H	-0.084786085857	-0.443951218302	-1.409944393481
H	-1.234043366323	0.615484882742	0.503263508931
H	-2.986607461093	2.338595086681	0.090483768852
H	0.931140127553	2.192525194703	-3.222435423553
H	3.059679544498	3.020425895411	-4.112964386826
H	4.244310274818	1.790427065754	-5.931137730865
H	3.263472014777	-0.290808100419	-6.852664910855
H	1.462378708424	-1.973724790380	-6.876025358446
H	-0.661118803206	-2.834166926469	-6.018235000372
H	-2.057353632442	-3.708709194372	-4.392199912180
H	-3.628823318384	-2.967276525709	-3.987235994644
H	-2.943406200600	-2.743284559646	-5.619292146756

3-Sigma-6.xyz

```
39
-885.201036107
 C        -1.694387420298     -1.704881059486      0.701767062626
 C        -2.046846943270     -2.824312158849      1.502538972515
 C        -3.248136142740     -2.864164650788      2.174176978269
 C        -4.153392173473     -1.781433546824      2.070557149637
 C        -3.841729783877     -0.681287337279      1.301792892333
 C        -2.608624435425     -0.605952941574      0.599556981544
 C        -2.244656837521      0.518397894353     -0.206486099833
 C        -1.034943216276      0.526447219071     -0.873440959099
 C        -0.137989010349     -0.561843439541     -0.771659186725
 C        -0.459975494388     -1.652816937046      0.002047732064
 C        -3.154084633127      1.696336825625     -0.326074005777
 C        -4.072582451088      1.821681375385     -1.366780139437
 C        -4.223974663813      0.801090183988     -2.378638663261
 C        -5.114851888979      0.932160754990     -3.385003625616
 C        -5.987225911175      2.122037587249     -3.509235499998
 C        -5.817337818413      3.139707845594     -2.465633008139
 C        -4.911723353721      3.006984354375     -1.444918710643
 C        -4.783149520094      4.027906395481     -0.448427842067
 C        -3.886471654513      3.901021487700      0.569213631846
 C        -3.066461645203      2.735791718166      0.637827273797
 O        -2.177433291681      2.549748466907      1.596660828057
 C        -1.992409662936      3.527717414518      2.633340916504
 H        -5.856121418331      2.602347323394     -4.496521022819
 H        -7.051231723834      1.823237613519     -3.542963641030
 H        -1.347391199806     -3.654470138974      1.575514658934
 H        -3.505374937939     -3.726564165012      2.783918321518
 H        -5.101577142138     -1.819604309562      2.601008774492
 H        -4.548025744063      0.141742881500      1.228197703720
 H        -0.766333162107      1.385550454787     -1.483638402059
 H         0.806840089316     -0.527301240108     -1.307635644397
 H         0.227496885010     -2.491747395580      0.086580135106
 H        -3.597249399565     -0.082352380883     -2.312058294074
 H        -5.214900361115      0.154275903821     -4.136785115172
 H        -6.442192492052      4.028743633291     -2.517155772789
 H        -5.414228090438      4.909484951377     -0.516921579120
 H        -3.797497322626      4.678665719287      1.318299318800
 H        -1.210474100029      3.117366931637      3.270260002007
 H        -1.664895089554      4.479003483740      2.205951377930
 H        -2.913896275789      3.654048084305      3.207535680806
```

3-Sigma-7’.xyz

```
39
-885.190026674
 C         3.004106363058      1.012946246640     -3.423927103779
 C         4.122528941874      0.745757713527     -4.286706486007
 C         3.969094305416      0.663105338106     -5.628630618756
 C         2.642922198044      0.836082359950     -6.252230633271
 C         1.528965660646      1.123346458658     -5.350389361728
```

C	1.686305551805	1.237428478367	-3.985929588856
C	0.562104991590	1.528605395950	-3.123486042550
C	0.772929017114	1.534009718081	-1.760235161320
C	2.057814771815	1.300773191785	-1.224070530616
C	3.156791688726	1.054151662282	-2.032743783584
C	-0.781850663873	1.825018102163	-3.680025471593
C	-1.860575012476	0.899982309720	-3.529650055018
C	-1.699938378877	-0.379546082104	-2.928256726992
C	-2.766013058816	-1.244310901467	-2.806159403240
C	-4.048854582474	-0.885309007004	-3.282564294531
C	-4.234673192341	0.339528698067	-3.883054459749
C	-3.157555251290	1.253401959707	-4.024575822042
C	-3.330668780153	2.511034393745	-4.653330124834
C	-2.285437549494	3.391957577248	-4.797916413355
C	-1.000193997487	3.051073744111	-4.309015923129
O	0.066415695710	3.884635152839	-4.391894605153
C	-0.086042162034	5.149140231307	-5.031919699270
H	2.671492182464	1.629766428250	-7.024292862967
H	2.379562263370	-0.044479529179	-6.869209270306
H	5.097804782627	0.605199842669	-3.828958230061
H	4.815297468924	0.461179407675	-6.278476766595
H	0.541835923584	1.243541771647	-5.789984206638
H	-0.053865510520	1.743755352415	-1.088327235375
H	2.186903675695	1.328987187791	-0.145393146398
H	4.135691623157	0.889801591275	-1.592423113947
H	-0.722386237502	-0.681752229689	-2.563734857392
H	-2.617395705577	-2.216301446394	-2.342135026286
H	-4.879609082167	-1.578300574031	-3.178684180236
H	-5.213051057865	0.625915937185	-4.263224902705
H	-4.315106646174	2.781435001991	-5.028490906446
H	-2.452166519422	4.348518077426	-5.280282651453
H	-0.816846356461	5.772941773217	-4.505549619249
H	0.896138769389	5.620142611068	-4.981375498862
H	-0.379698768810	5.029005121907	-6.080528188641

3-Sigma-7.xyz

39

-885.183417608

C	0.241811181971	0.184623565498	0.781403494559
C	1.574270948009	0.471087378560	0.382397727160
C	1.873707408144	1.625250692083	-0.306169463380
C	0.846240467115	2.541120615642	-0.631023546913
C	-0.456750492978	2.293422959118	-0.256666831759
C	-0.798149760647	1.118819505704	0.465137954467
C	-2.137470362673	0.819648381729	0.878166090080
C	-2.406985439305	-0.370168184891	1.533592077050
C	-1.376856075375	-1.285919826495	1.840756607336
C	-0.078656233240	-1.010192729798	1.478456981238
C	-3.246669714024	1.770435527305	0.605073863437
C	-4.261904516133	1.417020080045	-0.367638443805
C	-4.177013778270	0.245731705490	-1.096021866225
C	-5.188115119412	-0.158299081091	-2.071955882927
C	-6.326111742504	0.756144115186	-2.270134582870

C	-6.388957302615	1.929219914893	-1.597440881641
C	-5.367493219463	2.306226710805	-0.663198093130
C	-5.404554899094	3.551218883398	-0.020413966247
C	-4.426085774980	3.882406780538	0.892046328912
C	-3.363083026839	3.003671446487	1.235721140799
O	-2.523197348795	3.511349962023	2.167350390578
C	-2.038834398943	2.692959200121	3.242479133822
H	-5.540570301054	-1.172439842459	-1.799576581898
H	-4.681805158646	-0.366750169479	-3.034187204038
H	2.356972330731	-0.243296717979	0.628008865728
H	2.897404138593	1.832834578346	-0.606747366581
H	1.086098472219	3.445912870926	-1.183713675491
H	-1.235604040972	3.001791720792	-0.524549834206
H	-3.425314295950	-0.589977961612	1.845323447887
H	-1.618603259767	-2.203416091413	2.370246864307
H	0.720393949736	-1.708604155356	1.716951280243
H	-3.327386643342	-0.416976614442	-0.960312495413
H	-7.100201343512	0.468136515422	-2.974892233641
H	-7.213303739528	2.619154318707	-1.755041015485
H	-6.211540216769	4.246773820740	-0.229913484455
H	-4.459318293006	4.838772127965	1.406809862546
H	-1.146093571441	2.136277057260	2.949923505306
H	-1.789889915294	3.391605509487	4.043010595382
H	-2.818610602729	2.004972681853	3.580990244837

3-Sigma-8’.xyz

39

-885.195716659

C	2.950047260566	0.519074501868	-3.563288400578
C	4.059756381399	-0.000974193932	-4.261113035174
C	3.962641117650	-0.625647503116	-5.515766308941
C	2.733090037068	-0.724215908795	-6.113862336018
C	1.520104270149	-0.180785195246	-5.498513803209
C	1.650630254824	0.409682934433	-4.141823986577
C	0.535291184901	0.875328026863	-3.429388516979
C	0.745747477417	1.449104231063	-2.169658985996
C	2.024275662269	1.576933694869	-1.600276879624
C	3.124637975069	1.111910485249	-2.286432162083
C	-0.850563646590	0.775660047317	-3.967230388043
C	-1.807755181461	-0.089502237756	-3.351698942671
C	-1.477039575819	-0.956318269503	-2.272008204189
C	-2.427527026802	-1.775127725673	-1.701275282409
C	-3.759692193648	-1.780074398992	-2.177391954641
C	-4.109797990471	-0.965034654193	-3.230366866435
C	-3.153960115594	-0.112009439347	-3.842119941301
C	-3.496984089216	0.718955674867	-4.936818144855
C	-2.565967658446	1.532841596074	-5.536448022026
C	-1.234692539968	1.561753072696	-5.051791505791
O	-0.269310757289	2.344137314702	-5.603105755497
C	-0.616508338348	3.245878579209	-6.652606587690
H	0.703584282839	-0.920989936805	-5.544919095209
H	1.144725891052	0.608560437214	-6.184803799128
H	5.039122357327	0.079930630706	-3.793828300974

H	4.853601764779	-1.016291732803	-5.995412022680
H	2.632593702241	-1.191278417052	-7.090238147523
H	-0.115924112365	1.814038029923	-1.616217806455
H	2.134570383206	2.036235777344	-0.622796081661
H	4.123503251212	1.188481355596	-1.866368392291
H	-0.457798761207	-0.982202274706	-1.897982225668
H	-2.147807569196	-2.429381985293	-0.879101983183
H	-4.498442505050	-2.430628009775	-1.716525942599
H	-5.127918336461	-0.964888970464	-3.614145844211
H	-4.518622496274	0.705884451827	-5.309817467862
H	-2.857530798112	2.153946145526	-6.376012921759
H	-1.378647326157	3.959097010620	-6.320695104257
H	0.303231116555	3.780489914627	-6.891962566133
H	-0.965986441543	2.706549195098	-7.539709283728

3-Sigma-8.xyz

39

-885.196013593

C	0.075175235750	0.057810983656	0.754061716261
C	1.340970247847	0.208787214267	0.127209064140
C	1.588787671583	1.263215558263	-0.722754080918
C	0.576694997320	2.217745349135	-0.979447610850
C	-0.661922024563	2.100293512880	-0.386774199603
C	-0.952849855584	1.020394495280	0.490369616579
C	-2.225822504881	0.860444336678	1.125900391544
C	-2.443674416649	-0.214468626830	1.967410048062
C	-1.423395034723	-1.156921890230	2.230102238914
C	-0.190608467700	-1.024062075138	1.634530746773
C	-3.362535771780	1.785589081613	0.820389526997
C	-4.146950651790	1.510466670035	-0.305246463358
C	-3.816870129128	0.360332368223	-1.201522294899
C	-4.693375759910	0.136318497100	-2.364427631310
C	-5.766838067508	0.931005921712	-2.632013968636
C	-6.056853432376	2.004233116469	-1.757255948197
C	-5.293684932769	2.304550897888	-0.626672864407
C	-5.652076334699	3.387805765336	0.223992193622
C	-4.896454377090	3.663447192045	1.323879862659
C	-3.741478610290	2.888032446911	1.633880020809
O	-3.134644751187	3.334252662097	2.728883583496
C	-1.856160786707	2.905060550104	3.222336584309
H	-2.780164304906	0.448194952010	-1.570121580190
H	-3.773917961441	-0.575096501788	-0.616908890372
H	2.115231460535	-0.527346393749	0.332400332882
H	2.561597693024	1.366928448618	-1.196364041488
H	0.778817414742	3.050878301002	-1.647787038659
H	-1.427713912801	2.845507146204	-0.588269837811
H	-3.416920557989	-0.337281960798	2.437273329812
H	-1.623921101783	-1.987493606277	2.901519736629
H	0.597015812557	-1.748812068782	1.828755438760
H	-4.447058766878	-0.700648871351	-3.012724779943
H	-6.401162692878	0.753278960803	-3.493942273549
H	-6.921183859357	2.630083870854	-1.970271939957
H	-6.525739136301	3.988234970701	-0.012858969804

H	-5.139308254529	4.484949788359	1.990176841426
H	-1.101950862269	2.950567273737	2.435522474791
H	-1.617825423150	3.622417799656	4.007809549568
H	-1.919453675831	1.899861271741	3.640395377302

8-sigma-1'a.xyz

35

-787.170670093

C	-0.007011778413	-0.011497818994	0.007021404392
C	0.018808761847	-0.002834017575	1.421451666116
C	1.286284108923	0.022275688366	2.097109533187
C	2.481717580285	0.040629425942	1.331373898032
C	2.420064761200	0.029357969842	-0.041025863113
C	1.170195561860	0.003770109724	-0.706223891432
C	-1.169029125191	-0.024306567705	2.191763473545
C	-1.097509708584	-0.022668621468	3.554043526586
N	0.125575252170	0.000821687963	4.159159888340
C	1.292710908994	0.024593148031	3.508709934039
C	2.559354681955	0.027681755294	4.344728076462
C	3.387381864153	1.285121954248	4.242518607546
C	4.811447817710	1.174033402764	4.299728781709
C	5.411185342766	-0.095735798948	4.335975552593
C	4.675060719402	-1.299374841231	4.294262455636
C	3.313000219757	-1.254948173614	4.245546237313
C	5.616931576476	2.343775360775	4.284945196353
C	5.024483703485	3.587015540682	4.207042062799
C	3.624795350647	3.682170487074	4.147591800778
C	2.811816180472	2.545928650671	4.171388391845
H	2.729662288492	-2.171392773984	4.212343141360
H	1.733339232386	2.665021903715	4.130596710124
H	3.158062608216	4.660791956609	4.081395513935
H	5.631220870333	4.486499499963	4.191667872650
H	6.697026821737	2.240912598633	4.334257505292
H	6.496613491901	-0.152078598108	4.377688652701
H	5.196518327984	-2.250339508342	4.289944996588
H	-0.965922578859	-0.031768432989	-0.502476159665
H	1.144675735458	-0.003144026374	-1.791994473599
H	3.336998058567	0.042809588295	-0.622184015707
H	3.448861432014	0.067365240486	1.821942393892
H	0.133446940706	-0.000787840054	5.176742438117
H	-1.953694751084	-0.038813224199	4.215110560490
H	-2.138902755014	-0.043772834612	1.706867367654
H	2.243549077975	0.002766404845	5.409396456076

8-sigma-1'b.xyz

35

-787.170251696

C	-0.018656231596	-0.018618556114	0.009368180204
C	0.017832262600	0.011232053108	1.423005150884
C	1.290782695337	-0.002436749193	2.096521135635
C	2.479114108280	-0.046233370047	1.315985636105
C	2.404868251255	-0.073658709041	-0.055775257979

```
C        1.151370155344    -0.060548734772    -0.713301584241
C       -1.170200724410     0.056661313549     2.190729126765
C       -1.099694462972     0.089852795712     3.552680597351
N        0.122430003741     0.073621182100     4.155785112717
C        1.292617584152     0.031279120049     3.508199963714
C        2.540130802016    -0.014274580548     4.379240434196
C        2.642152815652     1.112017713966     5.378341513358
C        3.053478298533     0.818505114793     6.713910959371
C        3.291896958648    -0.513293764817     7.092710485009
C        3.147433251404    -1.606212915763     6.210578321540
C        2.759309373011    -1.385967003367     4.923126058771
C        3.205595217489     1.866928289654     7.658842962958
C        2.950864300912     3.171350665771     7.287122417186
C        2.547103464573     3.447636310848     5.971536231574
C        2.396772605633     2.431234344756     5.023178792444
H        2.638705453275    -2.217329477193     4.233529521380
H        2.089685449867     2.686473618132     4.012770755730
H        2.346385314344     4.474091501767     5.678088402957
H        3.062194159704     3.979699210200     8.002458881488
H        3.524471511635     1.624552996841     8.668379952939
H        3.597274243803    -0.708930836765     8.118355850421
H        3.337662435158    -2.613206448672     6.565647730687
H       -0.982278336832    -0.006596069385    -0.491390799381
H        1.116874000411    -0.082333771012    -1.798637426971
H        3.318203309759    -0.105496647593    -0.642383522784
H        3.456326137315    -0.061893934858     1.786030077716
H        0.129771536838     0.103127316577     5.173155220737
H       -1.955321619538     0.129790318120     4.213591958458
H       -2.139221566159     0.067910810013     1.703979393924
H        3.404325843586     0.124341065196     3.704544367533
```

8-sigma-2'.xyz

```
35
-787.169164054
C        1.414091288749    -0.248093923498     0.265752833962
C        2.062423595599     0.562514124288     1.261223247893
C        1.338888193527     1.456374060715     1.999376700296
C       -0.059049099870     1.586096531912     1.785109064382
C       -0.721161557222     0.830848704415     0.834150486660
C       -0.020455276165    -0.098157245950     0.049103224862
C       -0.693385262217    -0.876020734536    -0.941782899489
C       -0.014164974210    -1.770498907675    -1.701323169456
C        1.431023736559    -1.980672663797    -1.528737216143
C        2.106054597359    -1.164872918925    -0.502317545247
C        3.570340816543    -1.388958907734    -0.327826356412
C        5.538112415228    -2.407813510245     0.696509490860
C        6.101144038813    -3.259302628939     1.675622535069
C        5.286708915939    -3.917219097859     2.569392116932
C        3.879624475800    -3.757056534746     2.526004685857
C        3.295713146201    -2.937974223579     1.590752944790
C        4.110734145102    -2.242832656902     0.656387127378
N        4.390805467539    -0.772564885424    -1.186290231746
C        5.748374020528    -0.903361639787    -1.174111426310
```

```
 C          6.335312266611     -1.713535917105     -0.244710264141
 H          1.949371842556     -1.866163293151     -2.499980240064
 H          1.626153765100     -3.050371472653     -1.316976428609
 H          3.131090751146      0.463154369765      1.420755763189
 H          1.825833349426      2.069683408253      2.750343846362
 H         -0.619578858433      2.299272996928      2.382852759709
 H         -1.789832085774      0.951211286503      0.686130235583
 H         -1.761317149588     -0.731272990480     -1.073608505721
 H         -0.517361211473     -2.363233192353     -2.459000363755
 H          7.179331580871     -3.384206527058      1.708565139463
 H          5.724366236077     -4.570111430293      3.318908719769
 H          3.258065237645     -4.288083540411      3.240339370741
 H          2.216723851435     -2.823464941222      1.568895453492
 H          3.990031765775     -0.144715160773     -1.880445527704
 H          6.285058058392     -0.336394504208     -1.923454507271
 H          7.414644369291     -1.819109215199     -0.234484997912
```

8-sigma-3’.xyz

```
35
-787.177746572
 C          1.385778315405     -0.308059177837     -0.210446112498
 C          1.931297245620      0.437701683315     -1.258253852953
 C          1.126161538811      1.288582401931     -2.010016744847
 C         -0.252949900584      1.437656168328     -1.752916174973
 C         -0.823462603889      0.729648446015     -0.724225157451
 C         -0.026890184846     -0.153411832995      0.069884120088
 C         -0.595385416675     -0.852694197047      1.114249400927
 C          0.178182811896     -1.754289438971      1.960479505466
 C          1.607908437431     -1.895428303751      1.631878839384
 C          2.173643209798     -1.216535815596      0.603449182538
 C          3.623697442674     -1.405914225253      0.319399675614
 C          5.548812723864     -2.334573151329     -0.869136969934
 C          6.065153372986     -3.161132869185     -1.894281791768
 C          5.208030850175     -3.857963923931     -2.716710185467
 C          3.804334266246     -3.765776603255     -2.549993131891
 C          3.266826170520     -2.972400402708     -1.565103598810
 C          4.125534071133     -2.234583847452     -0.707029525905
 N          4.487583999928     -0.760715313462      1.110710897543
 C          5.845200320503     -0.835215870981      0.993606137850
 C          6.390195214075     -1.609154224359      0.010191157155
 H          0.052277805301     -1.444024431732      3.016259922606
 H         -0.316441556467     -2.744499265492      1.974122285825
 H          2.987216216354      0.365710392533     -1.497568870962
 H          1.578020350014      1.855184926860     -2.819459530600
 H         -0.848420914368      2.109271841548     -2.362249325922
 H         -1.880301006267      0.827244028184     -0.493599344654
 H         -1.654634091774     -0.735744688418      1.331449716651
 H          2.196971480889     -2.575543679188      2.240397563058
 H          7.141252016660     -3.238496473022     -2.018690061734
 H          5.609841169912     -4.492232234466     -3.501621108181
 H          3.149133057758     -4.331416176985     -3.205479031664
 H          2.190916752759     -2.914491298103     -1.435586008520
 H          4.108490025688     -0.171943774834      1.849411276321
```

H	6.415961796330	-0.255868575435	1.707523060764
H	7.468784170848	-1.666121555494	-0.088925030402

8-sigma-3.xyz

35

-787.153758321

C	-1.995430335313	2.226228077385	0.713325382324
C	-0.955733522392	2.832969293703	1.464286450941
C	-1.244935845062	3.689096236172	2.505931035348
C	-2.582367327417	3.989158074213	2.846010652371
C	-3.612908109306	3.440117431422	2.118061242500
C	-3.350877348014	2.559907821820	1.036010055768
C	-4.412212597978	2.025417925125	0.261530585378
C	-4.167877435977	1.205051908526	-0.817250934824
C	-2.845257822811	0.871971009642	-1.157714134534
C	-1.776265524244	1.344080314815	-0.402922087647
C	-0.445805429900	0.825135984368	-0.725289468116
C	1.384265137658	0.233030975573	-2.331366590080
C	1.946465267052	0.255435866185	-3.636177393253
C	1.294520583990	0.931032276098	-4.652851974550
C	0.098466398757	1.603060769150	-4.374691531755
C	-0.483125115235	1.592676645791	-3.093418067302
C	0.124911895685	0.887403543676	-2.068777444763
N	0.261785805297	0.260892801193	0.225473193917
C	1.568506826925	-0.329744903059	0.079238114204
C	2.072132861302	-0.345451574240	-1.284427798366
H	1.548295217670	-1.357508818609	0.480250769725
H	2.297570771661	0.194045064432	0.724849377845
H	0.085123201497	2.648329958742	1.215777811995
H	-0.431590273266	4.143445071807	3.064979819331
H	-2.792429893643	4.663419207210	3.671491210757
H	-4.646581238268	3.681446373647	2.353302531689
H	-5.433603443638	2.283255623323	0.530718206065
H	-4.987128987434	0.800904921993	-1.403539063511
H	-2.670398812611	0.187852455380	-1.983012329545
H	2.891243448708	-0.249280967881	-3.813077473634
H	1.714484346547	0.957643761666	-5.652457874888
H	-0.398472636637	2.156505265770	-5.165809100039
H	-1.397453140580	2.148378467153	-2.921986160282
H	-0.160923458724	0.178684063987	1.148563735099
H	3.041599076045	-0.811310799175	-1.443525051663

8-sigma-4’.xyz

35

-787.175718576

C	1.356647363621	-0.127922524070	-0.213103732141
C	1.922697311296	0.788046497485	-1.144064420923
C	1.147873551665	1.790725984330	-1.680934334772
C	-0.205992879318	1.905792973500	-1.310839235679
C	-0.785822998525	1.013394185198	-0.415247885171
C	-0.026665927501	-0.012530666142	0.142100880862
C	-0.630334423600	-0.982613281619	1.090162739071

C	0.237984906158	-2.041827172671	1.598546049435
C	1.557324250198	-2.135542417240	1.239089680522
C	2.113267959143	-1.177635769135	0.364085776263
C	3.570794343018	-1.306592257480	0.080155446204
C	5.549255476676	-2.404478771307	-0.831418184744
C	6.123613324062	-3.449092839636	-1.592818777338
C	5.316938113373	-4.391506486856	-2.189827130664
C	3.907223171386	-4.329371200660	-2.061927182274
C	3.312358594297	-3.329729893486	-1.330937688913
C	4.119784127879	-2.352344124423	-0.689329984722
N	4.381411115910	-0.395848896111	0.632026891125
C	5.739867661688	-0.415791043271	0.517993719057
C	6.336947347189	-1.409069938801	-0.205635010626
H	-1.525250960505	-1.453730134942	0.642416974110
H	-1.071282037372	-0.455645506678	1.957392515920
H	2.961177978637	0.690005020402	-1.443113260067
H	1.575943038193	2.488541907179	-2.393333605543
H	-0.810648776258	2.700507753449	-1.738287432188
H	-1.834616483427	1.112013115716	-0.150065139299
H	-0.188854397457	-2.760929446202	2.292159942987
H	2.186906986211	-2.920519430497	1.644500502230
H	7.203400611938	-3.492272919278	-1.699374142379
H	5.762689352510	-5.192606283462	-2.772147152599
H	3.291895544515	-5.078390171144	-2.550792624625
H	2.231020860978	-3.284942612416	-1.251197415601
H	3.970694497130	0.340665535986	1.202616118675
H	6.270109200445	0.377309663230	1.028693394050
H	7.417659806100	-1.427779525669	-0.294732707366

8-sigma-4.xyz

35

-787.160175911

C	0.065293130892	-0.072200655297	-0.079762747523
C	0.020994642960	-0.006667568023	1.409207154043
C	1.236508363152	0.055378625030	2.155032561926
C	2.483127643476	0.034206051034	1.475229119982
N	2.461992879763	0.126273173902	0.073266001263
C	1.390946948965	0.062295733954	-0.664421553408
C	1.184065838724	0.215475981786	3.565646730162
C	-0.033988261477	0.226940427932	4.213513215616
C	-1.220545033765	0.109143108384	3.473513887628
C	-1.192823478295	0.010837347102	2.082204399114
C	3.770993269380	-0.139577449245	2.069810596139
C	4.953342627751	0.549118943368	1.599740486426
C	6.226924093826	0.082507919837	2.053022653296
C	6.290788358674	-0.984317732327	2.982663489357
C	5.144327093797	-1.579341052471	3.482959346631
C	3.891511495034	-1.158199054886	3.038572480732
C	7.406725427638	0.729184568451	1.611084594096
C	7.336758042501	1.822479638845	0.770876994317
C	6.081737882376	2.321420572768	0.379020386143
C	4.910268164216	1.709833636362	0.798963170969
H	1.535767403679	0.072178893525	-1.740328521359

```
 H          3.961111380124      2.169599435866      0.537325274024
 H          6.023128319740      3.207849858482     -0.245882549896
 H          8.244820893536      2.313943365287      0.434836578736
 H          8.367403576517      0.361615063140      1.961445391368
 H          7.267194963815     -1.331354423792      3.311142827616
 H          5.213674393650     -2.401331594214      4.187690394477
 H          3.008371051087     -1.702150056030      3.356072002751
 H         -2.121750594247     -0.043955538064      1.522508473018
 H         -2.177829656228      0.125688700927      3.986287977841
 H         -0.074071960432      0.353949649525      5.290404002200
 H          2.098137252029      0.370765966893      4.127251574956
 H          3.368053577695      0.132975030574     -0.397363220543
 H         -0.337446260296     -1.029603523330     -0.463147268315
 H         -0.596771286316      0.672657701245     -0.550641150038
```

8-sigma-5’.xyz

```
35
-787.182942255
 C          1.427031868933      0.821924486475     -1.569788591191
 C          1.334338891830      0.597775204391     -0.176086006293
 C          0.121140365865      0.544156184320      0.523885191722
 C         -1.055206362551      0.717201884776     -0.161379650996
 C         -1.080459852427      0.964606913686     -1.603114828114
 C          0.226646379588      1.026677812662     -2.307534031798
 C          0.291859700131      1.291346681509     -3.677822653141
 C          1.522418558198      1.356948588865     -4.312280000835
 C          2.719789888990      1.153476105889     -3.600205999963
 C          2.685981905426      0.879521562512     -2.246017461070
 C          3.969255543824      0.667808816699     -1.524424971460
 C          5.736390520021     -0.652713736957     -0.467992372792
 C          6.243817288141     -1.910921095771     -0.068501981748
 C          5.527395832390     -3.058349656902     -0.326762751735
 C          4.281804879480     -3.002466897175     -0.998953447403
 C          3.756186084189     -1.797794083309     -1.400186311526
 C          4.465840783468     -0.595441125684     -1.135662305020
 N          4.706535844926      1.756762276328     -1.276860770339
 C          5.921021624096      1.742368792987     -0.655448268066
 C          6.445800974168      0.552439875080     -0.241173716857
 H         -1.662813441515      1.886129956101     -1.787215944949
 H         -1.728117454325      0.198097242694     -2.069267805847
 H          2.251294289584      0.463285030466      0.392566773981
 H          0.126837875597      0.367705836407      1.593786972817
 H         -2.008647671155      0.674201630088      0.358412410349
 H         -0.622428696924      1.449791324423     -4.242514685657
 H          1.570896496849      1.565905298170     -5.376665223261
 H          3.669214335024      1.199523644956     -4.125178313766
 H          7.202857502106     -1.956709865839      0.439005708119
 H          5.922507116523     -4.021918457647     -0.018076172316
 H          3.741541746228     -3.922100096187     -1.202065412727
 H          2.808290098188     -1.762573047016     -1.927384744278
 H          4.331997711339      2.659005448282     -1.562440860725
 H          6.399588872928      2.703985279956     -0.523492342056
 H          7.409000515398      0.537814220308      0.257393143038
```

8-sigma-5.xyz

```
35
-787.167335007
 C         4.363454126300      1.406052747943      0.561897380985
 C         4.409633275375      2.116416081691     -0.664743257599
 C         5.566895944895      2.750207135954     -1.068005855746
 C         6.733776503082      2.694066496509     -0.275419666548
 C         6.729844444254      1.981913013107      0.903616210860
 C         5.558373252746      1.315646097183      1.345736640508
 C         5.561597763836      0.542065383775      2.535928106300
 C         4.439226740174     -0.142114361470      2.946984163008
 C         3.253954072926     -0.059477993647      2.194800425808
 C         3.198202160859      0.713481317080      1.038111976903
 C         1.894025538770      0.860836067785      0.395419394239
 C        -0.253796567687      0.035616622661     -0.475875064139
 C        -1.127624195416     -1.106905561887     -0.842162149999
 C        -0.544527844315     -2.445936696196     -0.734680348913
 C         0.760868044900     -2.651972626976     -0.353638071748
 C         1.555899630382     -1.552746494644     -0.017370386195
 C         1.062158830221     -0.220709997973     -0.007405801184
 N         1.400457921989      2.102485688675      0.215265653072
 C         0.168792485503      2.376369802265     -0.269404853212
 C        -0.689577398032      1.355400647259     -0.609434575265
 H        -2.062215765774     -1.075005784156     -0.251906715185
 H        -1.510504747473     -0.977087641879     -1.871524072893
 H         3.540703892819      2.148059488768     -1.316103207480
 H         5.580939228752      3.288521196862     -2.011658893332
 H         7.636661965077      3.199646390602     -0.606212876248
 H         7.630332945622      1.910113488781      1.508542768587
 H         6.475777834242      0.492515552033      3.122351665585
 H         4.451752193430     -0.729669736352      3.859648370459
 H         2.356598433919     -0.549905762054      2.562227681633
 H        -1.179584900395     -3.288438365634     -0.994986321523
 H         1.184981233283     -3.649299079195     -0.327261513448
 H         2.600325208744     -1.718919824076      0.229687151250
 H         1.974501427442      2.887216392049      0.521855253942
 H        -0.090269591068      3.425205410289     -0.347665161005
 H        -1.680624592895      1.592032277805     -0.979173238899
```

8-sigma-6’.xyz

```
35
-787.177464471
 C         1.326767338639      0.551745111133     -1.398149429342
 C         1.240839714948     -0.088278102693     -0.116610907603
 C         0.039571016116     -0.398814110370      0.428517807108
 C        -1.220346195683     -0.097923225814     -0.271383537430
 C        -1.105421726394      0.562895980541     -1.565117206296
 C         0.118171159619      0.886012902101     -2.121383448988
 C         0.187133386406      1.536031387024     -3.392220806716
 C         1.404574386491      1.847646023663     -3.940501745297
 C         2.584878799544      1.513229603856     -3.243574455495
```

C	2.560488703113	0.877796691695	-2.004102294984
C	3.853692420544	0.545866955381	-1.342794407456
C	5.773054729207	-0.880198129088	-0.826529401488
C	6.448100261684	-2.114311228573	-0.973167958879
C	5.904407327362	-3.109923245164	-1.754271142192
C	4.670136010809	-2.918321690223	-2.422503986937
C	3.985834194872	-1.732678123214	-2.303062498748
C	4.519788187859	-0.687994927063	-1.501595253428
N	4.413822633789	1.490689446719	-0.578956278190
C	5.604571908408	1.348851190809	0.072906701960
C	6.292918629209	0.175483813757	-0.037766559845
H	-1.824777989288	-1.017955484956	-0.401537341428
H	-1.899908810545	0.495930214175	0.370883111784
H	2.154213984636	-0.323390840610	0.420588205420
H	-0.020249538156	-0.882856414394	1.398430645194
H	-2.021562207657	0.800714004087	-2.100633258567
H	-0.737471383152	1.775700426005	-3.909290013384
H	1.474271218944	2.341949209963	-4.903445152698
H	3.544754844121	1.753673258515	-3.693490372982
H	7.395062884365	-2.262713566924	-0.462541127284
H	6.426655779261	-4.056173377359	-1.862914917542
H	4.263589568083	-3.717425460566	-3.034914778662
H	3.042615433206	-1.589955167477	-2.820429958516
H	3.920589181575	2.375225496720	-0.474208440354
H	5.932499754511	2.200989096056	0.653655321761
H	7.238153800322	0.060496381200	0.481800034558

8-sigma-6.xyz

35

-787.145942398

C	4.191527555530	1.408643970569	-1.222101936517
C	3.639270198381	2.127511409637	-2.313468022659
C	4.456147848189	2.772252568085	-3.218629666361
C	5.861337518205	2.725490339766	-3.085227115512
C	6.428489343765	2.011062454399	-2.054398101455
C	5.617696042281	1.331505078105	-1.108612971387
C	6.200812235126	0.562375547982	-0.069295071724
C	5.420677027166	-0.132475190398	0.828862233738
C	4.019695643858	-0.070185487493	0.733818332184
C	3.405609999488	0.697890832971	-0.249968337117
C	1.947742769437	0.827956597740	-0.188628126897
C	-0.365290689897	0.007605308005	-0.011203207442
C	-1.262662081111	-1.062181011638	0.046164742885
C	-0.810945251632	-2.428329200767	-0.096767424114
C	0.614055387668	-2.655045392898	-0.334752315708
C	1.493855026516	-1.612721621912	-0.372394406912
C	1.031982253183	-0.280929913326	-0.175448186840
N	1.416846980536	2.055031084130	-0.112743760177
C	0.092630505435	2.362994665087	0.001579485371
C	-0.820435397533	1.352990809361	0.076624664097
H	-1.433325372961	-2.932480819395	-0.867554293170
H	-1.159048845456	-3.009567170813	0.786481817882
H	2.563832644541	2.160211956883	-2.464539882141

```
 H         4.011269496779      3.314704186766     -4.048288727098
 H         6.490181562229      3.241637901410     -3.805206505225
 H         7.509218640117      1.946926723867     -1.954569724840
 H         7.284836282606      0.522547660340      0.004196072936
 H         5.876047361132     -0.717480341767      1.621868221497
 H         3.416394911352     -0.584416457507      1.476863285415
 H        -2.321788298536     -0.876074439374      0.202775314325
 H         0.954183372848     -3.674746354193     -0.483716362233
 H         2.544613505370     -1.789810711232     -0.567412127826
 H         2.072564666957      2.836820422493     -0.102190222662
 H        -0.149299312577      3.416834254661      0.046751090090
 H        -1.874918585957      1.574723754386      0.193352140702
```

8-sigma-7’.xyz

```
35
-787.175919013
 C        -0.355068085369      1.613185608567     -1.113009516641
 C         0.446196833336      1.579298888024      0.012275689459
 C         1.725069333421      0.879280418290      0.036505513526
 C         2.138536959166      0.200365410921     -1.201137590762
 C         1.358141389135      0.239736253882     -2.309382696143
 C         0.108449673635      0.939509191010     -2.308170704771
 C        -0.689104874405      0.986513887747     -3.467963492665
 C        -1.889345609956      1.664868390966     -3.459769022249
 C        -2.360553654565      2.327826719571     -2.296720148950
 C        -1.621349269102      2.305980051845     -1.141012597536
 C        -2.149442673307      2.996368823140      0.068182125478
 C        -2.605188094368      4.932057167070      1.490417575525
 C        -2.479528604688      6.318838247933      1.738218897474
 C        -1.795270544789      7.119362847883      0.850921462318
 C        -1.207180774717      6.574184643315     -0.316932324397
 C        -1.308812342752      5.231596382616     -0.591174256014
 C        -2.011773856328      4.380749658351      0.303956100773
 N        -2.822969928429      2.250831873897      0.952408998998
 C        -3.397457065651      2.737001432393      2.090436469890
 C        -3.298390746549      4.068737692914      2.373762387557
 H         1.716684898401      0.171815526067      0.889754149120
 H         2.505624216716      1.588109852519      0.377280782610
 H         0.143094477782      2.083801534090      0.926124757576
 H         3.086282954201     -0.329099907397     -1.200808052549
 H         1.665013657743     -0.257880596903     -3.224423511887
 H        -0.349185463354      0.485502199100     -4.369102291352
 H        -2.497084554327      1.696434946277     -4.359132756391
 H        -3.312652211349      2.848084895885     -2.329720694120
 H        -2.928938884048      6.737912715957      2.633707529834
 H        -1.702306006441      8.183836139344      1.046053530649
 H        -0.671251693638      7.225822671885     -1.000284241133
 H        -0.857185646808      4.819487034494     -1.487665012610
 H        -2.930212848110      1.257332115445      0.758979074978
 H        -3.911502468896      2.013736345021      2.709642687087
 H        -3.755467239307      4.457961597063      3.277168455412
```

8-sigma-7.xyz

```
35
```

-787.159841790

C	4.324657012411	1.403838690640	0.841706875642
C	4.478455032112	1.937681095872	-0.461034058101
C	5.673717053060	2.509591001488	-0.852283065220
C	6.773373753795	2.559118385173	0.028497425914
C	6.667894820739	2.008785989212	1.287513844557
C	5.456210114612	1.413467386556	1.718876260697
C	5.364427800270	0.800400630220	2.995495453874
C	4.208542900417	0.173767922631	3.410192082491
C	3.085554201027	0.157541116219	2.570554419415
C	3.116591105902	0.777335957650	1.316347606820
C	1.859637519911	0.857867355084	0.599996660592
C	-0.362391493204	-0.034495876724	-0.111334916408
C	-1.284154107194	-1.120135098342	-0.221652026451
C	-0.898179273171	-2.383511431395	0.090359047101
C	0.481504136165	-2.685431472043	0.507429182703
C	1.390240918786	-1.553254449878	0.642085082248
C	0.965486107125	-0.256027951447	0.416816029814
N	1.447756964382	2.044312253340	0.120792561099
C	0.224008840052	2.268924755419	-0.431555803422
C	-0.701248645024	1.263605354686	-0.535727463599
H	0.492114551044	-3.251871423653	1.461040788851
H	0.939618361436	-3.434014994237	-0.168961599115
H	3.670346672505	1.877999808385	-1.184484179473
H	5.767722387394	2.913830466479	-1.856206165058
H	7.705739534983	3.013179840313	-0.294435394098
H	7.518194375483	2.010664221817	1.964639898105
H	6.231979467757	0.830075615056	3.649936197356
H	4.147676864357	-0.283561625702	4.392489172302
H	2.156771081834	-0.263838562891	2.943227615831
H	-2.289891716208	-0.911499568919	-0.571968412145
H	-1.596966703642	-3.211259105320	0.016804763265
H	2.425806920684	-1.759433996635	0.896129597777
H	2.053452150741	2.851263042436	0.262941300204
H	0.034864958323	3.287281380910	-0.749308595907
H	-1.677520495810	1.478346981752	-0.954340885364

8-sigma-8’.xyz

35

-787.183805773

C	-0.060479179721	2.133402018002	-0.201859820600
C	-0.179891964907	0.652261865475	-0.310392070187
C	1.069074264124	-0.118176924821	-0.317751969140
C	2.302240129168	0.477188615033	-0.255786714096
C	2.374333206736	1.875495067821	-0.178313313794
C	1.238153612476	2.717032540536	-0.150890079510
C	1.398565699111	4.122362380905	-0.069583451000
C	0.289206593781	4.940742463386	-0.036499591753
C	-0.990583301682	4.370544424612	-0.079367893943
C	-1.173463794631	2.985324366299	-0.159328774402
C	-2.564273070455	2.453442999544	-0.196751696203
C	-4.593895548077	1.487601493816	0.769982492666
C	-5.287539662172	0.987306963695	1.896687030332

C	-4.675150991805	0.962959825030	3.129989523377
C	-3.351424081658	1.439693295369	3.294583005631
C	-2.646820181017	1.932361644682	2.222419555515
C	-3.248715425101	1.961237411030	0.935764975564
N	-3.199405723623	2.479390324817	-1.373893737037
C	-4.478889766276	2.047262758739	-1.570774159445
C	-5.185717451773	1.546320328692	-0.516053841020
H	-0.828292835472	0.240112330944	0.482632654924
H	-0.732308323093	0.372656860390	-1.227392631411
H	0.980965519497	-1.199343059998	-0.384482777497
H	3.211828099176	-0.112953492164	-0.268667220181
H	3.354738054309	2.344904668290	-0.134820701394
H	2.400136150711	4.540806643533	-0.034304362298
H	0.395841624722	6.018588430459	0.025772662050
H	-1.863265761614	5.016931886892	-0.042750510266
H	-6.304972026696	0.627896938535	1.773951036852
H	-5.212941761204	0.578428924194	3.991827215705
H	-2.893090185297	1.418478752001	4.278634193850
H	-1.637111572048	2.308390428562	2.355097349462
H	-2.694703219482	2.840324260125	-2.180808403591
H	-4.858511301415	2.129822557061	-2.580711351512
H	-6.201642600521	1.198941320252	-0.670147761309

8-sigma-8.xyz

35

-787.159491215

C	4.235510787984	1.344519217119	0.924752868888
C	4.306359874884	1.732945786320	-0.439478584885
C	5.502110251501	2.131809181578	-0.995609649759
C	6.685016988964	2.155420874964	-0.221459169793
C	6.652258820116	1.764540039478	1.097697653965
C	5.438183338949	1.345183948618	1.702957357512
C	5.398224452149	0.919796719153	3.056169184800
C	4.222649611928	0.500095678993	3.635561502287
C	3.028561474279	0.499381625821	2.885785394226
C	3.025919675848	0.920419864682	1.566366200566
C	1.736134586881	0.964947034652	0.847122700668
C	-0.242124015394	0.026403219969	-0.200736632723
C	-1.014173544238	-1.124579201053	-0.585427628185
C	-0.598223858460	-2.424069218626	-0.343681103064
C	0.618456878331	-2.630849449820	0.289410767219
C	1.477375282798	-1.538565652555	0.722105532666
C	0.984757418300	-0.163182291285	0.470931575682
N	1.233364600277	2.174681804325	0.527109691282
C	0.069264158272	2.397074754554	-0.120536398262
C	-0.702534132067	1.324130932496	-0.497004960615
H	2.468031652433	-1.690779189949	0.247349503520
H	1.734915845544	-1.705631680665	1.784368382130
H	3.416304284824	1.708547938561	-1.062344761534
H	5.536216960217	2.426180076716	-2.041086811319
H	7.619598366864	2.475050491002	-0.674321892192
H	7.559574878184	1.766777260535	1.697097159627
H	6.320028841452	0.928345423342	3.632853303069

H	4.200519638240	0.178504594570	4.672430382371
H	2.099312444425	0.203271808305	3.366344708417
H	-1.962603381130	-0.953579724137	-1.088551100059
H	-1.213427296205	-3.261793002229	-0.652095736930
H	0.969665135071	-3.642454828446	0.476381340114
H	1.775264084598	2.988803338494	0.816861893199
H	-0.194979352915	3.431241493072	-0.301292488809
H	-1.641941971488	1.486476105902	-1.012595550199

9-Sigma-1’-a.xyz

35

-787.175959807

C	-0.007238140750	-0.416397640379	0.187712202684
C	0.004221019574	-0.111598973884	1.544519211235
C	1.261264920460	0.064275376681	2.202300395697
C	2.472999228309	-0.071537057010	1.473726188527
C	2.436467857847	-0.378779320616	0.130111947307
C	1.195229456035	-0.552152025383	-0.506726556328
C	1.290618440387	0.340278755628	3.581041001042
C	0.129377240258	0.439892191448	4.372741772977
C	-1.096703028239	0.289510207229	3.786657642167
C	-1.264497398593	0.113364788089	2.322834724008
C	-2.448316355164	-0.765901973287	1.936062062481
C	-2.447915358325	-2.176275510566	2.181936580849
C	-3.579423626022	-2.969587894297	1.794705371804
C	-4.691137903740	-2.343871276032	1.181784488914
C	-4.673336302417	-0.987895485438	0.963626386373
C	-3.553981019407	-0.204893028860	1.338277579576
C	-1.374176259769	-2.837423663005	2.795248057673
N	-1.414547648916	-4.150443782774	2.998981142669
C	-2.466596973447	-4.939781625616	2.639696769862
C	-3.551113530538	-4.365316249369	2.040762495098
H	-2.005081010315	0.368084283480	4.378988622763
H	-0.948498843087	-0.553695871562	-0.336021746981
H	1.168070943880	-0.798989178673	-1.564389962839
H	3.356293583483	-0.487407654098	-0.435566969071
H	3.418089439353	0.069326451276	1.990565528329
H	2.259501381488	0.468114130684	4.058824322915
H	0.216596466169	0.627908856552	5.437380617063
H	-4.392955073189	-4.986409639661	1.754806557163
H	-2.373060371996	-5.995205966953	2.858911427539
H	-0.615209122241	-4.590379787876	3.448153830903
H	-0.473105783916	-2.340067904191	3.130902054223
H	-3.576882319855	0.863316819740	1.140473784775
H	-5.522192877237	-0.499767178694	0.494291125091
H	-5.547121326887	-2.945323947469	0.890997821489
H	-1.569250182397	1.145376613614	2.035688227953

9-Sigma-1’-b.xyz

35

-787.175258710

C	0.397474367019	0.446495617840	0.030673249422
C	0.193223429281	0.101953597726	1.361646873650

C	1.323470193321	-0.004756898579	2.226482678294
C	2.630989896694	0.236080130954	1.727222865786
C	2.809798006051	0.576267519318	0.402466602584
C	1.690415567631	0.680949566228	-0.440430011305
C	1.131876227095	-0.331209182577	3.581507328093
C	-0.136597171789	-0.571416677138	4.145908008446
C	-1.246150716155	-0.499706953773	3.352726674320
C	-1.180071960219	-0.207962565022	1.896908563391
C	-2.245261104672	0.825686176327	1.520325046087
C	-3.462824841456	0.489709640857	0.848855650773
C	-4.422166862014	1.522803635235	0.564419723239
C	-4.153953609525	2.854014524435	0.956434906120
C	-2.978633810253	3.149856850439	1.605270265496
C	-2.032547039151	2.138301885024	1.884183378439
C	-3.784613061494	-0.814042549354	0.438095158721
N	-4.929974458019	-1.065605661164	-0.186894423238
C	-5.863386768110	-0.113532514475	-0.475606485066
C	-5.622383507513	1.178263512838	-0.107887075253
H	-2.232366293035	-0.687318316046	3.769827466391
H	-0.444619704145	0.537682017344	-0.649137937987
H	1.830270731166	0.952380932776	-1.483088802937
H	3.804464635575	0.762688982764	0.010378626561
H	3.478724349560	0.146010416810	2.400525685746
H	2.008095905585	-0.396844914614	4.222923545627
H	-0.221334790417	-0.808966858126	5.200802627919
H	-6.357991851505	1.943021226375	-0.332348967929
H	-6.754191090417	-0.451445364193	-0.988446119903
H	-5.121224005429	-2.025016549496	-0.465340481961
H	-3.147245740763	-1.674659937826	0.595545494246
H	-1.113493088266	2.411031751543	2.395639463316
H	-2.765893597042	4.169354804656	1.912698971766
H	-4.883142484400	3.628898970949	0.740411528602
H	-1.441628325942	-1.180327915128	1.440340898424

9-Sigma-1.xyz

35

-787.158818860

C	1.040038200682	-2.459270087522	2.713372248338
C	1.980553088731	-3.057226953119	3.570355506449
C	2.413985701014	-2.381058779254	4.688631326949
N	1.923306889451	-1.152185285230	4.953506011039
C	1.026660518894	-0.532905527590	4.169545668533
C	0.571525253153	-1.158107956992	3.021146508168
C	-0.440692976231	-0.509746408827	2.169450021275
C	-0.952983600681	-1.251988647928	1.024589869630
C	-0.411831663481	-2.588355702832	0.754415621308
C	0.523585809343	-3.152051673245	1.549126052644
C	-0.800953061876	1.268931544444	0.301962938306
C	-2.116454585793	1.550811364541	0.700605163791
C	-2.750467413932	2.708739550049	0.273320097674
C	-2.100430637717	3.632993785947	-0.566316681496
C	-0.809579777255	3.383360329497	-0.974096067087
C	-0.137591432162	2.205941858239	-0.552296871472

```
C        1.186060358637      1.955669460348     -0.970458565799
C        1.881805295586      0.810235851056     -0.587281622872
C        1.266930906126     -0.116432548020      0.231071605933
C       -0.059469038912      0.073588401688      0.710277853745
H        2.368079631691     -4.048974275113      3.365561740200
H        3.135844037722     -2.776764077460      5.391873729502
H        0.700490456503      0.451235309271      4.485585922410
H       -1.965998102877     -1.070793502389      0.678482596523
H       -0.820687992786     -3.132668688550     -0.091167662461
H        0.887481146304     -4.154711263746      1.346306718904
H       -2.669167642808      0.873755572687      1.345093927954
H       -3.770089959660      2.900627643333      0.595460205354
H       -2.616548921261      4.532565033644     -0.887167088483
H       -0.285839854514      4.080224978067     -1.622707134414
H        1.671327011167      2.681885736332     -1.618202575261
H        2.895531211834      0.641626153121     -0.934185275859
H        1.809672550096     -1.014945999575      0.506576694234
H       -1.081063439408      0.209192022475      2.671442017366
H        2.248273826691     -0.669608814357      5.787475895962
```

9-Sigma-2'.xyz

```
35
-787.179038951
C       -0.050972889009      0.103891969846      0.004112471735
C        0.018698332651     -0.049413040614      1.409631948431
C        1.297271777977     -0.053078987403      2.056183734774
C        2.488690373694      0.096061807477      1.271150332084
C        2.367646977665      0.282766492017     -0.092322028593
C        1.102401602584      0.272385426248     -0.724135514287
C        1.321374749791     -0.152013675796      3.456321516842
N        0.188361708987     -0.270205186564      4.139892384374
C       -1.046983041629     -0.299258456430      3.562275925661
C       -1.146849548399     -0.187552858162      2.205054653414
C        3.839571034473      0.084604156253      1.878781441294
C        4.405467679577     -1.053792989609      2.446993872560
C        5.723569628673     -0.958832800219      3.048902845998
C        6.450090439945      0.276062785669      3.036280527362
C        5.928780549536      1.378238374013      2.450029411134
C        4.583783820184      1.358629015159      1.850343505303
C        6.279995560078     -2.110526120226      3.628521820703
C        5.593794386156     -3.312153408748      3.605163829429
C        4.322658439300     -3.425503181731      2.990000446069
C        3.739814326416     -2.324054077546      2.421631358406
H        4.611677718953      1.739960586085      0.812398196273
H        3.953834934606      2.128242218576      2.337400439336
H        2.776958053622     -2.411012073401      1.929490983478
H        3.820077387759     -4.387009511507      2.964876109134
H        6.044259210621     -4.192002881315      4.056090441819
H        7.261451800461     -2.050939648324      4.089173265475
H        7.434740909950      0.298929390850      3.493937308371
H        6.479467085193      2.313456604603      2.415905656124
H       -2.126059648133     -0.198292376015      1.739131220916
H       -1.889501972343     -0.404718642818      4.232775758984
```

H	0.245925693269	-0.336654464765	5.153362708195
H	2.231361372355	-0.128565259846	4.043372804548
H	3.257280356756	0.399458736935	-0.704153904795
H	1.053973121402	0.397613710981	-1.801484780582
H	-1.021198604199	0.096439380893	-0.483210289736

9-Sigma-2.xyz

35

-787.165958656

C	0.143875007201	-0.083432282796	-0.031128743033
C	0.042434825497	0.007843177366	1.369253998083
C	1.256833642556	-0.029348312187	2.127550085136
C	2.496169460124	-0.237636976469	1.475788137932
C	2.553882908590	-0.377633087238	0.103733469063
C	1.371797716633	-0.282020720244	-0.648385465061
C	1.225469120178	0.183845645451	3.526613171838
C	0.040215190978	0.448472948909	4.192415521597
C	-1.154436436726	0.476551892322	3.481727533711
C	-1.207218994384	0.227283062173	2.082041358047
C	-2.497575789876	0.019877333661	1.529997701889
C	-3.657711050975	0.705232188315	2.055116963990
C	-4.953867784322	0.107018834298	1.966436805055
C	-5.125905632989	-1.141989682276	1.275335610108
C	-4.112311659130	-1.629699232367	0.533544015186
C	-2.772355766074	-0.976649525562	0.456348440558
C	-3.549928598970	1.987286536213	2.600126436258
N	-4.631105011083	2.597170147851	3.095711152026
C	-5.860660302340	2.025959878747	3.108113219904
C	-6.042498064906	0.786771139222	2.545410810274
H	-4.254925699940	-2.502066802930	-0.097937604658
H	-0.728899048908	0.031816510136	-0.660770318148
H	1.413273415869	-0.354726977754	-1.731421625321
H	3.505821227525	-0.537701565182	-0.393451194968
H	3.402574349810	-0.266813190319	2.074476515000
H	2.158713789803	0.133960725074	4.082092214583
H	0.029528828289	0.587748618187	5.268263808525
H	-2.077651542002	0.562512215460	4.043418185902
H	-7.034979535413	0.351115521984	2.531496089267
H	-6.657963736867	2.607972277089	3.553008921676
H	-4.529302416637	3.539642004375	3.465748819647
H	-2.628458928761	2.556574022789	2.602057899528
H	-2.764044702198	-0.461084576345	-0.525276882472
H	-6.097758344154	-1.624270031814	1.294562671702
H	-1.974043718561	-1.722899654091	0.390392477325

9-Sigma-3’.xyz

35

-787.181442376

C	-0.055195140274	0.080695516581	0.001849206191
C	-0.008358508476	0.004944549933	1.413565889789
C	1.265248759028	-0.024720569127	2.071440862023
C	2.471083332736	0.027511139537	1.303536597912

C	2.374435951506	0.095446714076	-0.069651797677
C	1.115991757505	0.120610701376	-0.718368074762
C	1.278180737344	-0.114049985116	3.471448553195
N	0.137521504805	-0.160379008666	4.152228041641
C	-1.095371534307	-0.127801460647	3.566611664629
C	-1.183423857352	-0.047234108437	2.206440012183
C	3.811014898485	-0.002688870521	1.962219985170
C	4.409120677793	1.227422863241	2.447002108538
C	5.712120666262	1.197549873079	3.080445818732
C	6.381528723863	-0.002433789374	3.198874212828
C	5.818730550311	-1.253876234717	2.705578640606
C	4.484790592653	-1.175420645824	2.083271256095
C	6.303354764719	2.405551829603	3.569289289382
C	5.642468254682	3.600337951475	3.436493406585
C	4.376829571591	3.622607385659	2.809061088787
C	3.772266901933	2.468868054627	2.324805321929
H	6.546317491995	-1.716290402918	2.010176940296
H	5.822703566671	-1.992956606092	3.530111031022
H	2.801777519242	2.539153407935	1.845071392918
H	3.858547547374	4.571382297569	2.699863205875
H	6.081855571129	4.522249688252	3.803016736520
H	7.280502248102	2.352813244754	4.040823363009
H	7.362251686007	-0.033118423564	3.668122160136
H	4.047486239544	-2.100270917709	1.717913923861
H	-2.160384652943	-0.024837296706	1.735913056298
H	-1.945897526968	-0.171806707298	4.233888824511
H	0.185453808398	-0.226582033624	5.165987962273
H	2.189846766327	-0.153182299790	4.055431642142
H	3.280193636065	0.135061034766	-0.667779186922
H	1.084610245531	0.174514084599	-1.802639821662
H	-1.018900504281	0.104622431119	-0.498272979002

9-Sigma-3.xyz

35

-787.144818591

C	-0.100820515249	-0.135900580194	-0.023645379158
C	0.010442769806	0.051713920364	1.353326853278
C	1.320777223091	0.055586579287	1.945714524052
C	2.513341457969	-0.200921823823	1.185004860890
C	2.386797762416	-0.337130411098	-0.174012318298
C	1.084159911356	-0.295723365136	-0.840638109569
C	1.397688720687	0.343748844612	3.326054790206
N	0.288520874162	0.544523854030	4.022341725417
C	-0.976455961639	0.486986389457	3.510817605437
C	-1.135765236119	0.250224149493	2.175127150878
C	3.818090933798	-0.410518296665	1.854715932432
C	4.924732316181	0.464838762158	1.621841156370
C	6.182846135909	0.142761894952	2.226462448079
C	6.291106447079	-1.010449684601	3.047932012599
C	5.201559640506	-1.820887292249	3.277583817184
C	3.961785406710	-1.519659248310	2.679505311664
C	7.294041016072	0.994187342302	2.003354524114
C	7.172570191516	2.127148360126	1.225282102037

C	5.927675861499	2.458259902571	0.649146438340
C	4.825389630487	1.651012364037	0.848592641131
H	0.956690736644	-1.151500182969	-1.534162314597
H	1.084076518552	0.525689849696	-1.594961358812
H	3.869164654530	1.940046636169	0.422152765564
H	5.832459217295	3.361396845698	0.052489517196
H	8.031631772963	2.772614575098	1.063964720161
H	8.246556145298	0.741446106936	2.463120286912
H	7.253376228715	-1.243740691089	3.497497342525
H	5.291974914310	-2.703021139046	3.904514706650
H	3.119745365680	-2.190195957597	2.833003668179
H	-2.130635490850	0.223439896628	1.745311300125
H	-1.786954262261	0.652186353328	4.208151029812
H	0.386755392214	0.764450073076	5.013040383092
H	2.337336264087	0.429943201984	3.857515324460
H	3.262403769061	-0.515813188276	-0.790982665995
H	-1.075398748835	-0.147475483800	-0.503573101621

9-Sigma-4’.xyz

35

-787.186010761

C	-0.047386002309	0.095227893829	0.018360039276
C	-0.046387585416	-0.099114509827	1.421858819110
C	1.190578547054	-0.042877816168	2.141339059096
C	2.411250833219	0.214575697577	1.432238056534
C	2.353912569207	0.430734377858	0.066432920793
C	1.130215479198	0.360418084546	-0.637957195852
C	1.139687056856	-0.180898652234	3.536837319492
N	-0.020569686332	-0.399249456614	4.147480443285
C	-1.214884014672	-0.491421905250	3.496888595795
C	-1.244249475869	-0.337849958124	2.139937637157
C	3.733812191403	0.143373087394	2.084900514380
C	4.646682610274	1.237578055248	2.020476587801
C	5.972895661577	1.062582771947	2.523318114866
C	6.380445052434	-0.236459327472	3.122009479078
C	5.344615107014	-1.267815603022	3.209227921516
C	4.083813918875	-1.076339517440	2.720789321277
C	6.874506579712	2.123267072460	2.450891322959
C	6.481240959934	3.344947963028	1.914853427286
C	5.168768417272	3.540311583734	1.450915956200
C	4.260421640086	2.504627773725	1.504253291666
H	6.807649437774	-0.078110606421	4.127943298758
H	7.238644149806	-0.664654984037	2.571648304365
H	3.240571894476	2.663822750251	1.172120662182
H	4.868632900692	4.508055075542	1.061346456432
H	7.194633657967	4.163048213142	1.868947785496
H	7.887379151022	1.993824700126	2.822576829614
H	5.616400068786	-2.222771843344	3.651093236280
H	3.359706293256	-1.884236183606	2.753306228589
H	-2.192489552709	-0.392726665224	1.616595750577
H	-2.085720860855	-0.674527441923	4.112267296018
H	-0.017337959281	-0.493900686406	5.160711706033
H	2.007941366945	-0.105642294261	4.179737247004

H	3.270273561805	0.609355683344	-0.487832866125
H	1.134084970599	0.512706795779	-1.712801266012
H	-0.986734402276	0.038990059446	-0.523243422595

9-Sigma-4.xyz

35

-787.175581686

C	-0.050204107541	-0.156042321974	-0.067068225285
C	0.006156503479	-0.050259301982	1.414074605378
C	1.256586978964	0.056561784908	2.069267224670
C	2.503956683930	0.035001025945	1.313622413552
C	2.433520367171	0.117656153506	-0.102410923203
C	1.242366380482	-0.006483193700	-0.754721826678
C	1.261290236836	0.292332852185	3.448223871516
N	0.109551619310	0.321242756544	4.122254567036
C	-1.096885308019	0.143984829179	3.539082954617
C	-1.168271414727	-0.031449602689	2.175721032638
C	3.770012391225	-0.150336101425	1.975369474009
C	4.973911705067	0.548022463362	1.584541149526
C	6.219994626137	0.101144229707	2.128239666158
C	6.240698576813	-0.984191859515	3.039756095254
C	5.074830033583	-1.607495415422	3.443037386519
C	3.845870405169	-1.181182857019	2.930554420926
C	7.418201922389	0.774344892612	1.782068894800
C	7.391644231775	1.871882029068	0.947863299714
C	6.159652101488	2.344177307899	0.454433239017
C	4.974664451659	1.704850833289	0.774206554764
H	1.231755026237	-0.055148350199	-1.840206998911
H	4.039668871175	2.123718180101	0.415347052846
H	6.134632949805	3.228752678106	-0.175869271744
H	8.312846784590	2.385328852098	0.688078477747
H	8.356421396345	0.420705546985	2.201679391518
H	7.198537829245	-1.318330913393	3.431027787466
H	5.105815848973	-2.442343686332	4.135764358500
H	2.951270195044	-1.736557196398	3.196157430503
H	-2.139669416622	-0.137211293742	1.705158158423
H	-1.961160077876	0.176523691790	4.190106801548
H	0.142489278879	0.504625004525	5.123873537470
H	2.159040768715	0.495593346114	4.017812227535
H	3.355073623360	0.146285569822	-0.672463777481
H	-0.494511218567	-1.124223831440	-0.360175507012
H	-0.771018060553	0.576142644024	-0.466660789597

9-Sigma-5’.xyz

35

-787.187459788

C	-0.076914270160	-0.361618510416	0.062521347517
C	-0.070511044328	0.047519113327	1.416197241285
C	1.174632069602	0.112849934516	2.124820545992
C	2.396877978269	-0.227338984739	1.460403602835
C	2.335789080241	-0.633653871537	0.142162419826
C	1.105614227237	-0.699091164088	-0.553606124652

C	1.139201385225	0.486065057425	3.476905149309
N	-0.016039684162	0.787721221982	4.060644548843
C	-1.220424557181	0.756915204712	3.418393862481
C	-1.263387004884	0.388514758909	2.104673538702
C	3.711997119871	-0.113672219943	2.147757380184
C	4.487148784224	-1.273627334289	2.475567539406
C	5.777976206211	-1.127501254941	3.067032690588
C	6.268747782322	0.147408134972	3.342961354435
C	5.498947177227	1.262853108132	3.039262893796
C	4.230686402535	1.135344309493	2.446581279127
C	6.576856779630	-2.341087035743	3.389594694969
C	5.966690882977	-3.641404423452	3.099124665641
C	4.710623232588	-3.746908120525	2.562790694458
C	3.989771463690	-2.577726559385	2.265705012335
H	7.558022500389	-2.298972106100	2.881760904400
H	6.873670822146	-2.332619994404	4.454325422608
H	2.989740720207	-2.688624297768	1.854937763998
H	4.262924122654	-4.715289477695	2.367412958779
H	6.545557452611	-4.531498874799	3.331139473044
H	7.249665232420	0.265886469204	3.794400426807
H	5.884755043933	2.255733289871	3.251944744348
H	3.666570031095	2.029617103379	2.197962626905
H	-2.218786076321	0.354625195759	1.592082417381
H	-2.086518661292	1.028052214695	4.007553274660
H	-0.001454352798	1.053610186266	5.042171178363
H	2.022542275994	0.539976133461	4.101101068271
H	3.252015830389	-0.889228822786	-0.382576310433
H	1.105932902247	-1.016267482291	-1.592265776358
H	-1.019027084665	-0.408821827357	-0.475136006088

9-Sigma-5.xyz

35

-787.150196660

C	-0.098643978628	0.031069976096	0.014551789180
C	-0.041438678081	0.062097218643	1.385865411145
C	1.248103150835	0.001812994479	2.032583754452
C	2.476300838712	0.037767584255	1.261638963893
C	2.352267329429	-0.039441355048	-0.125398629118
C	1.096102229836	-0.031886595782	-0.728961030598
C	1.295800317973	-0.247359357381	3.383408571033
N	0.140364964409	-0.286319017776	4.149847015543
C	-1.056924192133	-0.101797717012	3.663946651371
C	-1.266458723880	0.120357439785	2.239179632220
C	3.760774705270	0.240759314988	1.922775663939
C	4.929477544447	-0.536518247526	1.602743282485
C	6.178387477862	-0.155455034030	2.190445323250
C	6.231107079610	0.950043197064	3.079627695824
C	5.093775888159	1.659168481973	3.402858170353
C	3.862478394696	1.302845991344	2.830653994114
C	7.343935373967	-0.906893925709	1.899923802906
C	7.284429630328	-2.011417250753	1.075389429835
C	6.048485109704	-2.413332429504	0.530324350692
C	4.895230264874	-1.697934116895	0.793565653708

H	-1.779993624311	1.098802723486	2.152936358867
H	-2.046483338369	-0.583660663459	1.904384157368
H	3.952219634375	-2.050610671052	0.387973457581
H	5.997190986591	-3.299722184989	-0.095992346663
H	8.183528079745	-2.581703220915	0.859335291095
H	8.286159170186	-0.604313037927	2.350168809564
H	7.190700875418	1.229951573597	3.507480370433
H	5.144945852424	2.508532901314	4.077102449159
H	2.992159871548	1.921399290555	3.031093967070
H	-1.889406811918	-0.122022269780	4.361034273701
H	0.246275258766	-0.465540669985	5.148602364770
H	2.207571999217	-0.465247155350	3.925544851597
H	3.241143191946	-0.017518231130	-0.746671422506
H	1.036886551681	-0.037629223744	-1.813507784697
H	-1.054569244191	0.054027004290	-0.499370999480

9-Sigma-6’.xyz

35

-787.181271104

C	-0.055529286066	-0.107624367140	0.001633716164
C	-0.034121720652	0.031780905168	1.408834223795
C	1.225822090672	0.040844079342	2.093847861790
C	2.444960575775	-0.087669087826	1.355804545551
C	2.371848764709	-0.223371492099	-0.014625021871
C	1.127319511383	-0.233607900355	-0.689197482613
C	1.209942158792	0.166303695409	3.491201163334
N	0.057672971550	0.280554835860	4.143452400680
C	-1.161854305969	0.284170171357	3.529891743939
C	-1.222969778718	0.160494972526	2.171682043013
C	3.773970116117	-0.047843313896	2.035641870462
C	4.495727490165	-1.230191079708	2.319574441683
C	5.800485275497	-1.101065594519	2.937108931734
C	6.332034897749	0.187882990621	3.252156803481
C	5.604214156720	1.313700184357	2.968917448351
C	4.334739487453	1.185538546537	2.362965812626
C	6.527705753800	-2.241717848643	3.216244627759
C	6.028015952165	-3.578308488107	2.914474355656
C	4.692406218065	-3.654539897796	2.296599574868
C	3.978086616337	-2.536652356093	2.021310324762
H	6.069150627545	-4.186371824777	3.839155420185
H	6.781215091139	-4.101745585222	2.293354979515
H	2.995821778187	-2.616396330639	1.567417208271
H	4.293420566432	-4.638347018491	2.068831809451
H	7.509437742905	-2.156111543964	3.676725497813
H	7.313188189953	0.252199211148	3.713360452759
H	5.990478348816	2.301358196385	3.198038143579
H	3.773605804513	2.088378569441	2.134867641816
H	-2.189527790572	0.160066504312	1.679610425974
H	-2.024404076493	0.385550921399	4.175079586995
H	0.086138088000	0.369127272389	5.156165740889
H	2.108103674679	0.173054819455	4.096551758354
H	3.286970927681	-0.318523215994	-0.592143827717
H	1.116467500226	-0.340361200759	-1.769937777869

H	-1.008968190822	-0.114625877861	-0.518276459654

9-Sigma-6.xyz

35

-787.152960015

C	-0.068134278306	0.103754554225	-0.003284798775
C	0.000562436312	0.010701084443	1.410160497972
C	1.285028644992	-0.042335981699	2.070163052277
C	2.465115227968	0.056241762467	1.325686011764
C	2.342008037625	0.105972640914	-0.085616214512
C	1.105875064618	0.131024155460	-0.738663496579
C	1.307175909943	-0.244948714049	3.486525695229
N	0.213606774391	-0.300061491775	4.185025271654
C	-1.114914833149	-0.157192423836	3.634380253399
C	-1.144898472092	-0.026101650294	2.179376718269
C	3.793373444189	0.210187989858	1.946599700018
C	4.861763115783	-0.695293472940	1.646475237201
C	6.159386048519	-0.414740294476	2.181779017836
C	6.344160303274	0.723918108247	3.010554905031
C	5.292390961902	1.560089324628	3.310815909602
C	4.014090881779	1.304441133830	2.775204025844
C	7.232667518398	-1.293400083745	1.887631783389
C	7.036059824048	-2.413052818307	1.106673314101
C	5.750330083510	-2.706966836201	0.604669208312
C	4.683255782752	-1.874563577269	0.878433099583
H	-1.724492389152	-1.025092960377	3.938164753665
H	-1.621853727597	0.707576816067	4.097522669679
H	3.693963378093	-2.145720170048	0.519926103782
H	5.594864477926	-3.603162333554	0.010231599061
H	7.866319795791	-3.079271467306	0.888930391803
H	8.216081194434	-1.072636615732	2.295786288725
H	7.335967480570	0.925739209590	3.408072418366
H	5.442030619941	2.431306769347	3.941549838742
H	3.206711316680	2.006848576724	2.967729365393
H	-2.126876740047	0.041692328626	1.718173723948
H	0.278387033426	-0.477876698848	5.185823595049
H	2.243169909146	-0.388230008727	4.016492371817
H	3.250007686462	0.174360911605	-0.678117906913
H	1.072635878940	0.199001909109	-1.820778679984
H	-1.036793053466	0.153544381577	-0.491153947671

9-Sigma-7'.xyz

35

-787.181721827

C	-0.061218909146	-0.191618090370	0.035561141893
C	-0.056701097341	0.079151652870	1.423755256422
C	1.186290271551	0.074219630460	2.138539648252
C	2.410598803933	-0.191147708662	1.442281824766
C	2.350638387879	-0.466166535768	0.089481722326
C	1.121645057410	-0.465325675281	-0.610309650816
C	1.143865278381	0.299435989584	3.522906607974
N	-0.013875612037	0.538523410478	4.130068865608

C	-1.214921123145	0.580399427084	3.482736624858
C	-1.252036714187	0.349725750676	2.137915302699
C	3.727022592580	-0.118001557537	2.124860119223
C	4.580562497341	-1.282090592216	2.229287041309
C	5.905075867752	-1.156973239159	2.806501844149
C	6.319075293518	0.095680932312	3.284482797765
C	5.469161707314	1.183656373502	3.202961876604
C	4.182030083696	1.082045554497	2.626130177898
C	6.769384673544	-2.299484391355	2.894252617068
C	6.360109468617	-3.521518967332	2.478543918493
C	5.005201792236	-3.719390579170	1.934854847225
C	4.154284415067	-2.536335921426	1.841461321125
H	5.046277098151	-4.192426127071	0.933922318544
H	4.462213626322	-4.497715672738	2.505562224486
H	3.147643427687	-2.678396743212	1.458018499563
H	7.015208415910	-4.384914078093	2.543352508877
H	7.762322548417	-2.156735846324	3.310737912172
H	7.308158036922	0.204023738854	3.719018254331
H	5.798904264461	2.149644886175	3.574920148681
H	3.567527491173	1.973972257458	2.547999491767
H	-2.204440570175	0.369579301658	1.619281724475
H	-2.083274914480	0.791542867890	4.092686328102
H	-0.003737304044	0.694884638627	5.135140413240
H	2.023102629123	0.283037234322	4.154827593970
H	3.268231915129	-0.655621108038	-0.460145148688
H	1.124064716197	-0.674976800514	-1.675797391371
H	-1.003022045372	-0.183012435206	-0.504739838124

9-Sigma-8’.xyz

35

-787.187475206

C	-0.081283654373	-0.231810386478	0.047524942241
C	-0.051805166559	0.049899568627	1.432737083437
C	1.209026180809	0.081931529862	2.115346095199
C	2.422375835191	-0.163064782116	1.396632434424
C	2.340972038832	-0.430016023820	0.045797961358
C	1.095370459093	-0.466708336051	-0.625433500906
C	1.201075400911	0.354233419834	3.491805232999
N	0.055487056943	0.582258271710	4.125452064477
C	-1.164768061694	0.569423524896	3.512830844291
C	-1.233512330320	0.304447011475	2.175409720787
C	3.750144238035	-0.095730065340	2.076114500009
C	4.456103315140	-1.254634800312	2.431951151056
C	5.727945418171	-1.120060325434	3.063951453535
C	6.275570932472	0.161059545097	3.324606523317
C	5.570630878507	1.289536668999	2.965392305037
C	4.317955976547	1.155180785316	2.347028588405
C	6.440782442589	-2.280587142902	3.435766402208
C	5.968207529129	-3.584962523971	3.220476556843
C	4.750596505415	-3.754688783492	2.615701500896
C	3.922580826929	-2.623373060351	2.181788478669
H	2.921466896811	-2.744972589740	2.633364453212
H	3.702347303363	-2.769442257909	1.108981593202

H	4.359161060324	-4.752535618777	2.435609021817
H	6.565062226563	-4.435359285061	3.531454033711
H	7.407957054729	-2.149826684374	3.916651513408
H	7.246521838398	0.236709875197	3.805541023926
H	5.974212278957	2.279117918169	3.154185538333
H	3.770012310733	2.049979358066	2.062860512736
H	-2.200356240806	0.289824970721	1.683992229123
H	-2.020967088909	0.773444304605	4.142065648708
H	0.090735034109	0.777409526238	5.122790872047
H	2.100164903296	0.389220295819	4.094885597267
H	3.250795421588	-0.601717952320	-0.522793511189
H	1.078494054135	-0.678505161656	-1.690522367224
H	-1.035189728251	-0.256798164792	-0.470811848479

9-Sigma-8.xyz

35

-787.137010586

C	-0.026335039928	0.117721972042	-0.040935320486
C	-0.001439835424	0.036902643777	1.392405240809
C	1.254464225707	0.061596273296	2.107383474159
C	2.443873289121	0.068567698969	1.391968694263
C	2.374889003024	0.101967280759	-0.015135868055
C	1.155898202791	0.155745438053	-0.728737979546
C	1.244183826569	0.066621322901	3.596972968751
N	-0.042066741163	-0.245876436506	4.180054721917
C	-1.149575581624	-0.357838876370	3.518487025788
C	-1.168532439242	-0.183478930644	2.093358410005
C	3.780498998074	0.091392870024	2.040565043243
C	4.648516083307	-1.038652042936	1.924888342607
C	5.968972333217	-0.943902001444	2.469633941766
C	6.370998336160	0.249167521811	3.122642162597
C	5.506932890283	1.319461388779	3.235460979568
C	4.211954542445	1.240307093070	2.689966990211
C	6.846856192277	-2.050390640702	2.347029959873
C	6.442353112751	-3.210260070310	1.713429403303
C	5.138394028972	-3.309307938023	1.190153122320
C	4.253464471660	-2.250812745258	1.302534331864
H	1.991264659404	-0.631207837255	4.004912179152
H	1.521047475271	1.054056029519	3.999133127299
H	3.239887378545	-2.364335647446	0.929002712227
H	4.818724584631	-4.227776218937	0.705756489171
H	7.126036527142	-4.049930987866	1.625822691062
H	7.848930216370	-1.972266536560	2.761905928087
H	7.375337913096	0.309618565237	3.534623967992
H	5.823657122515	2.232077458168	3.731454651668
H	3.556237928821	2.105070206983	2.750268805262
H	-2.125422105045	-0.264307991161	1.588509926696
H	-2.055448588697	-0.577498874292	4.073602279661
H	-0.065503631423	-0.339781655650	5.196138575692
H	3.306743486000	0.106289291323	-0.574476189904
H	1.171375062629	0.200494214988	-1.812275138021
H	-0.984372048692	0.120190668647	-0.551043577852

25-Sigma-1-a1.xyz

51

-1155.26281565

C	-1.992707068486	2.076354779465	1.260015462068
C	-0.942384967400	1.260040136260	1.671526008096
C	0.399163502676	1.698078565092	1.446923549037
C	0.646601602377	2.946166175402	0.815527711485
C	-0.410176573308	3.734425533372	0.413271065158
C	-1.727736429892	3.293517391604	0.635648899703
C	1.471732369009	0.870371831218	1.830137280938
C	1.294204587985	-0.397267665643	2.411115536274
C	0.024383766655	-0.852315045680	2.649647540735
C	-1.181210627309	-0.029580421579	2.404922389012
C	-2.405004305499	-0.834528283282	1.978291029365
C	-2.456111042095	-1.464928920849	0.695987053007
C	-3.621768523691	-2.221701261378	0.338004746176
C	-4.713107500358	-2.326658603981	1.260440838704
C	-4.612004815205	-1.711641956878	2.491284568254
C	-3.465164898188	-0.969513091692	2.847368654912
C	-1.391722588227	-1.378133156930	-0.239983668194
C	-2.599193759835	-2.765352975790	-1.808351867583
C	-3.652979029341	-2.865895126986	-0.927655983384
H	-0.133666944889	-1.827771705211	3.103257425258
H	-3.019602665293	1.760786646752	1.418529275071
H	-2.559159839813	3.913877681599	0.312179916937
H	-0.232096042484	4.688690839793	-0.072463051142
H	1.673466422164	3.263890800491	0.657353878671
H	2.484421918229	1.226091002197	1.652105264395
H	2.158508592132	-1.004548352875	2.657977950169
H	-4.528100598799	-3.450957511888	-1.195437677382
H	-2.644354809401	-3.267534487138	-2.771233477694
H	-0.503737308940	-0.801332861843	0.004363605482
H	-3.431133377836	-0.495567230764	3.825766483439
H	-5.433040075391	-1.787443546500	3.199479538831
H	-1.406508107592	0.298792757750	3.446216729718
C	-1.457139885793	-2.010318180946	-1.461765999605
H	-0.627768554036	-1.928373451975	-2.159318098105
C	-5.941447910280	-3.105729135511	0.925243480053
C	-6.948248230902	-2.564199734268	0.061677668888
C	-6.122704312471	-4.359517521622	1.479126327015
C	-6.845457830104	-1.269917646358	-0.517359661349
C	-8.114015387745	-3.347890964188	-0.222770454069
C	-7.275753073571	-5.128740530150	1.197818752837
H	-5.357956942904	-4.765699880549	2.137147170495
C	-7.835201940875	-0.779332627860	-1.340856891879
H	-5.972545534447	-0.660054367138	-0.300832961780
C	-9.115096148324	-2.813667213595	-1.078347211849
C	-8.249360563053	-4.635438003597	0.360725268050
H	-7.383596786853	-6.113251163013	1.646047852839
C	-8.981059338021	-1.558503766329	-1.628192137573
H	-7.737455321754	0.213982880057	-1.772083672033
H	-9.995707479266	-3.416804927205	-1.290022564857
H	-9.137200949574	-5.223457628945	0.137733326554

H	-9.755094671981	-1.161498070918	-2.280186869147

25-Sigma-1-a2.xyz
51
-1155.26276202

C	0.047650779100	-2.067615643850	3.235345198301
C	-0.182787433757	-0.694369783881	3.234800765879
C	0.530290890442	0.123686095843	4.164420917526
C	1.455726925613	-0.462574894820	5.068781036330
C	1.662524420225	-1.825237643339	5.053312804020
C	0.953864604114	-2.623115172960	4.136280692307
C	0.286200615515	1.509817882393	4.195806580478
C	-0.653908128078	2.143560164181	3.364863219922
C	-1.357408854512	1.395967590865	2.457377492692
C	-1.106690477255	-0.046577436511	2.241793305278
C	-2.346710895565	-0.848079253878	1.858346504601
C	-3.406824411184	-1.049062293132	2.796378741102
C	-4.557073749113	-1.808195969585	2.395820093380
C	-4.628055220675	-2.345363174548	1.069341883112
C	-3.576394924928	-2.131769720866	0.202280046406
C	-2.443880064405	-1.385619087801	0.593829327069
C	-3.363598519967	-0.532752762237	4.118339104924
C	-5.522745903430	-1.498916760282	4.613856092100
C	-5.597834714886	-2.016256075741	3.339881212974
H	-2.087216557605	1.868387147862	1.804300893227
H	-0.486089731314	-2.707856987976	2.539415312909
H	1.113837423198	-3.697835026532	4.130615831617
H	2.365017068959	-2.283938797433	5.742017366677
H	1.990643286103	0.176688790145	5.765658568187
H	0.841978939516	2.113142956063	4.910518247028
H	-0.821125400485	3.211647454797	3.454220516342
H	-6.465035733922	-2.598693699433	3.042325811246
H	-6.330642991818	-1.671649896999	5.320101894865
H	-2.499878786289	0.039346504393	4.446202850285
H	-1.639540280762	-1.238674348026	-0.123436358730
H	-3.619151626118	-2.537606114034	-0.805131355565
H	-0.498859264062	0.001168768246	1.308249846513
C	-4.394205894317	-0.746194801011	5.006461447328
H	-4.335412414357	-0.339811407047	6.012710804111
C	-5.798754878576	-3.154783853659	0.619037626451
C	-7.031045115489	-2.530780169725	0.239596894130
C	-5.680168932099	-4.530111877446	0.540243862664
C	-7.214653032210	-1.121318881108	0.263129473576
C	-8.120731932679	-3.353719996867	-0.195919322498
C	-6.757220021345	-5.338579356907	0.108168783986
H	-4.741725710447	-5.000090982215	0.825206828899
C	-8.412875003960	-0.554779851173	-0.113512648242
H	-6.392625962292	-0.485198482324	0.580233555388
C	-9.344118255283	-2.737981881850	-0.574735169714
C	-7.954490116704	-4.763029792636	-0.248478919725
H	-6.630850336164	-6.417341440106	0.063460674768
C	-9.490822553558	-1.369558330886	-0.534101337168
H	-8.531994454548	0.525678443305	-0.089770986598

H	-10.166512882006	-3.370690808403	-0.902418149103
H	-8.788223257512	-5.378927980470	-0.579047704751
H	-10.431850529111	-0.911431360395	-0.828180156503

25-Sigma-1-b1.xyz

51

-1155.26150802

C	0.222734101285	0.041384510292	1.260424121213
C	1.418587907943	-0.006200294849	2.035031625038
C	2.682346924355	0.169532790608	1.412054909119
C	2.754312005208	0.385856404954	0.051728207199
C	1.333385388076	-0.216641978014	3.426306409254
C	0.115620347251	-0.396592883973	4.104358689394
C	-1.054029827293	-0.381482393063	3.391874966507
C	-1.105443623858	-0.179953191904	1.926849362987
C	-2.167433496922	0.882174366078	1.599803909879
C	-3.466073213359	0.537455116142	1.111509324121
C	-4.404144605913	1.595683948145	0.844561723725
C	-4.036825522499	2.957252525623	1.090592305619
C	-2.771047172346	3.235276918608	1.566669302938
C	-1.846037105757	2.205059405897	1.823524306990
C	-3.878659214448	-0.800008099265	0.859031633114
C	-6.040246413037	-0.044788543872	0.080133847238
C	-5.681236328605	1.262090345462	0.318984211844
H	-2.004951068627	-0.530232894196	3.897644073345
H	-0.578417826188	0.305239393319	-0.719512856511
H	3.715115984251	0.521537915665	-0.434801046167
H	3.581868832002	0.127880116277	2.019836552210
H	2.258038724787	-0.240181670683	3.999492318089
H	0.112498627065	-0.547421861704	5.178584404691
H	-6.380376402157	2.064046788284	0.100696740176
H	-7.023015609251	-0.276084328257	-0.322260439847
H	-3.207936239455	-1.629514983429	1.063945666627
H	-0.858253626559	2.469663293499	2.193380891237
H	-2.480596274816	4.265941709668	1.752941155378
H	-1.468750871874	-1.147226651100	1.533668276710
C	-5.128862993275	-1.086518371954	0.357845643443
H	-5.412050238601	-2.119490080284	0.173688471771
C	-4.969455885817	4.086542962996	0.802491329496
C	-6.062471799215	4.398565669231	1.674538757091
C	-4.745397782394	4.870580973717	-0.314508813147
C	-6.333166144605	3.661407984016	2.859582282616
C	-6.910429219041	5.508735399098	1.353153472926
C	-5.583711935512	5.965494844058	-0.627091636928
H	-3.911094884893	4.634098037758	-0.970791785366
C	-7.390840549990	3.994633717165	3.677504737981
H	-5.690286365162	2.825253021764	3.121394464924
C	-7.993886088385	5.824223638172	2.216737273186
C	-6.647422646515	6.276079196363	0.187625799494
H	-5.381227348069	6.556728431181	-1.516562374380
C	-8.233021526038	5.084729872161	3.353118006761
H	-7.579453020813	3.418239392801	4.579942481148
H	-8.633056099636	6.667756577277	1.963993401182

H	-7.298425738582	7.115701586332	-0.046908953294
H	-9.064887768066	5.337790571609	4.005690460929
C	1.569830642917	0.430843668596	-0.704909639351
C	0.320031348507	0.260703562941	-0.110512653762
H	1.627251643926	0.604153570792	-1.776075318837

25-Sigma-1-b2.xyz

51

-1155.26152517

C	1.311084447334	0.032303698884	2.271983802758
C	2.626904099114	0.293572742784	1.806431237114
C	2.837956049843	0.602756942107	0.478950353297
C	1.742543165222	0.656865230033	-0.401096408335
C	1.082763889324	-0.268446672496	3.630060806549
C	-0.193836700630	-0.532188514551	4.155683014386
C	-1.277214938433	-0.515424835090	3.317760609668
C	-1.175491087809	-0.228643950310	1.869766563601
C	-2.247275325916	0.801655352674	1.480806843737
C	-3.460285590132	0.432190724625	0.820880238586
C	-4.416059953406	1.462909903600	0.512083240853
C	-4.146095096282	2.821701545998	0.872099143196
C	-2.969066826838	3.121505200839	1.528159268851
C	-2.028965423017	2.117988976637	1.831985870029
C	-3.778151161675	-0.904552279701	0.453855757608
C	-5.897257373238	-0.203875803679	-0.478028054136
C	-5.627033844647	1.101747980822	-0.137220277932
H	-2.270692800435	-0.730754858302	3.703416275178
H	-0.383834249551	0.456758384466	-0.663843897139
H	1.909143233016	0.904047248691	-1.445995835873
H	3.839602257366	0.804401190824	0.112512400833
H	3.455981122461	0.243910033589	2.506940712553
H	1.939249116107	-0.295324499005	4.300550976232
H	-0.309002403788	-0.749163456984	5.212263466848
H	-6.350101452005	1.880376639752	-0.362098464420
H	-6.831431951486	-0.456932034724	-0.972479729530
H	-3.090249818871	-1.714496278972	0.678698089883
H	-1.110126617141	2.398745610832	2.341404619332
H	-2.756549967068	4.149300369109	1.810702542736
H	-1.440689160045	-1.188921903560	1.389385744133
C	-4.959593239400	-1.216550455860	-0.181094192673
H	-5.171454617796	-2.248389181987	-0.448687698328
C	-5.123309085911	3.914064978812	0.588393378652
C	-5.246954533899	4.480681201702	-0.721465970148
C	-5.899838633764	4.409083620955	1.619849770245
C	-4.460206729266	4.044557650993	-1.822271914223
C	-6.189608739795	5.539494057791	-0.933231862845
C	-6.827951758867	5.454735551645	1.406902568917
H	-5.799950296604	3.979051202706	2.613753064451
C	-4.602832420007	4.616185361234	-3.067847241714
H	-3.734041080310	3.250202571502	-1.672110175370
C	-6.313389930102	6.105949059696	-2.230568188085
C	-6.972805144890	6.006490177166	0.155325904829
H	-7.425679885036	5.816207774208	2.239827670409

C	-5.540368622888	5.655500891532	-3.277038079572
H	-3.990634575380	4.268501161909	-3.896381023090
H	-7.032345776052	6.908458192702	-2.382399931511
H	-7.685838808603	6.810062510814	-0.016633582927
H	-5.644968045927	6.098260774031	-4.264419414498
C	0.206293225666	0.088345688659	1.372608604262
C	0.443663061458	0.403094520899	0.037869402621

25-Sigma-1’-a1.xyz

51

-1155.26769825

C	-0.015746724495	-0.705206720456	-0.180037981497
C	0.043648505340	-0.153449790737	1.098859795623
C	1.317596083602	0.112103529816	1.684321343676
C	2.488882404723	-0.123289585410	0.917367798746
C	2.405398468601	-0.664052046800	-0.350579508058
C	1.149279706347	-0.972269309500	-0.892971593989
C	1.396357630251	0.645046440344	3.015139981239
C	0.206104076128	1.011810247285	3.707742388704
C	-1.022398691048	0.764139972011	3.178033576685
C	-1.225680353982	0.234884533808	1.810387681235
C	-2.386668705890	-0.753383327577	1.705920873490
C	-2.318460696351	-2.044657287205	2.319798980669
C	-3.446550626974	-2.922124870343	2.199338403921
C	-4.594595288061	-2.494752987799	1.480946103335
C	-4.633251687867	-1.249127239171	0.901998363073
C	-3.523954021986	-0.379918938345	1.018564292503
C	-1.186884825872	-2.504379866378	3.044585140425
C	-2.282918450460	-4.615995742770	3.505122789925
C	-3.395790874174	-4.205006318311	2.806686722230
H	-1.914915486722	0.980759215472	3.760139623582
H	-0.983672899675	-0.921572385700	-0.623365263384
H	1.080881308647	-1.402883593721	-1.888235728511
H	3.307580816820	-0.835384554799	-0.929831216744
H	3.456125691987	0.153086302798	1.322361609053
H	0.289094937806	1.401827073132	4.716802926422
H	-4.258381755727	-4.860790272426	2.709769938253
H	-2.257200993631	-5.600397127193	3.965182377576
H	-0.314852256634	-1.865875488642	3.147843123575
H	-3.574600100967	0.602173810016	0.553236041376
H	-5.511584063021	-0.922692071701	0.351757703438
H	-5.443087343563	-3.170158402870	1.397153803054
H	-1.573313479888	1.136799288135	1.263174435135
C	2.662899511014	0.730060829245	3.737296101333
C	3.054086392514	1.907791493625	4.468278134562
C	3.438023936015	-0.427891303052	3.825511723822
C	2.401236162418	3.158509230232	4.335370870462
C	4.207539892973	1.830943074087	5.312734978639
C	4.560289653373	-0.491100368090	4.669159425677
H	3.125759231626	-1.322758311113	3.295572675390
C	2.831749932182	4.261368747816	5.045598276152
H	1.564216429984	3.264836525048	3.651491836909
C	4.625145985889	2.981536498747	6.029993266427

C	4.931954940679	0.613849472650	5.403624582436
H	5.118098835402	-1.419497698387	4.746803858450
C	3.945944368706	4.173750607778	5.907770573813
H	2.313016365692	5.209249842012	4.929812850307
H	5.501819177126	2.908047025260	6.669079212960
H	5.796638718520	0.568425701342	6.061542011081
H	4.274255934746	5.050419994348	6.459325492325
C	-1.168046575409	-3.754621277514	3.623514593341
H	-0.289069196715	-4.081182568998	4.173305011156

25-Sigma-1’-a2.xyz

51

-1155.26808527

C	-0.219081999950	-0.166436225381	0.191912907012
C	-0.020657445990	-0.146247693731	1.571532990033
C	1.310647232754	-0.153063219021	2.082021097700
C	2.397235712201	-0.252589744222	1.174001470971
C	2.175680400703	-0.286264374375	-0.189195848837
C	0.863946906918	-0.228117812908	-0.680583421162
C	1.533658673335	-0.139074070211	3.501659399604
C	0.433783107068	-0.314047430945	4.391934054961
C	-0.846648527490	-0.288937263911	3.934790855821
C	-1.194386064432	-0.060202006820	2.511037809142
C	-2.456160800697	-0.793465095798	2.063805815119
C	-2.493816217121	-2.222332246668	1.992662458491
C	-3.710170322334	-2.856935484262	1.575246361283
C	-4.839527888509	-2.061204330609	1.245783634818
C	-4.775768319760	-0.690676358536	1.324250872026
C	-3.578494149860	-0.060421275484	1.735369193478
C	-1.382858246676	-3.047141940642	2.313747711259
C	-2.667647766974	-5.042920700713	1.824331278975
C	-3.763625843215	-4.274276531644	1.502539827188
H	-1.672865146140	-0.332244969235	4.640311957799
H	-1.230781900791	-0.147947622170	-0.202550089472
H	0.686356870891	-0.255062513284	-1.752241278686
H	3.013152236081	-0.372242602772	-0.874793022571
H	3.408107416711	-0.334511534917	1.557795435979
H	0.624381603061	-0.364341096784	5.458593078968
H	-4.692214268000	-4.744307244779	1.186024933589
H	-2.721701866161	-6.126825629329	1.764153851216
H	-0.445647948955	-2.595006697950	2.626484826162
H	-3.547804630754	1.025626214015	1.789723826338
H	-5.639806109726	-0.082172456641	1.071297819569
H	-5.755935083338	-2.555142264533	0.930588768756
H	-1.477256709418	1.013860206385	2.518702206633
C	2.851005459298	0.133207297186	4.063798071544
C	3.410578441011	-0.624029808301	5.154629827808
C	3.514599849481	1.272880792726	3.601950584591
C	2.885338222767	-1.864634211711	5.593026783753
C	4.606226153460	-0.137151212992	5.774207590090
C	4.681505869580	1.742473073262	4.227012579714
H	3.077772488295	1.853696536008	2.795307520629
C	3.475668943825	-2.556515403791	6.632281618597

H	2.022180204721	-2.296029073841	5.094880638752
C	5.187969630446	-0.869587383901	6.840652586967
C	5.210495061390	1.055714122235	5.298176601116
H	5.149170277358	2.654910106287	3.869487973650
C	4.629248743319	-2.053094504831	7.270649328419
H	3.052183938669	-3.504482344418	6.953271749220
H	6.093415552177	-0.485570533000	7.304434516549
H	6.109934677438	1.417571812979	5.790731952940
H	5.082726080039	-2.608595820362	8.086968416342
C	-1.465968290193	-4.420294066311	2.234017480725
H	-0.601144206514	-5.028797359355	2.485573396429

25-Sigma-1’-b1.xyz

51

-1155.26704849

C	0.213174862125	0.097127346281	1.246740935555
C	1.398101912462	0.185469199182	2.030665435946
C	2.646886922386	0.353644104701	1.376934380078
C	2.724926653312	0.363897888668	-0.002493091348
C	1.557039558037	0.218449941373	-0.764259986400
C	1.308172263766	0.173006382122	3.465622943393
C	0.032098169177	0.217889954032	4.098784598146
C	-1.112824118896	0.095243130873	3.375413953915
C	-1.137385083534	-0.023633722778	1.899123207386
C	-2.194929010017	0.933632158274	1.330341277786
C	-3.464552647912	0.469488351939	0.861094006352
C	-4.400377243027	1.441701066007	0.365616291206
C	-4.055013573028	2.817699037040	0.365535951974
C	-2.831126065392	3.232910491670	0.835431190635
C	-1.905156508590	2.284980293626	1.319905412996
C	-3.860946435052	-0.896940057378	0.849000017981
C	-6.008365065505	-0.321738643995	-0.119771778512
C	-5.663724707364	1.010185529340	-0.119961469209
H	-2.069551551278	0.033295358786	3.888254103162
H	-0.583157128528	0.020198846710	-0.747142651930
H	1.614227442090	0.223673198729	-1.849344124032
H	3.684341675454	0.499178807163	-0.492531986290
H	3.543983301639	0.504124089217	1.967113239501
H	-0.014157680127	0.240413101918	5.182372883086
H	-6.358224851617	1.758764311344	-0.495179878707
H	-6.978538410673	-0.638228328229	-0.493797986041
H	-3.194202372671	-1.667402822519	1.225248670300
H	-0.939418389866	2.630498623187	1.682619948413
H	-2.568026712732	4.287235232124	0.836946793170
H	-4.776155737047	3.539138600643	-0.012137635197
H	-1.477029990758	-1.055170356372	1.705222411642
C	-5.094500572780	-1.282041327650	0.371071585808
H	-5.366693056108	-2.334377646762	0.373852737413
C	2.491103424804	0.018252243449	4.304709729049
C	2.725212917483	0.822113334757	5.477303517719
C	3.341577226068	-1.052733327616	4.020049840580
C	1.998458993137	2.002602452060	5.770046641579
C	3.799363379807	0.453216037016	6.349032253680

C	4.382794742924	-1.409843815893	4.892946104326
H	3.146831487365	-1.672572645807	3.150050664852
C	2.280675173497	2.746968639707	6.898444024867
H	1.223190502850	2.345796265787	5.091393037698
C	4.064347630196	1.237676810327	7.500860163781
C	4.601503780267	-0.675794186469	6.038449246838
H	4.999091499450	-2.274033271815	4.663910349133
C	3.314006497760	2.359273388902	7.778122084540
H	1.707139679739	3.646931885590	7.103810599406
H	4.881743847009	0.944534447165	8.155266174571
H	5.403110905342	-0.950395113223	6.720081094602
H	3.525313714773	2.955516272292	8.661529680571
C	0.316638749583	0.099178444505	-0.143296595968

25-Sigma-1'-b2.xyz

51

-1155.26514125

C	0.305215461425	0.198291161513	0.058312136844
C	0.136253635159	-0.021242536836	1.425516696548
C	1.285546466863	-0.125855508989	2.258874704517
C	2.571038647461	0.068028941941	1.690238379615
C	2.714585338113	0.303019995930	0.338008775029
C	1.577301049152	0.350831637612	-0.483459173172
C	1.124690433171	-0.342382105415	3.670722356973
C	-0.173247495751	-0.296928354628	4.252188366790
C	-1.289580448108	-0.243056285991	3.474650948318
C	-1.246929651678	-0.168751265821	1.997715069135
C	-2.246627705508	0.888381457632	1.509395434916
C	-3.478005614115	0.535303513371	0.872608544733
C	-4.359471202064	1.596634607363	0.468705523247
C	-4.000308665947	2.946201888603	0.717955161605
C	-2.813333384721	3.252069446098	1.341323264647
C	-1.940326231576	2.216903428862	1.737251741885
C	-3.885670114853	-0.801412993664	0.606513939057
C	-5.940880955607	-0.026881971118	-0.421898107453
C	-5.583636338514	1.279166129221	-0.178178196984
H	-2.272335557435	-0.303634012218	3.935651516595
H	-0.567649842081	0.268256457409	-0.584590129100
H	1.687410640625	0.529851260428	-1.549504557413
H	3.701758147072	0.457653382193	-0.086775930131
H	3.446391676154	0.047894265088	2.329897575874
H	-0.267920033469	-0.408955684503	5.327516375089
H	-6.237115444576	2.094908196803	-0.479810976133
H	-6.880692453448	-0.256029988154	-0.917370721700
H	-3.259446228399	-1.638421970352	0.901661964147
H	-1.001709197891	2.476275731381	2.222251872565
H	-2.539142684386	4.286011882221	1.532433862704
H	-4.681304497318	3.735315584267	0.407141244037
H	-1.606923800775	-1.157389723607	1.662498720934
C	-5.079773403365	-1.075069564676	-0.023956956525
H	-5.361982198750	-2.107259728277	-0.214648922140
C	2.252335553402	-0.581733407692	4.573130522518
C	3.233719938411	-1.616176974133	4.365849568923

C	2.350107801104	0.251178630549	5.687110709386
C	3.120368801672	-2.614480664858	3.364634291400
C	4.337607869749	-1.691735143846	5.275541795882
C	3.452480669992	0.180616178678	6.555672061207
H	1.597168797709	1.017207149375	5.851597493031
C	4.074950264710	-3.601425710475	3.236727917450
H	2.266722244955	-2.615639762731	2.694029383872
C	5.309638541242	-2.713766775029	5.110355585882
C	4.431950697108	-0.766281122079	6.346380248653
H	3.525826232528	0.877884838947	7.384891081108
C	5.187108662110	-3.647754890220	4.106902403332
H	3.965663644162	-4.355331211992	2.461583137381
H	6.146366594781	-2.753771516356	5.803749388605
H	5.285687358645	-0.828271250902	7.017058031963
H	5.933117982860	-4.429245640925	3.990945874350

25-Sigma-2’.xyz
51
-1155.26710758

C	0.211009292892	0.199728171965	-0.001498852880
C	0.038387384014	-0.363312481504	1.303430637350
C	1.102936237320	-0.980578690177	1.932683391776
C	2.373320494290	-1.057305012580	1.316449852754
C	2.569515316316	-0.512460970716	0.068915283778
C	1.501907451870	0.123025541601	-0.619077401600
C	1.682812638232	0.679934667292	-1.913506603594
C	0.638093360167	1.283566758062	-2.576955710309
C	-0.639502623509	1.350772201863	-1.972680021348
C	-0.847768963762	0.821224399186	-0.717679024425
H	-1.837245503644	0.871908141191	-0.271119942213
H	-1.462824042198	1.821257083582	-2.504308287067
H	0.790005790921	1.705381187904	-3.567255513971
H	2.666295659421	0.618981217445	-2.374790073324
H	3.545326243583	-0.564003626668	-0.409254628317
H	3.191424220694	-1.546697169303	1.838333173412
H	0.962470806197	-1.407015023454	2.923014952969
C	-1.282683393580	-0.316405678752	1.996495096155
C	-2.062230588532	-1.422455817400	2.057201390864
C	-3.337186932130	-1.455278692135	2.795503084488
C	-3.835083997077	-0.184309988060	3.368221065508
C	-3.037683499027	0.966632471942	3.282365474081
C	-1.744786334369	0.904854275829	2.632832631945
C	-0.955960083578	2.065206864785	2.621771808610
C	-1.417847057119	3.249642416029	3.176449064371
C	-2.702469985858	3.337146715300	3.750378420419
C	-3.498890676768	2.218901356666	3.797133992758
H	-4.497397454467	2.279164067620	4.215280488549
H	-3.060359849827	4.283573424236	4.143177488600
H	-0.783737636435	4.131606256967	3.148140548415
H	0.027478933049	2.035438121859	2.164489960530
C	-5.090288274272	-0.228859028550	4.126518891703
C	-5.029289999757	0.050850407867	5.488246083208
C	-6.142778679744	-0.159720138573	6.324570927142

C	-7.325054977256	-0.623482950688	5.793738870423
C	-7.453245632141	-0.869381895206	4.401391998731
C	-6.328290874569	-0.665911553629	3.539378152316
C	-6.513195024668	-0.825733047718	2.141279840248
C	-7.729168929325	-1.225581615424	1.628942600567
C	-8.825289756945	-1.473048670677	2.485830966994
C	-8.689682508376	-1.288322865502	3.843370203610
H	-9.534575208339	-1.446524121181	4.509532855226
H	-9.777125114866	-1.790427823666	2.068584535536
H	-7.847558194564	-1.340193033648	0.554750435049
H	-5.700444671757	-0.602079867651	1.455839708053
H	-8.185037091537	-0.796100624552	6.436571762241
H	-6.052842044398	0.033175088914	7.389561561244
H	-4.088185809696	0.369518266361	5.927696937466
H	-3.202374326526	-2.128617763869	3.666782012869
H	-4.119169544230	-1.984570415000	2.233661704042
H	-1.745262146090	-2.345318712283	1.579081998991

25-Sigma-2.xyz

51

-1155.26762775

C	0.361994876870	-0.096111182075	-0.258986097407
C	0.014612797810	-0.316600490057	1.113030069835
C	0.966264619840	-0.806426319173	1.989597124806
C	2.286675724969	-1.076021533491	1.565109491593
C	2.649297128773	-0.858816796144	0.255807704903
C	1.703544210518	-0.371565907249	-0.683733037902
C	2.056835439717	-0.156457475616	-2.043225237837
C	1.129761787452	0.300172151886	-2.952591116315
C	-0.198579763303	0.558344946274	-2.538734984517
C	-0.572310381593	0.364637691303	-1.226770475489
H	-1.596828245732	0.562498711962	-0.924187932216
H	-0.929713791056	0.909455229762	-3.262734387584
H	1.413236451715	0.458817699788	-3.990025592865
H	3.078293008254	-0.364459382046	-2.355080039115
H	3.664823372362	-1.061181252477	-0.077798523948
H	3.012164592019	-1.451140551087	2.282286432358
H	0.696800099826	-0.973561725720	3.029888605258
C	-1.346245947482	0.008184572066	1.627230207609
C	-1.718842232073	1.332246420651	1.776390226523
C	-2.961559751993	1.683797104980	2.328635103775
C	-3.851783319413	0.710495120353	2.767219219643
C	-3.538881143850	-0.677100332674	2.562354973697
C	-2.270813002301	-1.024573962663	1.990460701575
C	-1.981381826376	-2.395502892473	1.756519290739
C	-2.896938469776	-3.379687447836	2.055096453802
C	-4.158705019484	-3.033456983116	2.583746235130
C	-4.473517405873	-1.713272248288	2.821476637028
H	-5.468715079475	-1.466212414251	3.180209317518
H	-4.892432864914	-3.808296386548	2.789096786693
H	-2.657240628228	-4.422007614066	1.862785214348
H	-1.024490428904	-2.660401950014	1.317490263667
C	-5.020427185139	1.151352274514	3.535184940371

C	-5.110697336723	0.716650276455	4.951740439799
C	-6.270316817260	1.176452286235	5.741067854807
C	-7.151055477695	2.065533097971	5.235658608654
C	-7.026272240582	2.535441541545	3.882653522358
C	-5.965581658928	2.055330915666	3.024536734340
C	-5.941380094451	2.471531924632	1.656594015167
C	-6.870354089653	3.363672824364	1.177955248509
C	-7.871733933252	3.863003155500	2.038823731641
C	-7.954168726241	3.448792697459	3.358932604791
H	-8.748104874723	3.823089581729	3.998829434099
H	-8.603825557656	4.567486966589	1.653442045289
H	-6.846522452935	3.673953721859	0.138060747067
H	-5.189717540657	2.060094236768	0.992204476569
H	-7.983341006663	2.429998404343	5.831186826643
H	-6.366008956065	0.801922115558	6.756040831710
H	-4.938667493840	-0.362826306386	5.059830634598
H	-4.199660281655	1.130944205172	5.430855909320
H	-3.187993074805	2.734061549466	2.489759950641
H	-1.026100188188	2.120877748902	1.495825230111

25-Sigma-3.xyz

51

-1155.26542833

C	0.176924582104	0.026548373400	-0.006766842381
C	0.133005392876	-0.191132136288	1.408725862499
C	1.289072301885	-0.549772084445	2.077083278095
C	2.513960076612	-0.721317814997	1.391083451679
C	2.577852014903	-0.528608388927	0.030640752833
C	1.419864272126	-0.149304957364	-0.698582196158
C	1.467459827344	0.069264491207	-2.102007023457
C	0.342350969950	0.450129797612	-2.798301869053
C	-0.883782866473	0.632833744622	-2.115670820584
C	-0.963340619292	0.426440905470	-0.755592141733
H	-1.909156952391	0.573853873698	-0.241383581768
H	-1.767798919143	0.939828839939	-2.669292948433
H	0.393059965939	0.614946532258	-3.871716135365
H	2.414942971864	-0.068036177418	-2.619239006245
H	3.516299815772	-0.660557999733	-0.503788597075
H	3.402470449107	-1.008147917695	1.947919746975
H	1.253217548336	-0.710419395573	3.152125148302
C	-1.130517724650	-0.002515617657	2.181111549870
C	-1.296812674545	1.138461887577	2.940498137912
C	-2.473661412545	1.357838835418	3.688553774749
C	-3.499597183751	0.435436672268	3.687578612203
C	-3.361260368328	-0.774425105243	2.936952973875
C	-2.164436414938	-0.993993477567	2.177141072551
C	-2.031991200923	-2.210589643576	1.454862396903
C	-3.026719301664	-3.162959880147	1.471410508046
C	-4.206029602536	-2.945075759581	2.218858119973
C	-4.365740325275	-1.779883220028	2.935451958492
H	-5.269233867972	-1.629181830482	3.520082601774
H	-4.985697536428	-3.702375898943	2.233463785482
H	-2.903218856937	-4.087487870712	0.913130424632

H	-1.122972529522	-2.386505205223	0.886595881754
C	-4.726555709608	0.712084629784	4.491077037572
C	-4.710522345579	0.559985444670	5.841813893368
C	-5.882265348936	0.854348895849	6.686547047746
C	-7.080809593133	1.319601281864	5.999003499701
C	-7.117057387287	1.485205672347	4.630710467370
C	-5.934078550422	1.191602627312	3.845676705406
C	-6.004919339077	1.399578818328	2.460486345794
C	-7.175451557435	1.860097664822	1.874073351604
C	-8.336153322616	2.139255136632	2.632829890263
C	-8.308405034370	1.956970439400	3.991154045514
H	-9.180224508309	2.167125555878	4.604363436791
H	-9.233288078518	2.497023231642	2.138099632783
H	-7.200065250670	2.011705419926	0.798242754661
H	-5.136141620894	1.201452709792	1.841762838733
H	-7.961356960641	1.542625215947	6.597305564676
H	-5.623530465461	1.586127097330	7.476473381927
H	-6.150165290849	-0.027209305243	7.301022692328
H	-3.812244145099	0.206333899164	6.340176737744
H	-2.574810626365	2.274919924768	4.263765280996
H	-0.512849360081	1.891436397168	2.954797375195

25-Sigma-4.xyz

51

-1155.27653235

C	0.173636879844	0.123664814701	-0.194279180399
C	-0.117205290142	-0.209669868466	1.169755448269
C	0.906648356680	-0.623702606520	2.005389803723
C	2.241589256741	-0.699085636849	1.551742388878
C	2.549251689060	-0.365533326016	0.252924500120
C	1.531967669273	0.045553153763	-0.647485512074
C	1.833416594821	0.374140687170	-1.996579169955
C	0.841163099932	0.752420792262	-2.872393635150
C	-0.502566738944	0.809201828258	-2.433767141245
C	-0.827616677580	0.501582900672	-1.130412746437
H	-1.865792710354	0.541459444205	-0.813783786450
H	-1.285621976738	1.093906569505	-3.132139707575
H	1.085017639443	1.000574992707	-3.902273309454
H	2.868273710237	0.316064335584	-2.327379717545
H	3.576134237068	-0.416060228021	-0.102554485163
H	3.022070363535	-1.014732681855	2.239067333519
H	0.682265439753	-0.872693246132	3.039788402856
C	-1.487362045221	-0.061114004319	1.728832706363
C	-2.051199877841	1.203277489620	1.808095751646
C	-3.299833567947	1.409011355956	2.406787287214
C	-4.012713476864	0.352801166235	2.975318351836
C	-3.497296141238	-0.985960670101	2.853998861496
C	-2.226091028162	-1.187868756085	2.221263893303
C	-1.751670650417	-2.515149105348	2.052233735676
C	-2.486951632901	-3.597531126944	2.484498667002
C	-3.744574241868	-3.398425447330	3.088382929613
C	-4.242382863214	-2.122504702079	3.255254998215
H	-5.235212797517	-1.995004626951	3.675899184580

H	-4.335702123013	-4.253050518652	3.406280023961
H	-2.107378581695	-4.605326688327	2.339701272831
H	-0.800569329570	-2.672470861748	1.553690956996
C	-5.176700477546	0.683264858130	3.788237088259
C	-5.235825361566	0.179141217664	5.121839084136
C	-6.159291759633	0.643664571205	6.006273545204
C	-7.177056383755	1.639194958488	5.639769068494
C	-7.195090325390	2.049855287312	4.206378705194
C	-6.205273884452	1.564237624517	3.304283031867
C	-6.296792588928	1.909747930618	1.930805499483
C	-7.295025723621	2.752837171217	1.482639777738
C	-8.235641420913	3.264229828721	2.389649541437
C	-8.190144763864	2.905786581447	3.733026076830
H	-8.940415706417	3.285783773939	4.421736661338
H	-9.020968354015	3.927481769546	2.037473004247
H	-7.360785698363	3.005306226435	0.428606157624
H	-5.595121513291	1.482512194457	1.222832374277
H	-8.170133660279	1.265570057660	5.940545128486
H	-7.040578210286	2.520921573996	6.291346581190
H	-6.130950282603	0.310938423511	7.040743489812
H	-4.465057212339	-0.508206222693	5.453970950737
H	-3.668401132757	2.424874287233	2.512230224911
H	-1.497073185765	2.061790047397	1.439532609827

25-Sigma-5’.xyz

51

-1155.27183090

C	-0.078087214749	-0.157064693458	0.085945032714
C	-0.059393088946	-0.065348277826	1.573836674399
C	1.198522101734	-0.059368535138	2.254540340587
C	2.378014245688	-0.028772368029	1.483669851232
C	2.399260750381	-0.081864686350	0.077299530124
C	1.216230052911	-0.153639954698	-0.603928204875
C	-1.248674435755	0.040040699584	2.304291501076
C	-1.160574828383	0.163531070251	3.696282609421
C	0.061927959067	0.141230985588	4.375574327261
C	1.258796411867	-0.000319676323	3.686637373999
C	-2.582505054235	0.061091668932	1.635779127371
C	-3.181077481945	-1.145027410865	1.148192244985
C	-4.452677054233	-1.067122165022	0.491993773790
C	-5.092597648950	0.193049920109	0.355900122651
C	-4.506294991473	1.333929384021	0.852210113011
C	-3.247947718066	1.264674880545	1.493223222046
C	-2.573260567431	-2.421340605693	1.297555784784
C	-3.180221203878	-3.557880425320	0.808610037938
C	-4.428746861155	-3.477172900690	0.148409332746
C	-5.050357358692	-2.257782711029	-0.001381928078
C	2.537094051246	-0.159881232942	4.419778451635
C	3.022950308866	0.847091382205	5.318721862270
C	4.216967435201	0.579499280097	6.065639780335
C	4.892300666053	-0.657518125555	5.892247166225
C	4.419314925398	-1.597692934582	5.006772549993
C	3.242230247588	-1.345025300822	4.270080507194

C	4.711857952030	1.566048283019	6.959134778996
C	4.069688965240	2.775161373457	7.105092750567
C	2.906448237818	3.051284360142	6.350362909133
C	2.398139387412	2.113195244542	5.477522929020
H	1.512498396847	2.350328787953	4.895375930641
H	2.412096100471	4.013755826909	6.454775547718
H	4.459363778574	3.522430032377	7.791486864992
H	5.615865650863	1.350289092367	7.524453988953
H	5.793851156272	-0.848112755488	6.470141859261
H	4.938347858051	-2.543228931768	4.875760235366
H	2.860995816656	-2.113095093802	3.601427814629
H	1.204499874801	-0.207856008418	-1.689455074687
H	3.349689762913	-0.058281238227	-0.445056513646
H	3.327088980854	0.057044136114	2.005897891827
H	-6.014896032498	-2.188411809769	-0.499875041993
H	-4.897244706808	-4.380594950031	-0.233509790396
H	-2.698813471456	-4.524342080552	0.934921874624
H	-6.057513424529	0.240625676092	-0.144152207122
H	-5.001475149460	2.296019437014	0.750955074570
H	-2.790354902977	2.176243772674	1.870522734552
H	-2.079205724549	0.251785521238	4.270756505729
H	0.067603002443	0.193184042547	5.460259469272
H	-1.621768586065	-2.502692153243	1.817126084699
H	-0.620948112930	-1.066971890833	-0.234245622201
H	-0.710008458080	0.640874058694	-0.342562179341

25-Sigma-5.xyz

51

-1155.27187505

C	0.092400673381	-0.144358255259	0.103119899835
C	0.091253705660	0.413785686494	1.422822059412
C	1.252415291305	0.970591681276	1.926614834889
C	2.449788117522	0.983212952942	1.173883526072
C	2.479177317614	0.438912026774	-0.089010950516
C	1.308445558762	-0.131271642377	-0.655443811962
C	1.313243658774	-0.682365128963	-1.965325073736
C	0.169015210655	-1.217910858189	-2.512685266153
C	-1.035615481520	-1.220767000532	-1.769962779168
C	-1.072192193582	-0.697533514616	-0.495809797426
H	-2.004438393473	-0.701342319354	0.062446174579
H	-1.938414272552	-1.637370042190	-2.209913969413
H	0.186091752674	-1.634904134106	-3.516536564536
H	2.241922185506	-0.669854513118	-2.532209244751
H	3.397564940978	0.442313031618	-0.672342350861
H	3.346043383599	1.423386636099	1.603682436510
H	1.246549698611	1.397793769288	2.926743805082
C	-1.149572274815	0.433883552491	2.251246194185
C	-1.836053936918	1.620704937008	2.415767715688
C	-3.017964636403	1.682859936608	3.180932400858
C	-3.536820773480	0.555874418190	3.794829947386
C	-2.834122914190	-0.688998429595	3.685961807096
C	-1.631692220695	-0.746411756598	2.905175100804
C	-0.930846568698	-1.979412997764	2.823759895785

```
 C        -1.383239378783     -3.103652858770      3.478016521464
 C        -2.559237458589     -3.043615224200      4.257589406354
 C        -3.262594619488     -1.863652910298      4.361229108715
 H        -4.154209686371     -1.825981671305      4.979994502992
 H        -2.905523751500     -3.928291614840      4.785723329399
 H        -0.829080415690     -4.035824931215      3.403834413098
 H        -0.015691606392     -2.024382301376      2.240349351634
 C        -4.838922923318      0.659211234251      4.498041017704
 C        -5.944656026540     -0.040172711942      4.033005031306
 C        -7.205441881727      0.131541951473      4.621017915853
 C        -7.395564608036      1.005344300400      5.688784679097
 C        -6.313780165005      1.712202068017      6.201480880682
 C        -5.016945861491      1.527811243333      5.626865104280
 C        -3.919505037115      2.164359123209      6.238364370991
 C        -4.033394459478      3.038826558544      7.337047089499
 C        -5.267724706498      3.289056423231      7.869728400257
 C        -6.481816350652      2.650684837608      7.347430577234
 H        -7.197581130616      3.449935532493      7.080378499765
 H        -6.997731126371      2.149702800074      8.185128142178
 H        -5.379900037385      3.970587844968      8.708956102117
 H        -3.140973524579      3.498609901257      7.748004800045
 H        -2.926037887856      1.962613194017      5.846716712670
 H        -8.384346867936      1.133818910904      6.120235425685
 H        -8.054514629849     -0.416331306341      4.221562476570
 H        -5.839339871722     -0.702724160452      3.178974623167
 H        -3.551200539625      2.628018997341      3.249112271471
 H        -1.474753344646      2.522278344830      1.927848459419
```

25-Sigma-6'.xyz

```
51
-1155.26622120
 C        -0.080474999683     -0.042352729995     -0.000772545809
 C        -0.051629069111     -0.071222757094      1.418447178842
 C         1.208875625480      0.009584058389      2.095994647758
 C         2.385398015122      0.155444264544      1.313762330283
 C         2.323165131442      0.184313772483     -0.063229541564
 C         1.081465326450      0.076777863045     -0.729804630958
 C         1.215967663521     -0.013418018708      3.531699206206
 C         0.014227229324     -0.080373657131      4.224775111131
 C        -1.220260647326     -0.160492058975      3.547606151935
 C        -1.251362615279     -0.162755074344      2.172452788409
 C         2.474627646461     -0.033684538229      4.305541670976
 C         3.403404840199     -1.042959670133      4.132930773211
 C         4.535827229338     -1.126604237130      4.959126857699
 C         4.802672078145     -0.220058343816      5.987925237885
 C         3.899364406591      0.839335581346      6.196074197081
 C         2.744134620547      0.960716262192      5.322205248106
 C         4.096686269567      1.804132622598      7.247060241084
 C         3.284143828400      2.875952883023      7.387617893675
 C         2.170845773498      3.114221511776      6.449282940704
 C         1.958854743461      2.090012393025      5.427885474161
 C         6.045164231166     -0.383122196235      6.797609052060
 C         6.136616033186     -1.398673017661      7.802039790545
```

C	7.372784710322	-1.556138449545	8.510512954888
C	8.465655378543	-0.701774192430	8.207905666101
C	8.346982281795	0.274335940524	7.246004607845
C	7.132505702873	0.430902423397	6.539788647979
C	7.475858440578	-2.565139132709	9.505081882868
C	6.407139416515	-3.382383483175	9.798079296496
C	5.182665940941	-3.220431651974	9.108225546366
C	5.050178155793	-2.252488585575	8.136337404003
H	4.100924648400	-2.136624954965	7.620483390531
H	4.339639790226	-3.863168280252	9.349061979173
H	6.499516666130	-4.150786395876	10.561402722078
H	8.418880794494	-2.679978559110	10.035334337804
H	9.399343091130	-0.831392994374	8.750879246362
H	9.185404309832	0.926863502034	7.017268466840
H	7.056975489761	1.197731624237	5.772319755620
H	1.152284180647	2.253409989629	4.718645118910
H	2.276364179446	4.095505651745	5.948367608642
H	1.217203944386	3.256543575955	6.993523370633
H	3.439998143456	3.593416940848	8.187765863690
H	4.929893727719	1.653360587547	7.925684757736
H	-1.042871122438	-0.108748466550	-0.503427352394
H	1.046457964959	0.099348421660	-1.815852637466
H	3.236880153901	0.298048058616	-0.640868349658
H	-2.198788771824	-0.230937165429	1.642678711646
H	-2.140077155185	-0.231395360590	4.121342942378
H	0.024614827286	-0.118828284679	5.311166050265
H	3.239617879206	-1.809983816646	3.382312707553
H	5.230552946842	-1.947914573614	4.800785893147
H	3.348203678855	0.258747127207	1.805198458257

25-Sigma-6.xyz

51

-1155.26509631

C	0.099479074028	-0.180633576440	0.072333897531
C	0.064957447918	0.479781749965	1.343390224807
C	1.206168870566	1.100411883400	1.817364068840
C	2.414844873482	1.080694768082	1.082959140835
C	2.476227819888	0.438027625176	-0.131584153903
C	1.327117681904	-0.201560826405	-0.667313822299
C	1.364592639470	-0.856534184149	-1.927936022586
C	0.240960635140	-1.460144861347	-2.446336157369
C	-0.975226791734	-1.430067785884	-1.723218510254
C	-1.043199611613	-0.806527755492	-0.496392118800
H	-1.983420196131	-0.786586577180	0.048007356778
H	-1.861649750127	-1.901434999755	-2.140652319296
H	0.283259223555	-1.956714801783	-3.412542277652
H	2.301753717774	-0.869279770324	-2.480761841906
H	3.403857874208	0.415127670425	-0.699643633004
H	3.294537240919	1.573847350092	1.488851007918
H	1.175513244601	1.604769534057	2.780445824012
C	-1.187691459923	0.532507724541	2.153147569892
C	-1.911266712611	1.707247523979	2.211502950497
C	-3.100428716777	1.800250218972	2.965465272335

C	-3.579313087360	0.718717610420	3.677006630056
C	-2.848539293944	-0.510840630088	3.669570241983
C	-1.642169374304	-0.603336183956	2.898292302935
C	-0.918125112995	-1.825940909414	2.913928123990
C	-1.356309763647	-2.906952336263	3.646096047560
C	-2.543511485414	-2.814734070769	4.406546886925
C	-3.268157588973	-1.643549018422	4.419307560287
H	-4.172661814936	-1.579145281578	5.017683471587
H	-2.881943061875	-3.668141163769	4.988590251579
H	-0.785082987591	-3.831806920182	3.644441861974
H	0.000697768264	-1.898632819379	2.339072355942
C	-4.871167319340	0.841366443022	4.413254128124
C	-6.025607391088	0.283447804578	3.864774646368
C	-7.299544742922	0.395533062786	4.466067367943
C	-7.430345563537	1.081260220445	5.644857566199
C	-6.277025157199	1.664394608465	6.256927446030
C	-4.971206918618	1.538784678513	5.638355015463
C	-3.824357945814	2.114173115231	6.286372058855
C	-3.940148567103	2.788076874131	7.455619955026
C	-5.247969161805	2.962008272490	8.114895589561
C	-6.394618925896	2.354190790118	7.446441544902
H	-7.370923548827	2.457165845068	7.915009118574
H	-5.468450271983	4.034587345714	8.281283000192
H	-5.215272823020	2.593318509938	9.158534506236
H	-3.067453326865	3.217162206924	7.938884961335
H	-2.854379595340	1.996161451129	5.814649995047
H	-8.395911367635	1.193595085612	6.129533127841
H	-8.160874422543	-0.056130683671	3.984486039207
H	-5.940318287113	-0.255327872439	2.924156382293
H	-3.652915573367	2.736839735621	2.969971718274
H	-1.569898822965	2.575076242036	1.652785719239

25-Sigma-7.xyz

51

-1155.26713458

C	0.086273556445	-0.152831274995	0.080438017509
C	0.094100855592	0.395237095676	1.404319354115
C	1.256383548750	0.954411520441	1.902861921905
C	2.447445885322	0.977728913370	1.140588956371
C	2.468767021282	0.442862852820	-0.126460428274
C	1.295993593709	-0.128199572013	-0.687746915921
C	1.292549216583	-0.668554429010	-2.002083063829
C	0.146169033762	-1.204354535872	-2.544568603695
C	-1.052384816640	-1.218346743654	-1.792226469276
C	-1.080947970587	-0.705730934729	-0.513608319681
H	-2.008953795558	-0.717892995463	0.051552031945
H	-1.956992860966	-1.634955359304	-2.228402998290
H	0.156743854667	-1.612975184827	-3.551930477625
H	2.216521111993	-0.647266960885	-2.576311071824
H	3.382326314409	0.454665082295	-0.717178755901
H	3.345170860989	1.418739941869	1.566371275669
H	1.256783125794	1.374305338035	2.906073231713
C	-1.139229529393	0.403767134640	2.243429966486

C	-1.824229766649	1.588838821805	2.428424370239
C	-2.999482528332	1.640644343101	3.202078762811
C	-3.517590095006	0.503306613122	3.801994750636
C	-2.812142971565	-0.740150984390	3.676421781416
C	-1.614489732765	-0.784836874251	2.887851094329
C	-0.907348901629	-2.012753237575	2.791008301305
C	-1.348182357899	-3.144789219538	3.439764710478
C	-2.516608287428	-3.096259646176	4.230512443926
C	-3.225866834593	-1.920925434763	4.349005491112
H	-4.108313878931	-1.893469202107	4.980966338306
H	-2.852332295225	-3.985663854998	4.757384134664
H	-0.789344792435	-4.073106363834	3.354292286383
H	0.004337386687	-2.047053574200	2.201655352848
C	-4.819649620426	0.600379206435	4.494437801789
C	-5.903359235922	-0.150749478494	4.075551759081
C	-7.177572929285	0.038464965122	4.643167738487
C	-7.405184779639	0.973919662210	5.644178406736
C	-6.345351800478	1.739267198603	6.138198289162
C	-5.019758182321	1.536882661587	5.582066912058
C	-3.959963301006	2.193063560166	6.172025412820
C	-4.130614311045	3.148616375288	7.267015542059
C	-5.513377103429	3.381071495509	7.729078493454
C	-6.549098706482	2.698660628650	7.190477453289
H	-7.563256549733	2.851313506669	7.549022125594
H	-5.670744560283	4.105525087559	8.522543000100
H	-3.482192048815	2.837406544041	8.108122844639
H	-3.648523467067	4.099731396953	6.968986341862
H	-2.945076570925	1.993919978714	5.839052635591
H	-8.403664668686	1.111305847591	6.048432701284
H	-8.009697250708	-0.550302940510	4.266558629601
H	-5.785561244882	-0.860323838884	3.262363551546
H	-3.535606482133	2.583056291856	3.279902655618
H	-1.468108594319	2.496160344370	1.947747462576

25-Sigma-8.xyz

51

-1155.27140202

C	0.095775968782	-0.220420530149	0.046306922206
C	-0.004707993128	0.573717631675	1.234726784337
C	1.082834953910	1.322512434801	1.645156924438
C	2.300930595049	1.308145110595	0.926269020826
C	2.425564423193	0.541458983922	-0.208923953235
C	1.332880598495	-0.235101228754	-0.677543134133
C	1.435678618191	-1.022665290934	-1.855983391473
C	0.366028282918	-1.761003565885	-2.310257806880
C	-0.859832923028	-1.738778645779	-1.603357610308
C	-0.990528791517	-0.987903476621	-0.455516074483
H	-1.937705740866	-0.975441678695	0.076873222753
H	-1.703738278026	-2.317709529182	-1.970575010148
H	0.458109451125	-2.358522752469	-3.213821626481
H	2.379470478666	-1.029421469419	-2.397495833023
H	3.360778573553	0.522843377384	-0.764592223800
H	3.137426898918	1.905040398041	1.280911972028

```
 H        1.002100382659      1.927213578220      2.545517381603
 C       -1.272242604035      0.628273639164      2.021350409038
 C       -2.084267933796      1.740970885072      1.927403374856
 C       -3.286477232064      1.837101633033      2.660678038114
 C       -3.692355082386      0.820393747588      3.501151092659
 C       -2.883230737259     -0.352731769610      3.629559557556
 C       -1.657129745321     -0.443395730784      2.890354041878
 C       -0.851106935286     -1.602766563938      3.051013179866
 C       -1.236211591412     -2.631991258916      3.880929600424
 C       -2.455706913429     -2.552704254213      4.590259697274
 C       -3.257594378068     -1.439858166144      4.465695049542
 H       -4.197776629765     -1.392338096192      5.009299990294
 H       -2.761528339160     -3.372096537089      5.235762624028
 H       -0.603244562718     -3.509056558903      3.989205761349
 H        0.085460324938     -1.669278335155      2.504808398211
 C       -4.975240223872      0.963212014679      4.250924236992
 C       -6.192257977852      0.738655381588      3.597078950614
 C       -7.432203092681      0.884522854449      4.241859650969
 C       -7.471096006950      1.264731497011      5.565590413806
 C       -6.257040959652      1.509609154281      6.257095121341
 C       -5.001469266510      1.358356264037      5.596343585912
 C       -3.752604775353      1.641855143507      6.355055511549
 C       -3.898621807765      2.070209190051      7.750496867763
 C       -5.122164441270      2.197731698537      8.353136428366
 C       -6.279621384719      1.917663839853      7.606763931071
 H       -7.248417178119      2.023140973586      8.090680656392
 H       -5.209117948732      2.511006978894      9.387841373356
 H       -2.987592178361      2.282152405184      8.304207562831
 H       -3.139189768150      2.391786658077      5.823061063107
 H       -3.082151203327      0.762181041986      6.344596952277
 H       -8.415981526777      1.384943928900      6.088062217275
 H       -8.349645011656      0.696704261010      3.692797305548
 H       -6.171484403000      0.438291933840      2.552522451583
 H       -3.899086964702      2.729937964306      2.560474513078
 H       -1.798802811389      2.558472394588      1.270069915008
```

RING CLOSURE SCHOLL REACTION

1a.xyz

```
37
-826.097604158
 C       -0.003313603441      0.166367530275      0.019176788096
 C       -0.027563590522      0.081068363710      1.435933068303
 C        1.209851452436      0.060767947237      2.155704371927
 C        2.424818837463      0.120547984822      1.423812302532
 C        2.414612215952      0.201815184420      0.048525545732
 C        1.191741566854      0.226646614832     -0.661550665933
 C       -1.252567417388      0.010440313434      2.149394924755
 C       -1.264125103855     -0.080077263931      3.518877465975
 C       -0.034734098216     -0.096865651786      4.207405021664
 C        1.193973902196     -0.024238302337      3.586165602794
```

C	2.465185649616	-0.046740550041	4.370915162087
C	2.995616989576	-1.258767743480	4.778408728554
C	4.205350236295	-1.318961418759	5.508358665314
C	4.879822526361	-0.162315896159	5.821867552517
C	4.372909334018	1.103913003286	5.426886503909
C	3.144584476309	1.173748989691	4.692664442077
C	5.056640309342	2.307382504575	5.746102881863
C	4.552571905375	3.529534721809	5.361904588584
C	3.336886379205	3.599442029887	4.641332655028
C	2.649475652715	2.450772459023	4.315325057555
N	-0.083194485781	-0.184011245535	5.677799898152
H	-0.947682588417	0.181770061160	-0.519824628397
H	1.199158892936	0.291142577265	-1.746394418881
H	3.353987502077	0.246086690890	-0.496348864481
H	3.369318589173	0.098305830364	1.959385590517
H	-2.200995103764	-0.135438796425	4.066246495777
H	-2.189519391284	0.026449165725	1.599049020337
H	1.716159101839	2.518545159550	3.762777201212
H	2.942656330366	4.567755774238	4.343580176456
H	5.085022067260	4.443542949624	5.611965994390
H	5.988932599709	2.245440590894	6.303217412648
H	5.812335650758	-0.202648677478	6.380428697639
H	4.596791281647	-2.285502309941	5.813543084967
H	2.479448196419	-2.181461825592	4.522867038216
H	-0.586966457401	-1.017599369210	6.001469309617
H	0.868216783180	-0.236405243118	6.065611900601
H	-0.542168361009	0.630625348028	6.101116315928

1a-sigma-5’.xyz

38

-826.482838884

C	0.070192157358	0.286383891279	0.076908016632
C	0.004964907396	0.114738203553	1.483981633025
C	1.217964192132	0.103619708622	2.244422184282
C	2.452744337136	0.252389108208	1.559033188828
C	2.481447039976	0.414622595247	0.191239897898
C	1.282421114910	0.435626736275	-0.557553329498
C	-1.237116298655	-0.051126047693	2.148007804582
C	-1.291724202438	-0.226956566338	3.508012582586
C	-0.089964360572	-0.234316088182	4.242962748903
C	1.151651003219	-0.064475493420	3.666397404199
C	2.405143559976	-0.080998124193	4.477204847030
C	2.981147728959	-1.285146125524	4.842952434031
C	4.180548924833	-1.312907426807	5.581116983151
C	4.822919447349	-0.141254462200	5.953186833276
C	4.272600358898	1.092881987822	5.602761817400
C	3.053276828181	1.136608677068	4.866658973573
C	4.940610225722	2.365352683609	5.985472446991
C	4.278974527007	3.612724377817	5.596598678909
C	3.098366069712	3.619947135697	4.899263549471
C	2.505451524419	2.398360295668	4.543346348183
N	-0.201260755918	-0.414731048088	5.701682572702
H	-0.856780954132	0.295490610671	-0.491086949171

H	1.322127606930	0.565843860705	-1.635543074084
H	3.435431351316	0.525250117650	-0.317139371344
H	3.385145736299	0.229523143923	2.115217869647
H	-2.244048407548	-0.355127458173	4.014534327383
H	-2.154838493136	-0.040919655754	1.566439067062
H	1.569404932703	2.427819978489	3.990722957204
H	2.617832403193	4.550891587054	4.618700559473
H	4.759408178280	4.545856953803	5.878861342860
H	5.972220656656	2.387924836754	5.586816230465
H	5.751208823514	-0.181034544472	6.515862886472
H	4.610577821682	-2.272279863574	5.853839296626
H	2.512512969890	-2.219727459533	4.547867262542
H	-0.740116109115	-1.255287066512	5.941764789783
H	0.718633957330	-0.515394772597	6.146610315589
H	5.121810480665	2.393742027581	7.075392432907
H	-0.668102645541	0.380839624235	6.153757712428

1a-TS-ring_closure.xyz

38

SCF=-826.450460622 ; NI=1 ; Neg-freq =-377.9257

C	0.208436883177	1.005687924887	0.479651590738
C	0.090375313799	0.288139277077	1.701286368122
C	1.214193139747	0.209991701299	2.563977637315
C	2.466415122608	0.829981941203	2.155742482115
C	2.509350813552	1.530563735457	0.926043334170
C	1.380929298346	1.652940666025	0.117806841529
C	-1.141964715185	-0.283483680798	2.100560753954
C	-1.270570308602	-0.830190067383	3.355202586796
C	-0.175000251362	-0.799216221200	4.233126282236
C	1.077565717895	-0.317016821426	3.877818988564
C	2.238875810089	-0.206300788357	4.788284311367
C	2.645828849152	-1.241519254705	5.627303529692
C	3.781686843043	-1.093130750289	6.432654292790
C	4.542172363439	0.063673304434	6.358660437595
C	4.192847734566	1.095414196259	5.474322576273
C	3.020112911489	0.978266278038	4.716194428147
C	5.085671303723	2.293902572628	5.338321135412
C	4.708884965692	3.235541816969	4.256115386171
C	3.597402528436	3.102642899010	3.503366455468
C	2.659711600881	2.023967341460	3.737345676705
N	-0.431569784528	-1.240921408584	5.614063147817
H	-0.660778022631	1.063526612088	-0.170659315286
H	1.430860701958	2.208405249006	-0.813027662564
H	3.456460983767	1.952168544867	0.600811347431
H	3.395537665979	0.333596671904	2.426435309832
H	-2.221255434440	-1.240159778151	3.683101586053
H	-1.994230591540	-0.249303562741	1.429022447747
H	1.614190968239	2.324448719122	3.705374583816
H	3.335319564734	3.857365594848	2.767830817912
H	5.345119518682	4.106809890614	4.117848204412
H	6.125166631451	1.964293673908	5.187578613908
H	5.441101797322	0.163019985489	6.961964992035
H	4.082789193481	-1.904106232732	7.089156416391

H	2.129372038651	-2.197616091596	5.619874896122
H	-0.573526184354	-2.256022576369	5.684308868986
H	0.341745829991	-0.994344577440	6.245000132985
H	5.119411581492	2.850811727495	6.289077365628
H	-1.275853382739	-0.797074512315	5.994729151615

Intermediary_for_the_formation_of_4_Table_3.xyz

38

-826.459650103

C	0.174134293832	0.949039739027	0.478813499528
C	0.062359517552	0.275276333797	1.719879637233
C	1.141416700994	0.345003534733	2.632904187061
C	2.360320324201	1.129603821655	2.303636846003
C	2.329568537467	1.839175629946	1.021998334732
C	1.287607469065	1.723317700948	0.132629188957
C	-1.123378679479	-0.412636692962	2.069166243725
C	-1.245211147804	-0.942199444639	3.334488246632
C	-0.177594786049	-0.825116963908	4.232465466360
C	1.055417238915	-0.251321814950	3.906797922449
C	2.233683225997	-0.168278276180	4.792454822838
C	2.579564982326	-1.196132588453	5.678910868791
C	3.702054960802	-1.067735871726	6.495385336743
C	4.493634458074	0.069785890597	6.405471410289
C	4.203432882877	1.084504310184	5.481862890329
C	3.071419564719	0.965825035947	4.670550598246
C	5.136668624061	2.262678761974	5.370913381332
C	4.886457037426	3.122881216038	4.176248702594
C	3.833230624557	2.987257948021	3.365547245403
C	2.749077796065	1.979795075477	3.591124813890
N	-0.435087601006	-1.291614850208	5.603906740837
H	-0.652761795803	0.872052214742	-0.223534801799
H	1.318243173856	2.227257475482	-0.827202353344
H	3.201327957936	2.428323119381	0.751716346023
H	3.212450504943	0.426317115625	2.184638354171
H	-2.166572169147	-1.427318035026	3.642864418936
H	-1.941236937847	-0.484652168487	1.359632042647
H	1.826880397925	2.521977715545	3.851705511080
H	3.695719711571	3.689787538297	2.547125732186
H	5.601801628922	3.921690931539	3.989804913279
H	6.176256241587	1.906965778032	5.362105741433
H	5.375897841785	0.165375352793	7.034408878795
H	3.965895647148	-1.868233064896	7.180329607711
H	2.030575744617	-2.133636620010	5.694178590227
H	-0.448012178260	-2.316722016369	5.677349749870
H	0.267290303276	-0.942039315328	6.269045592541
H	5.059467580715	2.880504753435	6.279641027534
H	-1.349214677810	-0.962005270076	5.937311264739

2a.xyz

43

-904.677226289

C	-0.113376852975	0.077869989685	-0.054399245478

C	-0.089182132566	-0.032134209796	1.323820749165
C	1.169485149933	-0.088068651050	2.007842004707
C	2.376036968084	-0.071716364807	1.235140281512
C	2.299467690765	0.015682180002	-0.179926359723
C	1.080668196975	0.095022389777	-0.811477280112
C	3.627437911816	-0.136555503752	1.904787501894
C	3.692005761231	-0.207419372423	3.278092330459
C	2.501445785761	-0.215632422731	4.042808254495
C	1.272095727203	-0.158482055617	3.423393512048
C	-1.371728837128	0.011065976490	2.094297605673
C	-1.815948380708	1.286781185764	2.590581479448
C	-3.038726178166	1.390864416945	3.327121919339
C	-3.805600771284	0.223976872761	3.559302460633
C	-3.383166878645	-0.988706796083	3.079986295113
C	-2.174593960589	-1.087172692333	2.353464308224
C	-1.068079098190	2.474735062634	2.363350484774
C	-1.505158357579	3.690264076263	2.841796795775
C	-2.712535812128	3.786490307425	3.572213413537
C	-3.462742622227	2.657802556081	3.808288623778
N	-1.868828343932	-2.469102398388	1.886525169153
C	-2.098788836147	-2.675326133964	0.414398296456
C	-0.549706987700	-3.034442466510	2.328051578894
H	-4.393970150286	2.718588228032	4.366529146330
H	-3.044859716894	4.752442079208	3.943218665556
H	-0.917126685855	4.584975627397	2.654401605886
H	-0.142155019262	2.422354684152	1.799813040550
H	-3.982539102878	-1.877973551351	3.261201067092
H	-4.733347683873	0.293829798159	4.120663202710
H	0.366014200837	-0.164845210394	4.023477783980
H	2.558126996977	-0.267897546605	5.127156645272
H	4.655677234638	-0.255963644562	3.778635729106
H	4.537995890707	-0.127544596549	1.309496042596
H	3.221932980847	0.027206888021	-0.756247575413
H	1.024336370389	0.174276303935	-1.893750690109
H	-1.067585302158	0.169151363107	-0.567869057441
H	0.251636008254	-2.550743334947	1.775738487118
H	-0.442382653809	-2.864005833359	3.398696724014
H	-0.563346867278	-4.104026623131	2.114028141969
H	-2.075710029094	-3.748219427871	0.219621521310
H	-1.307423628895	-2.172579427650	-0.136190506380
H	-3.073643640313	-2.260808072472	0.158225794734
H	-2.571322000798	-3.051588761351	2.351503544890

2a-sigma-5’.xyz

44

-905.070772200

C	0.033871746988	-0.099985727107	0.057932849670
C	0.028939350163	-0.037974777932	1.469965307746
C	1.279103890367	-0.049213793198	2.153308496409
C	2.540662312686	-0.118555431837	1.369266315899
C	2.421496492954	-0.183099443659	-0.088456047140
C	1.202451592670	-0.174535042340	-0.716106057763
C	-1.196639744222	0.025859699121	2.213807204878

C	-1.127542432755	0.065468821169	3.596282354396
C	0.116028603116	0.059450134176	4.257501694558
C	1.305817001503	0.004598716004	3.548052485641
C	-2.524334844015	0.021716675923	1.526692704814
C	-3.077175951497	-1.214736564674	1.055931734967
C	-4.345195298614	-1.208194949647	0.391891784155
C	-5.033960104774	0.018751451277	0.222013870616
C	-4.508119277333	1.195236229558	0.693506757229
C	-3.258023695640	1.180760725647	1.345136900548
C	-2.411884207035	-2.456797491795	1.236290766132
C	-2.970111513107	-3.627376063970	0.771483911717
C	-4.216993348957	-3.617179340551	0.105062001148
C	-4.890408525154	-2.430927749992	-0.078316629750
N	-2.726758816581	2.470393527001	1.842729951789
C	-3.602539304209	3.088613057190	2.897415952973
C	-2.433612853223	3.450157741561	0.740135225234
H	-5.852778528229	-2.413016054861	-0.583889620178
H	-4.642984367791	-4.548859893220	-0.257069164349
H	-2.448009740300	-4.568441535215	0.921724312674
H	-1.458399844788	-2.489006864253	1.754832656932
H	-5.054415607602	2.123090442480	0.556938910251
H	-5.994891994377	0.022918244206	-0.285203894826
H	-0.921681006439	-0.089083078401	-0.461111835244
H	1.127238279809	-0.221783345253	-1.796969531952
H	3.340043927250	-0.237298951775	-0.666760729225
H	3.195572233790	0.735557000310	1.622557387967
H	2.256819421963	-0.001796369230	4.072833145412
H	0.136614288098	0.092319009866	5.342889061182
H	-2.040482648304	0.083909749691	4.184211641779
H	-1.949221581691	4.319846615868	1.185046324718
H	-1.775138216633	2.975944034458	0.014007175370
H	-3.372389124474	3.741581990155	0.271128097099
H	-4.545690410279	3.385506389211	2.441451163888
H	-3.084845090581	3.962614563560	3.293193061736
H	-3.775342857688	2.355579711159	3.684363344266
H	-1.829559783633	2.263035965055	2.297329060959
H	3.153216585586	-0.975981116796	1.704301194827

2a-TS-ring_closure.xyz

44

SCF=-905.038087026 ; NI=1 ; Neg-freq =-377.3796

C	0.012606965250	0.019603188703	-0.012494393121
C	0.006709848165	0.004213935136	1.408726751327
C	1.243770203881	0.003955660305	2.103555826005
C	2.483311888960	-0.020828946299	1.340241983097
C	2.413010697595	-0.001417249401	-0.073731268931
C	1.192119921709	0.061355404853	-0.741794367162
C	-1.204018143958	0.064750267678	2.139510714297
C	-1.177002522232	0.226443008709	3.504052721878
C	0.058092670374	0.342442788479	4.165625857671
C	1.278203153482	0.204431569345	3.511284910783
C	2.608466451865	0.373579983295	4.142451427863
C	3.583163683232	1.120796945990	3.428355964974

C	4.890620588335	1.227777247455	3.921982541252
C	5.202825886119	0.654123911773	5.163903344267
C	4.255718442308	-0.077283690350	5.863361223646
C	2.967357079793	-0.243326190205	5.339541975297
C	5.960320260050	1.924108714690	3.132708110985
C	5.562553836943	2.332057970876	1.763155577456
C	4.311906256288	2.194751075012	1.276385440229
C	3.245168032714	1.658654022193	2.095567603035
N	0.033132661937	0.713347510591	5.596146184338
C	-0.574234535885	2.073354400623	5.817863206784
C	-0.611788591546	-0.328250235864	6.468054417470
H	-0.941998128180	0.025880051583	-0.532771037097
H	1.161094440174	0.093120011740	-1.826011901394
H	3.337677843921	-0.066802050593	-0.640433250752
H	3.324602863993	-0.579417399366	1.743906696958
H	-2.110663211164	0.307967845375	4.051319578609
H	-2.153120260904	0.028626106859	1.613680314261
H	2.300499912089	2.191385965804	2.007418635266
H	4.059323525323	2.569938582504	0.289106091039
H	6.320351164695	2.821539035922	1.155569769543
H	6.309428523622	2.818221062795	3.674875981222
H	6.211451333215	0.754345704139	5.556900973002
H	4.518158954556	-0.556238450765	6.801961672373
H	2.281212199354	-0.917166236816	5.846898868827
H	-0.039651880796	2.794643643396	5.200367335738
H	-1.627895446115	2.040461030118	5.545490661941
H	-0.465225048998	2.316951621399	6.874712228505
H	-1.677361781379	-0.362387465152	6.246337828083
H	-0.452848517946	-0.039256573210	7.507527483512
H	-0.153664304579	-1.295155647403	6.261724650662
H	1.012920717389	0.790700832520	5.896973532371
H	6.853392608932	1.283126217752	3.076244446511

Intermediary_for_the_formation_of_5_Table_3.xyz

44

-905.047366496

C	-0.370606952191	-1.042379931674	0.314102198261
C	-0.069442892918	-0.294557407790	1.596636284474
C	1.209856837278	-0.279563098231	2.160144615310
C	2.380648640778	-0.943627485008	1.482210958811
C	2.000083937569	-1.813528140232	0.329890217256
C	0.767521067642	-1.878433945962	-0.181365283324
C	-1.147436661621	0.320806059541	2.275799588546
C	-0.938887712769	0.876943596170	3.544731567012
C	0.341126189478	0.903621534289	4.097285546644
C	1.404641130286	0.345737102551	3.400321495183
C	-2.480014318227	0.273202657375	1.634978893344
C	-2.785470303239	-0.856792268816	0.850703486039
C	-4.050808426605	-0.997840217265	0.233640630099
C	-5.036934900807	0.000760681013	0.405513490277
C	-4.723688642895	1.144821125948	1.104526828858
C	-3.452330118810	1.282748982409	1.678852764032
C	-1.710782533638	-1.853252250434	0.602434007133

C	-2.049585297070	-2.932510362751	-0.329617974859
C	-3.302152486643	-3.075761573828	-0.877523192458
C	-4.286660515549	-2.121108385824	-0.595734305652
N	-3.121274866958	2.591165621628	2.280595948070
C	-4.006332393460	2.957587177487	3.441722633867
C	-3.076500313113	3.687157028344	1.245950358238
H	-5.270921254461	-2.230528525862	-1.045505957072
H	-3.531210194077	-3.912898682869	-1.527891751749
H	-1.285650576981	-3.676470820842	-0.537412683052
H	-1.494096925088	-2.390151729525	1.550360504701
H	-5.459796197926	1.937229292753	1.186153325954
H	-6.016657759347	-0.117017910788	-0.046076033050
H	-0.644783358350	-0.331403055940	-0.480210524205
H	0.583797725601	-2.497702765086	-1.056107065770
H	2.796562168642	-2.399633345916	-0.124715873979
H	3.087653065799	-0.174432059724	1.132993620855
H	2.400911623870	0.355130872965	3.836668397262
H	0.494841490348	1.335651868423	5.081783729575
H	-1.772831790262	1.217265356278	4.153832033080
H	-2.723395701418	4.592295270962	1.740492034255
H	-2.387349568883	3.387751252227	0.457221862328
H	-4.076880265972	3.840927055432	0.844255241084
H	-5.005337015651	3.181251753932	3.070742120621
H	-3.579231397304	3.838425007190	3.921140952352
H	-4.043077540988	2.120600423135	4.138159691542
H	-2.163167521439	2.510342068737	2.646099658786
H	2.946683947362	-1.529674184421	2.219370661317

3.xyz

38

-884.802364960

C	3.038828331525	-0.920719157590	-3.364179597643
C	3.325735271077	-0.317356066356	-4.618108407119
C	3.023690151857	1.004867409550	-4.853316075540
C	2.417176706588	1.778555979472	-3.833694791503
C	2.128346998452	1.223425076125	-2.606568838671
C	2.429889013399	-0.140364119177	-2.327210318766
C	2.142177578743	-0.739497640478	-1.064865234116
C	2.452018298152	-2.081869679974	-0.860990492298
C	3.053719853550	-2.853330667815	-1.887296285217
C	3.337754741990	-2.280051670887	-3.105099231843
C	1.533206968179	0.058598035568	0.040290474240
C	0.113339786074	0.107150398458	0.217685124632
C	-0.785444385999	-0.583796659616	-0.639762145811
C	-2.147638346283	-0.518874876740	-0.443091581356
C	-2.682324196387	0.243073981399	0.623524351904
C	-1.839662262084	0.926433127303	1.471398041788
C	-0.430095040516	0.880886052560	1.295057513047
C	0.448671837854	1.584389876798	2.160474328276
C	1.809877451752	1.528001077536	1.967866752500
C	2.347914792968	0.764067511243	0.906017289432
O	2.149038414603	-2.596916412015	0.361676574500
C	2.439524837457	-3.963308935760	0.627289171187

H	3.790428410912	-0.923723613561	-5.393312742063
H	3.247274030274	1.456638867431	-5.816472918153
H	2.176806290200	2.822255618417	-4.022671752437
H	1.664900131415	1.829938033087	-1.833366245927
H	3.294886267121	-3.897311170879	-1.719516541290
H	3.800676109136	-2.878976875764	-3.886477593702
H	-0.381574392541	-1.170878455588	-1.460655489239
H	-2.818012222680	-1.055432272779	-1.110258670369
H	-3.758778243834	0.286866647861	0.769927929904
H	-2.243435448421	1.515207355852	2.292733665819
H	0.028243442927	2.169608682421	2.975835127282
H	2.480744912656	2.069383239725	2.630474843431
H	3.426305555213	0.728257099870	0.767984249333
H	2.110369095223	-4.139489820914	1.652420357273
H	1.889126066888	-4.628058641487	-0.048947164535
H	3.514604641191	-4.165334668754	0.552945945965

3-sigma-1.xyz

39

-885.207593173

C	0.842470066043	0.263356752613	1.993735160658
C	-0.129300717407	0.137373687447	1.019922306829
C	0.242801170522	0.027767246508	-0.356519128600
C	1.641748629967	0.065889040049	-0.684022960348
C	2.604802113009	0.195837175220	0.349132665936
C	2.214133001092	0.289831885495	1.664275822729
C	2.043256694681	-0.029781233191	-2.043533058788
C	1.115598691466	-0.160657983329	-3.050699071234
C	-0.261953760009	-0.202879387548	-2.734126266045
C	-0.684296081036	-0.113148719875	-1.426218746896
C	-1.592733501138	0.112738931703	1.470260855269
C	-2.002727893897	1.336209292350	2.257053548027
C	-2.748831148303	1.201428950598	3.451826077708
C	-3.060899112068	-0.102941628474	3.933717494120
C	-2.681425591439	-1.270828478877	3.304913003309
C	-1.945603429159	-1.195952275883	2.116702308562
C	-1.695334822100	2.613467480380	1.794637995866
C	-2.117726887213	3.737938363081	2.504340212787
C	-2.852440240694	3.609827905709	3.691070439521
C	-3.166764878362	2.347357583031	4.164085847599
O	-1.534617715134	-2.228757592426	1.443828350427
C	-1.828935692455	-3.575668190717	1.881595376779
H	-3.735865350441	2.223984244384	5.081711322039
H	-3.171859479922	4.494432994418	4.233650951606
H	-1.869190953095	4.726894786250	2.128888528039
H	-1.121121576286	2.736770984284	0.880608696978
H	-2.945598156927	-2.227072283955	3.739809421152
H	-3.630793031856	-0.180692665857	4.857247666568
H	-1.751585687877	-0.157623490326	-1.229007347722
H	-0.995371324339	-0.309429876519	-3.529231284473
H	1.435980774358	-0.233227124300	-4.086731897453
H	3.106145176475	0.001938273593	-2.273288626470
H	3.659009677797	0.220262748676	0.082538962614

H	2.952691342746	0.388532090488	2.455163418451
H	0.550521653612	0.351097829306	3.038199671416
H	-1.374761669487	-4.216591561900	1.129069494449
H	-1.374763146125	-3.752948337068	2.858480842337
H	-2.909905396913	-3.726764595568	1.911030502258
H	-2.234832208083	0.123506130231	0.574659523996

Intermediary_for_the_formation_of_6.xyz

39

-885.071545504

C	0.113213664789	-0.161118224137	-0.096240975592
C	0.097227553405	0.120786524714	1.281200603779
C	1.317269605085	0.217999425840	1.955586590970
C	2.538771484598	-0.028994345201	1.260979963968
C	2.511307475746	-0.396658283952	-0.099970932072
C	1.302223270899	-0.444842805818	-0.771847058025
C	3.782198800250	0.223308598991	1.913458509038
C	3.849230579225	0.848457946471	3.160564855230
C	2.686010939521	1.111244237026	3.852199328788
C	1.403319300389	0.500578993855	3.430504424660
C	-1.091455337667	0.780661412023	3.470177605032
C	-2.252835003096	0.942244256821	4.218935825660
C	-3.503112247489	0.575815768564	3.643660297110
C	-3.604347767702	-0.087124256853	2.435758495003
C	-2.441499254738	-0.318369806428	1.689073080730
C	0.219247692216	1.220458265347	4.050151981607
C	0.176220413886	0.931993058993	5.524778207769
C	-0.973179128575	1.142073051026	6.260763888875
C	-2.204233168413	1.269738862877	5.612178099755
O	-2.379146410240	-1.136777212582	0.681513705711
C	-3.536131884648	-1.894142940944	0.259350558671
H	-3.127657377925	1.415809481963	6.164086085353
H	-0.937870765724	1.097192446009	7.347392131176
H	1.087970094157	0.609452910641	6.021978520275
H	0.370713623766	2.322970849870	3.868240077448
H	-4.559609045195	-0.484634003757	2.114216813094
H	-4.409381302561	0.746569032735	4.220415266136
H	1.401920660421	-0.532005111452	3.891153401625
H	2.709380509151	1.689060914058	4.772324046700
H	4.809155375746	1.184085920662	3.543219701114
H	4.698175682401	0.028648151778	1.360757129679
H	3.445535381911	-0.600708587221	-0.616822530437
H	1.270824805629	-0.685961344069	-1.830585612593
H	-0.811924973282	-0.154218650273	-0.659392873784
H	-3.176653596200	-2.517447580469	-0.556822385610
H	-3.893973055635	-2.511763027523	1.085554792959
H	-4.314067478026	-1.214259763216	-0.094187848787
C	-1.201540326159	0.478591752546	1.996374341173
H	-1.493102789916	1.466156081086	1.561412887814

8a.xyz

34

-786.801054781

C	-0.006899007796	-0.070594885686	0.003021370364
C	0.011252341712	-0.013321925456	1.385690960735
C	1.252099811523	0.010422823457	2.100138256682
C	2.465951659201	0.005633651027	1.339833276173
C	2.405287918210	-0.032901587510	-0.077998104117
C	1.197137196503	-0.076348715881	-0.734005150201
C	3.710201119614	0.025078380905	2.024434662857
C	3.759216604772	0.037080220467	3.399818232432
C	2.560461571816	0.023291188997	4.150536016174
C	1.336730299171	0.008539393079	3.517854819661
C	-1.285712640222	0.006631808393	2.102676277497
C	-1.816851010683	1.126787019034	2.788637146148
C	-3.095963171699	1.001847128171	3.424708798793
C	-3.793590067057	-0.230154863516	3.329034449807
C	-3.245758127280	-1.268457443346	2.636339061423
N	-2.018694144085	-1.112519250110	2.055928922043
C	-3.631407580514	2.116060376460	4.112804764366
C	-2.933361109070	3.302891656716	4.155850629990
C	-1.679518225544	3.431389334482	3.510997494963
C	-1.127475070192	2.366960354873	2.837913422191
H	-4.598405922410	2.022613734184	4.598854928816
H	-3.350700671236	4.155546907329	4.684095392537
H	-1.153298064308	4.380672439782	3.546817489288
H	-0.169689028671	2.467750361529	2.337900721783
H	-4.764356117529	-0.350382355646	3.797696945750
H	-3.711839814906	-2.236847812561	2.509276989567
H	0.428435084718	-0.011947835670	4.113908780809
H	2.605172045130	0.020751382298	5.236479712390
H	4.717147016960	0.050313871486	3.912901220014
H	4.627035517008	0.024804858455	1.439280711026
H	3.335756075595	-0.033408986727	-0.641038621564
H	1.157931707718	-0.109049992897	-1.818951589083
H	-0.956631298506	-0.085605962646	-0.525976270916
H	-1.622398251195	-1.908910972915	1.562148045918

8a-sigma-5'.xyz

35

-787.182942255

C	1.427031868933	0.821924486475	-1.569788591191
C	1.334338891830	0.597775204391	-0.176086006293
C	0.121140365865	0.544156184320	0.523885191722
C	-1.055206362551	0.717201884776	-0.161379650996
C	-1.080459852427	0.964606913686	-1.603114828114
C	0.226646379588	1.026677812662	-2.307534031798
C	0.291859700131	1.291346681509	-3.677822653141
C	1.522418558198	1.356948588865	-4.312280000835
C	2.719789888990	1.153476105889	-3.600205999963
C	2.685981905426	0.879521562512	-2.246017461070
C	3.969255543824	0.667808816699	-1.524424971460
C	5.736390520021	-0.652713736957	-0.467992372792
C	6.243817288141	-1.910921095771	-0.068501981748
C	5.527395832390	-3.058349656902	-0.326762751735

C	4.281804879480	-3.002466897175	-0.998953447403
C	3.756186084189	-1.797794083309	-1.400186311526
C	4.465840783468	-0.595441125684	-1.135662305020
N	4.706535844926	1.756762276328	-1.276860770339
C	5.921021624096	1.742368792987	-0.655448268066
C	6.445800974168	0.552439875080	-0.241173716857
H	-1.662813441515	1.886129956101	-1.787215944949
H	-1.728117454325	0.198097242694	-2.069267805847
H	2.251294289584	0.463285030466	0.392566773981
H	0.126837875597	0.367705836407	1.593786972817
H	-2.008647671155	0.674201630088	0.358412410349
H	-0.622428696924	1.449791324423	-4.242514685657
H	1.570896496849	1.565905298170	-5.376665223261
H	3.669214335024	1.199523644956	-4.125178313766
H	7.202857502106	-1.956709865839	0.439005708119
H	5.922507116523	-4.021918457647	-0.018076172316
H	3.741541746228	-3.922100096187	-1.202065412727
H	2.808290098188	-1.762573047016	-1.927384744278
H	4.331997711339	2.659005448282	-1.562440860725
H	6.399588872928	2.703985279956	-0.523492342056
H	7.409000515398	0.537814220308	0.257393143038

8a-TS-ring_closure.xyz

35

SCF=-787.147084140 ; NI=1 ; Neg-freq =-360.0735

C	0.002169703580	0.010970534636	0.000966875298
C	-0.000310694379	0.006320245168	1.458630012230
C	1.214735971016	0.009263381641	2.179984421770
C	2.441873948923	-0.050880527479	1.451097735788
C	2.457058097562	-0.122348969552	0.070082721506
C	1.259448287375	-0.040068502685	-0.649119658443
C	1.177818485541	0.033780902498	3.594734151641
C	-0.033196835873	-0.009727222508	4.229206790065
N	-1.173744155408	-0.130568072150	3.498501783323
C	-1.217374086474	-0.146865036760	2.156202984939
C	-2.466583055158	-0.382053036833	1.439132666090
C	-2.383566488323	-1.024722393889	0.173379788675
C	-3.532963840626	-1.141551425554	-0.619284099732
C	-4.760159682744	-0.701364198279	-0.104084226785
C	-4.846430741114	-0.119000746554	1.155013983538
C	-3.697185782237	0.060069341309	1.925107653847
C	-3.468353188134	-1.722947139837	-2.001251235194
C	-2.106825442489	-2.084003712936	-2.464925852583
C	-1.003888662622	-1.986790754693	-1.696397587970
C	-1.080918736733	-1.531889028352	-0.318095414528
H	3.376229115243	-0.055367520309	2.005325740967
H	3.403055139944	-0.178903493417	-0.458166574771
H	1.288147117811	0.024375064071	-1.733079532975
H	-0.721692511084	0.659003990373	-0.493395509920
H	2.091187367872	0.065818944650	4.177337847205
H	-0.156772965100	0.004087099819	5.304208586947
H	-0.531319621121	-2.166067176263	0.377554154588
H	-0.044978872748	-2.340511655975	-2.063235741092

H	-2.024250197124	-2.511091457097	-3.461615075945
H	-3.923338170452	-1.020345525438	-2.716670163736
H	-5.655287739120	-0.805027900133	-0.712150098261
H	-5.803822236470	0.231075009796	1.528421891932
H	-3.769471346991	0.592196015198	2.869772601652
H	-4.106438113553	-2.618664837481	-2.064073779840
H	-2.042597070185	-0.266365784294	4.010773786745

Intermediary_for_ring_closure_from_8a_Table_3.xyz

35

-787.151580243

C	0.025399252257	0.024229325200	-0.015521698271
C	0.003668479846	-0.023091715112	1.585110114038
C	1.398596354371	-0.017663039815	2.075809558669
C	2.358889287718	-0.775057134747	1.376118799546
C	1.993751542200	-1.548797553524	0.201137310155
C	0.801926932920	-1.188582173744	-0.479572098480
C	1.788712682969	0.752107326656	3.183925539902
C	0.804423205755	1.581515658186	3.815385336379
C	-0.504236856643	1.656455791948	3.359270597742
C	-0.898547510549	0.888355834485	2.273366704645
N	3.621806697285	-0.743976646942	1.845237343664
C	4.027020600177	-0.054010357511	2.939238783295
C	3.128341669688	0.715302883330	3.632154933141
C	0.379158880955	-1.955918825187	-1.568594413244
C	1.164579855043	-3.040873189197	-1.982192299214
C	2.345042613269	-3.380693728453	-1.326848304304
C	2.759425409925	-2.641781819264	-0.226198997632
C	-1.323990570842	0.164003251630	-0.643154965636
C	-1.724836327749	-0.580415198960	-1.678110686200
C	-0.902649628965	-1.657360610966	-2.301320178815
H	1.105405421862	2.174038098129	4.675410571095
H	-1.218197566708	2.298435176789	3.863408519368
H	-1.937368419135	0.910765477085	1.956657880858
H	-0.441695918605	-1.020237885522	1.795458924252
H	3.452949633293	1.290244110298	4.491412323976
H	5.075451679789	-0.138543539690	3.194957343555
H	0.606495807284	0.932208366766	-0.236321207913
H	-1.958031726937	0.972564628170	-0.289033835043
H	-2.693307206031	-0.380300247780	-2.131755007630
H	-1.498004096618	-2.577746235979	-2.383662369310
H	0.832421036583	-3.635576698398	-2.830016727392
H	2.928415182647	-4.234533904448	-1.657290263898
H	3.648447835532	-2.955445321279	0.313405237393
H	-0.676433183879	-1.376561007319	-3.341481171167
H	4.336638581434	-1.255243779418	1.332213070535

9a.xyz

34

-786.799874048

C	-0.032222136795	-0.079722402438	0.056074592112
C	-0.006772786597	-0.024219666133	1.457126645818

C	1.273327413476	-0.002269993630	2.103514148265
C	2.441053871307	-0.060254366600	1.298323855277
C	2.340138084552	-0.124636759956	-0.061317286133
N	1.101080068245	-0.128364960097	-0.637157210717
C	-1.213071886822	0.029579925807	2.226769901404
C	-1.099232299649	0.125071973262	3.599042482122
C	0.163386264655	0.152842572034	4.238031523044
C	1.331776884722	0.084751376473	3.512885028655
C	-2.552191260395	0.011972842257	1.572017597520
C	-3.106323237368	-1.205488362668	1.059395276567
C	-4.394406832421	-1.164337530296	0.432109636420
C	-5.090749666333	0.069936647918	0.345991440836
C	-4.543886821440	1.221308715822	0.862843467127
C	-3.271221848008	1.189255155815	1.477373478666
C	-4.951560465349	-2.365532945215	-0.082651992597
C	-4.276656670553	-3.560903103915	0.022109943240
C	-3.011821955309	-3.605860378486	0.654405397045
C	-2.441648269114	-2.458249554736	1.160720176090
H	2.298703569937	0.100648544969	4.007295651076
H	0.203127983607	0.224339402952	5.321362556910
H	-2.000474462966	0.169824664432	4.203907374950
H	3.422733885714	-0.051115847311	1.759672342664
H	-0.952922364478	-0.080696034104	-0.514994774497
H	3.183926454009	-0.170417944312	-0.737082575548
H	-2.845079248503	2.108211180895	1.872540578258
H	-5.081920190664	2.163334125408	0.798766220534
H	-6.067501485592	0.089273396214	-0.132466299276
H	-5.928181247448	-2.323766226228	-0.560239530513
H	-4.715028640760	-4.473076342710	-0.374505778966
H	-2.487915820866	-4.554130065033	0.744148573191
H	-1.474459619477	-2.513255924133	1.653113052553
H	1.043616105253	-0.166793999467	-1.651445208764

9a-sigma-5’.xyz

35

-787.187535868

C	0.186155235160	0.272106729131	-0.016387381502
C	0.125278563714	0.053694839376	1.376844858228
C	1.344222162095	0.070981611747	2.119384840176
C	2.623981611775	0.366286123719	1.419306498438
C	2.557194039072	0.609212403862	-0.024110138547
C	1.374234152868	0.552355665846	-0.712656149301
C	1.322180629178	-0.197958787623	3.486645486208
C	0.116064492796	-0.482052348137	4.113943715129
C	-1.093602836925	-0.493260637899	3.398157094469
C	-1.115152292863	-0.218421752819	2.040712557044
C	-2.414706654631	-0.166362850087	1.316621406048
C	-3.186174822250	-1.349856303164	1.083065232783
C	-4.465149205063	-1.258781774691	0.440869825193
C	-4.947895137673	0.006874540548	0.034959669471
C	-4.184936204989	1.130227040152	0.253663403140
C	-2.924265369700	1.044015185157	0.890377132780
C	-5.204839892140	-2.450301335031	0.226638703874

C	-4.697176540749	-3.656664468070	0.614365963035
N	-3.466926700116	-3.700711120642	1.204744977563
C	-2.722586509859	-2.625168946099	1.439957899062
H	-5.917812523224	0.076917096407	-0.448263277715
H	-4.550902727053	2.104372476846	-0.057080474831
H	-2.359219956525	1.955006784807	1.066521061682
H	-6.178231528898	-2.409730443950	-0.250157958467
H	-1.760767506823	-2.796773168168	1.907599389863
H	-5.202088079737	-4.604601222050	0.482741399089
H	-2.022261559965	-0.694181690317	3.924460537941
H	0.100749785620	-0.689586169730	5.180055039822
H	2.246474056194	-0.187238032898	4.057249715682
H	3.352075103710	-0.446961152587	1.594074661048
H	3.487453292022	0.830031376753	-0.540920488281
H	1.339805050019	0.716593544707	-1.784187622170
H	-0.736212484827	0.217283180325	-0.588688827994
H	3.130815972520	1.226423514467	1.893244138857
H	-3.097294054148	-4.607818744150	1.477726652212

9a-TS-ring_closure.xyz

35

SCF=-787.127103672 ; NI=1 ; Neg-freq =-261.6618

C	0.040254775545	0.005922548065	-0.005454078271
C	0.014089453139	0.022767968009	1.725524627746
C	1.474646019991	0.003804999681	2.050446138655
C	2.317228913798	-0.912634653930	1.375946426381
C	1.721002772489	-1.770358554652	0.334200231316
C	0.571144593109	-1.318613021453	-0.330016438579
C	2.016071298825	0.914470701039	2.969388289104
C	1.164777922048	1.963759985554	3.621637507828
C	-0.244201117809	2.006816617344	3.161359535504
C	-0.772936785711	1.128653093915	2.288338895882
C	3.675768747227	-0.952342773617	1.679929243907
C	4.203854224429	-0.082513533407	2.639631885510
C	3.384673940507	0.845082554726	3.268525304293
C	-0.142462316008	-2.189571701103	-1.232755730084
C	0.406459053808	-3.459967908312	-1.570628844009
C	1.581838114990	-3.850649260808	-0.981466919552
C	2.214002457519	-3.028511579876	-0.017843955680
N	-1.192078949794	0.319745683754	-0.603519764297
C	-1.898218949684	-0.499868091596	-1.346977465999
C	-1.381686304436	-1.772117613220	-1.698471698009
H	-0.129168017252	-4.105884612113	-2.258144128602
H	2.016167830935	-4.818000069944	-1.211293936855
H	3.100888497240	-3.410462374346	0.479157137211
H	-1.980413396777	-2.404825249857	-2.345809701803
H	0.740143563257	0.828561578284	-0.180383818179
H	-2.856400638602	-0.147022512335	-1.710960490264
H	4.338085008401	-1.633996793242	1.154731592121
H	5.264831236535	-0.115841243523	2.869502911090
H	3.806989932125	1.543732921578	3.986654166304
H	1.627094377285	2.953609534915	3.480289570768
H	-0.888404771160	2.756576616550	3.614973015085

H	-1.835709859516	1.163169787677	2.061530596609
H	-0.482007914292	-0.936540151210	1.909271485594
H	1.167696584859	1.826678998505	4.715520142254
H	-1.545823460066	1.266762922533	-0.467144352228

Intermediary_for_ring_closure_from_9a_Table_3.xyz

35

-787.127577277

C	0.076721423814	0.040436332351	-0.043291759898
C	0.018519779360	-0.046840326703	1.576454139358
C	1.468616226032	-0.015873937584	2.002442678861
C	2.406836456155	-0.796411791144	1.289085973826
C	1.930332338192	-1.576008016932	0.132288668415
C	0.764061756790	-1.176203394635	-0.518928005189
C	1.892087439305	0.779438400214	3.073556086883
C	0.942453731656	1.681136470520	3.810492490186
C	-0.423848552846	1.750959916650	3.228123683906
C	-0.842596160037	0.986113330521	2.209445760850
C	3.747407802393	-0.802803489533	1.678365673745
C	4.161029617104	-0.037146157571	2.770211466216
C	3.242883933106	0.748320224432	3.453706793916
C	0.197154545085	-1.988101250380	-1.570104187478
C	0.894530686205	-3.149154555148	-2.034260405130
C	2.071564723564	-3.496502137669	-1.425871999801
C	2.565607351732	-2.734821388442	-0.340428488428
N	-1.192647234586	0.265291602060	-0.657276635711
C	-1.741318134131	-0.500144084913	-1.555293306289
C	-1.049242127571	-1.649627993735	-2.055637172101
H	0.464733304232	-3.744597713533	-2.832926573917
H	2.618128920528	-4.378748014249	-1.741299885693
H	3.465452583791	-3.083777929816	0.157075511616
H	-1.541808775948	-2.241513152012	-2.820512921891
H	0.683905143638	0.939935581457	-0.230011873432
H	-2.719743363135	-0.219102075042	-1.929949322452
H	4.481499191520	-1.379874639963	1.124181912033
H	5.205439818780	-0.041644416483	3.068221038095
H	3.571426406386	1.367158755130	4.285404850703
H	1.371058033559	2.692753305279	3.871688407368
H	-1.126661494692	2.432406442210	3.702544692706
H	-1.883085070138	1.039754385798	1.896959292622
H	-0.421528005036	-1.038518015690	1.754566699630
H	0.859656470504	1.358746443419	4.861130963521
H	-1.686854810181	1.116988776463	-0.390759950617

25.xyz

50

-1154.87976326

C	2.564858655928	1.754381628561	-0.469488469467
C	2.615333008772	2.557992311394	-1.640932908284
C	1.754153076080	2.341634457834	-2.693348304482
C	0.793711655648	1.306943915687	-2.622778480719
C	0.722326763727	0.504611770828	-1.506034476657

C	1.602180862296	0.692258447896	-0.405357411906
C	1.543406670798	-0.140481124321	0.758108095001
C	2.415198972646	0.104461134139	1.800436036504
C	3.360813679235	1.151142762995	1.738279436188
C	3.448567007245	1.976450297446	0.635067135626
C	4.470244923984	3.065057693556	0.609755496838
C	0.541099152643	-1.240336077827	0.879912963598
C	0.726400813025	-2.495342629131	0.214144008528
C	1.863827518505	-2.771959443000	-0.591939523160
C	2.013587632655	-3.991472160142	-1.215261081690
C	1.031978330895	-4.999544050755	-1.063897240120
C	-0.078886670017	-4.766586977972	-0.284930894060
C	-0.262204395288	-3.520913658055	0.373677307953
C	-1.397784231603	-3.275445215778	1.189771318215
C	-1.548806535488	-2.067430955435	1.829728243691
C	-0.576126744572	-1.052791065508	1.672869209915
C	5.131131011987	5.431305290715	0.869125940808
C	4.776919533889	6.779026116279	1.147141303850
C	3.475597991590	7.125012005919	1.433635610845
C	2.468271177343	6.131394273311	1.456514332401
C	2.778939633394	4.816100499173	1.189069439397
C	4.111096843192	4.424552540258	0.885031562168
C	5.791382073157	2.752982480924	0.347474119256
C	6.795605341893	3.748865274656	0.332377996446
C	6.472259477338	5.061644294277	0.584805466214
H	3.350874650800	3.355728447523	-1.697274839383
H	1.810866431201	2.968792929149	-3.579725116671
H	0.110848313541	1.144281718370	-3.453095097512
H	-0.017970609845	-0.289126451531	-1.456803504971
H	2.376711448790	-0.523611481319	2.687482784563
H	4.027975400226	1.313724623634	2.581624401235
H	2.624365396636	-2.005109656131	-0.711406288907
H	2.892052449201	-4.182626479894	-1.826870027403
H	1.160132909282	-5.957931337154	-1.561074451066
H	-0.835306088547	-5.538864232989	-0.159531820883
H	-2.143460307083	-4.059638177503	1.303292926720
H	-2.417252716398	-1.882946233958	2.457280516426
H	-0.712759356222	-0.100084712622	2.179431645997
H	5.557692069136	7.536939854253	1.132287375370
H	3.218965137182	8.160037078087	1.645768723665
H	1.442814529826	6.409258314938	1.687952360463
H	1.999062373587	4.059829796163	1.212260460166
H	6.061225406155	1.719670465776	0.141602427077
H	7.823844585596	3.467821825784	0.118355765279
H	7.239587656325	5.833027585599	0.573527347877

25a-Sigma-5.xyz
51
-1155.27187505

C	0.092400673381	-0.144358255259	0.103119899835
C	0.091253705660	0.413785686494	1.422822059412
C	1.252415291305	0.970591681276	1.926614834889
C	2.449788117522	0.983212952942	1.173883526072

C	2.479177317614	0.438912026774	-0.089010950516
C	1.308445558762	-0.131271642377	-0.655443811962
C	1.313243658774	-0.682365128963	-1.965325073736
C	0.169015210655	-1.217910858189	-2.512685266153
C	-1.035615481520	-1.220767000532	-1.769962779168
C	-1.072192193582	-0.697533514616	-0.495809797426
H	-2.004438393473	-0.701342319354	0.062446174579
H	-1.938414272552	-1.637370042190	-2.209913969413
H	0.186091752674	-1.634904134106	-3.516536564536
H	2.241922185506	-0.669854513118	-2.532209244751
H	3.397564940978	0.442313031618	-0.672342350861
H	3.346043383599	1.423386636099	1.603682436510
H	1.246549698611	1.397793769288	2.926743805082
C	-1.149572274815	0.433883552491	2.251246194185
C	-1.836053936918	1.620704937008	2.415767715688
C	-3.017964636403	1.682859936608	3.180932400858
C	-3.536820773480	0.555874418190	3.794829947386
C	-2.834122914190	-0.688998429595	3.685961807096
C	-1.631692220695	-0.746411756598	2.905175100804
C	-0.930846568698	-1.979412997764	2.823759895785
C	-1.383239378783	-3.103652858770	3.478016521464
C	-2.559237458589	-3.043615224200	4.257589406354
C	-3.262594619488	-1.863652910298	4.361229108715
H	-4.154209686371	-1.825981671305	4.979994502992
H	-2.905523751500	-3.928291614840	4.785723329399
H	-0.829080415690	-4.035824931215	3.403834413098
H	-0.015691606392	-2.024382301376	2.240349351634
C	-4.838922923318	0.659211234251	4.498041017704
C	-5.944656026540	-0.040172711942	4.033005031306
C	-7.205441881727	0.131541951473	4.621017915853
C	-7.395564608036	1.005344300400	5.688784679097
C	-6.313780165005	1.712202068017	6.201480880682
C	-5.016945861491	1.527811243333	5.626865104280
C	-3.919505037115	2.164359123209	6.238364370991
C	-4.033394459478	3.038826558544	7.337047089499
C	-5.267724706498	3.289056423231	7.869728400257
C	-6.481816350652	2.650684837608	7.347430577234
H	-7.197581130616	3.449935532493	7.080378499765
H	-6.997731126371	2.149702800074	8.185128142178
H	-5.379900037385	3.970587844968	8.708956102117
H	-3.140973524579	3.498609901257	7.748004800045
H	-2.926037887856	1.962613194017	5.846716712670
H	-8.384346867936	1.133818910904	6.120235425685
H	-8.054514629849	-0.416331306341	4.221562476570
H	-5.839339871722	-0.702724160452	3.178974623167
H	-3.551200539625	2.628018997341	3.249112271471
H	-1.474753344646	2.522278344830	1.927848459419

25a-TS1_from_Sigma-5.xyz

51

SCF=-1155.24565251 ; NI=1 ; Neg-freq =-376.4118

C	-3.059188288578	1.061153904290	-0.625007092658
C	-1.600272956036	1.173621849170	0.806944132774

C	-0.773797048841	0.106433842913	0.264045579781
C	-1.343149474750	-1.150312794547	-0.069797283065
C	-2.804700684535	-1.328479997666	0.056764352840
C	-3.648524517252	-0.212594299363	-0.186802583847
C	-0.509324996656	-2.139651692092	-0.572281384400
C	0.864189969443	-1.911213916016	-0.766512097169
C	1.432648922876	-0.669882796193	-0.535683742256
C	0.589442529049	0.379200053432	-0.049972148533
C	-1.067939143549	2.474300047357	0.918880124293
C	0.226319849513	2.755721952805	0.485315617039
C	1.044746210910	1.720850995639	0.052808977684
C	-5.030579829823	-0.301671721727	0.045477472065
C	-5.578831106067	-1.535083312862	0.423813115385
C	-4.761999099419	-2.642861271189	0.609774494319
C	-3.375570744151	-2.535818921241	0.448164182953
C	-3.885749681373	2.244722814546	-0.650105058040
C	-5.213242970901	2.168226330919	-0.413305359613
C	-5.916267268270	0.901512808588	-0.097281491591
C	2.869581557622	-0.441636988859	-0.854793846099
C	3.838555935032	-0.217863288258	0.178117066004
C	5.208068445176	-0.012564445757	-0.192481335483
C	5.574841008115	-0.045653553733	-1.563717891885
C	4.630106637618	-0.281906036590	-2.535080323520
C	3.277736949704	-0.482104894767	-2.176134592794
C	3.505638104071	-0.214814620603	1.560382697169
C	4.468402334185	-0.001147900962	2.522727501093
C	5.816418405549	0.218496479539	2.153639379067
C	6.175198709214	0.208870731639	0.824616186478
H	2.067352005126	1.942322837989	-0.239706570878
H	0.614933306987	3.768103128325	0.535184248476
H	-1.674251496318	3.254964762303	1.370165739740
H	-2.363291638405	0.911832168816	1.535356696156
H	-0.925072304478	-3.103919502232	-0.852207346170
H	1.485171946525	-2.718355657255	-1.145613605051
H	-2.302464481533	1.011675924182	-1.404587614693
H	-3.431290566242	3.183807337802	-0.951768330171
H	-5.827281412254	3.059941908462	-0.518536288517
H	-6.506496078013	1.035200709939	0.823187804838
H	-6.650645855892	-1.613549497038	0.589650240198
H	-5.195769088649	-3.591554352955	0.913941269508
H	-2.741825608949	-3.392451231201	0.660403756426
H	7.211094183202	0.367679040518	0.532370684606
H	6.566576646395	0.387750755447	2.921958731055
H	4.191634866914	-0.005248579687	3.574089528991
H	2.476934945940	-0.391938432936	1.861675573401
H	2.539699157557	-0.656894505316	-2.955359907959
H	4.914362044894	-0.311361429414	-3.583751872872
H	6.616802474894	0.114014292274	-1.832669295703
H	-6.678274805581	0.725525963564	-0.874100089371

Intermediary_for_the_formation_of_26_from_Sigma5_ring_closure-1.xyz

51

-1155.26001795

C	0.052654401086	0.033596296033	-0.036953562940
C	0.001849888732	-0.023010872272	1.541493602921
C	1.398412324577	-0.022219010040	2.064090792641
C	2.367975943704	-0.779119675746	1.392981603149
C	1.971676466613	-1.573594958330	0.215578301697
C	0.814017490380	-1.194627827045	-0.500839557968
C	3.680018597146	-0.733259650730	1.879100240418
C	4.028272652195	0.001116365203	3.019559195207
C	3.087346025891	0.747113312920	3.712520542191
C	1.748273470702	0.759331294574	3.203077684174
C	-0.923719575083	0.907654609046	2.201222565498
C	-0.553697244914	1.672982765762	3.274079788670
C	0.762915104241	1.595035776274	3.762754748575
C	0.398913692346	-1.948975131927	-1.604232108171
C	1.151594760974	-3.066774185056	-1.990623096186
C	2.295493729293	-3.438570767991	-1.292226390029
C	2.702086281927	-2.697389952590	-0.184924584179
C	-1.283764370818	0.194552617716	-0.696745667400
C	-1.673262618824	-0.527540040745	-1.751481054527
C	-0.848020453561	-1.602862786684	-2.378473304157
C	3.490090057051	1.534969173574	4.908054806085
C	3.066633433521	1.160447938689	6.225431745178
C	3.493051108824	1.959938061607	7.336320602563
C	4.327316976427	3.085821438317	7.109326185936
C	4.738169461908	3.409631505029	5.837146679588
C	4.320282165917	2.628393905023	4.736487119275
C	2.264743823395	0.015347788846	6.484644870807
C	1.886716734836	-0.309586742922	7.769216251514
C	2.291757055532	0.492056067263	8.862163456678
C	3.080723664543	1.599433433469	8.647157286004
H	1.037140606309	2.221375797574	4.607973902692
H	-1.264536913913	2.335545688190	3.756519510507
H	-1.951156401635	0.937288196072	1.850436443383
H	-0.434940383206	-1.022332712169	1.758803959199
H	4.464395808909	-1.274575507843	1.358039955522
H	5.057668503932	-0.015110616431	3.365462378332
H	0.651384615329	0.931465286757	-0.262484792135
H	-1.923008045299	0.999439799395	-0.341664134847
H	-2.633713497910	-0.313272090090	-2.217261568530
H	-1.457693152676	-2.507527515269	-2.514933439695
H	0.822306262900	-3.656093750331	-2.844124423931
H	2.862182906204	-4.314232897220	-1.597398113425
H	3.575446553519	-3.014279919632	0.378042036708
H	3.406070775419	2.215142288515	9.482990748082
H	1.984347569826	0.226484896153	9.870354540291
H	1.275408739272	-1.191143537354	7.944961150047
H	1.953605803664	-0.620147041108	5.660083104238
H	4.643175086900	2.901270699866	3.734776111228
H	5.380944263593	4.269354750408	5.667998522695
H	4.641936104851	3.685714167516	7.960486432693
H	-0.576262220192	-1.293262326109	-3.400235077938

25a-TS2_from_Sigma-5.xyz

51

SCF=-1155.23467390 ; NI=1 ; Neg-freq =-374.9025

C	-2.731531531853	1.048114294453	-0.730138569354
C	-1.436081289732	1.187398945857	0.137725890936
C	-0.660048936176	-0.093849712730	0.021547984026
C	-1.371640185187	-1.307845115732	0.128378175652
C	-2.839079853956	-1.259127711696	0.280731415262
C	-3.534591217552	-0.103633778882	-0.149172191142
C	-0.650497053407	-2.505326103777	0.083703935373
C	0.741835890743	-2.511914628348	-0.015850312483
C	1.451254606312	-1.322596378853	-0.157429109551
C	0.722560935676	-0.106657632858	-0.199389342630
C	-0.622687616126	2.409469086901	-0.085407452028
C	0.707284104636	2.395655550666	-0.324570093043
C	1.428302659090	1.154517564889	-0.449027748791
C	-4.927469180574	-0.038649985662	-0.015184978531
C	-5.616116471520	-1.126460034462	0.538125797654
C	-4.936881888658	-2.264336533748	0.962079690470
C	-3.551256241343	-2.327913568393	0.839806668164
C	-3.528972822612	2.312253527670	-0.872036606800
C	-4.858564659136	2.366093043563	-0.754081035070
C	-5.711181660968	1.180518067952	-0.436810869515
C	2.925185089653	-1.268559026819	-0.230289581556
C	3.592528187148	-0.134442327310	0.308390920764
C	4.982399866199	0.047915722395	0.054682656905
C	5.717759593207	-0.979424143701	-0.588117900678
C	5.074542914636	-2.122316181575	-1.012130044346
C	3.679789884116	-2.249029890338	-0.864779952434
C	2.866307541415	0.912650564449	1.008993398173
C	3.528277602797	2.114197461099	1.334815966828
C	4.863752147898	2.319400805931	0.985766467904
C	5.584043384222	1.286651955361	0.401576092317
H	2.200576816158	1.142371189940	-1.213966854996
H	1.242114705614	3.323139136640	-0.506856055589
H	-1.134467109401	3.367337319366	-0.045564433806
H	-1.797713695286	1.260934420064	1.182190356838
H	-1.170802171674	-3.456711950286	0.140968510806
H	1.271050223943	-3.459785647670	0.027448984193
H	-2.380213349987	0.761932732220	-1.737081128128
H	-2.988838229198	3.211726442739	-1.158435146028
H	-5.373451700033	3.310652006672	-0.923190721542
H	-6.434763748670	1.440265492448	0.348779855224
H	-6.697512596907	-1.068397751217	0.646751957444
H	-5.482357350507	-3.095410492674	1.401778651696
H	-3.024419990954	-3.205534135540	1.203401426997
H	6.640203259649	1.426074375292	0.182188941533
H	5.353942560744	3.258612017750	1.222369192766
H	2.991672151868	2.873039990718	1.897756873969
H	2.056726459350	0.627388801214	1.675422743816
H	3.181659890641	-3.114206935795	-1.293946022915
H	5.630922584117	-2.910023878357	-1.511997888080
H	6.780751305419	-0.839798972734	-0.765596463599
H	-6.329233813833	0.937242002912	-1.316258053073

Intermediary_for_the_formation_of_26_from_Sigma5_ring_closure-2.xyz

51
-1155.25034441

C	-2.724734285161	1.072255101115	-0.638782009266
C	-1.467933745135	1.177892288271	0.276751472754
C	-0.663157715004	-0.087178860989	0.093824680739
C	-1.369479164090	-1.309292093962	0.133588792934
C	-2.840755299005	-1.277098382953	0.259371021050
C	-3.536961900391	-0.112305020284	-0.143887843965
C	-0.651787743064	-2.508186469685	0.038007468731
C	0.733107494675	-2.503197088306	-0.068737791828
C	1.436291937279	-1.296187942261	-0.147363318774
C	0.719087132468	-0.080885252785	-0.096302116908
C	-0.649287481315	2.421018945418	0.130076842767
C	0.671065251356	2.438564955217	-0.075339413639
C	1.492957267293	1.207052429872	-0.285056689433
C	-4.934345047517	-0.068444680197	-0.052815611026
C	-5.627523293112	-1.183389301308	0.437324309853
C	-4.947649372682	-2.329269982080	0.839393136065
C	-3.558292112976	-2.373952982129	0.755312677647
C	-3.533567533154	2.333559200413	-0.741827082263
C	-4.867249601423	2.369955566361	-0.671525297914
C	-5.717255451974	1.158420663455	-0.455517668879
C	2.906100003252	-1.249070339970	-0.256575173618
C	3.576951439548	-0.076496809636	0.125207277851
C	4.993756842615	0.003597630106	0.025655486283
C	5.743564803006	-1.113479907081	-0.422764277175
C	5.079051867973	-2.265212507202	-0.790379386313
C	3.678353423016	-2.323575706257	-0.721651932361
C	2.789493948666	1.096291478432	0.600235196546
C	3.556809060345	2.330255827970	0.822195914910
C	4.922194048314	2.378292720138	0.716531998926
C	5.627328045930	1.223811207987	0.332422844143
H	1.870589766872	1.234339114049	-1.320628151686
H	1.178023223639	3.396722400545	-0.168680999179
H	-1.167964673432	3.370078249838	0.245178296912
H	-1.861127187719	1.178590556642	1.309727058203
H	-1.172867173786	-3.460912481159	0.046518651157
H	1.261496340810	-3.451973080839	-0.088825609394
H	-2.343012600256	0.841598575829	-1.650231463296
H	-2.990463163166	3.253196386879	-0.948115884351
H	-5.387036883525	3.318205353642	-0.802148492040
H	-6.483541886466	1.369056543533	0.303845603020
H	-6.712507954033	-1.141123728016	0.514354535333
H	-5.496353043921	-3.182099354617	1.231240504649
H	-3.032441687183	-3.260316635608	1.098963084328
H	6.711333990466	1.274956019963	0.254597595569
H	5.464641408391	3.293120449714	0.930504777986
H	3.011800772767	3.211686842909	1.148656369322
H	2.408989822849	0.855700098061	1.618281632005
H	3.184238121547	-3.230480801773	-1.057442069752
H	5.631435314176	-3.126646640427	-1.152706727314
H	6.825163370399	-1.041309349260	-0.489611131591
H	-6.286319698160	0.948486792424	-1.376013087717

25a_Sigma-4.xyz

```
51
-1155.27653235
 C        0.173636879844      0.123664814701     -0.194279180399
 C       -0.117205290142     -0.209669868466      1.169755448269
 C        0.906648356680     -0.623702606520      2.005389803723
 C        2.241589256741     -0.699085636849      1.551742388878
 C        2.549251689060     -0.365533326016      0.252924500120
 C        1.531967669273      0.045553153763     -0.647485512074
 C        1.833416594821      0.374140687170     -1.996579169955
 C        0.841163099932      0.752420792262     -2.872393635150
 C       -0.502566738944      0.809201828258     -2.433767141245
 C       -0.827616677580      0.501582900672     -1.130412746437
 H       -1.865792710354      0.541459444205     -0.813783786450
 H       -1.285621976738      1.093906569505     -3.132139707575
 H        1.085017639443      1.000574992707     -3.902273309454
 H        2.868273710237      0.316064335584     -2.327379717545
 H        3.576134237068     -0.416060228021     -0.102554485163
 H        3.022070363535     -1.014732681855      2.239067333519
 H        0.682265439753     -0.872693246132      3.039788402856
 C       -1.487362045221     -0.061114004319      1.728832706363
 C       -2.051199877841      1.203277489620      1.808095751646
 C       -3.299833567947      1.409011355956      2.406787287214
 C       -4.012713476864      0.352801166235      2.975318351836
 C       -3.497296141238     -0.985960670101      2.853998861496
 C       -2.226091028162     -1.187868756085      2.221263893303
 C       -1.751670650417     -2.515149105348      2.052233735676
 C       -2.486951632901     -3.597531126944      2.484498667002
 C       -3.744574241868     -3.398425447330      3.088382929613
 C       -4.242382863214     -2.122504702079      3.255254998215
 H       -5.235212797517     -1.995004626951      3.675899184580
 H       -4.335702123013     -4.253050518652      3.406280023961
 H       -2.107378581695     -4.605326688327      2.339701272831
 H       -0.800569329570     -2.672470861748      1.553690956996
 C       -5.176700477546      0.683264858130      3.788237088259
 C       -5.235825361566      0.179141217664      5.121839084136
 C       -6.159291759633      0.643664571205      6.006273545204
 C       -7.177056383755      1.639194958488      5.639769068494
 C       -7.195090325390      2.049855287312      4.206378705194
 C       -6.205273884452      1.564237624517      3.304283031867
 C       -6.296792588928      1.909747930618      1.930805499483
 C       -7.295025723621      2.752837171217      1.482639777738
 C       -8.235641420913      3.264229828721      2.389649541437
 C       -8.190144763864      2.905786581447      3.733026076830
 H       -8.940415706417      3.285783773939      4.421736661338
 H       -9.020968354015      3.927481769546      2.037473004247
 H       -7.360785698363      3.005306226435      0.428606157624
 H       -5.595121513291      1.482512194457      1.222832374277
 H       -8.170133660279      1.265570057660      5.940545128486
 H       -7.040578210286      2.520921573996      6.291346581190
 H       -6.130950282603      0.310938423511      7.040743489812
 H       -4.465057212339     -0.508206222693      5.453970950737
 H       -3.668401132757      2.424874287233      2.512230224911
```

H   -1.497073185765   2.061790047397   1.439532609827

25a_TS_ring_closure1_from_Sigma-4.xyz

51

SCF=-1155.21192092 ; NI=1 ; Neg-freq =-454.0212

C	3.721591723671	0.084711977916	-0.415611164755
C	2.927691927126	0.830516962725	0.514011761000
C	3.539778688335	1.480499066409	1.569660123109
C	4.939904309308	1.416002861743	1.758330887491
C	5.722873459482	0.697153905672	0.885493657063
C	5.139458993403	0.018489624612	-0.217024626190
C	5.931910054648	-0.721088106742	-1.135332142007
C	5.356970297412	-1.367321873775	-2.206766215765
C	3.958242060998	-1.296895314884	-2.408297044925
C	3.161288888389	-0.588495450327	-1.535366578644
H	2.089139138940	-0.536156214262	-1.706067595207
H	3.510591845843	-1.803705966457	-3.259601552914
H	5.975187583656	-1.929516166796	-2.902098365775
H	7.007448481768	-0.766286509650	-0.977261390764
H	6.800063737819	0.640911363054	1.026949792909
H	5.389639633000	1.936451220853	2.599913940597
H	2.931841822270	2.046831051536	2.271234629082
C	1.448669169806	0.938956321984	0.352258326414
C	0.892800732740	2.065407195883	-0.230451906081
C	-0.489062532516	2.174794346680	-0.445883177784
C	-1.361596997145	1.170548480271	-0.036178425731
C	-0.832167162702	0.047109708354	0.652593802333
C	0.574859745986	-0.102532125130	0.792287177418
C	1.071921845728	-1.329442870063	1.316993701312
C	0.235508723101	-2.367430359238	1.711709925750
C	-1.140914792890	-2.199738253242	1.675471834588
C	-1.733576156818	-1.012744002642	1.144834376948
H	-2.559317881421	-0.664253802975	1.768992240744
H	-1.794423464053	-2.965160605887	2.085054769196
H	0.665296203807	-3.285413833984	2.101000012032
H	2.147723193360	-1.458581651934	1.393431067182
C	-2.809268636329	1.220057314889	-0.288762292359
C	-3.491278514700	2.493170599788	-0.329201032131
C	-4.834377369863	2.558412440885	-0.439057955752
C	-5.684254576599	1.343754019452	-0.535906485802
C	-4.951542949517	0.032776989459	-0.432290877873
C	-3.509425163525	0.028090981676	-0.410692629614
C	-2.770100322034	-1.263194525758	-0.331604248276
C	-3.581004657814	-2.470143196612	-0.275228652590
C	-4.941227694075	-2.402184989671	-0.278705328867
C	-5.634588964844	-1.154335044121	-0.346503460773
H	-6.721631106155	-1.153133709615	-0.338043386971
H	-5.523157300382	-3.320627631529	-0.268238884553
H	-3.075287625172	-3.431452703815	-0.273147051492
H	-1.946721677344	-1.348194059053	-1.044265122954
H	-6.488822349463	1.400568192748	0.211586301126
H	-6.217779494707	1.378678199074	-1.501526354164
H	-5.336240942213	3.522653148899	-0.456015198044

H	-2.908464912048	3.403510229790	-0.231147783425
H	-0.868060289245	3.044089347394	-0.974641228758
H	1.545517272977	2.864252416418	-0.571967165354

Intermediary_for_the_formation_of_26_from_Sigma4_ring_closure-1.xyz

51

-1155.21720643

C	3.763574214556	0.098928725894	-0.399990143403
C	2.946074740009	0.813331257619	0.534708578560
C	3.532891472536	1.457848297206	1.608478336486
C	4.930127647528	1.409458597531	1.817240415224
C	5.735342900795	0.714990460766	0.944855652227
C	5.178962016558	0.049035241400	-0.178819246802
C	5.997716368384	-0.659965845181	-1.098031537096
C	5.451555034264	-1.288014552210	-2.194788088276
C	4.056718210465	-1.226978829059	-2.422848465807
C	3.233956674965	-0.550320008146	-1.548725505203
H	2.166271926044	-0.503475373148	-1.745980143293
H	3.631809575951	-1.714593339067	-3.296619422704
H	6.089684843032	-1.826349431024	-2.890875291689
H	7.070471779151	-0.694979778917	-0.920350520947
H	6.810771017382	0.670013225136	1.102287218631
H	5.359624021783	1.921045552216	2.674510617378
H	2.906261395261	2.002193916579	2.310826474484
C	1.469888296485	0.905809624346	0.359155738426
C	0.888496721005	2.078683112730	-0.096646098517
C	-0.496037492970	2.189761374280	-0.276316929495
C	-1.371518596809	1.132833989788	0.009929492337
C	-0.809939743146	-0.061031043246	0.496102732152
C	0.599158386899	-0.186394130357	0.667351565606
C	1.102750022405	-1.421077367788	1.127369331540
C	0.293558898826	-2.535223859317	1.404852737504
C	-1.064586228181	-2.440695879057	1.253025589684
C	-1.717478360227	-1.186782343478	0.858146139603
H	-2.237986244566	-0.883864402998	1.794761255331
H	-1.695241407028	-3.288008500773	1.505183285672
H	0.751457994416	-3.456469550461	1.749388552094
H	2.176308722501	-1.514807405716	1.270766929830
C	-2.825598988546	1.220773542295	-0.163689467974
C	-3.482291392683	2.524408541495	-0.180969914234
C	-4.799062131558	2.645086301274	-0.417261410400
C	-5.655813168983	1.458479640612	-0.725402960920
C	-5.005523128333	0.127736452570	-0.422843962558
C	-3.563942175371	0.066257673135	-0.273539942950
C	-2.869829240254	-1.284609167306	-0.206535156023
C	-3.797739372897	-2.445146728162	-0.002988684372
C	-5.134920106298	-2.298086053555	-0.068663893094
C	-5.747540304739	-1.008340993078	-0.294103341415
H	-6.831847152122	-0.953282464059	-0.361292641629
H	-5.779831894604	-3.168357032979	0.027249266592
H	-3.364000012765	-3.435210092624	0.102699201017
H	-2.345890214059	-1.452074841571	-1.165374331177
H	-6.620533415748	1.538661924862	-0.208747947412

```
 H        -5.910858219526      1.493169614068     -1.799559926958
 H        -5.270857629294      3.624878410428     -0.419217997826
 H        -2.890695994180      3.410108434212      0.031926560406
 H        -0.887595558586      3.122103533325     -0.671043634813
 H         1.521766292271      2.925830569509     -0.344568063797
```

OXIDATIVE AROMATIC COUPLING

8.xyz

```
35
SCF = -786.793209718
 C        -0.007155257129     -0.090923837031      0.003866877349
 C         0.011065144528     -0.018476417956      1.386380342314
 C         1.252049365882      0.015413182267      2.100535170429
 C         2.465271420564      0.004399255552      1.339081426130
 C         2.403807291340     -0.049991511114     -0.078007964433
 C         1.195883826773     -0.102503869513     -0.733779562268
 C         3.710064985480      0.033284942887      2.021890023193
 C         3.760620076237      0.059952195961      3.396772764102
 C         2.562916961498      0.051708387635      4.148720083168
 C         1.338456487798      0.028247859076      3.517872842623
 C        -1.286867828563      0.006532131526      2.099573582742
 C        -1.814637755614      1.123308058362      2.793840716451
 C        -3.097707601563      1.000569211255      3.423242425174
 C        -3.803114692890     -0.226283590225      3.312807190760
 C        -3.259329580838     -1.261330600262      2.612403138256
 N        -2.028173183082     -1.107730097024      2.038935323229
 C        -3.629179370271      2.111685719833      4.118636121305
 C        -2.924603425878      3.294326893219      4.174159814585
 C        -1.668035166117      3.421339786933      3.534934423718
 C        -1.118878203751      2.359332683147      2.855690324619
 H        -4.598411441566      2.020260380966      4.600590096825
 H        -3.339244723221      4.144794435781      4.708006241074
 H        -1.137138933879      4.367582968166      3.579808615868
 H        -0.158483979397      2.458777702403      2.360575151238
 H        -4.776940848143     -0.344112361114      3.775819811537
 H        -3.731130408083     -2.225738604644      2.475082174099
 H         0.431322321395      0.012780199427      4.115997260188
 H         2.609023911480      0.060626669022      5.234528247099
 H         4.718954700789      0.080544038321      3.908607570581
 H         4.626335227061      0.028587204969      1.435967597339
 H         3.333950456013     -0.055107141220     -0.641383902817
 H         1.156819235882     -0.145995314134     -1.818288536355
 H        -0.957082377731     -0.110053582261     -0.524987228762
 H        -1.633159988259     -1.901192679650      1.539731602959
```

8-rc.xyz (rc= radical cation)

```
34
SCF = -786.53997828
 C        -0.007155257129     -0.090923837031      0.003866877349
 C         0.011065144528     -0.018476417956      1.386380342314
```

C	1.252049365882	0.015413182267	2.100535170429
C	2.465271420564	0.004399255552	1.339081426130
C	2.403807291340	-0.049991511114	-0.078007964433
C	1.195883826773	-0.102503869513	-0.733779562268
C	3.710064985480	0.033284942887	2.021890023193
C	3.760620076237	0.059952195961	3.396772764102
C	2.562916961498	0.051708387635	4.148720083168
C	1.338456487798	0.028247859076	3.517872842623
C	-1.286867828563	0.006532131526	2.099573582742
C	-1.814637755614	1.123308058362	2.793840716451
C	-3.097707601563	1.000569211255	3.423242425174
C	-3.803114692890	-0.226283590225	3.312807190760
C	-3.259329580838	-1.261330600262	2.612403138256
N	-2.028173183082	-1.107730097024	2.038935323229
C	-3.629179370271	2.111685719833	4.118636121305
C	-2.924603425878	3.294326893219	4.174159814585
C	-1.668035166117	3.421339786933	3.534934423718
C	-1.118878203751	2.359332683147	2.855690324619
H	-4.598411441566	2.020260380966	4.600590096825
H	-3.339244723221	4.144794435781	4.708006241074
H	-1.137138933879	4.367582968166	3.579808615868
H	-0.158483979397	2.458777702403	2.360575151238
H	-4.776940848143	-0.344112361114	3.775819811537
H	-3.731130408083	-2.225738604644	2.475082174099
H	0.431322321395	0.012780199427	4.115997260188
H	2.609023911480	0.060626669022	5.234528247099
H	4.718954700789	0.080544038321	3.908607570581
H	4.626335227061	0.028587204969	1.435967597339
H	3.333950456013	-0.055107141220	-0.641383902817
H	1.156819235882	-0.145995314134	-1.818288536355
H	-0.957082377731	-0.110053582261	-0.524987228762
H	-1.633159988259	-1.901192679650	1.539731602959

9.xyz

34

SCF = -786.791411722

C	-0.042538722433	-0.095266506086	0.062883343917
C	-0.011961330375	-0.026544144010	1.462765261030
C	1.271123425546	-0.002062449427	2.104438856966
C	2.435425972478	-0.074771762473	1.294806227124
C	2.329548884521	-0.155378795743	-0.063519376937
N	1.087821235629	-0.158712976361	-0.634907857549
C	-1.216527091290	0.037174749141	2.234880677446
C	-1.097189628319	0.146342401403	3.605890478223
C	0.167657640439	0.179006119802	4.239747835559
C	1.334235115956	0.100614769513	3.511979265199
C	-2.556176382892	0.015310020217	1.582530184337
C	-3.101617392363	-1.201173538792	1.058075320223
C	-4.387718777190	-1.161039952089	0.426901228032
C	-5.091708882980	0.069445031902	0.352235245306
C	-4.554736487506	1.218837100510	0.883304728603
C	-3.283101005002	1.188785252265	1.499534535368
C	-4.936301728262	-2.359877872974	-0.101773827542

C	-4.256216328630	-3.552724193549	-0.005273916731
C	-2.995086546715	-3.597643157347	0.633835383249
C	-2.432454888114	-2.452024875978	1.152763797491
H	2.302089051703	0.119790706267	4.004454814912
H	0.211282491174	0.261596275475	5.322143685556
H	-1.996356418553	0.196856435583	4.213361676819
H	3.419215563585	-0.065307177302	1.751919065673
H	-0.965731617267	-0.095175416328	-0.504213993892
H	3.171101470791	-0.213989516423	-0.741415880403
H	-2.864044108928	2.106366301576	1.905547527040
H	-5.099843603813	2.157333769993	0.829366018883
H	-6.067642637645	0.087028156184	-0.127843641381
H	-5.911459783081	-2.318577995027	-0.582195676107
H	-4.688650111223	-4.463382293557	-0.411531830585
H	-2.469233158886	-4.545076684218	0.720952878416
H	-1.469872934060	-2.508397881459	1.654147696349
H	1.024290082277	-0.210101783899	-1.648109447260

9-rc.xyz

34

SCF = -786.54757818

C	-0.040076329500	-0.201035067689	0.052793275262
C	-0.021388296656	-0.074798809035	1.450184927602
C	1.257557582732	-0.031612862626	2.096710090566
C	2.426422850912	-0.174633781771	1.306917970257
C	2.331138635298	-0.319595405482	-0.047807784005
N	1.096267909265	-0.318063355619	-0.627726271727
C	-1.218389156316	0.082934556546	2.227748583176
C	-1.096408276886	0.336442233214	3.582147322092
C	0.166629181346	0.365807061846	4.215161306448
C	1.323635275439	0.174054757619	3.495713187354
C	-2.570513433731	0.009949120128	1.631058580219
C	-3.080657393683	-1.182266996546	1.029688520713
C	-4.369924146170	-1.148106605099	0.408831009281
C	-5.124699483890	0.041855507843	0.428785966009
C	-4.633680914931	1.186922163123	1.073337346902
C	-3.384297115115	1.163735096849	1.676181066409
C	-4.888773452969	-2.323644233950	-0.180417949641
C	-4.170063476632	-3.524858492266	-0.142903671703
C	-2.934127388696	-3.569371686385	0.493920791196
C	-2.394665315216	-2.414751040426	1.080849731190
H	2.292703669156	0.195478983314	3.985136557938
H	0.213701637902	0.536912962306	5.286267009813
H	-1.990285820850	0.475894044880	4.183240569232
H	3.405293202998	-0.158260258991	1.773527629686
H	-0.949128444090	-0.194013173042	-0.536207266419
H	3.177188994035	-0.426952454493	-0.713978151334
H	-2.999325420174	2.058689052604	2.155841981260
H	-5.228547077659	2.094419593972	1.090816278427
H	-6.102737028006	0.063138127227	-0.044021926897
H	-5.864029887129	-2.289529997258	-0.657983545144
H	-4.586559330037	-4.417814506092	-0.597899412231
H	-2.380284654565	-4.501329115502	0.548344684304

H	-1.445803808507	-2.486995883543	1.603575543429
H	1.041448080897	-0.401341418868	-1.640153666328

11.xyz

35

-786.727987288

C	-0.010673457960	-2.330069196818	4.730642977232
C	1.102368978889	-2.753732622328	4.000740665214
C	1.196430245523	-2.527605476170	2.632872432493
C	0.164603670148	-1.858436806309	1.944078576898
C	-0.955714079723	-1.433292804155	2.678665732166
C	-1.046836146110	-1.674139780705	4.051419611425
O	-0.096676147863	-2.590731156261	6.082063071028
N	0.258346828074	-1.682605754732	0.568956946439
O	-2.148112628865	-1.199587646370	4.724420281445
C	-0.599395488501	-0.736986070378	-0.132391736164
C	0.306259691701	-1.500088575982	6.911839730706
C	-3.070909164337	-2.208963926424	5.142109085685
C	-0.236933537858	-0.677942602675	-1.597335923708
C	0.356885315668	0.465358126469	-2.142205826928
C	0.697500053420	0.515944232624	-3.496502904920
C	0.449946348144	-0.582457316855	-4.320260376197
C	-0.140802645059	-1.730522815155	-3.784608759103
C	-0.481798666857	-1.776102876659	-2.433352968840
H	1.903981891463	-3.267655007527	4.526067713937
H	2.071586322380	-2.873959622153	2.087026627222
H	-1.780150364497	-0.909674527012	2.205217027566
H	1.206727230492	-1.709334013862	0.214090167205
H	-0.537751694547	0.271931875606	0.307728712156
H	-1.640550619260	-1.069107237820	-0.027275531999
H	0.176834694850	-1.831651047049	7.944810355632
H	1.360700250521	-1.249592565284	6.737731837028
H	-0.315893910741	-0.616321017509	6.728595273515
H	-3.875507031882	-1.689296436525	5.666900305704
H	-3.482108080541	-2.734763864622	4.270982515356
H	-2.591339857698	-2.925891235218	5.816462084928
H	0.552514506013	1.324483144848	-1.503164954372
H	1.156397523588	1.413100019130	-3.905228134068
H	0.713564114494	-0.545466546539	-5.374555037165
H	-0.339944693403	-2.588871173376	-4.421982473597
H	-0.944490783778	-2.669971711130	-2.019863706822

11-rc.xyz

35

-786.546796822

C	0.559024219612	-2.217821276314	4.802055807214
C	1.681062075190	-2.436426586834	3.965930308774
C	1.630097242051	-2.191325251197	2.622530408656
C	0.420445694974	-1.701603983300	2.032471242924
C	-0.711740323871	-1.472236513201	2.852059394091
C	-0.662823125522	-1.721108716530	4.210978771386
O	0.766041100581	-2.503443425539	6.077794181919

N	0.393931404720	-1.474316913135	0.711038426149
O	-1.687609846228	-1.533616599447	5.047187300473
C	-0.751007895127	-0.979178970698	-0.055263822753
C	-0.193824459187	-2.379940087975	7.145576096328
C	-2.932647916497	-1.052561787215	4.533392046013
C	-0.409187195058	-0.890906367783	-1.521269148108
C	0.049364211011	0.311829077915	-2.071033423468
C	0.382751115974	0.387858954114	-3.423998967536
C	0.260610891538	-0.739558773061	-4.238098106581
C	-0.197579694897	-1.942520566648	-3.696712038106
C	-0.531931477202	-2.017187083466	-2.344380102771
H	2.586948090194	-2.808519572512	4.433531322894
H	2.501074630678	-2.365373379471	1.997784208991
H	-1.624954762127	-1.097326929105	2.409001850407
H	1.242159365394	-1.657970620487	0.185463371574
H	-1.035055587175	0.006045479782	0.334614012972
H	-1.596962460508	-1.659612056150	0.103671271390
H	0.351902571317	-2.710021244379	8.028827096661
H	-0.509948487355	-1.342222306413	7.256751589141
H	-1.053087225319	-3.025854357927	6.962672888024
H	-3.593918777815	-0.987597691115	5.396479000782
H	-2.805929314942	-0.061841902382	4.085370307710
H	-3.342457253200	-1.756176972845	3.801567088680
H	0.140733935494	1.194021849434	-1.440803307494
H	0.733286708569	1.328086281163	-3.841898104038
H	0.516882127852	-0.679937269597	-5.292804422003
H	-0.300596522984	-2.821319768044	-4.328065706281
H	-0.895994394248	-2.954318704562	-1.927727446918

12.xyz
48
-1153.69621125

C	-2.117717707869	3.558388728924	-0.622281592524
C	-2.426098805552	2.675000453665	0.462628951275
C	-3.595638243299	2.885858168736	1.184929971034
C	-4.472257567893	3.939154427254	0.879333707098
C	-4.186761400364	4.805889032635	-0.150586159085
C	-3.006968696756	4.646049860233	-0.920552415942
C	-2.704688151365	5.542292788898	-1.993567235750
C	-1.570042669650	5.313767815454	-2.747998429094
C	-0.706063156355	4.244500638540	-2.465596045106
C	-0.942443421432	3.365564140344	-1.416017475602
C	-3.593135499047	6.693625258724	-2.325303967891
C	-3.602697574723	7.879221610801	-1.519514780993
C	-4.400472977775	6.630795751275	-3.446386445090
C	-5.249949958302	7.703190865118	-3.805094563561
C	-5.287311769814	8.843501282572	-3.036956250358
C	-4.467384634161	8.962306382984	-1.883923636901
C	-4.478137815164	10.137931739175	-1.085709567368
C	-3.667256096975	10.250276417897	0.021053146114
C	-2.804064005222	9.186653554411	0.375713709343
C	-2.773453005659	8.032063498136	-0.375247103083
C	-0.026833019191	2.253199616628	-1.106713853141

C	-1.503203034888	1.570219059596	0.783107510244
C	0.566545497435	0.307209677444	0.297881034129
C	0.278020606819	-0.554082277628	1.386040599101
C	-0.854205655682	-0.357047438058	2.142958685182
C	-1.734750485246	0.697042612105	1.841368792273
C	-0.328040997512	1.384096223078	-0.011155378204
C	1.130199699223	2.019480410324	-1.843487068387
C	2.003161197293	0.959704945480	-1.539860906976
C	1.730904398128	0.115793750072	-0.487375476351
H	-3.860900833304	2.229014064290	2.006223366848
H	-5.380472431020	4.063848612158	1.463672635419
H	-4.866797091409	5.618506102989	-0.387108558073
H	-1.331403573738	5.983297486456	-3.571006803691
H	0.169155270722	4.128002890811	-3.095526448929
H	-4.389411316287	5.731465807697	-4.057980119231
H	-5.875851091064	7.616021393769	-4.689786865045
H	-5.941837682912	9.670466736880	-3.304647488757
H	-5.140264787273	10.953903090728	-1.368601173741
H	-3.684614733175	11.156020529101	0.622253984357
H	-2.160286686887	9.283033458205	1.246665184790
H	-2.104711731426	7.222705542201	-0.095534940027
H	0.964725020666	-1.367189946383	1.610506917419
H	-1.077576754895	-1.013683386066	2.980060194788
H	-2.612629533607	0.814030361538	2.467393965669
H	1.388002137997	2.661110429246	-2.679283047094
H	2.895506293281	0.815980330396	-2.143895135981
H	2.401363529066	-0.705243874656	-0.243665538732

12-rc.xyz

48

-1153.50075527

C	-2.103000000000	3.568000000000	-0.635000000000
C	-2.387000000000	2.724000000000	0.481000000000
C	-3.548000000000	2.973000000000	1.240000000000
C	-4.416000000000	4.010000000000	0.920000000000
C	-4.141000000000	4.845000000000	-0.154000000000
C	-2.981000000000	4.659000000000	-0.937000000000
C	-2.700000000000	5.523000000000	-2.041000000000
C	-1.604000000000	5.230000000000	-2.855000000000
C	-0.757000000000	4.167000000000	-2.571000000000
C	-0.964000000000	3.328000000000	-1.459000000000
C	-3.546000000000	6.699000000000	-2.362000000000
C	-3.614000000000	7.838000000000	-1.492000000000
C	-4.260000000000	6.698000000000	-3.549000000000
C	-5.100000000000	7.777000000000	-3.899000000000
C	-5.209000000000	8.866000000000	-3.065000000000
C	-4.468000000000	8.930000000000	-1.856000000000
C	-4.545000000000	10.065000000000	-1.005000000000
C	-3.799000000000	10.135000000000	0.149000000000
C	-2.935000000000	9.071000000000	0.497000000000
C	-2.844000000000	7.953000000000	-0.303000000000
C	-0.064000000000	2.238000000000	-1.144000000000
C	-1.484000000000	1.634000000000	0.801000000000

C	0.553000000000	0.338000000000	0.302000000000
C	0.289000000000	-0.485000000000	1.420000000000
C	-0.828000000000	-0.259000000000	2.210000000000
C	-1.700000000000	0.783000000000	1.904000000000
C	-0.338000000000	1.411000000000	-0.015000000000
C	1.085000000000	1.966000000000	-1.916000000000
C	1.948000000000	0.919000000000	-1.602000000000
C	1.690000000000	0.110000000000	-0.505000000000
H	-3.794000000000	2.346000000000	2.088000000000
H	-5.313000000000	4.164000000000	1.513000000000
H	-4.829000000000	5.646000000000	-0.404000000000
H	-1.391000000000	5.868000000000	-3.708000000000
H	0.088000000000	4.007000000000	-3.230000000000
H	-4.196000000000	5.836000000000	-4.210000000000
H	-5.665000000000	7.735000000000	-4.827000000000
H	-5.859000000000	9.698000000000	-3.327000000000
H	-5.201000000000	10.884000000000	-1.288000000000
H	-3.864000000000	11.011000000000	0.790000000000
H	-2.334000000000	9.138000000000	1.400000000000
H	-2.165000000000	7.152000000000	-0.023000000000
H	0.973000000000	-1.298000000000	1.652000000000
H	-1.028000000000	-0.892000000000	3.069000000000
H	-2.560000000000	0.928000000000	2.547000000000
H	1.323000000000	2.578000000000	-2.777000000000
H	2.823000000000	0.742000000000	-2.219000000000
H	2.360000000000	-0.708000000000	-0.255000000000

13.xyz

48

-1152.03476926

C	0.177810626837	-0.166301617745	-0.232346201671
C	0.075750200496	-0.163613567903	1.159098152558
C	1.203664769515	0.090580696412	1.945346280924
C	2.430599206025	0.346590309904	1.310034200378
C	2.534726183557	0.359932053410	-0.082820570968
C	1.390413770867	0.089569466242	-0.863601247536
C	1.143748724132	0.037361477751	3.432401471690
C	2.048681390223	-0.804981395401	4.099151576780
C	2.024337469855	-0.960915312842	5.483125688975
C	1.075806288075	-0.267860946750	6.234736771308
C	0.178812047594	0.581255222764	5.590959411569
C	0.196530236418	0.759202028890	4.197666153432
C	-0.757849106545	1.736805009306	3.605506782321
C	-2.124301777368	1.651697923596	3.922370713129
C	-3.041851912090	2.578171651758	3.421353578122
C	-2.582519675421	3.630265053780	2.600328370912
C	-1.229395809244	3.713485471809	2.289615282342
C	-0.319292561621	2.776817205846	2.780333611302
O	-4.380291667636	2.539563340546	3.654882694077
C	-4.891692692408	1.497849999633	4.474467564945
O	-3.460617901979	4.529208798946	2.047458780820
C	-3.975278856237	5.506825949125	2.954940584891
O	3.677008136242	0.633958105008	-0.766530350283

C	4.854552728319	0.919594869251	-0.024675116421
O	1.440083904455	0.146894757446	-2.234378910463
C	2.093937929664	-0.958213066741	-2.863254528402
H	-0.538077662962	1.149010003161	6.179843872377
H	1.040126494743	-0.376896969052	7.315962605475
H	2.733925618239	-1.627422101056	5.967328401335
H	2.765629606711	-1.371048415546	3.508739732705
H	-2.466374249453	0.831729534607	4.545758425723
H	0.733418109534	2.864694675228	2.527826175841
H	-0.897925298812	4.528518957352	1.651090763176
H	-0.880935334832	-0.371204377447	1.629625753462
H	3.300938271232	0.563903012186	1.920903183696
H	-0.692106262603	-0.366823739494	-0.852719642620
H	5.632921050561	1.105273639346	-0.766190068812
H	4.724707332165	1.812819554963	0.598366663665
H	5.144628060974	0.068987374839	0.604141299270
H	2.051412217821	-0.766514240390	-3.937555406084
H	3.138527725131	-1.033439910859	-2.544217171716
H	1.566756602773	-1.893807197207	-2.637292375035
H	-4.651367318733	6.137125983455	2.373269501879
H	-4.526938696771	5.034305396274	3.774198360684
H	-3.160115414931	6.120148558060	3.359442122230
H	-5.968635703683	1.664237000664	4.526051637865
H	-4.696151849084	0.512898513457	4.033396019566
H	-4.466657245721	1.541680710355	5.484646424897

13-rc.xyz

48

-1151.83070846

C	0.031000000000	-0.093000000000	-0.063000000000
C	0.009000000000	-0.062000000000	1.317000000000
C	1.215000000000	0.055000000000	2.056000000000
C	2.426000000000	0.138000000000	1.349000000000
C	2.461000000000	0.143000000000	-0.042000000000
C	1.236000000000	0.003000000000	-0.777000000000
C	1.205000000000	0.013000000000	3.524000000000
C	2.147000000000	-0.790000000000	4.194000000000
C	2.090000000000	-0.969000000000	5.569000000000
C	1.083000000000	-0.340000000000	6.314000000000
C	0.161000000000	0.479000000000	5.678000000000
C	0.217000000000	0.697000000000	4.288000000000
C	-0.685000000000	1.685000000000	3.681000000000
C	-2.032000000000	1.759000000000	4.071000000000
C	-2.897000000000	2.710000000000	3.536000000000
C	-2.391000000000	3.672000000000	2.598000000000
C	-1.041000000000	3.598000000000	2.224000000000
C	-0.202000000000	2.632000000000	2.742000000000
O	-4.208000000000	2.776000000000	3.819000000000
C	-4.767000000000	1.860000000000	4.760000000000
O	-3.090000000000	4.642000000000	2.004000000000
C	-4.373000000000	5.118000000000	2.443000000000
O	3.581000000000	0.304000000000	-0.765000000000
C	4.832000000000	0.451000000000	-0.093000000000

O	1.107000000000	-0.023000000000	-2.106000000000
C	2.184000000000	-0.242000000000	-3.032000000000
H	-0.582000000000	1.010000000000	6.267000000000
H	1.035000000000	-0.474000000000	7.391000000000
H	2.813000000000	-1.613000000000	6.061000000000
H	2.894000000000	-1.325000000000	3.614000000000
H	-2.420000000000	1.021000000000	4.764000000000
H	0.850000000000	2.640000000000	2.475000000000
H	-0.670000000000	4.346000000000	1.529000000000
H	-0.933000000000	-0.195000000000	1.838000000000
H	3.350000000000	0.257000000000	1.903000000000
H	-0.884000000000	-0.216000000000	-0.634000000000
H	5.574000000000	0.557000000000	-0.884000000000
H	4.830000000000	1.347000000000	0.537000000000
H	5.055000000000	-0.436000000000	0.509000000000
H	1.686000000000	-0.416000000000	-3.987000000000
H	2.826000000000	0.637000000000	-3.096000000000
H	2.768000000000	-1.119000000000	-2.747000000000
H	-4.529000000000	6.035000000000	1.874000000000
H	-5.155000000000	4.393000000000	2.214000000000
H	-4.359000000000	5.337000000000	3.512000000000
H	-5.820000000000	2.130000000000	4.835000000000
H	-4.674000000000	0.830000000000	4.400000000000
H	-4.285000000000	1.966000000000	5.737000000000

14.xyz

57

-1286.17412145

C	6.205516210371	-1.746188078939	0.320975869632
C	7.472653452831	-2.357712857645	0.314542891395
C	8.287053647588	-2.298676404752	-0.808346524640
C	7.842940064195	-1.611609385204	-1.954997990888
C	6.596162377260	-1.009601043318	-1.951379537090
C	5.762681501259	-1.070790719466	-0.822101729109
C	3.912274970017	-1.378358612488	1.295644619248
C	5.361000557705	-1.790901455151	1.574120114756
C	4.383697139463	-0.417995403802	-0.875639606184
N	3.794287603256	-0.193184506252	0.448326102822
C	3.431383792367	-1.215463389114	-1.790835463094
C	4.304306981015	1.010615062537	1.090880294043
O	9.530256592628	-2.852754047868	-0.938373146708
O	8.640498410930	-1.538490381587	-3.061033551750
C	10.071567076572	-3.557183438429	0.170312558419
C	2.093663488529	-0.503362288495	-2.036837262201
C	1.175063423426	-1.292500065315	-2.937811441398
C	0.277359701364	-2.224677144414	-2.405134467018
C	1.216885771761	-1.128167866173	-4.327109640564
C	0.399039103381	-1.885623004563	-5.165316386716
C	-0.478994036512	-2.841931140496	-4.628895640809
C	-0.543133928111	-2.990932911274	-3.233714697083
O	0.512196014546	-1.732302119818	-6.526175254340
O	-1.283775829706	-3.594701729372	-5.446994685132
O	-1.361043272389	-3.937349459269	-2.666797460779

C	-2.759128402640	-3.640995467937	-2.723892481659
C	-0.650821099621	-4.771974114246	-5.958946939571
C	-0.587480385614	-1.060317724577	-7.146851490002
H	7.808332410908	-2.880151841708	1.206621964336
H	6.275526901009	-0.479856952986	-2.846601291733
H	3.383083397866	-1.186045976699	2.236729438189
H	3.385421687687	-2.203549679899	0.801471470256
H	5.379426026629	-2.798034864073	2.011308341278
H	5.800088506737	-1.126068924411	2.332801596704
H	4.499614696636	0.574939748989	-1.333500426442
H	3.249925853105	-2.211694042672	-1.366533779621
H	3.936111437698	-1.383793128560	-2.750565376948
H	4.054344374715	1.883917916377	0.477887632650
H	3.818059912416	1.132970056690	2.065576992951
H	5.397562980373	1.013600937983	1.251376151315
H	9.464000066752	-2.017698872730	-2.863890509943
H	11.053148402760	-3.909911695183	-0.148813213717
H	10.182366609437	-2.895909463663	1.037943444561
H	9.441205173872	-4.414314672539	0.435318588599
H	2.290883625942	0.480518516852	-2.484125618902
H	1.608847452447	-0.321859470617	-1.071421824334
H	0.204818276199	-2.372847995265	-1.329908665342
H	1.890770329512	-0.405098710034	-4.781515755331
H	-3.267040334230	-4.475286353294	-2.235365648089
H	-2.974947485239	-2.712453219060	-2.180321248028
H	-3.101097227881	-3.555085621379	-3.759998633842
H	-1.392427350810	-5.270012629232	-6.587211698342
H	0.226487796231	-4.510105073144	-6.561027243830
H	-0.354593025201	-5.438216440715	-5.140130599453
H	-0.362283445915	-1.029342106205	-8.214986584548
H	-1.524115812458	-1.602685112898	-6.982219350837
H	-0.676150568997	-0.036639792808	-6.761613704913

14-rc.xyz

57

-1285.97184395

C	6.199917435277	-1.770842086152	0.264547941027
C	7.406907161334	-2.489847818701	0.289357422868
C	8.290787564186	-2.422520197534	-0.777579663191
C	7.977464342089	-1.620847950041	-1.905709501691
C	6.777890366697	-0.928394235851	-1.945566160030
C	5.875571575405	-1.026077212220	-0.881141352314
C	3.932785958471	-1.170881468861	1.226165446785
C	5.336362656068	-1.758276670377	1.496935365411
C	4.518806301562	-0.335041018863	-1.016633666970
N	4.067698130991	-0.046172668122	0.335096153810
C	3.510335835362	-1.165893148718	-1.833516162814
C	3.993354526629	1.292966391417	0.861883364616
O	9.481325720785	-3.055071437970	-0.874970269705
O	8.833848897813	-1.552597612771	-2.946950305938
C	9.893506709627	-3.912711531093	0.188595168593
C	2.135678069980	-0.496400258187	-1.971029556051
C	1.215599339323	-1.286063914465	-2.871894903576

C	0.414868230490	-2.309789452598	-2.355202771320
C	1.168160206349	-1.023069193455	-4.244695784401
C	0.356621630447	-1.778981738358	-5.090886354195
C	-0.421079317249	-2.830330653859	-4.575834737009
C	-0.399118481074	-3.074261800804	-3.192012365604
O	0.389024437116	-1.524977504429	-6.438104981366
O	-1.212373451460	-3.586142082979	-5.400967268343
O	-1.116096145507	-4.109807392584	-2.650138757798
C	-2.536063049961	-3.926957633721	-2.635025326273
C	-0.533766748105	-4.692311190361	-6.007587617706
C	-0.807618559240	-0.955771908444	-6.980803195395
H	7.654852292439	-3.076237376010	1.168677355547
H	6.540175387794	-0.345755635588	-2.831497048347
H	3.457542941602	-0.846784236975	2.153419999581
H	3.297572677544	-1.924150098456	0.749357970898
H	5.228769876126	-2.762193053549	1.921711429135
H	5.809489232316	-1.135533289958	2.267606731796
H	4.658299870637	0.629480920547	-1.514715239462
H	3.412229154310	-2.170948673833	-1.406388429016
H	3.958598433107	-1.299864076867	-2.824861795831
H	4.256929688057	2.020152902056	0.095005263102
H	2.970826936374	1.482767878814	1.215044611954
H	4.666566248595	1.393272643268	1.723490421808
H	9.595679034315	-2.130485763823	-2.759616282369
H	10.854922711071	-4.322990774587	-0.119286981963
H	10.012757544238	-3.344195809725	1.117049122267
H	9.170905970610	-4.723484063565	0.330517266217
H	2.267181160565	0.516352874922	-2.373147344216
H	1.674636844558	-0.387427390389	-0.980773747396
H	0.411742438722	-2.533878387768	-1.290780264592
H	1.763961419679	-0.224718827204	-4.681144547735
H	-2.952734385578	-4.830690307755	-2.185889182395
H	-2.800800798099	-3.056984485293	-2.021122928805
H	-2.927483600196	-3.802560121184	-3.649030071189
H	-1.268720962498	-5.189307164905	-6.643901904459
H	0.307082693438	-4.344174901449	-6.617751131655
H	-0.175800061471	-5.390835869077	-5.242480251068
H	-0.619688097785	-0.817123375931	-8.047297595490
H	-1.661477752421	-1.624941550710	-6.838618157166
H	-1.010934647384	0.016466244784	-6.515005930350

15.xyz

44

-1165.82101694

C	-0.765840964678	-2.204076728886	5.018418839427
C	-2.145821608956	-2.123065559721	5.197610064212
C	-2.930780125795	-3.289090015669	5.107987651265
C	-2.315904188687	-4.501009177972	4.795283569573
C	-0.937895587592	-4.573206499137	4.603780940790
C	-0.142230711030	-3.424682551292	4.722567486772
C	1.321752366590	-3.525887663642	4.595970781895
N	3.418215756229	-4.313861972325	5.016690852778
C	2.142077437375	-4.487755484558	5.252340574424

C	2.238540455603	-2.750357256807	3.918901043677
O	3.484679047803	-3.217997444120	4.171474559238
C	2.166848631589	-1.613691290131	3.001653753434
C	1.049686254539	-1.427728210972	2.174026033728
C	3.237908926042	-0.710859330341	2.912551522112
C	3.193480502731	0.337192996190	1.999359863287
C	2.092483911999	0.509849244205	1.159097768212
C	0.997988709550	-0.371428121711	1.268043136716
O	-2.693304240877	-0.911568540954	5.535180263992
O	-4.291596281525	-3.253194112749	5.278596488045
O	-0.099402552110	-0.263000595373	0.453834676839
O	2.086828470508	1.562287541677	0.283622487282
C	-3.554535402124	-0.331723605284	4.548928329295
C	-4.721758787438	-3.075820224043	6.633365922634
C	2.191333407795	1.204393106086	-1.100795896527
C	-0.917189979891	0.891589667816	0.679244215400
H	-0.181000415472	-1.294650217689	5.127781373262
H	-2.935448013190	-5.390308339409	4.713667420516
H	-0.479240972532	-5.528232034481	4.360221652966
H	1.828672415590	-5.288858977422	5.911807361189
H	0.206160331688	-2.110365200487	2.211746644609
H	4.106512882557	-0.832433181259	3.552244833449
H	4.022277480453	1.034923178318	1.915154459426
H	-3.905338106265	0.611518965935	4.971949659959
H	-2.996177312802	-0.134774681352	3.626137425481
H	-4.406968882768	-0.983504725800	4.334981116356
H	-5.812994502993	-3.060391953079	6.605797274743
H	-4.381646216490	-3.914333761301	7.253217910144
H	-4.347666421688	-2.133183398319	7.045988179035
H	2.165131524555	2.142990324939	-1.657202787206
H	3.142361175548	0.691770655401	-1.287983972401
H	1.357364980519	0.565935411914	-1.408348790618
H	-1.747129299058	0.814661463797	-0.025485493219
H	-1.304024572346	0.887486437198	1.705017357258
H	-0.358784123630	1.814984113242	0.497755001268

15-rc.xyz

44

-1165.61339718

C	-0.771573987222	-2.371959025379	4.872296860120
C	-2.131565192072	-2.317833172364	5.140976210239
C	-2.872732109890	-3.552332217666	5.285069107314
C	-2.172294818671	-4.782010851289	5.212589770793
C	-0.823515963086	-4.811556546440	4.985770156109
C	-0.091886336706	-3.600066434932	4.782911517993
C	1.328828501862	-3.669218191706	4.550660370636
N	3.467827916140	-4.385160210167	4.845872519816
C	2.217733730589	-4.655127143881	5.088333640129
C	2.198570994407	-2.789665377022	3.897477739692
O	3.457162112443	-3.216429400969	4.081975451465
C	2.042141346287	-1.634647142883	3.033796948234
C	0.947939152370	-1.533442407985	2.156830586479
C	3.035959513530	-0.637194410107	3.007685530600

C	2.935738796201	0.416503469160	2.115248461010
C	1.871938989552	0.495452281690	1.204595621377
C	0.847577604016	-0.479546805767	1.256260073210
O	-2.826594917082	-1.194105207854	5.310953798591
O	-4.164878279998	-3.672049795340	5.501380366077
O	-0.213237839050	-0.451575719832	0.392590811655
O	1.878997132875	1.540689990552	0.346343534185
C	-2.148385046963	0.067716966416	5.283339151159
C	-5.130294826589	-2.599879988631	5.558101932384
C	1.636678885160	1.300519704092	-1.050648494033
C	-1.194294860778	0.556125216616	0.669417758357
H	-0.211121194447	-1.449797614274	4.781385256107
H	-2.747543819549	-5.693919379758	5.334642299293
H	-0.314207478457	-5.766876341298	4.916245571524
H	1.976130357793	-5.522473174650	5.690826659476
H	0.173901776595	-2.294741431958	2.134303380748
H	3.883920101209	-0.695769148590	3.682630628660
H	3.701048989526	1.186004702804	2.075578823036
H	-2.924505883704	0.813119313293	5.450028082346
H	-1.403652822495	0.113353485743	6.083829773369
H	-1.676774045214	0.228307484569	4.309940284538
H	-6.084448560276	-3.112640791339	5.669320074430
H	-4.931893050512	-1.961066846251	6.418899701582
H	-5.107529993589	-2.021671875940	4.633754334238
H	2.047345035222	2.170438853856	-1.565004562366
H	2.159935955804	0.396291276464	-1.375578284273
H	0.570575123177	1.209648883428	-1.267170229368
H	-1.921966203711	0.500090934028	-0.141697597406
H	-1.691797906649	0.351690295284	1.624027572125
H	-0.745631478716	1.555346046711	0.696390363062

16.xyz

100

-1784.87122109

C	2.787537734240	-7.553705191105	-0.459692104781
C	1.688131337512	-8.318439021095	-0.054718814878
C	0.938419409609	-7.958616837848	1.064488075486
C	1.237771970283	-6.825130098628	1.830880895112
C	2.331996209180	-6.024641828184	1.424191778401
C	3.076697976595	-6.412995331043	0.301998501595
C	0.421807621894	-6.555086311610	3.044258353417
C	2.708982896784	-4.752230002435	2.098086480032
C	3.648876668447	-7.901657822991	-1.680108477228
C	1.009961422726	-6.366748906884	4.304697574419
C	0.228902917450	-6.176084952210	5.440031162028
C	-1.175557024991	-6.157956533739	5.381085989490
C	-1.755909310808	-6.345237738957	4.120569544056
C	-0.976283151007	-6.543385480798	2.978360476376
C	4.038262486029	-4.508128640148	2.471476450213
C	1.769073307666	-3.733633515028	2.320650753263
C	2.145651384869	-2.519005869585	2.884958984419
C	3.475944403481	-2.274867975537	3.260662429987
C	4.413152030169	-3.295828860867	3.048927758054

C	3.875355014918	-1.000401786886	3.917502864867
C	3.563831514741	0.274046026584	3.385950564942
C	4.551794933710	-1.078165095785	5.142569300495
C	4.925946307236	0.047247489318	5.890008876127
C	4.587374710004	1.296753457034	5.359125287387
C	3.925269875733	1.399952098767	4.136408040857
C	2.918972126166	0.481134380145	2.062187446193
C	1.728046384945	0.982597897509	-0.487298100191
C	2.841119347114	0.135557906381	-0.345211469561
C	3.422493508257	-0.112185660494	0.893927199733
C	1.815253950173	1.331381032359	1.927626324254
C	1.231576221964	1.572686382397	0.681440952641
C	1.117440346525	1.225860932111	-1.873235303945
C	5.655113793166	-0.126728038046	7.228393858344
C	-1.998282353827	-5.950643224870	6.658746120280
C	3.569999623412	-6.754686170634	-2.708031645584
C	5.114474948149	-8.090156682105	-1.239412444391
C	3.185574112165	-9.193973522985	-2.367638632005
C	-0.089116676961	2.173652197532	-1.821603694690
C	0.650277421804	-0.116364675159	-2.471642278040
C	2.180887763019	1.851488922043	-2.798511191662
C	5.991719451182	1.219465033840	7.885353728717
C	6.972979822683	-0.894119863975	7.000960037099
C	4.761882671161	-0.925712887390	8.199104537227
C	-3.508997759852	-5.945034015219	6.385692399616
C	-1.620981981554	-4.601040520109	7.302556437671
C	-1.693392246318	-7.090235947097	7.652244457002
H	1.405146243515	-9.214455989890	-0.598490321632
H	0.111415155801	-8.595129393869	1.372251297760
H	3.898708916737	-5.766600651263	0.002702410690
H	2.093064843460	-6.382239833807	4.399511416155
H	0.733881841863	-6.043799852877	6.394186704152
H	-2.835747560364	-6.337468280732	4.007608020837
H	-1.468463399672	-6.676521554349	2.016896280700
H	4.788560077987	-5.281047071029	2.318341690294
H	0.731078301258	-3.891423556736	2.039285420225
H	1.394801451647	-1.748622671523	3.041332937789
H	5.453051972419	-3.135190702296	3.325645693413
H	4.761612832236	-2.071149454823	5.533825856339
H	4.845281027329	2.210664997000	5.884967650856
H	3.708658836556	2.387804009556	3.735122488536
H	3.274030839795	-0.343508034720	-1.220510077353
H	4.287841624635	-0.767748557485	0.954570387901
H	1.391083023442	1.802896900722	2.812030383637
H	0.371314194256	2.233754912723	0.639090269089
H	4.186193160685	-6.989900483328	-3.585047879122
H	3.929957930902	-5.807370550705	-2.291763077232
H	2.538424755484	-6.603036353509	-3.047817362422
H	5.741159142018	-8.334871309784	-2.106243878898
H	5.203962952293	-8.907046907933	-0.513244681640
H	5.522726304948	-7.183642040914	-0.779203436911
H	3.833459888383	-9.403558659929	-3.226877712737
H	3.240167767644	-10.057863065170	-1.694309891240
H	2.158056759845	-9.113516203025	-2.742183421244

H	-0.484997428447	2.316574973632	-2.834005291971
H	-0.900827622143	1.770601113518	-1.204095649120
H	0.182520065385	3.161763365196	-1.431157411253
H	0.211982034070	0.044448621415	-3.464758155441
H	-0.109852674141	-0.585823787095	-1.835783369746
H	1.479276076980	-0.823837624631	-2.582958754645
H	1.760493377760	2.021682836383	-3.797801475919
H	3.055635305527	1.201658177753	-2.911020353009
H	2.524995580296	2.815746653057	-2.405499979974
H	6.517029378099	1.042942202201	8.831308219299
H	6.646524987643	1.831252485665	7.253247751791
H	5.090432687757	1.801281003762	8.112579418285
H	7.503438948076	-1.025154763093	7.952588541936
H	7.632598424446	-0.347076442713	6.316658581048
H	6.798280360028	-1.889291820881	6.577520883575
H	5.271161080459	-1.053893215804	9.162619960896
H	4.526531899664	-1.922869061639	7.810909846700
H	3.815486399359	-0.402591288385	8.381362022707
H	-4.050129339072	-5.794621979304	7.327258642271
H	-3.799109882006	-5.135737810954	5.704891613590
H	-3.851457163339	-6.894621215474	5.957269151405
H	-2.204091037066	-4.443048392692	8.218684427546
H	-1.828501994501	-3.768642595204	6.619517185967
H	-0.559889936661	-4.558654998834	7.571970843058
H	-2.270646533247	-6.952655622877	8.575321373428
H	-0.632358374193	-7.121712601302	7.923513581263
H	-1.961478305918	-8.064287182994	7.225547812666

16-rc.xyz

100

SCF = -1784.65658213

C	3.031291725605	-7.665074237676	-0.359753247534
C	1.795349285799	-8.294023165865	-0.110350791225
C	0.940058052315	-7.845882161453	0.888635285790
C	1.260183160288	-6.747074148013	1.696763298473
C	2.483491614250	-6.058424516440	1.423574480146
C	3.345022590748	-6.559266570548	0.429431506537
C	0.383470024153	-6.417042240493	2.832549613723
C	2.846381288489	-4.797651753069	2.075842161982
C	3.995275165362	-8.129995157194	-1.455977113387
C	0.904287441518	-6.152684236063	4.116311299780
C	0.062192451915	-5.894891178866	5.188691147854
C	-1.337217054055	-5.875328743758	5.044170800869
C	-1.849653347285	-6.143515839668	3.763135201805
C	-1.014366694678	-6.421203540391	2.686125049593
C	4.191988227540	-4.539787391314	2.440186717789
C	1.892240290256	-3.775419447072	2.301922284788
C	2.263507772403	-2.562701083710	2.845484348885
C	3.601476576904	-2.317330297247	3.240627033053
C	4.551374397124	-3.350077469436	3.038696362578
C	3.988953668017	-1.070854010707	3.906359558049
C	3.473695803490	0.205490246538	3.519075382361
C	4.834343268534	-1.156976520285	5.028278657813

C	5.139920285369	-0.060778927595	5.833849656545
C	4.573834086467	1.176078064779	5.466300232382
C	3.767816832434	1.300934942793	4.341257553538
C	2.741706266631	0.428777402242	2.261320959470
C	1.364850092914	0.939026824464	-0.185353528413
C	2.507453337595	0.119434746708	-0.141746815711
C	3.178476857114	-0.136629054116	1.045377550676
C	1.615276567417	1.268094880440	2.221215359355
C	0.938282245772	1.503708028418	1.028898630565
C	0.649282037257	1.187450462167	-1.515251267110
C	6.030662438198	-0.235538242636	7.068852748565
C	-2.231170003652	-5.583518703300	6.251809712019
C	4.156119314001	-7.005465169832	-2.500173385505
C	5.367949629807	-8.446354238358	-0.828358847485
C	3.491621963168	-9.389595353091	-2.174951157559
C	-0.572894446173	2.103385599776	-1.360795462407
C	0.174784711210	-0.161057985493	-2.095674224442
C	1.630567924751	1.851528151224	-2.504095110636
C	6.262077571875	1.088944133226	7.809450196104
C	7.400980799458	-0.793682389507	6.633910291044
C	5.359417156795	-1.225261751849	8.043677119262
C	-3.722761502696	-5.589131199388	5.890622009404
C	-1.877049779418	-4.195195483187	6.824749633542
C	-1.990152821501	-6.659019725089	7.332134148320
H	1.500188697301	-9.165816888431	-0.684703903847
H	0.025841047451	-8.398731931541	1.087200119372
H	4.261318104509	-6.010093745809	0.232027216616
H	1.978075370398	-6.187451397178	4.279241935429
H	0.510140345168	-5.714911527716	6.162400334449
H	-2.920906299338	-6.134980477314	3.591071021368
H	-1.452473746310	-6.609031554561	1.708861454692
H	4.945708516031	-5.306528954042	2.285359293333
H	0.858427205244	-3.934490026137	2.013903211447
H	1.510457263771	-1.797593149648	3.002490350609
H	5.587367017379	-3.189176636044	3.322856252737
H	5.201617438276	-2.139595501662	5.309868863167
H	4.781742802431	2.066878667901	6.049657821869
H	3.403213329242	2.285158198374	4.059287981533
H	2.892088171694	-0.324098138715	-1.056294126233
H	4.073804933891	-0.752183760956	1.029125609470
H	1.242124161732	1.712188645425	3.140960725905
H	0.058945792923	2.138632849083	1.055968889929
H	4.842253820799	-7.328361490375	-3.292302217230
H	4.564669539136	-6.090558397981	-2.057096083063
H	3.193786288123	-6.757813937020	-2.963234016817
H	6.065119969768	-8.779373068793	-1.606518716224
H	5.285135112664	-9.244622763087	-0.081506699630
H	5.809530800378	-7.570377350649	-0.340931093891
H	4.221531077893	-9.688387610560	-2.935552125934
H	3.369734490870	-10.234275438486	-1.486483393571
H	2.537003745860	-9.218766706894	-2.686437804014
H	-1.045554134791	2.246216813112	-2.339260802985
H	-1.326886918815	1.672584771852	-0.691271111047
H	-0.298070954233	3.094450613960	-0.980741715486

H	-0.339571888492	0.004156909035	-3.050219928978
H	-0.525196811284	-0.657829260573	-1.413459100100
H	1.010688778638	-0.844369589809	-2.280728357583
H	1.128643099350	2.032245510836	-3.462334485964
H	2.505158547906	1.221636503334	-2.699646476768
H	1.985426765666	2.814705618698	-2.119014216568
H	6.907279173378	0.910373669375	8.676931881592
H	6.761365404257	1.832021273055	7.176359941614
H	5.325603044511	1.521486734703	8.180687076074
H	8.045443904428	-0.922179121839	7.511808312305
H	7.903225177802	-0.108258382560	5.941217340137
H	7.311212090919	-1.768283686634	6.141885516442
H	5.985472740623	-1.352543104648	8.934946874889
H	5.217973554331	-2.213549459323	7.592481322004
H	4.379044785561	-0.855829022201	8.366550332337
H	-4.314697797067	-5.370337806660	6.786494024345
H	-3.965813216486	-4.826506981002	5.141190319427
H	-4.049541808395	-6.564363771775	5.510929353542
H	-2.506897857155	-3.979678122839	7.696458106683
H	-2.046513072846	-3.408036036267	6.080497222487
H	-0.831755552203	-4.137576475388	7.146980129138
H	-2.624259431910	-6.459729190822	8.204583425251
H	-0.949140779065	-6.673160150913	7.672565671204
H	-2.236970795640	-7.657872296375	6.953469470429

17.xyz

40

-923.066663518

C	-1.062040930956	-1.899054674323	-3.247814771368
C	-1.941690569438	-1.012492399063	-3.860909919791
C	-1.877119231647	0.346669495497	-3.550635329901
C	-0.940320482425	0.795372287405	-2.624921287435
C	-0.046983888967	-0.085847652647	-1.992170833833
C	-0.106078290773	-1.463552833710	-2.314100385279
C	0.762780535586	-2.490925461452	-1.672062710804
C	0.953345685294	0.491168418729	-1.049271228833
C	2.257752691004	-4.416772567011	-1.806812223791
C	1.500667787265	-3.422561486793	-2.431054319623
C	0.821319056409	-2.606670646024	-0.274057415059
C	1.572350987167	-3.598246631676	0.352804748499
C	2.295447771472	-4.510382910995	-0.417193449704
C	2.326719499649	0.268639855639	-1.237129655903
C	0.572116237754	1.330050010518	0.018098571350
C	1.531071695054	1.910840610749	0.851377072828
C	2.887981976015	1.674832137341	0.640677193696
C	3.286681380414	0.847940789526	-0.410552681563
O	1.462033458922	-3.427323870174	-3.808934044584
O	-0.747722654131	1.654708352210	0.247851216957
C	2.265232820688	-2.435969413291	-4.446686984204
C	-1.515952593642	0.653013882758	0.912088551521
H	-1.115164127156	-2.957870297562	-3.483621868508
H	-2.673865887412	-1.381712237944	-4.574985089030
H	-2.550669050780	1.054613037756	-4.027523373689

H	-0.884038172443	1.853657681272	-2.387285948734
H	2.808554589192	-5.115046909133	-2.431808337414
H	0.250998127525	-1.908186770012	0.331737278109
H	1.585779391875	-3.661276201169	1.437949855364
H	2.882996628837	-5.291569291203	0.058798471848
H	2.645254245836	-0.363061114124	-2.061583733473
H	1.191627267561	2.552447870222	1.660521555015
H	3.626904440191	2.136136833164	1.291234627313
H	4.341643487013	0.659311478587	-0.592738378058
H	2.162504735883	-2.602266386352	-5.521097062869
H	1.917899107598	-1.424663546845	-4.204070821203
H	3.318326586647	-2.545288893189	-4.157608561190
H	-2.510351691072	1.078338342403	1.063304298891
H	-1.601225668279	-0.253964048980	0.301843863098
H	-1.071406945558	0.404847976003	1.884356590049

17-rc.xyz

40

SCF = -922.85989943

C	-0.802688086190	-1.750910923836	-3.634471151462
C	-1.824582352373	-0.927873772145	-4.073001833898
C	-2.080513993864	0.293183927519	-3.422393207091
C	-1.310645019804	0.680776804325	-2.340300266763
C	-0.245050917190	-0.123982458352	-1.888637265280
C	-0.018565450717	-1.394971251254	-2.518225417032
C	0.903855514116	-2.368402631774	-1.932634575375
C	0.692065267842	0.402033866275	-0.895440192727
C	2.611002392697	-4.126189452199	-2.148265671865
C	1.794900401215	-3.154997869808	-2.739695543026
C	0.887911517672	-2.610566713217	-0.539601305493
C	1.685843752090	-3.581800098841	0.035015291598
C	2.551441244133	-4.335357343715	-0.775586376820
C	2.085197368709	0.328014409974	-1.123667730227
C	0.241408736876	1.069328486812	0.293975954048
C	1.166557489429	1.631085843115	1.181579689262
C	2.526118286086	1.544276351811	0.907713534361
C	2.992131076941	0.895081925272	-0.248123398484
O	1.823600052898	-2.853294096128	-4.042960566865
O	-1.079561116927	1.057429162198	0.506128096828
C	2.707754276770	-3.563054114696	-4.914066617220
C	-1.610442450239	1.659531625947	1.689577825788
H	-0.639954314715	-2.709370684405	-4.114070022440
H	-2.444264229727	-1.236503950564	-4.910275699872
H	-2.871762184553	0.944463574199	-3.782993412298
H	-1.485429393496	1.642620174405	-1.871374011853
H	3.292062651617	-4.714931507008	-2.751688782052
H	0.190052337748	-2.051453569285	0.075597532282
H	1.631631932359	-3.772892770958	1.102294466033
H	3.183230082097	-5.098475704591	-0.329347277065
H	2.436633679995	-0.140369139573	-2.037625282295
H	0.831028869337	2.130395315693	2.083127217499
H	3.234493405456	1.991090270654	1.599963573977
H	4.055880079528	0.855707038964	-0.461373618541

```
 H        2.540684228821     -3.136881097496     -5.902705394240
 H        3.748937350071     -3.409018817504     -4.613679650863
 H        2.467447907465     -4.630880904555     -4.922694328661
 H       -2.688765312713      1.518166270115      1.625717641673
 H       -1.221308941585      1.158907777671      2.581920811817
 H       -1.374976131703      2.727968863060      1.718300443338
```

18.xyz

```
48
-1376.32569933
 C       -5.294524729374      2.036684989149      5.890443709022
 C       -5.359882275634      1.425808042654      7.173277860395
 C       -4.813736772237      0.182785039155      7.388467933240
 C       -4.177653559228     -0.498276305153      6.320706049032
 C       -4.098104193263      0.066372348594      5.067312703269
 C       -4.651823334891      1.350654152186      4.807471668526
 C       -4.588176301445      1.959219355356      3.521960176899
 C       -5.144833283663      3.217140674578      3.326211844788
 C       -5.788537101277      3.905647003169      4.408280791004
 C       -5.849571209275      3.309534092570      5.656233974039
 C       -3.923661154406      1.271807625397      2.378853324511
 C       -4.652227075256      0.393884657674      1.526974250042
 C       -6.033989392478      0.118585764223      1.722968134685
 C       -6.710731068540     -0.739232142057      0.885028037661
 C       -6.050983006139     -1.373558574018     -0.197062492596
 C       -4.715955163727     -1.130282303835     -0.416556716274
 C       -3.988272601020     -0.249392631251      0.430569015542
 C       -2.620780759457      0.009584259581      0.215810318373
 C       -1.908891128706      0.863035309720      1.041259358959
 C       -2.574433567717      1.502017059268      2.140412727958
 C       -6.373795688211      5.238037975208      4.172118171064
 O       -5.067169926557      3.768083948617      2.098366659157
 O       -1.911514156023      2.335626922704      2.966739637506
 C       -0.477262944135      1.128341573718      0.809597631695
 O       -6.343761247005      5.814234327166      3.081642148925
 O       -6.946548695713      5.779934993010      5.244544320589
 C       -7.534051095904      7.080872259703      5.074168575906
 O        0.032645715374      0.492301813431     -0.242700255264
 O        0.201958155245      1.877614031946      1.516207290384
 C        1.424575926685      0.719789218484     -0.520994855180
 H       -5.851336010265      1.962835335388      7.981989957281
 H       -4.866443976588     -0.278998839625      8.370898310002
 H       -3.746115955930     -1.481246170871      6.494702319934
 H       -3.605884400491     -0.469105723899      4.260135301662
 H       -6.337340800018      3.834376850458      6.472414119219
 H       -6.555094319269      0.597007791621      2.547608031335
 H       -7.767159661628     -0.933299776930      1.055330004027
 H       -6.601884186504     -2.047468882194     -0.847854037243
 H       -4.193582534795     -1.607911485510     -1.242908664478
 H       -2.113369908869     -0.472640867854     -0.614615318022
 H       -5.505885086269      4.653693067955      2.145372951149
 H       -0.977120742022      2.386438138162      2.645372771421
 H       -8.320121455014      7.044523660241      4.316464062499
```

H	-7.950845794778	7.334591762821	6.048296801444
H	-6.769626525127	7.804868040405	4.782454496151
H	1.600144269082	1.778472061574	-0.725777322629
H	1.640610763956	0.117380859605	-1.402715966468
H	2.037012510453	0.398289889813	0.324564960482

18-rc.xyz

48

C	-4.940528331632	2.187032867983	5.975664787951
C	-4.728734781731	1.766984928654	7.306034653340
C	-4.028260482296	0.601499854711	7.573518480267
C	-3.543885530188	-0.181368543085	6.510967448868
C	-3.745115322683	0.206522342679	5.196794426936
C	-4.417096705701	1.414860078141	4.892484376957
C	-4.639836219107	1.839559482590	3.547033051173
C	-5.426571878611	2.998724600782	3.306831281523
C	-5.956342454646	3.755292322001	4.400774897967
C	-5.698476543225	3.349546035719	5.694484142238
C	-4.002611086059	1.173053967410	2.409779007471
C	-4.764008780421	0.569438313625	1.362910115259
C	-6.165162948739	0.392632100937	1.460279254353
C	-6.878741311792	-0.197659064786	0.430574140376
C	-6.223502112482	-0.662641315789	-0.723093929035
C	-4.846611087438	-0.544360293525	-0.827645718533
C	-4.098346197867	0.060530023521	0.204544021019
C	-2.686514312296	0.137541906904	0.127463182614
C	-1.930644878950	0.682032342437	1.145690869003
C	-2.585691479208	1.208979766139	2.304413364504
C	-6.742908096191	4.973266162414	4.109314408177
O	-5.589883331037	3.401525420211	2.057550373613
O	-1.897111510018	1.805979693795	3.263190667553
C	-0.456187691887	0.760816273019	1.063420304789
O	-6.957966132176	5.368938962353	2.961100807991
O	-7.193478764816	5.595549230858	5.184699985821
C	-7.963744011994	6.796065035190	4.967443652120
O	0.051628994549	0.236344513693	-0.038248360748
O	0.234649886502	1.271309555892	1.948507691409
C	1.486692923839	0.283250862288	-0.180056541097
H	-5.132055392013	2.365809017018	8.118547494455
H	-3.869855536296	0.283732389173	8.599783177710
H	-3.016948880984	-1.107943536578	6.720662816188
H	-3.396831783238	-0.431736830264	4.390273046398
H	-6.095097176050	3.932177361179	6.520612049777
H	-6.683757680226	0.707537492143	2.360740928487
H	-7.954800830522	-0.314251165911	0.523305844459
H	-6.792758119029	-1.129819619464	-1.521192326263
H	-4.326244322047	-0.926857294965	-1.701926637804
H	-2.184609134581	-0.251727534150	-0.753263589255
H	-6.142093156641	4.236181326306	2.079945524740
H	-0.928791441964	1.755739398868	3.014285347230
H	-8.853550956319	6.566597309876	4.377947508836
H	-8.237141560952	7.139749112234	5.963575654781
H	-7.353450515079	7.541474731184	4.453718131309
H	1.827094464780	1.320538879507	-0.179745957742

H	1.690916959848	-0.191034621584	-1.138429057648
H	1.959229791563	-0.268752649232	0.634779972088

19.xyz

36

-920.716931798

C	-2.425078383172	-0.680207952635	1.526616666747
C	-3.527436937497	-0.824496917772	2.407503499273
C	-4.515554193069	0.133868386018	2.468486505594
C	-4.432812355651	1.280321401874	1.645134038323
C	-3.374469191305	1.448769594177	0.777422493828
C	-2.341733622911	0.477461073555	0.688117097072
C	-1.230619067915	0.625066430930	-0.205234207356
C	-0.250882738736	-0.360487253080	-0.228047219047
C	-0.331334344639	-1.505203073855	0.602601977774
C	-1.394657953372	-1.656221403053	1.455200164242
C	-1.098537821191	1.806885685270	-1.107697101270
C	-0.374258041304	2.979488335915	-0.714576988777
C	0.247354423704	3.093241389245	0.557746156573
C	0.940784485476	4.232351603701	0.908567052753
C	1.049965839828	5.316927173251	0.008091083335
C	0.454696005231	5.238422139563	-1.232123928287
C	-0.266899175760	4.081264296048	-1.622671447835
C	-0.891203381556	3.985611390433	-2.895674266848
C	-1.588733162582	2.863136177983	-3.262270517164
C	-1.691508266219	1.772375927411	-2.364306267808
O	0.837592145025	-0.287625625280	-1.038971879443
O	-2.402278461331	0.700750686517	-2.805029892944
H	-3.578807967328	-1.709951087872	3.038090175765
H	-5.355982533590	0.013065727169	3.147206568827
H	-5.211574695580	2.037519804085	1.697212029950
H	-3.319447952153	2.335540371077	0.151326193427
H	0.460465573995	-2.246829027511	0.546054361875
H	-1.457503934746	-2.535526293912	2.092612960426
H	0.168143003152	2.267017887203	1.259494350583
H	1.407595621314	4.297701674036	1.888531937698
H	1.600473417795	6.208573508310	0.297022632700
H	0.529992205000	6.067523591631	-1.932973529887
H	-0.810575781047	4.822517734635	-3.585939893334
H	-2.069384650752	2.785694555114	-4.233405723264
H	0.794196456812	0.540515285845	-1.550540043505
H	-2.414142953791	0.022396948693	-2.105615927212

19-rc.xyz

36

-920.499616233

C	-2.497693406145	-0.773469714179	1.440673278837
C	-3.667920898892	-1.105746537784	2.148167742264
C	-4.770998242523	-0.260704855248	2.134503223663
C	-4.714621508993	0.951693445106	1.430956853822
C	-3.569457603103	1.307936805531	0.734840873090
C	-2.447388297874	0.442342223666	0.691178103903

C	-1.250017287622	0.792899416615	-0.018780892548
C	-0.116514402241	-0.057799439571	0.101090565078
C	-0.176848429786	-1.248914471314	0.856296321100
C	-1.338003983192	-1.600716787869	1.494777066231
C	-1.201433454278	1.943414074406	-0.922331891350
C	-0.278813832037	3.022217049728	-0.711487312647
C	0.465229993458	3.141480748910	0.489347711490
C	1.356594263736	4.187762365936	0.670996524462
C	1.510917496462	5.169836062768	-0.319027442304
C	0.755595888832	5.105165937812	-1.483785662770
C	-0.146561873968	4.046740779204	-1.698692586430
C	-0.969358179414	3.995157112793	-2.861826704893
C	-1.885233450149	2.991790788466	-3.047778410606
C	-2.010628693476	1.960939152973	-2.091333341712
O	1.033652387938	0.152977259546	-0.545891748829
O	-2.863718782741	0.982524045481	-2.409330530589
H	-3.694623471060	-2.030338463060	2.719181230807
H	-5.667492652556	-0.529162335727	2.685518834431
H	-5.565130128633	1.627119603181	1.444563800665
H	-3.523289512425	2.273866557679	0.239462599811
H	0.706049371794	-1.878863484128	0.893216180056
H	-1.379487905104	-2.524641723746	2.065622083080
H	0.317735263140	2.417713784426	1.286209888452
H	1.923744203797	4.254432779316	1.594889245280
H	2.204434407470	5.991596751988	-0.166905170500
H	0.847517105878	5.881949314325	-2.238623711184
H	-0.868021901612	4.776274547660	-3.610886710685
H	-2.507318546136	2.947110459954	-3.935957417584
H	1.011287135646	0.982162569651	-1.056910710001
H	-2.886339464056	0.290714320224	-1.723982769103

20.xyz

34

-770.318641821

C	1.474057372343	2.258262945645	0.629321173766
C	2.412003063993	3.041128605101	1.354954038665
C	3.757753113383	2.977240749153	1.072158089778
C	4.223993002933	2.125785594107	0.042544547832
C	3.340378841968	1.351539088577	-0.676963201262
C	1.945408280435	1.386128948251	-0.405937614261
C	0.998360410960	0.597291638765	-1.137259882701
C	-0.347855738415	0.710610773395	-0.841774529361
C	-0.810099673175	1.574840077452	0.177876098748
C	0.082541419251	2.329622047540	0.902448657422
C	1.434367727744	-0.321473569635	-2.230112406749
C	2.055584008546	-1.580155086990	-1.940422890747
C	2.283969703067	-2.032141227664	-0.612233859357
C	2.881511841047	-3.249264800690	-0.368135119253
C	3.281846956953	-4.079842182122	-1.441604404138
C	3.067214258643	-3.676450278436	-2.740188911762
C	2.450313782554	-2.428662325162	-3.026079821966
C	2.209064022787	-2.011542707506	-4.361628727195
C	1.597020843149	-0.806504095069	-4.616265499344

C	1.210840309451	0.035278499003	-3.547385965979
H	2.044910177224	3.698346095165	2.140824321524
H	4.464237595228	3.582594023085	1.634776812811
H	5.287063635875	2.084466467511	-0.182126082048
H	3.708657067549	0.703666227219	-1.467581859016
H	-1.064772239693	0.113067814663	-1.400237835673
H	-1.875558463211	1.633816715478	0.386138471772
H	-0.265535622842	2.993551541174	1.691083479282
H	1.976619941278	-1.404054877344	0.219331226770
H	3.045679781784	-3.576000967278	0.655877484351
H	3.754123551224	-5.037627639010	-1.237332390377
H	3.365946362917	-4.312667230846	-3.571074780507
H	2.512646404381	-2.662263010248	-5.179102359854
H	1.409957515057	-0.490702153176	-5.639661795325
H	0.736618999321	0.989679005167	-3.764519297780

20-rc.xyz

34

-770.099520037

C	1.480612187654	2.199093921746	0.644542322712
C	2.411708209842	2.856022058533	1.485496612266
C	3.772436741222	2.681962509718	1.304735774380
C	4.237010506687	1.857826874802	0.266335344195
C	3.345468202972	1.189430576234	-0.560574055240
C	1.951152070788	1.312318236253	-0.376862340061
C	0.984586439127	0.667316217322	-1.231571718955
C	-0.372448635139	1.030507006402	-1.119671095937
C	-0.818271987249	1.885675573332	-0.113843437034
C	0.093402362154	2.450742476903	0.770273934941
C	1.323166559178	-0.351972782305	-2.220845239804
C	2.113517493909	-1.523208198125	-1.930991536489
C	2.453373538025	-1.916873786865	-0.618586993008
C	3.184854262824	-3.072762208187	-0.385860591626
C	3.599398973424	-3.886112181084	-1.453583201841
C	3.248229984962	-3.549340902391	-2.748949123330
C	2.488034689467	-2.383937958988	-3.012629730713
C	2.050583299974	-2.089834633177	-4.326326657555
C	1.220288303931	-1.003416753754	-4.576349020185
C	0.837094136723	-0.166061290876	-3.530403354203
H	2.039960566405	3.516409226240	2.264723619665
H	4.479327042139	3.194486763379	1.950560756415
H	5.304894902340	1.743609981498	0.102918815683
H	3.732010960505	0.576301343734	-1.368266414613
H	-1.097664334754	0.568039863419	-1.783033805554
H	-1.878479188062	2.097418155974	-0.015704076822
H	-0.251625349052	3.119846601817	1.554462046839
H	2.127176427950	-1.318547669816	0.226041410209
H	3.433667062823	-3.350548304922	0.634486455342
H	4.177507322387	-4.785064941346	-1.260696022296
H	3.535098646380	-4.188101877837	-3.580314136684
H	2.349332584859	-2.745097418012	-5.140723708650
H	0.878831592425	-0.796977611187	-5.585976587472
H	0.214632676889	0.698415836841	-3.743109076511

21.xyz

36

-6062.94502669

C	-1.419680289287	0.291278387757	3.092450301816
C	-2.133920722295	0.101023252023	4.301930573190
C	-1.560868171972	0.467791004722	5.495775296808
C	-0.270777125915	1.035605631819	5.548702246114
C	0.434713071788	1.228042886391	4.381997245728
C	-0.110683817746	0.865394312744	3.122744292805
C	0.610587198739	1.052433615155	1.900781772806
C	0.018274669389	0.664957037376	0.704491482598
C	-1.281701787267	0.101817574557	0.672889455866
C	-1.979127298158	-0.077994832917	1.839134230719
C	1.982439860516	1.638069824477	1.886308832329
C	3.144745837104	0.823605300973	2.071870292699
C	2.143398815830	3.005793057961	1.697966944416
C	3.429073348209	3.601535235746	1.674418995477
C	4.546656489136	2.827096948048	1.847677147109
C	4.441871788463	1.424833961739	2.054502684035
C	5.593988990566	0.621110274723	2.242828783847
C	5.455475970831	-0.731255767456	2.442972681444
C	4.185729818900	-1.345284811922	2.464030619309
C	3.057208378131	-0.577661189122	2.281061846885
O	0.641317296296	0.794493193321	-0.494582651922
O	1.092857608791	3.849206698154	1.530798058184
Br	6.992764550497	-1.811574642223	2.702163640049
Br	-2.506638049961	0.210077025413	7.119490641412
H	-3.128694649733	-0.334459756498	4.270647445473
H	0.160549696299	1.317509966586	6.504150945218
H	1.428880257817	1.663952806953	4.425854615298
H	-1.702000899358	-0.182830383309	-0.287405506025
H	-2.975849762679	-0.511926467380	1.813490036011
H	3.502227400219	4.674355337596	1.521048343336
H	5.532333658655	3.286063438489	1.831200640302
H	6.575926590428	1.085519660817	2.228455305813
H	4.101854837135	-2.415828458014	2.623965781041
H	2.080732204535	-1.053606028934	2.298755123859
H	1.531367627477	1.164774971466	-0.353367985087
H	0.263344004581	3.341713232621	1.589110009364

21-rc.xyz

36

-6062.72285997

C	-1.516484649124	0.402136584442	3.085172859541
C	-2.408362896511	0.468904428376	4.167922843010
C	-1.995596078695	1.029253140091	5.369637568000
C	-0.685423800957	1.504735393597	5.537108746048
C	0.204614456332	1.430288454161	4.483398648614
C	-0.190889539543	0.916941334360	3.220979759496
C	0.716318466639	0.849094356393	2.116621724499
C	0.282676170835	0.214067699390	0.921748566528

C	-1.027280154901	-0.305501349614	0.805382035416
C	-1.904151826652	-0.201408943189	1.850919562987
C	2.032480719529	1.494918201568	2.162751515182
C	3.240148260457	0.740873826509	2.022050215631
C	2.123242350189	2.909696695198	2.253273505290
C	3.371801136397	3.567504845729	2.164230058311
C	4.519806271840	2.843765607711	1.986683227840
C	4.493360885739	1.418149039907	1.915867683272
C	5.679223198810	0.679356874616	1.772419558134
C	5.629216121868	-0.708125993155	1.742686193288
C	4.413131474046	-1.394294236041	1.887349543867
C	3.242418720750	-0.677952809158	2.042867507862
O	1.027760951906	0.134152826451	-0.182983339057
O	1.059848839068	3.711774064868	2.341983921273
Br	7.219225162065	-1.688485953517	1.540033940036
Br	-3.199330948936	1.121377071699	6.809384909747
H	-3.414049127896	0.074252005708	4.061048089565
H	-0.368033600070	1.908100144293	6.493061029553
H	1.229284347552	1.756958453144	4.635910428308
H	-1.316753969138	-0.760507482831	-0.136367896580
H	-2.914174334028	-0.589286381021	1.750541029709
H	3.385408108115	4.651454461486	2.214823765922
H	5.474627992744	3.356030829860	1.904655461289
H	6.629417771726	1.198406561024	1.692412148219
H	4.395425347213	-2.478999927117	1.897246428882
H	2.312521133949	-1.216788480842	2.200245414460
H	1.908593935035	0.529577318394	-0.054528459157
H	0.228356499610	3.205213637364	2.371771973343

22.xyz

34

-956.615330804

C	-1.233013069657	2.109445124894	7.243513178665
C	-1.361203974248	3.484847996764	7.575641801242
C	-1.952150869168	4.371319480479	6.704157913812
C	-2.447264796293	3.915853545610	5.459469897208
C	-2.335636334494	2.589233836783	5.105734208848
C	-1.720564232980	1.649957781774	5.977155338134
C	-1.592592905459	0.260597107718	5.655552213539
C	-1.029589465616	-0.609935789091	6.569920060903
C	-0.549168098374	-0.149086182163	7.816924401859
C	-0.644109851785	1.183247006393	8.144187829916
C	-2.075313881846	-0.277324520669	4.353553123607
C	-1.359830669019	-0.009777356711	3.125596375535
C	-0.173891239937	0.744095454853	3.039059609557
C	0.461908012370	0.949499876863	1.827908134811
C	-0.079942812125	0.394672262380	0.654070814993
C	-1.246221048125	-0.366427516059	0.702719516756
C	-1.865684447511	-0.560287600638	1.931522907993
C	-3.694832290595	-1.620942097101	3.079192378797
C	-3.187971291200	-1.063128268408	4.311890239104
O	0.496938115054	0.564192179432	-0.560676097989
O	-4.685289989923	-2.321522037105	2.960559158972

```
O        -2.995995606933     -1.324082370550      1.926548203571
H        -0.986461786916      3.825196568368      8.538716572133
H        -2.045849653271      5.420893470187      6.971248212329
H        -2.922633658819      4.618323135039      4.779437153562
H        -2.728538492490      2.252991205295      4.150077757745
H        -0.942936310154     -1.664321158884      6.319221749984
H        -0.103402000554     -0.856350221721      8.511395132497
H        -0.274709376134      1.544754896415      9.101461928526
H         0.248949954282      1.170652403443      3.943773543085
H         1.376366503144      1.535756173100      1.781354663207
H        -1.662568050382     -0.804021968159     -0.198563585733
H        -3.757235386288     -1.290563645352      5.206568691057
H         1.298051479536      1.105113813678     -0.479427622664
```

22-rc.xyz

```
34
-956.385897518
C        -1.378979445091      2.140677839747      7.391977318421
C        -1.888799677353      3.250873443875      8.102974449072
C        -2.867695595339      4.069329990901      7.545081642430
C        -3.345015829411      3.806943134461      6.257240332182
C        -2.853143506435      2.724766440514      5.528242173717
C        -1.891304960924      1.850125233154      6.087277351931
C        -1.381084029058      0.714285439032      5.389696072768
C        -0.354044885664     -0.056543799902      5.961694177154
C         0.150126647270      0.245760839907      7.226565595870
C        -0.360126440029      1.326153837474      7.939442282032
C        -1.973021871832      0.257396957955      4.119341900858
C        -1.182587262895      0.040047156972      2.944150567295
C         0.168640316224      0.437017495332      2.804225848217
C         0.872321803388      0.178956942878      1.649735458892
C         0.235261802200     -0.488062686067      0.579220721442
C        -1.108653962106     -0.866844989512      0.668812300951
C        -1.798578616106     -0.593302137529      1.835150303465
C        -3.920983945774     -0.713955923378      2.944729338090
C        -3.304418256619     -0.087872218710      4.102730919194
O         0.867811360035     -0.770905147888     -0.568660978741
O        -5.086434077648     -1.042791626196      2.868617173544
O        -3.108680712671     -0.962697207200      1.864821729391
H        -1.503788703984      3.462895648114      9.096861171803
H        -3.248158978531      4.917206096799      8.106507474595
H        -4.092735779584      4.455570805405      5.810843452099
H        -3.195856804836      2.574129240546      4.509332048829
H         0.012865239624     -0.929808508068      5.430027687890
H         0.925197721161     -0.377086244457      7.661936368446
H         0.025459730975      1.554830353591      8.929488301217
H         0.654337338244      0.968849709729      3.616667795522
H         1.906395222257      0.498017584755      1.552344652933
H        -1.601657071932     -1.357024873845     -0.163677693790
H        -3.938445601867      0.023532445101      4.975007432953
H         1.793391308400     -0.477598686633     -0.538939965111
```

23.xyz

```
32
```

-5836.34887967

C	3.197954063927	0.062218736025	2.547953900217
C	4.063209155840	0.476226456754	1.536668659887
C	3.580009512512	0.643557798570	0.238736099227
C	2.237418641118	0.389800108835	-0.037249343214
C	1.358490186682	-0.020281699606	0.972781305679
C	1.848860274176	-0.200739163196	2.280217686822
C	0.955017511085	-0.638944427782	3.390832722205
C	-0.071118369596	-0.284458135933	0.639337571900
C	-0.037816255678	-2.393963423418	4.771030602556
C	0.772462516214	-1.989475831793	3.712044653701
C	0.268720563101	0.305422587746	4.167947702077
C	-0.555434890597	-0.080569874152	5.223134948681
C	-0.711444755326	-1.433697292006	5.523667529068
C	-0.483035972251	-1.561375360028	0.231275659129
C	-1.049996794278	0.714281816556	0.708679511468
C	-2.379273131120	0.470871824597	0.368825392579
C	-2.761263470211	-0.809203167961	-0.028571054895
C	-1.810183960600	-1.827017977709	-0.100074442448
Br	1.723659407065	-3.326326484483	2.751053677936
Br	-0.555842727308	2.488461790617	1.181576021057
H	3.571284511696	-0.070227634865	3.560859080904
H	5.108080365937	0.672196536156	1.764037753861
H	4.245268332366	0.965292541549	-0.558628181408
H	1.857021403949	0.521065332084	-1.047707178829
H	-0.145103649442	-3.449632453118	5.000954591675
H	0.395350566277	1.358958443150	3.934307194939
H	-1.071896537731	0.675326120630	5.808759262715
H	-1.348260184727	-1.748038551574	6.346420378509
H	0.260452884036	-2.351512005244	0.172020293217
H	-3.109089702221	1.273128321600	0.420008706637
H	-3.798970602001	-1.002145189988	-0.287827737993
H	-2.097873155551	-2.826638661204	-0.414841298842

23-rc.xyz

32

-5836.10806851

C	3.122529682334	0.379928449658	2.590147004316
C	4.013024343885	0.641050658621	1.559148945145
C	3.614647797144	0.476231144903	0.218984664134
C	2.321080483188	0.075393555226	-0.081468935583
C	1.390381559296	-0.145983896197	0.944618182738
C	1.824537782242	-0.072158351586	2.309383440410
C	0.949992852044	-0.482280098000	3.414832202508
C	-0.009493700039	-0.424119556718	0.600072679894
C	-0.560398072189	-2.087348651088	4.510731418613
C	0.332161449216	-1.760177602592	3.483823437941
C	0.703432339442	0.412975993285	4.470494609485
C	-0.182154450074	0.094677821715	5.487826363873
C	-0.830959413594	-1.155160749858	5.499336601706
C	-0.309240247938	-1.487650409197	-0.269217169498
C	-1.099128368682	0.339375081755	1.098886162899
C	-2.425123662291	-0.004748307122	0.813533674015

C	-2.685115763219	-1.101159781443	0.007397489385
C	-1.620904431527	-1.835115985522	-0.550954958894
Br	0.937578508980	-3.158394863016	2.385759824519
Br	-0.791431090283	2.015293440167	1.889671020980
H	3.449578621793	0.449551643423	3.623560282688
H	5.029442557835	0.948110526733	1.787728073262
H	4.314566720703	0.688795246875	-0.583809474265
H	1.999011199375	0.004908958453	-1.116423525964
H	-1.012582650108	-3.073243256213	4.538370357082
H	1.168356343077	1.393707269495	4.440854019807
H	-0.396754760373	0.823741467051	6.263501422580
H	-1.523009396289	-1.402719051604	6.298776289081
H	0.508361728142	-2.082478439072	-0.664853036921
H	-3.236030025013	0.600628537760	1.204829516926
H	-3.712293530029	-1.374645754069	-0.215242025162
H	-1.827408669708	-2.694423961011	-1.182066888684

24.xyz

36

-1102.9885791

C	0.366194253336	6.470283561787	1.313757192031
C	0.173471529150	7.256133214230	0.179982102287
C	-0.013589256183	6.643910640298	-1.058594334161
C	-0.021768001132	5.253724293212	-1.144711888356
C	0.158794507017	4.448110489721	-0.010269805844
C	0.372441678525	5.069176293302	1.241714531054
C	0.651921445001	4.304409101432	2.487493138447
C	0.066962046573	2.971899486236	-0.175788831119
C	0.221299829878	3.875081483400	4.819591225626
C	-0.081873132499	4.566484576174	3.648748386672
C	1.683238266813	3.352109988470	2.546863464706
C	1.972631433244	2.673725906594	3.730359745740
C	1.239241416980	2.926525131957	4.886322638532
C	-0.818871006745	2.202823955613	0.597514420160
C	0.829288069293	2.327284888017	-1.155010886392
C	0.698245372600	0.951014867106	-1.327542574311
C	-0.173995498595	0.180387709321	-0.562044138997
C	-0.937410029735	0.826711256237	0.406014519201
N	-0.563384540051	4.155585110883	6.022229647591
N	1.510780046871	0.292988666052	-2.350950980504
O	1.397113011079	-0.922917154788	-2.485977116561
O	-1.455651925594	4.997236050378	5.949721692766
O	-0.291792397593	3.535354613850	7.047439575595
O	2.267307634992	0.986975270525	-3.026114120452
H	0.546016094089	6.948173265534	2.273944279174
H	0.180085898168	8.339682094343	0.264494447331
H	-0.163183019415	7.242808624811	-1.953136834382
H	-0.202319761835	4.776682238008	-2.105100552690
H	-0.891211081394	5.288292143987	3.644739251807
H	2.276060289003	3.151811180038	1.658748560831
H	2.779791319072	1.947363756948	3.754187572896
H	1.447638866612	2.410004816149	5.816072773837
H	-1.433928288826	2.691130020952	1.348141685494

```
 H         1.529784995102      2.881412677195     -1.770109258047
 H        -0.250641451564     -0.888101291761     -0.726601956453
 H        -1.634235952780      0.254367625781      1.011372426353
```

24-rc.xyz

```
36
-1102.74093340
 C         0.097427196762      6.442052700360      1.364377945462
 C         0.036657187845      7.211410479763      0.218477342945
 C         0.120625572179      6.603689631450     -1.057600558236
 C         0.239408366071      5.231652359011     -1.172355789607
 C         0.254443806262      4.415345062698     -0.025749114049
 C         0.267915487211      5.046541605698      1.283196166111
 C         0.560648458532      4.297799118260      2.499866719438
 C         0.148319138749      2.969412217034     -0.185755881885
 C         0.232613422067      3.943056728797      4.858528919655
 C        -0.077994504697      4.646881615216      3.711312140054
 C         1.531615331117      3.263245001612      2.516434515853
 C         1.842027336279      2.595450289360      3.694036585826
 C         1.186167140980      2.919956359524      4.880176333890
 C        -0.683545725301      2.190973889432      0.659390223250
 C         0.829113805474      2.328672746084     -1.244654621197
 C         0.695470423803      0.961999446713     -1.396252552679
 C        -0.121192891136      0.188398038785     -0.565251495739
 C        -0.819668437434      0.823019168806      0.460416948249
 N        -0.467809366207      4.286404110007      6.105655486076
 N         1.440515689452      0.298683143737     -2.477134967134
 O         1.294865429845     -0.910603040977     -2.604991710498
 O        -1.307455599014      5.177315473977      6.061518159929
 O        -0.167212532317      3.659354627487      7.113794994582
 O         2.159651882188      0.994057285550     -3.184259649781
 H         0.109491714605      6.928603935813      2.334359519381
 H        -0.042375529677      8.291495495533      0.295452293737
 H         0.057464107890      7.217887491544     -1.950657720666
 H         0.225691418897      4.774540624205     -2.156586125854
 H        -0.838966466345      5.417832015582      3.746250568046
 H         2.080304868803      3.026281622559      1.611312509743
 H         2.603513533240      1.822348836108      3.693931661826
 H         1.406462742300      2.403427562341      5.807520232152
 H        -1.264917574762      2.676104793674      1.436330585257
 H         1.488863445668      2.879904852707     -1.904662536757
 H        -0.205994619923     -0.879732926102     -0.730031917945
 H        -1.477691599947      0.245184189643      1.101104790430
```

25.xyz

```
50
SCF = -1154.87796159
 C         2.564858655928      1.754381628561     -0.469488469467
 C         2.615333008772      2.557992311394     -1.640932908284
 C         1.754153076080      2.341634457834     -2.693348304482
 C         0.793711655648      1.306943915687     -2.622778480719
 C         0.722326763727      0.504611770828     -1.506034476657
```

```
 C          1.602180862296      0.692258447896     -0.405357411906
 C          1.543406670798     -0.140481124321      0.758108095001
 C          2.415198972646      0.104461134139      1.800436036504
 C          3.360813679235      1.151142762995      1.738279436188
 C          3.448567007245      1.976450297446      0.635067135626
 C          4.470244923984      3.065057693556      0.609755496838
 C          0.541099152643     -1.240336077827      0.879912963598
 C          0.726400813025     -2.495342629131      0.214144008528
 C          1.863827518505     -2.771959443000     -0.591939523160
 C          2.013587632655     -3.991472160142     -1.215261081690
 C          1.031978330895     -4.999544050755     -1.063897240120
 C         -0.078886670017     -4.766586977972     -0.284930894060
 C         -0.262204395288     -3.520913658055      0.373677307953
 C         -1.397784231603     -3.275445215778      1.189771318215
 C         -1.548806535488     -2.067430955435      1.829728243691
 C         -0.576126744572     -1.052791065508      1.672869209915
 C          5.131131011987      5.431305290715      0.869125940808
 C          4.776919533889      6.779026116279      1.147141303850
 C          3.475597991590      7.125012005919      1.433635610845
 C          2.468271177343      6.131394273311      1.456514332401
 C          2.778939633394      4.816100499173      1.189069439397
 C          4.111096843192      4.424552540258      0.885031562168
 C          5.791382073157      2.752982480924      0.347474119256
 C          6.795605341893      3.748865274656      0.332377996446
 C          6.472259477338      5.061644294277      0.584805466214
 H          3.350874650800      3.355728447523     -1.697274839383
 H          1.810866431201      2.968792929149     -3.579725116671
 H          0.110848313541      1.144281718370     -3.453095097512
 H         -0.017970609845     -0.289126451531     -1.456803504971
 H          2.376711448790     -0.523611481319      2.687482784563
 H          4.027975400226      1.313724623634      2.581624401235
 H          2.624365396636     -2.005109656131     -0.711406288907
 H          2.892052449201     -4.182626479894     -1.826870027403
 H          1.160132909282     -5.957931337154     -1.561074451066
 H         -0.835306088547     -5.538864232989     -0.159531820883
 H         -2.143460307083     -4.059638177503      1.303292926720
 H         -2.417252716398     -1.882946233958      2.457280516426
 H         -0.712759356222     -0.100084712622      2.179431645997
 H          5.557692069136      7.536939854253      1.132287375370
 H          3.218965137182      8.160037078087      1.645768723665
 H          1.442814529826      6.409258314938      1.687952360463
 H          1.999062373587      4.059829796163      1.212260460166
 H          6.061225406155      1.719670465776      0.141602427077
 H          7.823844585596      3.467821825784      0.118355765279
 H          7.239587656325      5.833027585599      0.573527347877
```

25-rc.xyz

```
50
SCF= -1154.6520724
C          2.629946542116      1.711234040742     -0.300011032943
 C          2.709148679839      2.484125720419     -1.482397511956
 C          1.834329693177      2.277294023125     -2.534089468654
 C          0.837173096996      1.293378918559     -2.435553600839
```

C	0.766244144949	0.488118902365	-1.312723112193
C	1.680313839261	0.637994533109	-0.243431232937
C	1.648524595401	-0.223391724322	0.907494439925
C	2.514799191633	0.048814882161	1.978470020971
C	3.448908944748	1.073723185059	1.913988662686
C	3.499965365768	1.950558802753	0.818938626664
C	4.446760551002	3.072348240947	0.892901406323
C	0.749772780858	-1.378553260013	1.039903970959
C	0.692638178394	-2.460347594632	0.091514136507
C	1.616234779268	-2.607128083114	-0.971880105068
C	1.538905935617	-3.680228488938	-1.839499248970
C	0.538592782504	-4.661217959693	-1.682136789210
C	-0.355757465431	-4.566196871010	-0.636380292246
C	-0.291519374321	-3.484107099352	0.278623196626
C	-1.161768092418	-3.425873506391	1.395814784474
C	-1.045738579351	-2.415712099979	2.332644375835
C	-0.078201970133	-1.414965620179	2.166462798891
C	5.074508488864	5.457500557654	0.773871216559
C	4.708811939903	6.823864620148	0.664433810191
C	3.383572197544	7.195609593377	0.576964794686
C	2.376001370119	6.209747546516	0.609046722824
C	2.703183651219	4.870201690540	0.701388177687
C	4.054788708465	4.451472209321	0.759601872323
C	5.789057817212	2.764049463523	1.132908223354
C	6.780001309682	3.755930303361	1.129549743797
C	6.430740996798	5.079844336780	0.938354277761
H	3.469288593132	3.254031449557	-1.563709286243
H	1.913077293497	2.886302576140	-3.430121333478
H	0.126744539353	1.154062023707	-3.245391426841
H	-0.003750329542	-0.273611736377	-1.251753718783
H	2.486293546733	-0.589077131581	2.857282865011
H	4.117931189053	1.235910024345	2.754386082509
H	2.406492503985	-1.874266843951	-1.103127022454
H	2.260385513511	-3.769984291609	-2.647174472013
H	0.485151935959	-5.497269434136	-2.373922796378
H	-1.113581849947	-5.331741749874	-0.488019302735
H	-1.910398342147	-4.204640885038	1.519735456498
H	-1.707407771353	-2.385545791663	3.193329839794
H	-0.015851201027	-0.611948518020	2.896199449342
H	5.493392978059	7.576721967394	0.669665719777
H	3.113551003345	8.245142597694	0.499964611154
H	1.330996926505	6.504881371842	0.566676514367
H	1.908828386249	4.131357026346	0.743625932096
H	6.078723314242	1.724446529920	1.261203285009
H	7.820296847014	3.476734785654	1.268476854250
H	7.195247733982	5.853094480917	0.935941706031

1.xyz

37

SCF = -826.0871786

C	-0.017158398199	0.127949119517	0.009009120842
C	-0.032806592427	0.064791421192	1.426772110268
C	1.210031121776	0.050756353321	2.137970824864

C	2.420707917282	0.097907710307	1.398871733725
C	2.401358305698	0.159084911825	0.022919703132
C	1.173999812903	0.174861077553	-0.679317927776
C	-1.253420210907	0.011190346086	2.149418194883
C	-1.257753498056	-0.054602063723	3.520765260021
C	-0.022623525833	-0.065332341859	4.198496135432
C	1.202678737464	-0.013288197031	3.568686922664
C	2.475158273339	-0.040501853772	4.351374087266
C	3.013684300045	-1.257692924067	4.733617130033
C	4.219952856382	-1.324326105405	5.468072907947
C	4.881048360623	-0.168853747606	5.813546108771
C	4.366323558603	1.101697289027	5.444415947373
C	3.143404911171	1.178152381526	4.701535575205
C	5.037481671525	2.303110224494	5.795827276368
C	4.527263704600	3.529106500452	5.433565355075
C	3.319077684566	3.605397213289	4.701442153624
C	2.643220095906	2.459243570156	4.344549700452
N	-0.053276302670	-0.125274078769	5.672521688781
H	-0.964229467431	0.137793573054	-0.525174085181
H	1.174848865811	0.222784478575	-1.764954634348
H	3.336976546264	0.194960801033	-0.528768125459
H	3.368044143615	0.083321577537	1.929581966216
H	-2.192094959172	-0.096199742179	4.074684079042
H	-2.193645229565	0.021187406879	1.604582080620
H	1.718751825364	2.531639307124	3.777546154410
H	2.923082095428	4.576846221233	4.416669764035
H	5.050626833238	4.441430042290	5.707381955807
H	5.966094338968	2.236468405182	6.358447299556
H	5.810408771033	-0.214259398816	6.376803709806
H	4.620060440145	-2.293972879003	5.751255127081
H	2.506963832689	-2.178356490600	4.452490844805
H	-0.544673254061	-0.956991442821	6.019030640426
H	0.906393021994	-0.162895797372	6.043666250000
H	-0.512722360115	0.693944632318	6.086397852265

2.xyz

43

SCF = -904.66849836

C	-0.111057833999	0.076281835774	-0.055537085598
C	-0.088246320027	-0.031302909866	1.323037290218
C	1.169435698108	-0.088409567098	2.008506109425
C	2.376542482809	-0.076797297917	1.236806735620
C	2.301216201528	0.009439488336	-0.178263045019
C	1.083537491135	0.091055194057	-0.811552473559
C	3.626919233567	-0.143764059115	1.907707849699
C	3.689868455803	-0.212261615856	3.280885984970
C	2.498861182751	-0.215191635781	4.044501792142
C	1.270342981597	-0.155972719221	3.424102981492
C	-1.370770617659	0.013592555846	2.093407400460
C	-1.816318866591	1.288509373388	2.589849157512
C	-3.038735376723	1.390641431404	3.327794806158
C	-3.803022563943	0.222683368823	3.562344933246
C	-3.379399346394	-0.989962867095	3.083653040356

C	-2.171790002325	-1.085406929554	2.354985068330
C	-1.070138146558	2.476728185610	2.361357826606
C	-1.509276025832	3.691134424962	2.839921662962
C	-2.716194636071	3.785826767750	3.570795589368
C	-3.464636365861	2.656641174773	3.808542836879
N	-1.864709836419	-2.467101438934	1.885672750451
C	-2.106828908165	-2.673708937218	0.415747710649
C	-0.542397870855	-3.031040758137	2.319648579766
H	-4.395494699059	2.716616674277	4.367526019927
H	-3.049543462846	4.751430789399	3.941534604016
H	-0.922551967263	4.586574320233	2.652600896681
H	-0.143957068957	2.425275531671	1.798311975780
H	-3.977383266621	-1.880281993379	3.267003650567
H	-4.730080537607	0.291993599315	4.124842367226
H	0.363365210831	-0.154174224855	4.023078210209
H	2.554903855907	-0.262098765425	5.129090458944
H	4.652840200951	-0.260888230395	3.782470949820
H	4.538253164937	-0.136803830702	1.313704322591
H	3.224182930247	0.020384583851	-0.753600123720
H	1.029235176052	0.172678134253	-1.893747388271
H	-1.064818188076	0.171276049624	-0.569467190736
H	0.256145811298	-2.540519280053	1.769459546101
H	-0.431576324265	-2.865476384032	3.391039879837
H	-0.552139785725	-4.099551990133	2.099384290301
H	-2.086825361366	-3.746545930027	0.219353004839
H	-1.320589609889	-2.170079495633	-0.141600347439
H	-3.083580471563	-2.257997220017	0.167944279241
H	-2.562800273800	-3.051000514762	2.354962585488

2-rc.xyz

43

SCF= -904.422421327

C	-0.125657133193	0.276256610930	-0.012853798796
C	-0.078765295724	0.027479544433	1.371998046984
C	1.183904512666	-0.129202113481	2.011570302106
C	2.372327757886	-0.119841181071	1.215707297900
C	2.276949850843	0.066671898134	-0.180089861529
C	1.034058500323	0.272338701384	-0.784498740375
C	3.627841156371	-0.262741142251	1.849677351409
C	3.721091236743	-0.397746477962	3.240210531747
C	2.566208854398	-0.381803777083	4.018921391416
C	1.309777445639	-0.242594658513	3.416558663588
C	-1.350877248215	0.028491025538	2.150484039089
C	-1.835808381060	1.307610833946	2.584330487179
C	-3.087094743897	1.404685136743	3.270642696491
C	-3.823325860894	0.227329855227	3.530722551216
C	-3.350568051317	-0.995261558724	3.118516642005
C	-2.124016455865	-1.093211747197	2.431446618246
C	-1.094031823278	2.500551918507	2.364789067917
C	-1.586948646478	3.725226012178	2.772857317791
C	-2.825480281841	3.816674982584	3.438773087148
C	-3.558162035099	2.674650272888	3.689741036908
N	-1.812216466798	-2.470900568938	1.958777107276

C	-2.051128396931	-2.661306677184	0.482837846599
C	-0.497137731582	-3.055174271259	2.390692486040
H	-4.505681364826	2.732130563809	4.219043058218
H	-3.196766192324	4.784907031189	3.761533846006
H	-1.007489880544	4.624624797382	2.585458005246
H	-0.124288485531	2.458794749246	1.879609096142
H	-3.938028125696	-1.889501283425	3.312340005222
H	-4.771733666682	0.291503091797	4.056157014056
H	0.421444631863	-0.206435614150	4.040739410365
H	2.633352398295	-0.470730065886	5.098696955896
H	4.695232527780	-0.503885461182	3.706888542120
H	4.530151453978	-0.254642078199	1.244446144962
H	3.181673217617	0.068309232373	-0.781831128072
H	0.971459476268	0.432847604421	-1.856054301716
H	-1.087342584649	0.453133478947	-0.485965492602
H	0.309290243110	-2.592647920672	1.828478889946
H	-0.377661697530	-2.892225751044	3.461475273378
H	-0.528365912341	-4.123685122662	2.174104227917
H	-2.038148614079	-3.732230256980	0.278370877180
H	-1.257781966915	-2.163364212111	-0.070940847959
H	-3.023471909044	-2.236499844584	0.233971958821
H	-2.515997968387	-3.056160668957	2.419475790053

3.xyz

38

SCF = -884.537496

C	3.039341821666	-0.920477478227	-3.364400590990
C	3.326226024123	-0.317247960813	-4.618103102379
C	3.023028192326	1.004329217179	-4.853670711488
C	2.415198327308	1.777397973317	-3.834594083229
C	2.126323748317	1.222507511357	-2.607675287060
C	2.429385649699	-0.140586334629	-2.327867217681
C	2.141969428478	-0.739514193155	-1.065503324398
C	2.452735679242	-2.081345878601	-0.861458092454
C	3.054989748982	-2.852576936484	-1.887444357892
C	3.339131815034	-2.279256303308	-3.105162822311
C	1.533081952944	0.057469284409	0.040479569113
C	0.113512025305	0.105938933784	0.218392662808
C	-0.785224949417	-0.583129944780	-0.640038802699
C	-2.147201575057	-0.517208476394	-0.444054346235
C	-2.681707700288	0.243976832563	0.622871276361
C	-1.838932445564	0.925820243648	1.471459363294
C	-0.429556542146	0.879244378785	1.295792303731
C	0.449472171483	1.581749681935	2.161325098989
C	1.810464174269	1.524787394684	1.968803776726
C	2.348114166251	0.761537909076	0.906597404317
O	2.149507040370	-2.594814646260	0.361432061094
C	2.438539855902	-3.959976131936	0.628492095827
H	3.791865099949	-0.923506913701	-5.392901329358
H	3.246518998215	1.456045028142	-5.816923047286
H	2.173298218554	2.820661659195	-4.024029494905
H	1.661120306868	1.828118955318	-1.834928661259
H	3.296908551996	-3.896367754068	-1.719154931395
H	3.802828407244	-2.877654935201	-3.886607542791

H	-0.381168446635	-1.168905053773	-1.461677424334
H	-2.817672349918	-1.052247293468	-1.112402142325
H	-3.758264543404	0.288743695558	0.768730940080
H	-2.242607145955	1.514429023542	2.293031101945
H	0.029287780504	2.166776505404	2.977039444519
H	2.481623093357	2.065334577087	2.631888583835
H	3.426395465305	0.724948317381	0.768187405921
H	2.109280879273	-4.133692732845	1.654045054566
H	1.886888794114	-4.625468640908	-0.046394271395
H	3.513609729938	-4.163526849268	0.554163063654

3-rc.xyz

38

SCF = -884.319168

C	3.039341821666	-0.920477478227	-3.364400590990
C	3.326226024123	-0.317247960813	-4.618103102379
C	3.023028192326	1.004329217179	-4.853670711488
C	2.415198327308	1.777397973317	-3.834594083229
C	2.126323748317	1.222507511357	-2.607675287060
C	2.429385649699	-0.140586334629	-2.327867217681
C	2.141969428478	-0.739514193155	-1.065503324398
C	2.452735679242	-2.081345878601	-0.861458092454
C	3.054989748982	-2.852576936484	-1.887444357892
C	3.339131815034	-2.279256303308	-3.105162822311
C	1.533081952944	0.057469284409	0.040479569113
C	0.113512025305	0.105938933784	0.218392662808
C	-0.785224949417	-0.583129944780	-0.640038802699
C	-2.147201575057	-0.517208476394	-0.444054346235
C	-2.681707700288	0.243976832563	0.622871276361
C	-1.838932445564	0.925820243648	1.471459363294
C	-0.429556542146	0.879244378785	1.295792303731
C	0.449472171483	1.581749681935	2.161325098989
C	1.810464174269	1.524787394684	1.968803776726
C	2.348114166251	0.761537909076	0.906597404317
O	2.149507040370	-2.594814646260	0.361432061094
C	2.438539855902	-3.959976131936	0.628492095827
H	3.791865099949	-0.923506913701	-5.392901329358
H	3.246518998215	1.456045028142	-5.816923047286
H	2.173298218554	2.820661659195	-4.024029494905
H	1.661120306868	1.828118955318	-1.834928661259
H	3.296908551996	-3.896367754068	-1.719154931395
H	3.802828407244	-2.877654935201	-3.886607542791
H	-0.381168446635	-1.168905053773	-1.461677424334
H	-2.817672349918	-1.052247293468	-1.112402142325
H	-3.758264543404	0.288743695558	0.768730940080
H	-2.242607145955	1.514429023542	2.293031101945
H	0.029287780504	2.166776505404	2.977039444519
H	2.481623093357	2.065334577087	2.631888583835
H	3.426395465305	0.724948317381	0.768187405921
H	2.109280879273	-4.133692732845	1.654045054566
H	1.886888794114	-4.625468640908	-0.046394271395
H	3.513609729938	-4.163526849268	0.554163063654
